# Characterisation of MRSA strains isolated from patients in a hospital in Riyadh, Kingdom of Saudi Arabia

**DOI:** 10.1186/1471-2180-12-146

**Published:** 2012-07-23

**Authors:** Stefan Monecke, Leila Skakni, Rami Hasan, Antje Ruppelt, Sameeh S Ghazal, Ahmed Hakawi, Peter Slickers, Ralf Ehricht

**Affiliations:** 1 Institute for Medical Microbiology and Hygiene, Technical University of Dresden, Dresden, Germany; 2 Alere Technologies GmbH, Jena, Germany; 3 Molecular Pathology Laboratory, King Fahad Medical City, PO Box 59046, Riyadh, 11525, Kingdom of Saudi Arabia; 4 Infection Control & Environmental Health Department King Fahad Medical City, PO Box 59046, Riyadh, 11525, Kingdom of Saudi Arabia

**Keywords:** *Staphylococcus aureus*, MRSA, Panton-Valentine leukocidin, Saudi-Arabia

## Abstract

**Background:**

Methicillin-resistant *Staphylococcus aureus* (MRSA) is spreading worldwide and poses a serious public health problem, being present in hospital settings and communities. However, from the Middle East and the Arabian Peninsula few molecular typing data on MRSA strains are currently available. In order to obtain data on the population structure of MRSA in Riyadh, Saudi Arabia, 107 clinical and environmental MRSA isolates were genotyped using a microarray-based assay.

**Results:**

Five major MRSA strains from four clonal complexes were identified CC8/ST239-III (20.75%), PVL-positive as well as -negative CC22-IV (18.87% and 9.43%, respectively), PVL-positive CC30-IV (12.26%) and PVL-positive CC80-IV (17.92%). Minor strains, which accounted for less than 3% each, included CC1-IV/SCC*fus*, PVL-positive CC1/ST772-V, PVL-positive as well as- negative CC5-IV, CC5-IV/SCC*fus*, CC5-V, CC6-IV, CC45-IV, PVL-negative CC80-IV, PVL-positive CC88-IV, CC97-V and a CC9/ST834-MRSA strain.

**Conclusions:**

Typing of MRSA strains from Riyadh revealed a high diversity of clonal complexes. The prevalence of the genes encoding the Panton-Valentine leukocidin was surprisingly high (54.21%), and a significant rate of resistance markers was detected also in strains considered as community-associated.

## Background

Methicillin-resistant *Staphylococcus aureus* (MRSA) infections remain a major healthcare burden considering the emergence of more virulent community-acquired or -associated MRSA (CA-MRSA) in addition to the longer existent hospital-acquired (HA-) MRSA strains.

While an abundance of MRSA typing data from the United States, Western Europe and Australia are available, comparable data for the Middle East are generally scarce. With regard to HA-MRSA strains, the pandemic strain ST239-III appears to be widespread in the region [1-5]. That strain was reportedly common in Saudi Arabia during the 1990s [6]. Another pandemic strain, CC22-IV (UK-EMRSA-15) has been detected in Kuwait [7] and Abu Dhabi [2]. Studies in various hospitals and several countries indicated an increased number of CA-MRSA infections confirmed by strain typing data. PVL-positive strains, which are usually regarded as community-associated, have been found in Kuwait [8], Abu Dhabi [2], Lebanon [9], Egypt [10], Tunisia [11], Algeria [12,13] as well as in people travelling from and to various Middle Eastern countries [14].

In Riyadh, the capital of the Kingdom of Saudi Arabia, an increasing number of MRSA cases has been detected since the application of an infection control policy requiring a systematic MRSA screening of patients prior to admission in hospitals in 2008 [15,16]. The MRSA prevalence in patients seen in King Fahad Medical City in Riyadh was 50.4% for the year 2011 (unpublished internal statistics, based on susceptibility tests of isolates from diagnostic samples), and thus it is within a similar order of magnitude to other hospitals in Saudi Arabia [17]. According to an earlier one year study (2005) performed in a hospital in the Western region of Saudi Arabia [18], the MRSA prevalence was 38.9% of which 78.8% showed resistance to erythromycin, gentamicin and oxytetracycline. The prevalence of CA-MRSA in a hospital in the Eastern region increased by six-fold during a 5-year period, between 2000 and 2008 [19].

To obtain the first MRSA typing data concerning Saudi Arabian patients, one hundred and seven MRSA isolates from King Fahad Medical City (KFMC) in Riyadh were characterised using DNA microarrays.

## Results

Altogether, 102 patient isolates were analysed for this study. Detailed data on patients' demographics and the origin of samples are provided as Additional file 1.

For ten isolates, clinical and patient data could not be retrieved; for two additional isolates, patient data were not available. Out of the ninety isolates for which demographic data were available, 57 (63.33%) originated from male patients and 33 (36.67%) from females. The mean age of patients was 26, the median 22 years. Patients carrying ST239-III were older than average (mean, 43 years; median, 39 years).

Additionally, five isolates from four environmental samples collected by the Infection Control & Environmental Health Department (IC & EH) were included. This included two PVL-negative CC22-IV, two PVL-positive CC22-IV and one PVL-positive CC30-IV.

### Prevalence of resistance- and virulence-associated genes

Table 1 shows which percentages of clinically important genes, i.e.*,* resistance or virulence associated markers, SCC*mec* elements and *agr* groups were found among the studied isolates.

**Table 1:** Prevalences of resistance markers and virulence-associated genes

**Marker**	**Number of positive isolates**	**Percent of positive isolates**	**Marker**	**Number of positive isolates**	**Percent of positive isolates**
*mecA*	107	100.00	*lukF-PV + lukS-PV*	58	54.21
SCC*mec* I, SCC*mec* II	0	0.00	*tst1*	8	7.48
SCC*mec* III	22	20.56	*sea*	9	8.41
SCC*mec* IV	76	71.03	*sea-N315*	5	4.67
SCCmec IV/SCCfus (CC1)	1	0.93	*seb*	2	1.87
SCCmec IV/SCCfus (CC5)	3	2.80	*sec + sel*	3	2.80
SCC*mec* V	4	3.74	*sed*	2	1.87
atypical SCC*mec* (ST834)	1	0.93	*see*	0	0.00
*merA + merB*	14	13.08	*egc*	54	50.47
*blaZ*	100	93.46	*seh*	1	0.93
*erm*(A)	21	19.63	*sej + ser*	3	2.80
*erm*(C)	30	28.04	*sek + seq*	24	22.43
*msr*(A)	9	8.41	ORF CM14	1	0.93
*mph*(C)	7	6.54	*etA, etB, edinC*	0	0.00
*aacA-aphD*	37	34.58	*etD*	21	19.63
*aadD*	8	7.48	*edinA*	1	0.93
*aphA3 + sat*	38	35.51	*edinB*	21	19.63
*dfrA*	28	26.17	ACME	0	0.00
*far1*	17	15.89	*sak*	103	96.26
Q6GD50 (*fusC*)	7	6.54	*chp*	70	65.42
*tet*(K)	11	10.28	*scn*	104	97.20
*tet*(M)	22	20.56	*agr* group I	58	54.21
*cat*	1	0.93	*agr* group II	10	9.35
*qacA*	20	18.69	*agr* group III	38	35.51
*mupA, ermB, cfr, fexA, vanA*	0	0.00	*agr* group IV	1	0.93

Most significantly, the prevalence of the genes encoding the Panton-Valentine leukocidin (*lukF/S-PV*) was high (54.21%).

### Clonal complexes and strains

Isolates were assigned to CCs and strains based on hybridisation profiles as defined previously [20,21]. Five major MRSA clones from four clonal complexes (CC) predominated. These highly prevalent strains included CC8/ST239-III, (Vienna/Hungary/Brazil Epidemic Strain), PVL-positive CC22-IV and PVL-negative CC22-IV (UK-EMRSA-15/Barnim Epidemic Strain), PVL-positive ST30-IV (Southwest Pacific Clone) and PVL-positive CC80-IV (European CA-MRSA Clone). Sporadic MRSA strains included PVL-negative CC5-IV, CC5-IV/SCC*fus*, CC6-IV (West Australian, WA, MRSA-51/66) and PVL-positive CC88-IV, PVL-positive CC5-IV, PVL-negative CC80-IV, CC97-V as well as CC1-IV/SCC*fus* (WA MRSA-1/45), PVL-positive CC1/ST772-V (Bengal Bay Clone/WA MRSA-60), PVL-negative CC5-V, CC45-IV (WA MRSA-23) and a CC9/ST834-MRSA strain with an unidentified SCC*mec* element.

Some major international MRSA clones, such as CC5-II (New-York-Japan Clone, USA100, UK-EMRSA-3 or Rhine-Hesse Epidemic Strain), PVL-positive ST8-IV (USA300), CC30-II (UK-EMRSA-16 or USA200), or CC45-II (USA600) were not detected among the tested sample population.

### Strain descriptions

Short strain profiles with regard to the carriage of resistance- or virulence-associated genes and to other genes of relevance for the determination of CCs are shown in Table 2. Full hybridisation profiles are provided in the Additional file 1.

**Table 2:** Characterisation of MRSA strains detected within this study

**CC**	**Strain**	**Number and percentage of isolates**	**Resistance-associated genes**	**Virulence-associated genes**	**Other relevant markers**
**1**	**CC1-IV/SCC*fus*** (WA MRSA-1/45)	1 (0.93%)	*mecA* (SCC*mec* IV), *blaZ/I/R, ccrA/B-1,* Q6GD50 (*fusC*)	*lukD/E, sea, seh, sek, seq, sak/scn,*	*agr* III, capsule type 8, *cna, sasG*
	**CC1/ST772-V [PVL+]** (Bengal Bay Clone)	1 (0.93%)	*mecA* (SCC*mec* V), *blaZ/I/R, msr*(A), *mph*(C) *aacA-aphD, aphA3/sat*	*lukF/S-PV, sea, sec, sel, egc-*cluster*, ORF CM14, scn*	*agr* II, capsule type 5, *cna, sasG*
**5**	**CC5-IV** (Paediatric Clone)	3 (2.80%)	*mecA* (SCC*mec* IV), *blaZ/I/R, erm*(C) (in 2/3)	*lukD/E, seb* (in 1/3), *egc-*cluster*, edinA* (in 1/3)	*agr* II, capsule type 5, *sasG*
	**CC5-IV [PVL+]** (Paediatric Clone)	2 (1.87%)	*mecA* (SCC*mec* IV), *blaZ/I/R* (in 1/2), *erm*(C), *aphA3/sat* (in 1/2)	*lukF/S-PV, lukD/E, sea-N315, sed/j/r* (in 1/2), *egc-*cluster*, sak/scn,*	*agr* II, capsule type 5, *sasG*
	**CC5-IV/SCC*fus*** ("Maltese Clone", see [22])	3 (2.80%)	*mecA* (SCC*mec* IV), *ccrA-3,* Q6GD50 (*fusC*), *blaZ/I/R* (in 2/3)	*lukD/E, tst1* (in 1/3), *sea, sec/l* (in 1/3), *egc-*cluster, *sak/scn*	*agr* II, capsule type 5, *sasG*
	**CC5-V**	1 (0.93%)	*mecA* (SCC*mec* V), *aacA-aphD*	*lukD/E, sea-N315, sed/j/r, egc-*cluster, *sak/scn*	*agr* II, capsule type 5, *sasG*
**6**	**CC6-IV** (WA MRSA-51/66)	3 (2.80%)	*mecA* (SCC*mec* IV), *blaZ/I/R*	*lukD/E, sea, sak/scn*	*agr* I, capsule type 8, *cna, sasG*
**8**	**CC8/ST239-III** (Vienna/Hungarian/Brazilian Clone)	22 (20.56%)	*mecA* (SCC*mec* III), *merA/B* (in14/22), *ccrC* (in 21/22), *blaZ/I/R, erm*(A) (in 21/22), *erm*(C) (in 1/22), *aacA-aphD* (in 13/22), *aphA3/sat* (in 13/22), *tet*(M). *tet*(K) (in 3/22), *cat* (in 1/22), *qacA* (in 20/22)	*lukD/E, sea* (in 1/22), *sek/q, sak/scn, chp* (in 1/22)	*agr* I, capsule type 8, *cna, sasG*
**9**	**CC9/ST834-(atyp. SCC*mec*)**	1 (0.93%)	*mecA, delta mecR, ugpQ, Q9XB68-dcs, ccrB-*4, Q6GD50 (*fusC*), *blaZ/I/R, msr(A)*	*lukD/E, tst1*, *sec/l, sak/chp/scn*	*agr* I, capsule type 8, *sasG*
**22**	**CC22-IV** (Barnim/UK-EMRSA-15)	10, including 2 environmental samples (9.35%)	*mecA* (SCC*mec* IV), *blaZ/I/R, erm*(C) (in 1/10), *msr*(A) (in 1/10), *aacA-aphD* (in 1/10), *tet*(K) (in 1/10), *dfrA*	*tst1* (in 6/10), *egc-*cluster, *sak/chp/scn* (in 9/10)/	*agr* I, capsule type 5, *cna, sasG*
	**CC22-IV [PVL+]**	20, including 2 environmental samples (18.69%)	*mecA* (SCC*mec* IV), *blaZ/I/R, erm*(C) (in 17/20), *aacA-aphD, aadD* (in 8/20), *dfrA* (in 19/20)	*lukF/S-PV, egc-*cluster, *sak/chp/scn*	*agr* I, capsule type 5, *cna, sasG*
**30**	**CC30-IV [PVL+]** (USA1100, Southwest Pacific or WSPP Clone)	13, including 1 environmental sample (12.15%)	*mecA* (SCC*mec* IV), *blaZ/I/R* (in 12/13), *erm*(C) (in 1/13), *msr*(A)/*mph*(C) (in 6/13), *aphA3/sat* (in 6/13)	*lukF/S-PV, egc-*cluster, *sak/chp/scn*	*agr* III, capsule type 8, *cna*
**45**	**CC45/*agr*IV-IV** (WA MRSA-23)	1 (0.93%)	*mecA* (SCC*mec* IV), *blaZ/I/R*	*sej/r, egc-*cluster, *sak/chp/scn*	*agr* IV, capsule type 8, *cna, sasG*
**80**	**CC80-IV**	2 (1.87%)	*mecA* (SCC*mec* IV), *blaZ/I/R, erm*(C), *aphA3/sat* (in 1/2), *far1* (in 1/2)	*lukD/E, seb/k/q* (in 1/2), *edinB, etD, sak/scn, chp* (in 1/2)	*agr* III, capsule type 8, *sasG*
	**CC80-IV [PVL+]** (European caMRSA Clone)	19 (17.76%)	*mecA* (SCC*mec* IV), *blaZ/I/R* (in 16/19), *erm*(C) (in 4/19), *aphA3/sat* (in 16/19), *far1* (in 17/19), *tet*(K) (in 2/19)	*lukF/S-PV, lukD/E, edinB, etD, sak/chp/scn*	*agr* III, capsule type 8, *sasG*
**88**	**CC88-IV [PVL+]**	3 (2.80%)	*mecA* (SCC*mec* IV), *blaZ/I/R, tet*(K) (in 2/3)	*lukF/S-PV, lukD/E, sea-N315* (in 2/3), *sak/chp/scn* (in 2/3)	*agr* III, capsule type 8, *sasG*
**97**	**CC97-V**	2 (1.87%)	*mecA* (SCC*mec* V)*,* Q6GD50 (*fusC*), *blaZ/I/R, aacA-aphD* (in 1/2), *tet*(K) (in 1/2)	*lukD/E, sak/scn*	*agr* I, capsule type 5, *sasG*

#### Clonal complex 1

Two isolates were identified that belong to CC1. One PVL-negative isolate carried SCC*mec* IV as well as *ccrA-1, ccrB-1* and the fusidic acid resistance marker Q6GD50 (*fusC*, GenBank BX571857.1:SAS0043). Thus it can be regarded as identical to the West Australian (WA) strain WA MRSA-45 and some of the isolates originally described as WA MRSA-1 [20,23].

The other isolate was identified as PVL-positive ST772-V, also known as Bengal Bay Clone or WA MRSA-60. This is a distinct clone which differs from other CC1 strains in several features such as in the *agr* allele (II rather than III), capsule type (5 rather than 8), carriage of the enterotoxin-like gene ORF CM14 (GenBank U10927.2) and the absence of the enterotoxin H gene *seh*.

#### Clonal complex 5

Both, PVL-negative as well as PVL-positive CC5-IV (Paediatric Clone or USA800) as well as one PVL-negative CC5-V isolate were found.

Three isolates belonged to a strain previously known only from Malta [22]. It is characterised by the presence of the fusidic acid resistance marker Q6GD50 (*fusC*) and additional recombinase genes beside a SCC*mec* IV element [22]. One out of these isolates harboured, beside *egc*, also *tst1, sec* and *sel* (encoding toxic shock syndrome toxin, enterotoxins C and L).

#### Clonal complex 6

Three isolates belonged to CC6-IV, which is identical to the Australian strains WA MRSA-51 and −66. They lacked PVL, but carried *sea.*

#### Clonal complex 8/sequence type 239

All 22 CC8 isolates belonged to ST239-III, which is a divergent strain that emerged from an incorporation of a large fragment of CC30 DNA [24,25]. All these isolates harboured the beta-lactamase-operon and *tet*(M) (tetracycline resistance) as well as, variably, further resistance genes as shown in Table 2. All isolates carried enterotoxin genes *sek* and *seq*.

Patients carrying this strain were older than average (mean, 43 years; median, 39 years).

#### Clonal complex 9/sequence type 834

ST834 is a distinct sequence type within CC9 that differs in several markers including the alleles of the regulatory locus *agr* (*agr* group I rather than II) and of some adhesion factors (*bbp, map, vwb*), in capsule type (8 rather than 5) and *spa* type and in the presence of *sasG* (encoding *S. aureus* surface protein G).

One isolate was identified as CC9/ST834 being essentially identical to the Australian strain ST834-IV, or WA MRSA-13, in all markers but the SCC*mec* element. It yielded signals for *mecA*, a truncated signal transducer protein *mecR1*, *ugpQ* (a glycerophosphoryl diester phosphodiesterase gene, associated with *mecA*), *ccrB-4* and Q6GD50 (*fusC*) as well as beta-lactamase and *msr*(A) (macrolide resistance). This strain carried *tst1, sec* and *sel,* but lacked PVL genes.

#### Clonal complex 22

CC22 was common; and PVL-positive as well as PVL-negative CC22-IV were identified.

Eighteen patient samples and two environmental samples were PVL-positive CC22-IV. All isolates harboured the beta-lactamase-operon and *aacA-aphD.* Other common resistance genes included *aadD* (tobramycin), *erm*(C) and *dfrA* (trimethoprim resistance). Virulence markers included *egc* and *lukF/S-PVL*, but no other toxin genes were identified.

Eight patient samples and two environmental samples were PVL-negative CC22-IV, i.e. identical or similar to the UK-EMRSA-15/Barnim Epidemic Strain. One of the environmental strains originated from the same sample as one of the PVL-positive CC22-IV. Since it was identical to it with regard to all markers but PVL genes, being positive for *erm*(C)*, dfrA* and *aacA-aphD*, it is likely that it was in fact a deletion variant of that strain. The *tst1* gene was detected in six isolates, but enterotoxin genes *sec* and *sel* were not found.

#### Clonal complex 30

Thirteen samples, including one environmental, belonged to PVL-positive CC30-IV (USA1100, Southwest Pacific or WSPP Clone). These isolates could be clustered into three variants based on the carriage of resistance genes. One variant (six isolates) harboured the beta-lactamase gene, but lacked other resistance markers. A second variant (one isolate) lacked *blaZ*, but carried *erm*(C). The third variant (six isolates) was positive for beta-lactamase, *msr*(A) and *mph*(C) as well as *aphA3* and *sat.* Virulence-associated genes included *lukF/S-PVL* and *egc*; other enterotoxin genes were not found.

#### Clonal complex 45

One single isolate of CC45-IV was found. It differed from the CC45-IV or Berlin Epidemic Strain, which is commonly detected in Western Europe, in harbouring *sasG* as well as different alleles of the *agr* locus (*agr* IV rather than I) and of adhesion factors *fnbA, fnbB, sdrD* and *vwb*. Thus it was related or identical to the WA MRSA-23 strain and to isolates previously identified in Hong Kong [20]. The isolate carried beta-lactamase as well as enterotoxin genes *sej* and *ser.*

#### Clonal complex 80

Two different CC80-IV strains were observed, one being PVL-positive and the other one PVL-negative. Two isolates were PVL-negative CC80-IV; one of them harboured enterotoxin genes *seb, sek* and *seq*.

Nineteen isolates from eighteen patients belonged to the PVL-positive CC80-IV strain (European CA-MRSA or WA MRSA-17/30). Six different variants of that strain could be differentiated based on various combinations of resistance genes *blaZ, erm*(C)*, aphA3 + sat*, *far1* and *tet*(K). One patient carried two isolates which differed in carriage of *blaZ* and *far1.* All PVL-positive CC80-IV isolates also harboured *edinB* and *etD*, but no enterotoxin genes were found.

#### Clonal complex 88

Three isolates belonged to a PVL-positive CC88-IV strain. Two out of three were positive for the distinct variant of the enterotoxin A gene, *sea-*N315 or *sep*, which is mainly known from the CC5 genome sequence of strain N315 (BA000018.3: SA1761).

#### Clonal complex 97

Two isolates were identified as CC97-V. Both harboured the beta-lactamase operon and Q6GD50, one was positive for *aacA-aphD* and *tet*(K). Both isolates lacked PVL as well as other exotoxin genes.

## Discussion

A striking result of the study was a high diversity of different MRSA strains and clonal complexes as well as a high prevalence of PVL. The most common strains identified during this study were ST239-III, PVL-positive and -negative CC22-IV, PVL-positive CC30-IV and PVL-positive CC80-IV.

ST239-III is a pandemic clone which is mainly hospital-associated. This might be the reason why carriers of that strain were older than the average. ST239-III was previously identified in various Middle Eastern countries including Abu Dhabi [2], Iran [3], Iraq [1], Saudi Arabia [4] and Turkey [5]. PVL-positive CC22-IV has been previously found in Great Britain and Ireland, Germany and Abu Dhabi [2]. Middle Eastern isolates, i.e., those from Abu Dhabi [2] and from the present study, generally differed from European ones in carrying additional resistance markers (*aacA-aphD, aadD, dfrA*). PVL-negative CC22-IV represents a pandemic strain known as UK-EMRSA-15, or Barnim Epidemic Strain. This strain is increasingly common in Western Europe and has also been found in Malta [22], Kuwait [7] and Abu Dhabi [2]. However, with an incidence of only 8.9% among our isolates it was distinctly less common than in Western Europe, where 50-95% of MRSA isolates might belong to that strain [20,22,26-29]. Its prevalence was also markedly low compared to a study from Abu Dhabi [2], where this strain accounted for 27.4% of MRSA isolates. This observation might be attributed to different population structures, to different patient collectives served by the respective hospitals and to a significant presence of European expatriates in the United Arab Emirates. Isolates of that strain from both, Riyadh and Abu Dhabi, often harboured *tst1*, which is normally absent from European isolates. Interestingly, the *tst1* gene in that strain was not accompanied by *sec* and *sel* genes. This might indicate another genetic background than the previously characterised *tst1*-carrying pathogenicity island SaPI1 [30]. PVL-positive CC22-IV have been described from Bavaria, Germany [31] as well as from Australia [20,32,33], England [34,35] and Ireland [33]. Observations of that strain from Abu Dhabi [2] and in German patients with family ties to Turkey [14] as well as the present study might suggest that this strain is common and widespread in the Middle East. PVL-positive CC30-IV is a strain mainly known from the Pacific islands, Samoa and New Zealand, but also from Abu Dhabi [2] and Kuwait [8]. An importation of that strain into Gulf countries appears to be likely due to the high numbers of immigrant labourers from Pacific countries such as the Philippines, as similarly noted in Denmark [36]. PVL-positive CC80-IV has been dubbed the European CA-MRSA strain as it is widespread although sporadically detected across several European countries. However, it appears to be more predominant in the Middle East and Maghreb (North African) countries being detected not only in Saudi Arabia but also in Abu Dhabi [2], Kuwait [37], Lebanon [9], Tunisia [11] and Algeria [12].

Other strains were rare being identified only in sporadic cases, accounting for less than 3% each. Some of the minor strains have been previously observed in other regions so that an importation might be likely. For others no, or only few, data on distribution or prevalence are available. Therefore it is not clear if they emerged locally or if they have been imported. For instance, CC1/ST772-V is known to mainly occur in India and Bangladesh, and cases in Europe are usually linked to these countries [35,38]. There might also be an epidemiological link to India for the isolate from this study, as there are high numbers of Indian workers, including healthcare workers, in Riyadh. CC5-IV is known to occur essentially worldwide. CC5-IV/SCC*fus* has been described only from Malta [22], so it would be interesting to check whether this strain has a wider distribution in the Mediterranean countries and the Middle East. CC6-IV has previously been observed not only in Australia, but also in Abu Dhabi [2]. Interestingly, CC6-MSSA has been found to be a common clone in Middle Eastern camels [39] so that a local emergence of CC6-IV after inter-species transfer and acquisition of a SCC*mec* element appears to be possible. PVL-negative CC80-IV appear to be extremely scarce, and the few detected isolates might be deletion variants of the so-called European CA-MRSA clone. One of the two isolates identified in this study carried enterotoxin genes, which is also a rare feature among CC80. PVL-positive CC88-IV are known from Abu Dhabi and, sporadically, from Europe. CC97-V has been previously identified in Egypt, which warrants further study on its presence in the Middle East. Since CC97 MSSA are common among domestic animals, here again a possible transmission from livestock should be investigated.

The MRSA strains found in Saudi Arabian patients showed a significantly high carriage of PVL genes (54.21%). Comparable high figures have been reported from Algeria [13] as well as from Abu Dhabi (41.9%, [2]). These are much higher proportions than observed in Western European countries [20,32,33]. Reasons for this difference are largely unknown. A possible explanation was a generally higher carriage of PVL in *S. aureus* from the Middle East, possibly related to climatic or host factors. If that was the case, the frequency of PVL-positive-methicillin susceptible *S. aureus* (MSSA) should also be high. However, data on MSSA from this region are currently not yet available. In order to understand the local epidemiology of PVL, further studies need to focus on MSSA as well as on MRSA in Middle Eastern countries.

It also might be speculated that PVL-MRSA just replaced PVL-MSSA in the Middle East, possibly favoured by a liberal use of antimicrobial drugs during the last decades. Interestingly, previously published MRSA genotyping data from Saudi Arabia showed a much lower PVL prevalence of only 8% (three out of 37) in SCCm*ec* IV strains isolated from skin tissue infections from patients seen in outpatient clinics in Riyadh in 2007 [40]. This finding may possibly relate to the small number of isolates processed or to a different patient collective. It might also indicate a massive expansion of PVL-positive MRSA clones during very recent years. This is also in accordance to an otherwise observed increase in CA-MRSA infections [19]. These observations emphasise the need for a more systematic surveillance of this potential public-health hazard.

Another interesting finding was that resistance markers that are traditionally associated with HA-MRSA (e.g.*, aacA-aphD, aadD*) were common among CA-MRSA strains. For instance, all PVL-positive CC22-IV in this study carried *aacA-aphD.* Thus, the detection of, e.g., gentamicin resistance in a clinical isolate must not be used to rule out a community origin or a possible presence of PVL in that actual isolate; and the decision to perform a molecular assay for PVL should be guided by the clinical symptoms of the patient rather than by the susceptibility profile of the isolate.

## Conclusion

A number of very diverse MRSA strains were found in Riyadh, Saudi Arabia in addition to a long established healthcare-associated MRSA strain (ST239-III). The prevalence of Panton-Valentine leukocidin genes was surprisingly high (54.21%), with PVL-positive clones also being present in a healthcare setting. A significant rate of resistance markers was detected in strains usually considered as community-associated. This is a rather different situation than in European countries. Screening and eradication programs thus need to focus not only on patients, but also on contact persons such as family members and healthcare personnel, too. Further studies are still needed to understand the epidemiology of MRSA in Saudi Arabia, possible changes in population structures during the last decades and possible sources for importation of epidemic strains from other regions.

## Methods

### Specimen collection and bacterial strains

The KFMC is a 1,400-bed tertiary care centre managing mainly Saudi Arabian paediatric and adult patients. In compliance with KFMC IC & EH policy, each patient is screened for MRSA prior to hospital admission by PCR using the BD GenOhm MRSA assay according to manufacturer's instructions (Becton Dickinson, USA). Patients were isolated in wards according to MRSA PCR results and all PCR-positive samples were cultured.

Isolates for the study were collected between summer 2010 and spring 2011. Sample types for the respective isolates are listed in the Additional file 1.

Five isolates related to environmental swabbing of areas near patients which were considered as potential sources of infection. Seven isolates (six from nasal swabs and one from sputum, see the Additional file 1) originated from screening samples. Another six isolates came from nasal and oral swabs taken during diagnostic procedures. The remaining isolates included 50 from swabs from skin lesions, abscesses etc., 15 from blood cultures, nine from respiratory samples, two from urines, two from drains and one from cerebrospinal fluid. For ten isolates, data could not be retrieved.

Isolates were subjected to antimicrobial microbial susceptibility testing (Becton Dickinson Phoenix, USA, according to Clinical & Laboratory Standards Institute guidelines) and submitted for array-based MRSA typing to the Faculty of Medicine, Dresden, Germany. Approval from the KFMC Institutional Review Board was obtained to use patient isolates for this study. Individual patient´s consent was not sought as isolates were derived from routine diagnostics and as data were processed anonymously. Copy strains, i.e.*,* multiple isolates from one individual patient were excluded from further analysis unless they differed in array hybridisation profiles. This was the case for four individual patients.

### Array procedures

For characterisation of isolates, the StaphyType DNA microarray (Alere Technologies GmbH, Jena, Germany) was used. This DNA microarray covers *ca*. 170 genes and their allelic variants. This includes species markers, typing markers (SCC*mec*, capsule and *agr* group), resistance genes as well as genes encoding exotoxins and adhesion factors. A list of the included target genes as well as primer and probe sequences have been published previously [20,21].

Procedures were performed according to protocols as recommended by the manufacturer and as previously published [20,21]. In short, MRSA were cultured on Columbia blood agar, harvested and enzymatically lysed prior to DNA preparation using an automated system (EZ1, Qiagen, Hilden, Germany). The purified DNA was used as template in a linear primer elongation reaction during which biotin-16-dUTP was incorporated into the resulting amplicons. Reaction products were hybridised to the microarray. After washing and blocking, horseradish-peroxidase-streptavidin conjugate was added which bound to the biotin labels. After further incubation and washing, a dye was added which locally precipitated in presence of the peroxidase. This resulted in the formation of visible spots on the array at these positions where hybridisations occurred. An image of the microarray was taken and analysed using a designated reader and software (Alere Technologies GmbH, Jena, Germany). Analysis allowed to determine the presence or absence of the target genes as well as, by comparison to a database of reference strains, the assignment to clonal complexes as previously defined by MLST [41] and eBURST analysis of MLST data (http://saureus.mlst.net/eburst/). Sequence types which differ in nucleotide polymorphisms affecting MLST genes (such as ST22 and ST1117) cannot be differentiated. However, STs which originate from recombination events such as CC8/ST239 or CC30/ST34 [24,25] can be identified as well as some other STs which differ from their respective parent lineage such as CC1/ST772 or CC8/ST72. Epidemic strains are defined and identified based on profiles and MLST data previously described [20,21].

## Competing interests

Stefan Monecke, Peter Slickers and Ralf Ehricht are employees of Alere Technologies GmbH. There was no external funding for this study.

## Authors' contributions

PS performed bioinformatic work and array design. SG and AH provided isolates and clinical data. AR and RH carried out the laboratory procedures, AR, RH, RE, LS and SM analysed the data. LS and SM wrote the paper and RE critically revised the manuscript. All authors read and approved the final manuscript.

## Supplementary Material

Additional file 1 Patient demographics and full hybridisation profiles. Click here for file
