# Supplementary material for: Characterisation of MRSA strains isolated from patients in a hospital in Riyadh, Kingdom of Saudi Arabia
Source: BMC Microbiol. 2012 Jul 23;12:146. doi: 10.1186/1471-2180-12-146 (PMC3464608; doi:10.1186/1471-2180-12-146)
Supplement: Additional file 1 — Patient demographics and full hybridisation profiles. [file 1471-2180-12-146-S1.pdf]

	DIAGNOSIS / SAMPLE TYPE	PATIENT AGE	PATIENT GENDER	SUSCEPTIBILITY TESTS							
				Erythromycin	Clindamycin	Trimethoprim - Sulfamethoxazole	Vancomycin	Linezolid	Teicoplanin	Rifampicin	Tetracycline
**CC1-MRSA-IV&SCCfus, WA MRSA-1/45**											
Riyadh-3108609	Nasopharynx carcinoma / Nasal swab	66	female	S	S	S	S	S	S	S	S
**CC1/ST772-MRSA-V [PVL+], "Bengal Bay Clone/WA MRSA-60"**											
Riyadh-2819026	Anal abscess / Skin swab	35	female	S	S	R	S	S	S	S	S
**CC5-MRSA-IV, Paediatric clone**											
Riyadh-2915327-1	Tendonitis / Blood	5	female	R	R	S	S	S	S	S	S
Riyadh-2915327-2	Tendonitis / Blood	5	female	R	R	S	S	S	S	S	S
Riyadh-2	NA (Not available)										
**CC5-MRSA-IV [PVL+], Paediatric clone**											
Riyadh-2986666	Ano-rectal Abscess / Skin swab	2	male	R	R	S	S	S	S	S	S
Riyadh-2913335	Thigh cellulitis / Skin swab	2	male	R	R	S	S	S	S	S	
**CC5-MRSA-IVvar, "Maltese Clone"**											
Riyadh-2983654	Wound / Skin swab	18	male	S	S	S	S	S	S	S	S
Riyadh-4	NA (Not available)										
Riyadh-2790233	Sarcoma / Skin swab	7	male	S	S	S	S	S	S	S	S
**CC5-MRSA-V**											
Riyadh-2568944	Screening / Nasal swab	26	female	S	S	S	S	S	S	S	S
**CC6-MRSA-IV, WA MRSA-51/66**											
Riyadh-2556168	Screening / Nasal swab	67	male	S	S		S	S	S	S	
Riyadh-2824507	Screening / Nasal swab	77	female								
Riyadh-2990831	Screening / Nasal swab	67	male	S	S	S	S	S	S	S	S
**CC8/ST239-MRSA-III, Vienna/Hungarian/Brazilian Clone**											
Riyadh-9	NA (Not available)										
Riyadh-3028763	Pneumonia / Tracheal swab	71	female	R	R	R	S	S	S		
Riyadh-2817437	Leg wound / Skin swab	54	male	R	R	R	S	S	S	S	
Riyadh-2793706	Carcinoma / Endotracheal aspirate	65	male	R	R	R	S	S			
Riyadh-2818797	Furuncle leg / Skin swab	29	female	R	R	R	S	S	S	S	S
Riyadh-2822825	Pressure ulcers / Skin swab	75	male	R	R	R	S	S	S	S	R
Riyadh-2888905	Fever / Endotracheal aspirate	25	male	R	R	R	S	S	S	S	
Riyadh-2888915	Crohn's disease / Blood	24	male	R	R	R	S	S	S	S	S
Riyadh-2567782	Pneumonia / Sputum	26	female	R	R	R	S	S	S	S	S
Riyadh-2891670	Orthopedic surgery / Skin swab	25	male	R	R	R	S	S	S	S	R
Riyadh-3006920	Heel wound / Skin swab	30	male	R	R	R	S	S	S	S	S
Riyadh-2817276-1	Renal tubular acidosis / Chest drain	47	male	R	R	R	S	S	S	S	R
Riyadh-0295102	Hypertension / Nasal swab	62	male								
Riyadh-2820597	Wound / Skin swab	21	female	R	R	R	S	S	S	S	S
Riyadh-2822088	Septic arthritis / Sputum	58	male	R	R	R	S	S	S	S	R
Riyadh-3010092	Surgery / Blood	63	male	R	R	R	S	S	S	S	R
Riyadh-3022844	Abscess / Skin swab	76	male	R	R	R	S	S	S	S	S
Riyadh-3108214-2	Pressure Ulcers / Skin swab	71	female								
Riyadh-2823926	Surgery wound / Skin swab	21	female	R	R	R	S	S	S	R	R
Riyadh-1	NA (Not available)										
Riyadh-2818388	Thalamic Tumor / Sputum	19	male	R	R	R	S	S	S	S	R
Riyadh-2818316	Chronic liver disease / Endotracheal aspirate	5	male	R	induc.R	R	S	S	S	S	R
**CC9/ST834-MRSA-(atypical SCCmec)**											
Riyadh-3103521	Sepsis / urine	0	male	S	S	R	S	S	S	S	
**CC22-MRSA-IV, Barnim/UK-EMRSA-15**											
Riyadh-2553359	Skin abscess / / Skin swab	2	male				S	S	S	S	
Riyadh-2571758	Screening / Nasal swab	27	female								
Riyadh-3029203	Umbilicus / Skin swab	3	female	S	S	R	S	S	S		
Riyadh-3039785	Wound / Skin swab	1	male	R	R	R	S	S	S	R	R
Riyadh-3105594	Ear infection / Skin swab	3	female	S	S	R	S	S	S	S	S
Riyadh_IC_204-2	Environmental sample										
Riyadh-3003974	Plasmacytoma / EndoTracheal aspirate	35	male	R	S	R	S	S			
Riyadh_IC_067	Environmental sample										
Riyadh-2988627	Wound / Skin swab	25	male	S	S	R	S	S	S	S	R
Riyadh-3112581	Right knee / Skin swab	25	male	R	R	R	S	S	S		
**CC22-MRSA-IV [PVL+]**											
Riyadh-2781996-1	Meningitis / Oral swab	1	male								
Riyadh-3103432	Wound / Skin swab	48	male								
Riyadh-3026502	Infection / Urine	1	male	S	S	R	S	S	S	S	S
Riyadh-3081378-2	Hydrocephalus / Blood	1	male								
Riyadh_IC_185	Environmental sample										
Riyadh_IC_204-1	Environmental sample										
Riyadh-2559371	Skin abscess / Skin swab	0	female				S	S	S	S	
Riyadh-2753975	Pre-term delivery / Blood	0	male	R	induc.R	S	S	S	S	S	S
Riyadh-2775605	Pre-term delivery / Blood	1	female	R	R	S	S	S	S	S	
Riyadh-2781996-2	Meningitis / Nasal Swab	1	male	R	R	R	S	S	S	S	S
Riyadh-2823783-2	Preterm delivery / Endotracheal aspirate	1	female								
Riyadh-2876601	Heart Failure / Blood	73	male	R	R	R	S	S	S	S	S
Riyadh-3036074	Pulmonary Fibrosis / Nasal swab	0	male	R	R	S	S	S	S	S	S
Riyadh-3053099	PT (preterm infant) / Blood	1	male	R	R		S	S		S	
Riyadh-3055366	Fever / CSF	33	female								
Riyadh-3082712	Pre-term delivery / Blood	0	female	R	R	R	S	R	S	S	
Riyadh-3087502	Musculoskeletal disorder / Skin swab	71	male								
Riyadh-6	NA (Not available)										
Riyadh-7	NA (Not available)										
Riyadh-8	NA (Not available)										
**CC30-MRSA-IV [PVL+], Southwest Pacific Clone**											
Riyadh-10	NA (Not available)										
Riyadh_IC_123	Environmental sample										
Riyadh-3080713	Ear infection / Skin swab	0	male	S	S	S	S	S	S	S	S
Riyadh-2803856	Foot abscess / Skin swab	6	male	S	S	S	S	S	S	S	S
Riyadh-2817276-1	Renal tubular acidosis / Chest drain	47	male								
Riyadh-2817571-2	Abscess / Skin swab	1	male	S	S	S	S	S	S	S	S
Riyadh-3033868	Obstetric surgery / Skin swab	23	female	S	S	S	S	S	S	S	S
Riyadh-2550108	Blister / Skin swab	1	female				S	S	S	S	
Riyadh-3095056-2	Abscess / Skin swab	2	male			R	S	S		S	
Riyadh-2819899	Artery infarct / Orbital cellulitis / Blood	78	male	R	S	R	S	S	S	S	S
Riyadh-2821805	Artery infarct / Skin swab	78	male	R	S	R	S	S	S	S	S
Riyadh-3013928	Right arm abscess / Skin swab	4	male	R	S	R	S	S	S		
Riyadh-3029402	Lower back abscess / Skin swab	4	male	R	S	R	S	S	S		
**CC45/agr IV-MRSA-IV, WA MRSA-23**											
Riyadh-3081378-1	Hydrocephalus / Skin swab	1	male								
**CC80-MRSA-IV**											
Riyadh-3107635	Ulcers / Skin swab	23	male	R	R	S	S	S		S	
Riyadh-2987458	Screening / Sputum	46	male	R	R	S	S	S	S	S	
**CC80-MRSA-IV [PVL+], European caMRSA Clone**											
Riyadh-2988048	Shoulder W / Skin swab	0	female	S	S	S	S	S	S	S	S
Riyadh-2990585-2	Fever / Nasal swab	0	male	S	S	S	S	S	S	S	S
Riyadh-2990585-1	Fever / Nasal swab	0	male	S	S	S	S	S	S	S	S
Riyadh-2826033	Wound / Skin swab	39	female	S	S	S	S	S	S	S	S
Riyadh-1601562	Skin abscess / Skin swab	40	female				S	S	S		S
Riyadh-2569940	Skin abscess / Skin swab	40	female	S	S	S	S	S	S	S	S
Riyadh-2571692	Skin abscess / Skin swab	40	female	S	S	S	S	S	S	S	S
Riyadh-2763029	Fever / Blood	9	male	S	S	S	S	S	S	S	S
Riyadh-2767090	Fever / Blood	8	female	S	S	S	S	S	S	S	S
Riyadh-2775130	Septic arthritis / Skin	NA	NA								
Riyadh-2778256	Fever / Blood	9	male	S	S	S	S	S	S	S	S
Riyadh-2817505	Surgical wound / Skin abscess	29	female	S	S	S	S	S	S	S	S
Riyadh-3024912	Wound / Skin swab	3	female	S	S	S	S	S	S	S	S
Riyadh-2788690	Furuncle Leg / Skin swab	53	male	R	R		S	S	S	S	
Riyadh-2829034	Wound / Skin abscess	2	female	R	R	S	S	S	S	S	S
Riyadh-3	NA (Not available)										
Riyadh-5	NA (Not available)										
Riyadh-2553167	Abscess / Skin swab	6	female	S	S	S	S	S	S	S	R
Riyadh-3002592	Abscess / Skin swab	10	male	S	S	S	S	S	S	S	R
**CC88-MRSA-IV [PVL+]**											
Riyadh-2736996	Deep Vein Thrombosis / Blood	41	male	S	S	S	S	S	S	S	R
Riyadh-2942396	Eye infection / Skin swab	12	male	S	S	S	S	S	S	S	S
Riyadh-3105391	Abscess / Skin swab	42	female				S	S	S	S	
**CC97-MRSA-V**											
Riyadh-0297622	Screening / Nasal swab	NA	NA								
Riyadh-3025471	Diabetic ulcer / Skin swab	74	male	S	S	S	S				

	SPECIES MARKER							REGULATORY GENES					
	Ribos. STAU	gapA	katA	CoA	nuc1	spa	sbi	sarA, saeS, vraS	agrI (total)	agrII (total)	agrIII (total)	agrIV (total)	hld
	Ribosomal sequence from S. aureus	glycer-aldehyde 3-phosphate dehydro-genase, locus 1	katalase A	coagulase	thermo-stable extracellular nuclease	Protein A	IgG-binding protein		accessory gene regulator allele I	accessory gene regulator allele II	accessory gene regulator allele III	accessory gene regulator allele IV	haemo- lysin delta
**CC1-MRSA-IV&SCCfus, WA MRSA-1/45**													
Riyadh-3108609	POS	POS	POS	POS	POS	POS	POS	POS	NEG	NEG	POS	NEG	POS
**CC1/ST772-MRSA-V [PVL+], "Bengal Bay Clone/WA I**													
Riyadh-2819026	POS	POS	POS	POS	POS	POS	POS	POS	NEG	POS	NEG	NEG	POS
**CC5-MRSA-IV, Paediatric clone**													
Riyadh-2915327-1	POS	POS	POS	POS	POS	POS	POS	POS	NEG	POS	NEG	NEG	POS
Riyadh-2915327-2	POS	POS	POS	POS	POS	POS	POS	POS	NEG	POS	NEG	NEG	POS
Riyadh-2	POS	POS	POS	POS	POS	POS	POS	POS	NEG	POS	NEG	NEG	POS
**CC5-MRSA-IV [PVL+], Paediatric clone**													
Riyadh-2986666	POS	POS	POS	POS	POS	POS	POS	POS	NEG	POS	NEG	NEG	POS
Riyadh-2913335	POS	POS	POS	POS	POS	POS	POS	POS	NEG	POS	NEG	NEG	POS
**CC5-MRSA-IVvar, "Maltese Clone"**													
Riyadh-2983654	POS	POS	POS	POS	POS	POS	POS	POS	NEG	POS	NEG	NEG	POS
Riyadh-4	POS	POS	POS	POS	POS	POS	POS	POS	NEG	POS	NEG	NEG	POS
Riyadh-2790233	POS	POS	POS	POS	POS	POS	POS	POS	NEG	POS	NEG	NEG	POS
**CC5-MRSA-V**													
Riyadh-2568944	POS	POS	POS	POS	POS	POS	POS	POS	NEG	POS	NEG	NEG	POS
**CC6-MRSA-IV, WA MRSA-51/66**													
Riyadh-2556168	POS	POS	POS	POS	POS	POS	POS	POS	POS	NEG	NEG	NEG	POS
Riyadh-2824507	POS	POS	POS	POS	POS	POS	POS	POS	POS	NEG	NEG	AMB	POS
Riyadh-2990831	POS	POS	POS	POS	POS	POS	POS	POS	POS	NEG	NEG	AMB	POS
**CC8/ST239-MRSA-III, Vienna/Hungarian/Brazilian Cl**													
Riyadh-9	POS	POS	POS	POS	POS	POS	POS	POS	POS	NEG	NEG	AMB	POS
Riyadh-3028763	POS	POS	POS	POS	POS	POS	POS	POS	POS	NEG	NEG	AMB	POS
Riyadh-2817437	POS	POS	POS	POS	POS	POS	POS	POS	POS	NEG	NEG	AMB	POS
Riyadh-2793706	POS	POS	POS	POS	POS	POS	POS	POS	POS	NEG	NEG	AMB	POS
Riyadh-2818797	POS	POS	POS	POS	POS	POS	POS	POS	POS	NEG	NEG	AMB	POS
Riyadh-2822825	POS	POS	POS	POS	POS	POS	POS	POS	POS	NEG	NEG	AMB	POS
Riyadh-2888905	POS	POS	POS	POS	POS	POS	POS	POS	POS	NEG	NEG	AMB	POS
Riyadh-2888915	POS	POS	POS	POS	POS	POS	POS	POS	POS	NEG	NEG	AMB	POS
Riyadh-2567782	POS	POS	POS	POS	POS	POS	POS	POS	POS	NEG	NEG	AMB	POS
Riyadh-2891670	POS	POS	POS	POS	POS	POS	POS	POS	POS	NEG	NEG	AMB	POS
Riyadh-3006920	POS	POS	POS	POS	POS	POS	POS	POS	POS	NEG	NEG	AMB	POS
Riyadh-2817276-1	POS	POS	POS	POS	POS	POS	POS	POS	POS	NEG	NEG	AMB	POS
Riyadh-0295102	AMB	POS	POS	POS	POS	POS	POS	POS	POS	NEG	NEG	AMB	POS
Riyadh-2820597	POS	POS	POS	POS	POS	POS	POS	POS	POS	NEG	NEG	AMB	POS
Riyadh-2822088	POS	POS	POS	POS	POS	POS	POS	POS	POS	NEG	NEG	AMB	POS
Riyadh-3010092	POS	POS	POS	POS	POS	POS	POS	POS	POS	NEG	NEG	AMB	POS
Riyadh-3022844	POS	POS	POS	POS	POS	POS	POS	POS	POS	NEG	NEG	AMB	POS
Riyadh-3108214-2	POS	POS	POS	POS	POS	POS	POS	POS	POS	NEG	NEG	NEG	POS
Riyadh-2823926	POS	POS	POS	POS	POS	POS	POS	POS	POS	NEG	NEG	POS	POS
Riyadh-1	POS	POS	POS	POS	POS	POS	POS	POS	POS	NEG	NEG	AMB	POS
Riyadh-2818388	POS	POS	POS	POS	POS	POS	POS	POS	POS	NEG	NEG	AMB	POS
Riyadh-2818316	POS	POS	POS	POS	POS	POS	POS	POS	POS	NEG	NEG	AMB	POS
**CC9/ST834-MRSA-(atypical SCCmec)**													
Riyadh-3103521	POS	POS	POS	POS	POS	POS	POS	POS	POS	NEG	NEG	AMB	POS
**CC22-MRSA-IV, Barnim/UK-EMRSA-15**													
Riyadh-2553359	POS	POS	POS	POS	POS	POS	POS	POS	POS	NEG	NEG	AMB	POS
Riyadh-2571758	POS	POS	POS	POS	POS	POS	POS	POS	POS	NEG	NEG	AMB	POS
Riyadh-3029203	POS	POS	POS	POS	POS	POS	POS	POS	POS	NEG	NEG	NEG	POS
Riyadh-3039785	POS	POS	POS	POS	POS	POS	POS	POS	POS	NEG	NEG	AMB	POS
Riyadh-3105594	POS	POS	POS	POS	POS	POS	POS	POS	POS	NEG	NEG	AMB	POS
Riyadh_IC_204-2	POS	POS	POS	POS	POS	POS	POS	POS	POS	NEG	NEG	AMB	POS
Riyadh-3003974	POS	POS	POS	POS	POS	POS	POS	POS	POS	NEG	NEG	POS	POS
Riyadh_IC_067	POS	POS	POS	POS	POS	POS	POS	POS	POS	NEG	NEG	AMB	POS
Riyadh-2988627	POS	POS	POS	POS	POS	POS	POS	POS	POS	NEG	NEG	AMB	POS
Riyadh-3112581	POS	POS	POS	POS	POS	POS	POS	POS	POS	NEG	NEG	AMB	POS
**CC22-MRSA-IV [PVL+]**													
Riyadh-2781996-1	POS	POS	POS	POS	POS	POS	POS	POS	POS	NEG	NEG	AMB	POS
Riyadh-3103432	POS	POS	POS	POS	POS	POS	POS	POS	POS	NEG	NEG	AMB	POS
Riyadh-3026502	AMB	POS	POS	POS	POS	POS	POS	POS	POS	NEG	NEG	NEG	POS
Riyadh-3081378-2	AMB	POS	POS	POS	POS	POS	POS	POS	POS	NEG	NEG	NEG	POS
Riyadh_IC_185	POS	POS	POS	POS	POS	POS	POS	POS	POS	NEG	NEG	AMB	POS
Riyadh_IC_204-1	POS	POS	POS	POS	POS	POS	POS	POS	POS	NEG	NEG	AMB	POS
Riyadh-2559371	POS	POS	POS	POS	POS	POS	POS	POS	POS	NEG	NEG	AMB	POS
Riyadh-2753975	POS	POS	POS	POS	POS	POS	POS	POS	POS	NEG	NEG	AMB	POS
Riyadh-2775605	POS	POS	POS	POS	POS	POS	POS	POS	POS	NEG	NEG	NEG	POS
Riyadh-2781996-2	POS	POS	POS	POS	POS	POS	POS	POS	POS	NEG	NEG	NEG	POS
Riyadh-2823783-2	POS	POS	POS	POS	POS	POS	POS	POS	POS	NEG	NEG	AMB	POS
Riyadh-2876601	POS	POS	POS	POS	POS	POS	POS	POS	POS	NEG	NEG	NEG	POS
Riyadh-3036074	POS	POS	POS	POS	POS	POS	POS	POS	POS	NEG	NEG	AMB	POS
Riyadh-3053099	POS	POS	POS	POS	POS	POS	POS	POS	POS	NEG	NEG	AMB	POS
Riyadh-3055366	POS	POS	POS	POS	POS	POS	POS	POS	POS	NEG	NEG	AMB	POS
Riyadh-3082712	POS	POS	POS	POS	POS	POS	POS	POS	POS	NEG	NEG	AMB	POS
Riyadh-3087502	POS	POS	POS	POS	POS	POS	POS	POS	pos	NEG	NEG	NEG	POS
Riyadh-6	POS	POS	POS	POS	POS	POS	POS	POS	POS	NEG	NEG	AMB	POS
Riyadh-7	POS	POS	POS	POS	POS	POS	POS	POS	POS	NEG	NEG	AMB	POS
Riyadh-8	POS	POS	POS	POS	POS	POS	POS	POS	POS	NEG	NEG	AMB	POS
**CC30-MRSA-IV [PVL+], Southwest Pacific Clone**													
Riyadh-10	POS	POS	POS	POS	POS	POS	POS	POS	NEG	NEG	POS	NEG	POS
Riyadh_IC_123	POS	POS	POS	POS	POS	POS	POS	POS	NEG	NEG	POS	NEG	POS
Riyadh-3080713	POS	POS	POS	POS	POS	POS	POS	POS	NEG	NEG	POS	NEG	POS
Riyadh-2803856	POS	POS	POS	POS	POS	POS	POS	POS	NEG	NEG	POS	NEG	POS
Riyadh-2817276-1	POS	POS	POS	POS	POS	POS	POS	POS	NEG	NEG	POS	NEG	POS
Riyadh-2817571-2	POS	POS	POS	POS	POS	POS	POS	POS	NEG	NEG	POS	NEG	POS
Riyadh-3033868	POS	POS	POS	POS	POS	POS	POS	POS	NEG	NEG	POS	NEG	POS
Riyadh-2550108	POS	POS	POS	POS	POS	POS	POS	POS	NEG	NEG	POS	NEG	POS
Riyadh-3095056-2	POS	POS	POS	POS	POS	POS	POS	POS	NEG	NEG	POS	NEG	POS
Riyadh-2819899	POS	POS	POS	POS	POS	POS	POS	POS	NEG	NEG	POS	NEG	POS
Riyadh-2821805	POS	POS	POS	POS	POS	POS	POS	POS	NEG	NEG	POS	NEG	POS
Riyadh-3013928	POS	POS	POS	POS	POS	POS	POS	POS	NEG	NEG	POS	NEG	POS
Riyadh-3029402	POS	POS	POS	POS	POS	POS	POS	POS	NEG	NEG	POS	NEG	POS
**CC45/agr IV-MRSA-IV, WA MRSA-23**													
Riyadh-3081378-1	AMB	POS	POS	POS	POS	POS	POS	POS	NEG	NEG	NEG	POS	POS
**CC80-MRSA-IV**													
Riyadh-3107635	POS	POS	POS	POS	POS	POS	POS	POS	NEG	NEG	POS	NEG	POS
Riyadh-2987458	POS	POS	POS	POS	POS	POS	POS	POS	NEG	NEG	POS	NEG	POS
**CC80-MRSA-IV [PVL+], European caMRSA Clone**													
Riyadh-2988048	POS	POS	POS	POS	POS	POS	POS	POS	NEG	NEG	POS	NEG	POS
Riyadh-2990585-2	POS	POS	POS	POS	POS	POS	POS	POS	NEG	NEG	POS	NEG	POS
Riyadh-2990585-1	POS	POS	POS	POS	POS	POS	POS	POS	NEG	NEG	POS	NEG	POS
Riyadh-2826033	POS	POS	POS	POS	POS	POS	POS	POS	NEG	NEG	POS	NEG	POS
Riyadh-1601562	POS	POS	POS	POS	POS	POS	POS	POS	NEG	NEG	POS	NEG	POS
Riyadh-2569940	POS	POS	POS	POS	POS	POS	POS	POS	NEG	NEG	POS	NEG	POS
Riyadh-2571692	POS	POS	POS	POS	POS	POS	POS	POS	NEG	NEG	POS	NEG	POS
Riyadh-2763029	POS	POS	POS	POS	POS	POS	POS	POS	NEG	NEG	POS	NEG	POS
Riyadh-2767090	POS	POS	POS	POS	POS	POS	POS	POS	NEG	NEG	POS	NEG	POS
Riyadh-2775130	POS	POS	POS	POS	POS	POS	POS	POS	NEG	NEG	POS	NEG	POS
Riyadh-2778256	POS	POS	POS	POS	POS	POS	POS	POS	NEG	NEG	POS	NEG	POS
Riyadh-2817505	POS	POS	POS	POS	POS	POS	POS	POS	NEG	NEG	POS	NEG	POS
Riyadh-3024912	POS	POS	POS	POS	POS	POS	POS	POS	NEG	NEG	POS	NEG	POS
Riyadh-2788690	POS	POS	POS	POS	POS	POS	POS	POS	NEG	NEG	POS	NEG	POS
Riyadh-2829034	POS	POS	POS	POS	POS	POS	POS	POS	NEG	NEG	POS	NEG	POS
Riyadh-3	POS	POS	POS	POS	POS	POS	POS	POS	NEG	NEG	POS	NEG	POS
Riyadh-5	POS	POS	POS	POS	POS	POS	POS	POS	NEG	NEG	POS	NEG	POS
Riyadh-2553167	POS	POS	POS	POS	POS	POS	POS	POS	NEG	NEG	POS	NEG	POS
Riyadh-3002592	POS	POS	POS	POS	POS	POS	POS	POS	NEG	NEG	POS	NEG	POS
**CC88-MRSA-IV [PVL+]**													
Riyadh-2736996	POS	POS	POS	POS	POS	POS	POS	POS	NEG	NEG	POS	NEG	POS
Riyadh-2942396	POS	POS	POS	POS	POS	POS	POS	POS	NEG	NEG	POS	NEG	POS
Riyadh-3105391	POS	POS	POS	POS	POS	POS	POS	POS	NEG	NEG	POS	NEG	POS
**CC97-MRSA-V**													
Riyadh-0297622	POS	POS	POS	POS	POS	POS	POS	POS	POS	NEG	NEG	AMB	POS
Riyadh-3025471	POS	POS	POS	POS	POS	POS	POS	POS	POS	NEG	NEG	NEG	POS

	METHICILLIN RESISTANCE AND SCCmec TYPING												
	mecA	delta_mecR	ugpQ	ccrA-1	ccrB-1	plsSCC (COL)	Q9XB68-dcs	ccrA-2	ccrB-2	kdpA/B/C/D/E-SCC	mecI	mecR	xylR
	alternate penicillin binding protein 2, defining MRSA	truncated signal transducer protein MecR1	glycerophosphoryl diester phosphodi-esterase, associated with mecA	cassette chromosome recombinase genes A/B-1		plasmin-sensitive surface protein	hypothetical protein from SCCmec elements	cassette chromosome recombinase genes A/B-2		potassium-transporter locus from SCCmec II	methicillin-resistance regulatory protein	signal transducer protein MecR1	homolog of xylose repressor, associated with SCCmec-elements
**CC1-MRSA-IV&SCCfus, WA MRSA-1/45**													
Riyadh-3108609	POS	POS	POS	POS	POS	NEG	NEG	POS	POS	NEG	NEG	NEG	NEG
**CC1/ST772-MRSA-V [PVL+], "Bengal Bay Clone/WA I**													
Riyadh-2819026	POS	NEG	POS	NEG	NEG	NEG	NEG	NEG	NEG	NEG	NEG	NEG	AMB
**CC5-MRSA-IV, Paediatric clone**													
Riyadh-2915327-1	POS	POS	POS	NEG	NEG	NEG	POS	POS	POS	NEG	NEG	NEG	NEG
Riyadh-2915327-2	POS	POS	POS	NEG	NEG	NEG	POS	POS	POS	NEG	NEG	NEG	NEG
Riyadh-2	POS	POS	POS	NEG	NEG	NEG	POS	POS	POS	NEG	NEG	NEG	NEG
**CC5-MRSA-IV [PVL+], Paediatric clone**													
Riyadh-2986666	POS	POS	POS	NEG	NEG	NEG	POS	POS	POS	NEG	NEG	NEG	NEG
Riyadh-2913335	POS	POS	POS	NEG	NEG	NEG	POS	POS	POS	NEG	NEG	NEG	NEG
**CC5-MRSA-IVvar, "Maltese Clone"**													
Riyadh-2983654	POS	POS	POS	AMB	AMB	NEG	POS	POS	POS	NEG	NEG	NEG	NEG
Riyadh-4	POS	POS	POS	NEG	NEG	NEG	POS	POS	POS	NEG	NEG	NEG	NEG
Riyadh-2790233	POS	POS	POS	NEG	AMB	NEG	POS	POS	POS	NEG	NEG	NEG	NEG
**CC5-MRSA-V**													
Riyadh-2568944	POS	NEG	POS	NEG	NEG	NEG	NEG	NEG	NEG	NEG	NEG	NEG	NEG
**CC6-MRSA-IV, WA MRSA-51/66**													
Riyadh-2556168	POS	POS	POS	NEG	NEG	NEG	POS	POS	POS	NEG	NEG	NEG	NEG
Riyadh-2824507	POS	POS	POS	NEG	NEG	NEG	POS	POS	POS	NEG	NEG	NEG	NEG
Riyadh-2990831	POS	POS	POS	NEG	NEG	NEG	POS	POS	POS	NEG	NEG	NEG	NEG
**CC8/ST239-MRSA-III, Vienna/Hungarian/Brazilian Cl**													
Riyadh-9	POS	POS	POS	NEG	POS	NEG	NEG	NEG	NEG	NEG	POS	POS	POS
Riyadh-3028763	POS	POS	POS	NEG	POS	NEG	NEG	NEG	NEG	NEG	POS	POS	POS
Riyadh-2817437	POS	POS	POS	NEG	POS	NEG	NEG	NEG	NEG	NEG	POS	POS	POS
Riyadh-2793706	POS	POS	POS	NEG	POS	NEG	NEG	NEG	NEG	NEG	POS	POS	POS
Riyadh-2818797	POS	POS	POS	NEG	POS	NEG	NEG	NEG	NEG	NEG	POS	POS	POS
Riyadh-2822825	POS	POS	POS	NEG	POS	NEG	NEG	NEG	NEG	NEG	POS	POS	POS
Riyadh-2888905	POS	POS	POS	NEG	POS	NEG	NEG	NEG	NEG	NEG	POS	POS	POS
Riyadh-2888915	POS	POS	POS	NEG	POS	NEG	NEG	NEG	NEG	NEG	POS	POS	POS
Riyadh-2567782	POS	POS	POS	NEG	AMB	NEG	NEG	NEG	NEG	NEG	POS	POS	POS
Riyadh-2891670	POS	POS	POS	NEG	POS	NEG	NEG	NEG	NEG	NEG	POS	POS	POS
Riyadh-3006920	POS	POS	POS	NEG	AMB	NEG	NEG	NEG	NEG	NEG	POS	POS	POS
Riyadh-2817276-1	POS	POS	POS	NEG	POS	NEG	NEG	NEG	NEG	NEG	POS	POS	POS
Riyadh-0295102	POS	POS	POS	NEG	AMB	NEG	NEG	NEG	NEG	NEG	POS	POS	POS
Riyadh-2820597	POS	POS	POS	NEG	POS	NEG	NEG	NEG	NEG	NEG	POS	POS	POS
Riyadh-2822088	POS	POS	POS	NEG	POS	NEG	NEG	NEG	NEG	NEG	POS	POS	POS
Riyadh-3010092	POS	POS	POS	NEG	AMB	NEG	NEG	NEG	NEG	NEG	POS	POS	POS
Riyadh-3022844	POS	POS	POS	NEG	AMB	NEG	NEG	NEG	NEG	NEG	POS	POS	POS
Riyadh-3108214-2	POS	POS	POS	NEG	NEG	NEG	NEG	NEG	NEG	NEG	POS	POS	POS
Riyadh-2823926	POS	POS	POS	NEG	POS	NEG	NEG	NEG	NEG	NEG	POS	POS	POS
Riyadh-1	POS	POS	POS	NEG	POS	NEG	NEG	NEG	NEG	NEG	POS	POS	POS
Riyadh-2818388	POS	POS	POS	NEG	POS	NEG	NEG	NEG	NEG	NEG	POS	POS	POS
Riyadh-2818316	POS	POS	POS	NEG	POS	NEG	NEG	NEG	NEG	NEG	POS	POS	POS
**CC9/ST834-MRSA-(atypical SCCmec)**													
Riyadh-3103521	POS	POS	POS	NEG	NEG	NEG	POS	NEG	NEG	NEG	NEG	NEG	NEG
**CC22-MRSA-IV, Barnim/UK-EMRSA-15**													
Riyadh-2553359	POS	POS	POS	NEG	NEG	NEG	NEG	POS	POS	NEG	NEG	NEG	NEG
Riyadh-2571758	POS	POS	POS	NEG	NEG	NEG	NEG	POS	POS	NEG	NEG	NEG	NEG
Riyadh-3029203	POS	POS	POS	NEG	NEG	NEG	NEG	POS	POS	NEG	NEG	NEG	NEG
Riyadh-3039785	POS	POS	POS	NEG	NEG	NEG	NEG	POS	POS	NEG	NEG	NEG	NEG
Riyadh-3105594	POS	POS	POS	NEG	NEG	NEG	NEG	POS	POS	NEG	NEG	NEG	NEG
Riyadh_IC_204-2	POS	POS	POS	NEG	NEG	NEG	POS	POS	POS	NEG	NEG	NEG	NEG
Riyadh-3003974	POS	POS	POS	NEG	NEG	NEG	NEG	POS	POS	NEG	NEG	NEG	NEG
Riyadh_IC_067	POS	POS	POS	NEG	NEG	NEG	NEG	POS	POS	NEG	NEG	NEG	NEG
Riyadh-2988627	POS	POS	POS	NEG	NEG	NEG	NEG	POS	POS	NEG	NEG	NEG	NEG
Riyadh-3112581	POS	POS	POS	NEG	NEG	NEG	NEG	POS	POS	NEG	NEG	NEG	NEG
**CC22-MRSA-IV [PVL+]**													
Riyadh-2781996-1	POS	POS	POS	NEG	NEG	NEG	POS	POS	POS	NEG	NEG	NEG	NEG
Riyadh-3103432	POS	POS	POS	NEG	NEG	NEG	POS	POS	POS	NEG	NEG	NEG	NEG
Riyadh-3026502	POS	POS	POS	NEG	NEG	NEG	AMB	POS	POS	NEG	NEG	NEG	NEG
Riyadh-3081378-2	POS	POS	POS	NEG	NEG	NEG	AMB	POS	POS	NEG	NEG	NEG	NEG
Riyadh_IC_185	POS	POS	POS	NEG	NEG	NEG	POS	POS	POS	NEG	NEG	NEG	NEG
Riyadh_IC_204-1	POS	POS	POS	NEG	NEG	NEG	POS	POS	POS	NEG	NEG	NEG	NEG
Riyadh-2559371	POS	POS	POS	NEG	NEG	NEG	POS	POS	POS	NEG	NEG	NEG	NEG
Riyadh-2753975	POS	POS	POS	NEG	NEG	NEG	POS	POS	POS	NEG	NEG	NEG	NEG
Riyadh-2775605	POS	POS	POS	NEG	NEG	NEG	POS	POS	POS	NEG	NEG	NEG	NEG
Riyadh-2781996-2	POS	POS	POS	NEG	NEG	NEG	POS	POS	POS	NEG	NEG	NEG	NEG
Riyadh-2823783-2	POS	POS	POS	NEG	NEG	NEG	POS	POS	POS	NEG	NEG	NEG	NEG
Riyadh-2876601	POS	POS	POS	NEG	NEG	NEG	POS	POS	POS	NEG	NEG	NEG	NEG
Riyadh-3036074	POS	POS	POS	NEG	NEG	NEG	POS	POS	POS	NEG	NEG	NEG	NEG
Riyadh-3053099	POS	POS	POS	NEG	NEG	NEG	POS	POS	POS	NEG	NEG	NEG	NEG
Riyadh-3055366	POS	POS	POS	NEG	NEG	NEG	POS	POS	POS	NEG	NEG	NEG	NEG
Riyadh-3082712	POS	POS	POS	NEG	NEG	NEG	POS	POS	POS	NEG	NEG	NEG	NEG
Riyadh-3087502	POS	POS	POS	NEG	NEG	NEG	NEG	POS	POS	NEG	NEG	NEG	NEG
Riyadh-6	POS	POS	POS	NEG	NEG	NEG	POS	POS	POS	NEG	NEG	NEG	NEG
Riyadh-7	POS	POS	POS	NEG	NEG	NEG	POS	POS	POS	NEG	NEG	NEG	NEG
Riyadh-8	POS	POS	POS	NEG	NEG	NEG	POS	POS	POS	NEG	NEG	NEG	NEG
**CC30-MRSA-IV [PVL+], Southwest Pacific Clone**													
Riyadh-10	POS	POS	POS	NEG	NEG	NEG	POS	POS	POS	NEG	NEG	NEG	NEG
Riyadh_IC_123	POS	POS	POS	NEG	NEG	NEG	POS	POS	POS	NEG	NEG	NEG	NEG
Riyadh-3080713	POS	POS	POS	NEG	NEG	NEG	POS	POS	POS	NEG	NEG	NEG	NEG
Riyadh-2803856	POS	POS	POS	NEG	NEG	NEG	POS	POS	POS	NEG	NEG	#ZAHL!	NEG
Riyadh-2817276-1	POS	POS	POS	NEG	NEG	NEG	POS	POS	POS	NEG	NEG	NEG	AMB
Riyadh-2817571-2	POS	POS	POS	NEG	NEG	NEG	POS	POS	POS	NEG	NEG	NEG	NEG
Riyadh-3033868	POS	POS	POS	NEG	NEG	NEG	POS	POS	POS	NEG	NEG	NEG	NEG
Riyadh-2550108	POS	POS	POS	NEG	NEG	NEG	POS	POS	POS	NEG	NEG	NEG	NEG
Riyadh-3095056-2	POS	POS	POS	NEG	NEG	NEG	POS	POS	POS	NEG	NEG	NEG	NEG
Riyadh-2819899	POS	POS	POS	NEG	NEG	NEG	POS	POS	POS	NEG	NEG	NEG	NEG
Riyadh-2821805	POS	POS	POS	NEG	NEG	NEG	POS	POS	POS	NEG	NEG	NEG	NEG
Riyadh-3013928	POS	POS	POS	NEG	NEG	NEG	POS	POS	POS	NEG	NEG	NEG	NEG
Riyadh-3029402	POS	POS	POS	NEG	NEG	NEG	POS	POS	POS	NEG	NEG	NEG	NEG
**CC45/agr IV-MRSA-IV, WA MRSA-23**													
Riyadh-3081378-1	POS	POS	POS	NEG	NEG	NEG	AMB	POS	POS	NEG	NEG	NEG	NEG
**CC80-MRSA-IV**													
Riyadh-3107635	POS	POS	POS	NEG	NEG	NEG	POS	POS	POS	NEG	NEG	NEG	NEG
Riyadh-2987458	POS	POS	POS	NEG	NEG	NEG	POS	POS	POS	NEG	NEG	NEG	NEG
**CC80-MRSA-IV [PVL+], European caMRSA Clone**													
Riyadh-2988048	POS	POS	POS	NEG	NEG	NEG	POS	POS	POS	NEG	NEG	NEG	NEG
Riyadh-2990585-2	POS	POS	POS	NEG	NEG	NEG	POS	POS	POS	NEG	NEG	NEG	NEG
Riyadh-2990585-1	POS	POS	POS	NEG	NEG	NEG	POS	POS	POS	NEG	NEG	NEG	NEG
Riyadh-2826033	POS	POS	POS	NEG	NEG	NEG	POS	POS	POS	NEG	NEG	NEG	NEG
Riyadh-1601562	POS	POS	POS	NEG	NEG	NEG	POS	POS	POS	NEG	NEG	NEG	NEG
Riyadh-2569940	POS	POS	POS	NEG	NEG	NEG	POS	POS	POS	NEG	NEG	NEG	NEG
Riyadh-2571692	POS	POS	POS	NEG	NEG	NEG	POS	POS	POS	NEG	NEG	NEG	NEG
Riyadh-2763029	POS	POS	POS	NEG	NEG	NEG	POS	POS	POS	NEG	NEG	POS	NEG
Riyadh-2767090	POS	POS	POS	NEG	NEG	NEG	POS	POS	POS	NEG	NEG	AMB	NEG
Riyadh-2775130	POS	POS	POS	NEG	NEG	NEG	POS	POS	POS	NEG	NEG	AMB	NEG
Riyadh-2778256	POS	POS	POS	NEG	NEG	NEG	POS	POS	POS	NEG	NEG	NEG	NEG
Riyadh-2817505	POS	POS	POS	NEG	NEG	NEG	POS	POS	POS	NEG	NEG	NEG	NEG
Riyadh-3024912	POS	POS	POS	NEG	NEG	NEG	POS	POS	POS	NEG	NEG	NEG	NEG
Riyadh-2788690	POS	POS	POS	NEG	NEG	NEG	POS	POS	POS	NEG	NEG	NEG	NEG
Riyadh-2829034	POS	POS	POS	NEG	NEG	NEG	POS	POS	POS	NEG	NEG	NEG	NEG
Riyadh-3	POS	POS	POS	NEG	NEG	NEG	POS	POS	POS	NEG	NEG	NEG	NEG
Riyadh-5	POS	POS	POS	NEG	NEG	NEG	POS	POS	POS	NEG	NEG	NEG	NEG
Riyadh-2553167	POS	POS	POS	NEG	NEG	NEG	POS	POS	POS	NEG	NEG	NEG	NEG
Riyadh-3002592	POS	POS	POS	NEG	NEG	NEG	POS	POS	POS	NEG	NEG	NEG	NEG
**CC88-MRSA-IV [PVL+]**													
Riyadh-2736996	POS	POS	POS	NEG	NEG	NEG	POS	POS	POS	NEG	NEG	POS	NEG
Riyadh-2942396	POS	POS	POS	NEG	NEG	NEG	POS	POS	POS	NEG	NEG	NEG	NEG
Riyadh-3105391	POS	POS	POS	NEG	NEG	NEG	POS	POS	POS	NEG	NEG	NEG	NEG
**CC97-MRSA-V**													
Riyadh-0297622	POS	NEG	POS	NEG	NEG	NEG	NEG	NEG	NEG	NEG	NEG	NEG	NEG
Riyadh-3025471	POS	NEG	POS	NEG	NEG	NEG	NEG	NEG	NEG	NEG	NEG	NEG	NEG

	METHICILLIN RESISTANCE AND SCCmec TYPING									RESISTANCE : PENICILLINASE		
	ccrA-3	ccrB-3	merA	merB	ccrAA (MRSAZH47)_probe 1	ccrAA (MRSAZH47)_probe 2	ccrC (85-2082)	ccrA-4	ccrB-4	blaZ	blaI	blaR
	cassette chromosome recombinase genes A/B-3		mercury resistance operon		cassette chromosome recombinase genes "ccrAA" (hypothetical) and ccrC			cassette chromosome recombinase genes A/B-4		beta-lactamase	beta lactamase repressor (inhibitor)	beta-lactamase regulatory protein
**CC1-MRSA-IV&SCCfus, WA MRSA-1/45**												
Riyadh-3108609	NEG	AMB	NEG	NEG	NEG	NEG	NEG	NEG	NEG	POS	POS	POS
**CC1/ST772-MRSA-V [PVL+], "Bengal Bay Clone/WA I**												
Riyadh-2819026	NEG	NEG	NEG	NEG	POS	POS	POS	NEG	NEG	POS	POS	POS
**CC5-MRSA-IV, Paediatric clone**												
Riyadh-2915327-1	NEG	NEG	NEG	NEG	NEG	NEG	NEG	NEG	NEG	POS	POS	POS
Riyadh-2915327-2	NEG	NEG	NEG	NEG	NEG	NEG	NEG	NEG	NEG	POS	POS	POS
Riyadh-2	NEG	NEG	NEG	NEG	NEG	NEG	NEG	NEG	NEG	POS	POS	POS
**CC5-MRSA-IV [PVL+], Paediatric clone**												
Riyadh-2986666	NEG	NEG	NEG	NEG	NEG	NEG	NEG	NEG	NEG	POS	POS	POS
Riyadh-2913335	NEG	NEG	NEG	NEG	NEG	NEG	NEG	NEG	NEG	NEG	NEG	NEG
**CC5-MRSA-IVvar, "Maltese Clone"**												
Riyadh-2983654	POS	NEG	NEG	NEG	NEG	NEG	NEG	NEG	NEG	POS	POS	POS
Riyadh-4	POS	NEG	NEG	NEG	NEG	NEG	NEG	NEG	NEG	POS	POS	POS
Riyadh-2790233	POS	NEG	NEG	NEG	NEG	NEG	NEG	NEG	NEG	NEG	NEG	NEG
**CC5-MRSA-V**												
Riyadh-2568944	NEG	NEG	NEG	NEG	POS	POS	POS	NEG	NEG	NEG	NEG	NEG
**CC6-MRSA-IV, WA MRSA-51/66**												
Riyadh-2556168	NEG	NEG	NEG	NEG	NEG	NEG	NEG	NEG	NEG	POS	POS	POS
Riyadh-2824507	NEG	NEG	NEG	NEG	NEG	NEG	NEG	NEG	NEG	POS	POS	POS
Riyadh-2990831	NEG	NEG	NEG	NEG	NEG	NEG	NEG	NEG	NEG	POS	POS	POS
**CC8/ST239-MRSA-III, Vienna/Hungarian/Brazilian Cl**												
Riyadh-9	POS	POS	NEG	NEG	NEG	POS	POS	NEG	NEG	POS	POS	POS
Riyadh-3028763	POS	POS	NEG	NEG	NEG	NEG	POS	NEG	NEG	POS	POS	POS
Riyadh-2817437	POS	POS	NEG	NEG	NEG	AMB	POS	NEG	NEG	POS	POS	POS
Riyadh-2793706	POS	POS	NEG	NEG	NEG	NEG	POS	NEG	NEG	POS	POS	POS
Riyadh-2818797	POS	POS	NEG	NEG	NEG	AMB	POS	NEG	NEG	POS	POS	POS
Riyadh-2822825	POS	POS	NEG	NEG	NEG	AMB	POS	NEG	NEG	POS	POS	POS
Riyadh-2888905	POS	POS	NEG	NEG	NEG	AMB	POS	NEG	NEG	POS	POS	POS
Riyadh-2888915	POS	POS	NEG	NEG	NEG	AMB	POS	NEG	NEG	POS	POS	POS
Riyadh-2567782	POS	POS	POS	POS	NEG	NEG	POS	NEG	NEG	POS	POS	POS
Riyadh-2891670	POS	POS	POS	POS	NEG	AMB	POS	NEG	NEG	POS	POS	POS
Riyadh-3006920	POS	POS	POS	POS	NEG	NEG	POS	NEG	NEG	POS	POS	POS
Riyadh-2817276-1	POS	POS	POS	POS	NEG	AMB	POS	NEG	NEG	POS	POS	POS
Riyadh-0295102	POS	POS	POS	POS	NEG	NEG	POS	NEG	NEG	POS	POS	POS
Riyadh-2820597	POS	POS	POS	POS	NEG	AMB	POS	NEG	NEG	POS	POS	POS
Riyadh-2822088	POS	POS	POS	POS	NEG	AMB	POS	NEG	NEG	POS	POS	POS
Riyadh-3010092	POS	POS	POS	POS	NEG	NEG	POS	NEG	NEG	POS	POS	POS
Riyadh-3022844	POS	POS	POS	POS	NEG	NEG	POS	NEG	NEG	POS	POS	POS
Riyadh-3108214-2	POS	POS	POS	POS	NEG	NEG	POS	NEG	NEG	POS	POS	POS
Riyadh-2823926	POS	POS	POS	POS	NEG	AMB	POS	NEG	NEG	POS	POS	POS
Riyadh-1	POS	POS	POS	POS	NEG	AMB	POS	NEG	NEG	POS	POS	POS
Riyadh-2818388	POS	POS	POS	POS	NEG	AMB	POS	NEG	NEG	POS	POS	POS
Riyadh-2818316	POS	POS	POS	POS	NEG	AMB	NEG	NEG	NEG	POS	POS	POS
**CC9/ST834-MRSA-(atypical SCCmec)**												
Riyadh-3103521	NEG	NEG	NEG	NEG	NEG	NEG	NEG	NEG	POS	POS	POS	POS
**CC22-MRSA-IV, Barnim/UK-EMRSA-15**												
Riyadh-2553359	NEG	NEG	NEG	NEG	NEG	NEG	NEG	NEG	NEG	POS	POS	POS
Riyadh-2571758	NEG	NEG	NEG	NEG	NEG	NEG	NEG	NEG	NEG	POS	POS	POS
Riyadh-3029203	NEG	NEG	NEG	NEG	NEG	NEG	NEG	NEG	NEG	POS	POS	POS
Riyadh-3039785	NEG	NEG	NEG	NEG	NEG	NEG	NEG	NEG	NEG	POS	POS	POS
Riyadh-3105594	NEG	NEG	NEG	NEG	NEG	NEG	NEG	NEG	NEG	POS	POS	POS
Riyadh_IC_204-2	NEG	NEG	NEG	NEG	NEG	NEG	NEG	NEG	NEG	POS	POS	POS
Riyadh-3003974	NEG	NEG	NEG	NEG	NEG	NEG	NEG	NEG	NEG	POS	POS	POS
Riyadh_IC_067	NEG	NEG	NEG	NEG	NEG	NEG	NEG	NEG	NEG	POS	POS	POS
Riyadh-2988627	NEG	NEG	NEG	NEG	NEG	NEG	NEG	NEG	NEG	POS	POS	POS
Riyadh-3112581	NEG	NEG	NEG	NEG	NEG	NEG	NEG	NEG	NEG	POS	POS	POS
**CC22-MRSA-IV [PVL+]**												
Riyadh-2781996-1	NEG	NEG	NEG	NEG	NEG	NEG	NEG	NEG	NEG	POS	POS	AMB
Riyadh-3103432	NEG	NEG	NEG	NEG	NEG	NEG	NEG	NEG	NEG	POS	POS	POS
Riyadh-3026502	NEG	NEG	NEG	NEG	NEG	NEG	NEG	NEG	NEG	POS	POS	AMB
Riyadh-3081378-2	NEG	NEG	NEG	NEG	NEG	NEG	NEG	NEG	NEG	POS	POS	AMB
Riyadh_IC_185	NEG	NEG	NEG	NEG	NEG	NEG	NEG	NEG	NEG	POS	POS	POS
Riyadh_IC_204-1	NEG	NEG	NEG	NEG	NEG	NEG	NEG	NEG	NEG	POS	POS	POS
Riyadh-2559371	NEG	NEG	NEG	NEG	NEG	NEG	NEG	NEG	NEG	POS	POS	POS
Riyadh-2753975	NEG	NEG	NEG	NEG	NEG	NEG	NEG	NEG	NEG	POS	POS	POS
Riyadh-2775605	NEG	NEG	NEG	NEG	NEG	NEG	NEG	NEG	NEG	POS	POS	AMB
Riyadh-2781996-2	NEG	NEG	NEG	NEG	NEG	NEG	NEG	NEG	NEG	POS	POS	AMB
Riyadh-2823783-2	NEG	NEG	NEG	NEG	NEG	NEG	NEG	NEG	NEG	POS	POS	POS
Riyadh-2876601	NEG	NEG	NEG	NEG	NEG	NEG	NEG	NEG	NEG	POS	POS	POS
Riyadh-3036074	NEG	NEG	NEG	NEG	NEG	NEG	NEG	NEG	NEG	POS	POS	POS
Riyadh-3053099	NEG	NEG	NEG	NEG	NEG	NEG	NEG	NEG	NEG	POS	POS	POS
Riyadh-3055366	NEG	NEG	NEG	NEG	NEG	NEG	NEG	NEG	NEG	POS	POS	POS
Riyadh-3082712	NEG	NEG	NEG	NEG	NEG	NEG	NEG	NEG	NEG	POS	POS	POS
Riyadh-3087502	NEG	NEG	NEG	NEG	NEG	NEG	NEG	NEG	NEG	POS	POS	AMB
Riyadh-6	NEG	NEG	NEG	NEG	NEG	NEG	NEG	NEG	NEG	POS	POS	POS
Riyadh-7	NEG	NEG	NEG	NEG	NEG	NEG	NEG	NEG	NEG	POS	POS	POS
Riyadh-8	NEG	NEG	NEG	NEG	NEG	NEG	NEG	NEG	NEG	POS	POS	POS
**CC30-MRSA-IV [PVL+], Southwest Pacific Clone**												
Riyadh-10	NEG	NEG	NEG	NEG	NEG	NEG	NEG	NEG	NEG	NEG	NEG	NEG
Riyadh_IC_123	NEG	NEG	NEG	NEG	NEG	NEG	NEG	NEG	NEG	POS	POS	POS
Riyadh-3080713	NEG	NEG	NEG	NEG	NEG	NEG	NEG	NEG	NEG	POS	POS	POS
Riyadh-2803856	NEG	NEG	NEG	NEG	NEG	NEG	NEG	NEG	NEG	POS	POS	POS
Riyadh-2817276-1	AMB	NEG	NEG	NEG	NEG	NEG	NEG	NEG	NEG	POS	POS	POS
Riyadh-2817571-2	NEG	NEG	NEG	NEG	NEG	NEG	NEG	NEG	NEG	POS	POS	POS
Riyadh-3033868	NEG	NEG	NEG	NEG	NEG	NEG	NEG	NEG	NEG	POS	POS	POS
Riyadh-2550108	NEG	NEG	NEG	NEG	NEG	NEG	NEG	NEG	NEG	POS	POS	POS
Riyadh-3095056-2	NEG	NEG	NEG	NEG	NEG	NEG	NEG	NEG	NEG	POS	POS	POS
Riyadh-2819899	NEG	NEG	NEG	NEG	NEG	NEG	NEG	NEG	NEG	POS	POS	POS
Riyadh-2821805	NEG	NEG	NEG	NEG	NEG	NEG	NEG	NEG	NEG	POS	POS	POS
Riyadh-3013928	NEG	NEG	NEG	NEG	NEG	NEG	NEG	NEG	NEG	POS	POS	POS
Riyadh-3029402	NEG	NEG	NEG	NEG	NEG	NEG	NEG	NEG	NEG	POS	POS	POS
**CC45/agr IV-MRSA-IV, WA MRSA-23**												
Riyadh-3081378-1	NEG	NEG	NEG	NEG	NEG	NEG	NEG	NEG	NEG	POS	POS	POS
**CC80-MRSA-IV**												
Riyadh-3107635	NEG	NEG	NEG	NEG	NEG	NEG	NEG	NEG	NEG	POS	POS	POS
Riyadh-2987458	NEG	NEG	NEG	NEG	NEG	NEG	NEG	NEG	NEG	POS	POS	POS
**CC80-MRSA-IV [PVL+], European caMRSA Clone**												
Riyadh-2988048	NEG	NEG	NEG	NEG	NEG	NEG	NEG	NEG	NEG	NEG	NEG	NEG
Riyadh-2990585-2	NEG	NEG	NEG	NEG	NEG	NEG	NEG	NEG	NEG	NEG	NEG	NEG
Riyadh-2990585-1	NEG	NEG	NEG	NEG	NEG	NEG	NEG	NEG	NEG	POS	POS	POS
Riyadh-2826033	NEG	NEG	NEG	NEG	NEG	NEG	NEG	NEG	NEG	NEG	NEG	NEG
Riyadh-1601562	NEG	NEG	NEG	NEG	NEG	NEG	NEG	NEG	NEG	POS	POS	POS
Riyadh-2569940	NEG	NEG	NEG	NEG	NEG	NEG	NEG	NEG	NEG	POS	POS	POS
Riyadh-2571692	NEG	NEG	NEG	NEG	NEG	NEG	NEG	NEG	NEG	POS	POS	POS
Riyadh-2763029	NEG	NEG	NEG	NEG	NEG	NEG	NEG	NEG	NEG	POS	POS	POS
Riyadh-2767090	NEG	NEG	NEG	NEG	NEG	NEG	NEG	NEG	NEG	POS	POS	POS
Riyadh-2775130	NEG	NEG	NEG	NEG	NEG	NEG	NEG	NEG	NEG	POS	POS	POS
Riyadh-2778256	NEG	NEG	NEG	NEG	NEG	NEG	NEG	NEG	NEG	POS	POS	POS
Riyadh-2817505	NEG	NEG	NEG	NEG	NEG	NEG	NEG	NEG	NEG	POS	POS	POS
Riyadh-3024912	NEG	NEG	NEG	NEG	NEG	NEG	NEG	NEG	NEG	POS	POS	POS
Riyadh-2788690	NEG	NEG	NEG	NEG	NEG	NEG	NEG	NEG	NEG	POS	POS	POS
Riyadh-2829034	NEG	NEG	NEG	NEG	NEG	NEG	NEG	NEG	NEG	POS	POS	POS
Riyadh-3	NEG	NEG	NEG	NEG	NEG	NEG	NEG	NEG	NEG	POS	POS	POS
Riyadh-5	NEG	NEG	NEG	NEG	NEG	NEG	NEG	NEG	NEG	POS	POS	POS
Riyadh-2553167	NEG	NEG	NEG	NEG	NEG	NEG	NEG	NEG	NEG	POS	POS	POS
Riyadh-3002592	NEG	NEG	NEG	NEG	NEG	NEG	NEG	NEG	NEG	POS	POS	POS
**CC88-MRSA-IV [PVL+]**												
Riyadh-2736996	NEG	NEG	NEG	NEG	NEG	NEG	NEG	NEG	NEG	POS	POS	POS
Riyadh-2942396	NEG	NEG	NEG	NEG	NEG	NEG	NEG	NEG	NEG	POS	POS	POS
Riyadh-3105391	NEG	NEG	NEG	NEG	NEG	NEG	NEG	NEG	NEG	POS	POS	POS
**CC97-MRSA-V**												
Riyadh-0297622	NEG	NEG	NEG	NEG	NEG	POS	POS	NEG	NEG	POS	POS	POS
Riyadh-3025471	NEG	NEG	NEG	NEG	POS	POS	POS	NEG	NEG	POS	POS	POS

	RESISTANCE : MLS-ANTIBIOTICS											
	ermA	ermB	ermC	linA	msrA	mefA	mpbBM	vatA	vatB	vga	vga-BM 3327	vgb
	rRNA adenine N-6-methyl-transferase, erythromycin/clindamycin	erythro-mycin/clindamycin resistance	erythro-mycin/clindamycin resistance	Linco-samid-Nucleo-tidyltrans-ferase	energy-dependent efflux of erythro-mycin	macrolide efflux protein A	probable lysylphos-phatidyl-glycerol synthetase	virginia-mycin A acetyltransferase	acetyl-transferase inactivating streptogramin A	ATP binding protein, streptogramin-A-resistance		virginia-mycin B hydrolase
**CC1-MRSA-IV&SCCfus, WA MRSA-1/45**												
Riyadh-3108609	NEG	NEG	NEG	NEG	NEG	NEG	NEG	NEG	NEG	NEG	NEG	NEG
**CC1/ST772-MRSA-V [PVL+], "Bengal Bay Clone/WA I**												
Riyadh-2819026	NEG	NEG	NEG	NEG	POS	NEG	POS	NEG	NEG	AMB	NEG	NEG
**CC5-MRSA-IV, Paediatric clone**												
Riyadh-2915327-1	NEG	NEG	POS	NEG	NEG	NEG	NEG	NEG	NEG	NEG	NEG	NEG
Riyadh-2915327-2	NEG	NEG	POS	NEG	NEG	NEG	NEG	NEG	NEG	NEG	NEG	NEG
Riyadh-2	NEG	NEG	NEG	NEG	NEG	NEG	NEG	NEG	NEG	NEG	NEG	NEG
**CC5-MRSA-IV [PVL+], Paediatric clone**												
Riyadh-2986666	NEG	NEG	POS	NEG	NEG	NEG	NEG	NEG	NEG	AMB	NEG	NEG
Riyadh-2913335	NEG	AMB	POS	NEG	NEG	NEG	NEG	NEG	NEG	NEG	NEG	NEG
**CC5-MRSA-IVvar, "Maltese Clone"**												
Riyadh-2983654	NEG	NEG	NEG	NEG	NEG	NEG	NEG	NEG	NEG	NEG	NEG	NEG
Riyadh-4	NEG	NEG	NEG	NEG	NEG	NEG	NEG	NEG	NEG	NEG	NEG	NEG
Riyadh-2790233	NEG	NEG	NEG	NEG	NEG	NEG	NEG	NEG	NEG	NEG	NEG	NEG
**CC5-MRSA-V**												
Riyadh-2568944	NEG	NEG	NEG	NEG	NEG	NEG	NEG	NEG	NEG	NEG	NEG	NEG
**CC6-MRSA-IV, WA MRSA-51/66**												
Riyadh-2556168	NEG	NEG	NEG	NEG	NEG	NEG	NEG	NEG	NEG	NEG	NEG	NEG
Riyadh-2824507	NEG	NEG	NEG	NEG	NEG	NEG	NEG	NEG	NEG	NEG	NEG	NEG
Riyadh-2990831	NEG	NEG	NEG	NEG	NEG	NEG	NEG	NEG	NEG	AMB	NEG	NEG
**CC8/ST239-MRSA-III, Vienna/Hungarian/Brazilian Cl**												
Riyadh-9	NEG	NEG	POS	NEG	NEG	NEG	NEG	NEG	NEG	NEG	NEG	NEG
Riyadh-3028763	POS	NEG	NEG	NEG	NEG	NEG	NEG	NEG	NEG	NEG	NEG	NEG
Riyadh-2817437	POS	NEG	NEG	NEG	NEG	NEG	NEG	NEG	NEG	AMB	NEG	NEG
Riyadh-2793706	POS	NEG	NEG	NEG	NEG	NEG	NEG	NEG	NEG	NEG	NEG	NEG
Riyadh-2818797	POS	NEG	NEG	NEG	NEG	NEG	NEG	NEG	NEG	NEG	NEG	NEG
Riyadh-2822825	POS	NEG	NEG	NEG	NEG	NEG	NEG	NEG	NEG	AMB	NEG	NEG
Riyadh-2888905	POS	NEG	NEG	NEG	NEG	NEG	NEG	NEG	NEG	NEG	NEG	NEG
Riyadh-2888915	POS	NEG	NEG	NEG	NEG	NEG	NEG	NEG	NEG	AMB	NEG	NEG
Riyadh-2567782	POS	NEG	NEG	NEG	NEG	NEG	NEG	NEG	NEG	NEG	NEG	NEG
Riyadh-2891670	POS	NEG	NEG	NEG	NEG	NEG	NEG	NEG	NEG	AMB	NEG	NEG
Riyadh-3006920	POS	NEG	NEG	NEG	NEG	NEG	NEG	NEG	NEG	NEG	NEG	NEG
Riyadh-2817276-1	POS	NEG	NEG	NEG	NEG	NEG	NEG	NEG	NEG	NEG	NEG	NEG
Riyadh-0295102	POS	NEG	NEG	NEG	NEG	NEG	NEG	NEG	NEG	NEG	NEG	NEG
Riyadh-2820597	POS	NEG	NEG	NEG	NEG	NEG	NEG	NEG	NEG	AMB	NEG	NEG
Riyadh-2822088	POS	NEG	NEG	NEG	NEG	NEG	NEG	NEG	NEG	AMB	NEG	NEG
Riyadh-3010092	POS	NEG	NEG	NEG	NEG	NEG	NEG	NEG	NEG	NEG	NEG	NEG
Riyadh-3022844	POS	NEG	NEG	NEG	NEG	NEG	NEG	NEG	NEG	NEG	NEG	NEG
Riyadh-3108214-2	POS	NEG	NEG	NEG	NEG	NEG	NEG	NEG	NEG	NEG	NEG	NEG
Riyadh-2823926	POS	NEG	NEG	NEG	NEG	NEG	NEG	NEG	NEG	NEG	NEG	NEG
Riyadh-1	POS	NEG	NEG	NEG	NEG	NEG	NEG	NEG	NEG	NEG	NEG	NEG
Riyadh-2818388	POS	NEG	NEG	NEG	NEG	NEG	NEG	NEG	NEG	NEG	NEG	NEG
Riyadh-2818316	POS	NEG	NEG	NEG	NEG	NEG	NEG	NEG	NEG	NEG	NEG	NEG
**CC9/ST834-MRSA-(atypical SCCmec)**												
Riyadh-3103521	NEG	NEG	NEG	NEG	POS	NEG	NEG	NEG	NEG	NEG	NEG	NEG
**CC22-MRSA-IV, Barnim/UK-EMRSA-15**												
Riyadh-2553359	NEG	NEG	NEG	NEG	NEG	NEG	NEG	NEG	NEG	NEG	NEG	NEG
Riyadh-2571758	NEG	NEG	NEG	NEG	NEG	NEG	NEG	NEG	NEG	NEG	NEG	NEG
Riyadh-3029203	NEG	NEG	NEG	NEG	NEG	NEG	NEG	NEG	NEG	NEG	NEG	NEG
Riyadh-3039785	NEG	NEG	NEG	NEG	NEG	NEG	NEG	NEG	NEG	NEG	NEG	NEG
Riyadh-3105594	NEG	NEG	NEG	NEG	NEG	NEG	NEG	NEG	NEG	NEG	NEG	NEG
Riyadh_IC_204-2	NEG	NEG	POS	NEG	NEG	NEG	NEG	NEG	NEG	AMB	NEG	NEG
Riyadh-3003974	NEG	NEG	NEG	NEG	POS	NEG	NEG	NEG	NEG	NEG	NEG	NEG
Riyadh_IC_067	NEG	NEG	NEG	NEG	NEG	NEG	NEG	NEG	NEG	NEG	NEG	NEG
Riyadh-2988627	NEG	NEG	NEG	NEG	NEG	NEG	NEG	NEG	NEG	NEG	NEG	NEG
Riyadh-3112581	NEG	NEG	NEG	NEG	NEG	NEG	NEG	NEG	NEG	NEG	NEG	NEG
**CC22-MRSA-IV [PVL+]**												
Riyadh-2781996-1	NEG	NEG	NEG	NEG	NEG	NEG	NEG	NEG	NEG	NEG	NEG	NEG
Riyadh-3103432	NEG	NEG	NEG	NEG	NEG	NEG	NEG	NEG	NEG	NEG	NEG	NEG
Riyadh-3026502	NEG	NEG	NEG	NEG	NEG	NEG	NEG	NEG	NEG	NEG	NEG	NEG
Riyadh-3081378-2	NEG	NEG	POS	NEG	NEG	NEG	NEG	NEG	NEG	NEG	NEG	NEG
Riyadh_IC_185	NEG	NEG	POS	NEG	NEG	NEG	NEG	NEG	NEG	NEG	NEG	NEG
Riyadh_IC_204-1	NEG	NEG	POS	NEG	NEG	NEG	NEG	NEG	NEG	AMB	NEG	NEG
Riyadh-2559371	NEG	NEG	POS	NEG	NEG	NEG	NEG	NEG	NEG	NEG	NEG	NEG
Riyadh-2753975	NEG	NEG	POS	NEG	NEG	NEG	NEG	NEG	NEG	NEG	NEG	NEG
Riyadh-2775605	NEG	NEG	POS	NEG	NEG	NEG	NEG	NEG	NEG	NEG	NEG	NEG
Riyadh-2781996-2	NEG	NEG	POS	NEG	NEG	NEG	NEG	NEG	NEG	NEG	NEG	NEG
Riyadh-2823783-2	NEG	NEG	POS	NEG	NEG	NEG	NEG	NEG	NEG	NEG	NEG	NEG
Riyadh-2876601	NEG	NEG	POS	NEG	NEG	NEG	NEG	NEG	NEG	NEG	NEG	NEG
Riyadh-3036074	NEG	NEG	POS	NEG	NEG	NEG	NEG	NEG	NEG	NEG	NEG	NEG
Riyadh-3053099	NEG	NEG	POS	NEG	NEG	NEG	NEG	NEG	NEG	NEG	NEG	NEG
Riyadh-3055366	NEG	NEG	POS	NEG	NEG	NEG	NEG	NEG	NEG	NEG	NEG	NEG
Riyadh-3082712	NEG	NEG	POS	NEG	NEG	NEG	NEG	NEG	NEG	NEG	NEG	NEG
Riyadh-3087502	NEG	NEG	POS	NEG	NEG	NEG	NEG	NEG	NEG	NEG	NEG	NEG
Riyadh-6	NEG	NEG	POS	NEG	NEG	NEG	NEG	NEG	NEG	NEG	NEG	NEG
Riyadh-7	NEG	NEG	POS	NEG	NEG	NEG	NEG	NEG	NEG	NEG	NEG	NEG
Riyadh-8	NEG	NEG	POS	NEG	NEG	NEG	NEG	NEG	NEG	NEG	NEG	NEG
**CC30-MRSA-IV [PVL+], Southwest Pacific Clone**												
Riyadh-10	NEG	NEG	POS	NEG	NEG	NEG	NEG	NEG	NEG	NEG	NEG	NEG
Riyadh_IC_123	NEG	NEG	NEG	NEG	NEG	NEG	NEG	NEG	NEG	NEG	NEG	NEG
Riyadh-3080713	NEG	NEG	NEG	NEG	NEG	NEG	NEG	NEG	NEG	NEG	NEG	NEG
Riyadh-2803856	NEG	NEG	NEG	NEG	NEG	NEG	NEG	NEG	NEG	NEG	NEG	NEG
Riyadh-2817276-1	NEG	NEG	NEG	NEG	NEG	NEG	NEG	NEG	NEG	AMB	NEG	NEG
Riyadh-2817571-2	NEG	NEG	NEG	NEG	NEG	NEG	NEG	NEG	NEG	NEG	NEG	NEG
Riyadh-3033868	NEG	NEG	NEG	NEG	NEG	NEG	NEG	NEG	NEG	NEG	NEG	NEG
Riyadh-2550108	NEG	NEG	NEG	NEG	POS	NEG	POS	NEG	NEG	NEG	NEG	NEG
Riyadh-3095056-2	NEG	NEG	NEG	NEG	POS	NEG	POS	NEG	NEG	NEG	NEG	NEG
Riyadh-2819899	NEG	NEG	NEG	NEG	POS	NEG	POS	NEG	NEG	NEG	NEG	NEG
Riyadh-2821805	NEG	NEG	NEG	NEG	POS	NEG	POS	NEG	NEG	NEG	NEG	NEG
Riyadh-3013928	NEG	NEG	NEG	NEG	POS	NEG	POS	NEG	NEG	NEG	NEG	NEG
Riyadh-3029402	NEG	NEG	NEG	NEG	POS	NEG	POS	NEG	NEG	NEG	NEG	NEG
**CC45/agr IV-MRSA-IV, WA MRSA-23**												
Riyadh-3081378-1	NEG	NEG	NEG	NEG	NEG	NEG	NEG	NEG	NEG	NEG	NEG	NEG
**CC80-MRSA-IV**												
Riyadh-3107635	NEG	NEG	POS	NEG	NEG	NEG	NEG	NEG	NEG	NEG	NEG	NEG
Riyadh-2987458	NEG	NEG	POS	NEG	NEG	NEG	NEG	NEG	NEG	NEG	NEG	NEG
**CC80-MRSA-IV [PVL+], European caMRSA Clone**												
Riyadh-2988048	NEG	NEG	NEG	NEG	NEG	NEG	NEG	NEG	NEG	NEG	NEG	NEG
Riyadh-2990585-2	NEG	NEG	NEG	NEG	NEG	NEG	NEG	NEG	NEG	NEG	NEG	NEG
Riyadh-2990585-1	NEG	NEG	NEG	NEG	NEG	NEG	NEG	NEG	NEG	NEG	NEG	NEG
Riyadh-2826033	NEG	NEG	NEG	NEG	NEG	NEG	NEG	NEG	NEG	NEG	NEG	NEG
Riyadh-1601562	NEG	NEG	NEG	NEG	NEG	NEG	NEG	NEG	NEG	NEG	NEG	NEG
Riyadh-2569940	NEG	NEG	NEG	NEG	NEG	NEG	NEG	NEG	NEG	NEG	NEG	NEG
Riyadh-2571692	NEG	NEG	NEG	NEG	NEG	NEG	NEG	NEG	NEG	NEG	NEG	NEG
Riyadh-2763029	NEG	NEG	NEG	NEG	NEG	NEG	NEG	NEG	NEG	NEG	NEG	NEG
Riyadh-2767090	NEG	NEG	NEG	NEG	NEG	NEG	NEG	NEG	NEG	NEG	NEG	NEG
Riyadh-2775130	NEG	NEG	NEG	NEG	NEG	NEG	NEG	NEG	NEG	NEG	NEG	NEG
Riyadh-2778256	NEG	NEG	NEG	NEG	NEG	NEG	NEG	NEG	NEG	NEG	NEG	NEG
Riyadh-2817505	NEG	NEG	NEG	NEG	NEG	NEG	NEG	NEG	NEG	NEG	NEG	NEG
Riyadh-3024912	NEG	NEG	NEG	NEG	NEG	NEG	NEG	NEG	NEG	NEG	NEG	NEG
Riyadh-2788690	NEG	NEG	POS	NEG	NEG	NEG	NEG	NEG	NEG	NEG	NEG	NEG
Riyadh-2829034	NEG	NEG	POS	NEG	NEG	NEG	NEG	NEG	NEG	AMB	NEG	NEG
Riyadh-3	NEG	NEG	POS	NEG	NEG	NEG	NEG	NEG	NEG	NEG	NEG	NEG
Riyadh-5	NEG	NEG	POS	NEG	NEG	NEG	NEG	NEG	NEG	NEG	NEG	NEG
Riyadh-2553167	NEG	NEG	NEG	NEG	NEG	NEG	NEG	NEG	NEG	NEG	NEG	NEG
Riyadh-3002592	NEG	NEG	NEG	NEG	NEG	NEG	NEG	NEG	NEG	NEG	NEG	NEG
**CC88-MRSA-IV [PVL+]**												
Riyadh-2736996	NEG	NEG	NEG	NEG	NEG	NEG	NEG	NEG	NEG	NEG	NEG	NEG
Riyadh-2942396	NEG	NEG	NEG	NEG	NEG	NEG	NEG	NEG	NEG	AMB	NEG	NEG
Riyadh-3105391	NEG	NEG	NEG	NEG	NEG	NEG	NEG	NEG	NEG	NEG	NEG	NEG
**CC97-MRSA-V**												
Riyadh-0297622	NEG	NEG	NEG	NEG	NEG	NEG	NEG	NEG	NEG	NEG	NEG	NEG
Riyadh-3025471	NEG	NEG	NEG	NEG	NEG	NEG	NEG	NEG	NEG	NEG	NEG	NEG

	RESISTANCE : AMINOGLYSOSIDES			RESISTANCE : MISCELLANEOUS GENES									
	aacA-aphD	aadD	aphA3	sat	dfrA	far1	Q6GD50	mupR	tetK	tetM	cat (total)	cat (pC221)	cat (pMC524)
	bifunctional enzyme Aac/Aph, gentamicin resistance	amino-glycoside adenyl-transferase,tobramycin resistance	3'5'-aminog-lycoside phospho-transferase, neo-/ kanamycin	strepto-thricine-acetyl-transferase	dihydro-folate reductase type 1	fusidic acid resistence	hypothetical protein associated with fusidic acid resistance	mupirocin resistence protein	tetrazyklin-resistance	tetracycline-resistance	chloramphenicol acetyltransferase		
**CC1-MRSA-IV&SCCfus, WA MRSA-1/45**													
Riyadh-3108609	NEG	NEG	NEG	NEG	NEG	NEG	POS	NEG	NEG	NEG	NEG	NEG	NEG
**CC1/ST772-MRSA-V [PVL+], "Bengal Bay Clone/WA I**													
Riyadh-2819026	POS	NEG	POS	POS	NEG	NEG	NEG	NEG	NEG	NEG	NEG	NEG	NEG
**CC5-MRSA-IV, Paediatric clone**													
Riyadh-2915327-1	NEG	NEG	NEG	NEG	NEG	NEG	NEG	NEG	NEG	NEG	NEG	NEG	NEG
Riyadh-2915327-2	NEG	NEG	NEG	NEG	NEG	NEG	NEG	NEG	NEG	NEG	NEG	NEG	NEG
Riyadh-2	NEG	NEG	NEG	NEG	NEG	NEG	NEG	NEG	NEG	NEG	NEG	NEG	NEG
**CC5-MRSA-IV [PVL+], Paediatric clone**													
Riyadh-2986666	NEG	NEG	POS	POS	NEG	NEG	NEG	NEG	NEG	NEG	NEG	NEG	NEG
Riyadh-2913335	NEG	NEG	NEG	AMB	NEG	NEG	NEG	NEG	NEG	NEG	NEG	NEG	NEG
**CC5-MRSA-IVvar, "Maltese Clone"**													
Riyadh-2983654	NEG	NEG	NEG	NEG	NEG	NEG	POS	NEG	NEG	NEG	NEG	NEG	NEG
Riyadh-4	NEG	NEG	NEG	NEG	NEG	NEG	POS	NEG	NEG	NEG	NEG	NEG	NEG
Riyadh-2790233	NEG	NEG	NEG	NEG	NEG	NEG	POS	NEG	NEG	NEG	NEG	NEG	NEG
**CC5-MRSA-V**													
Riyadh-2568944	POS	NEG	NEG	NEG	NEG	NEG	NEG	NEG	NEG	NEG	NEG	NEG	NEG
**CC6-MRSA-IV, WA MRSA-51/66**													
Riyadh-2556168	NEG	NEG	NEG	NEG	NEG	NEG	NEG	NEG	NEG	NEG	NEG	NEG	NEG
Riyadh-2824507	NEG	NEG	NEG	NEG	NEG	NEG	NEG	NEG	NEG	NEG	NEG	NEG	NEG
Riyadh-2990831	NEG	NEG	NEG	NEG	NEG	NEG	NEG	NEG	NEG	NEG	NEG	NEG	NEG
**CC8/ST239-MRSA-III, Vienna/Hungarian/Brazilian Cl**													
Riyadh-9	POS	NEG	POS	POS	NEG	NEG	NEG	NEG	POS	POS	NEG	NEG	NEG
Riyadh-3028763	POS	NEG	POS	POS	NEG	NEG	NEG	NEG	NEG	POS	NEG	NEG	NEG
Riyadh-2817437	POS	NEG	POS	POS	NEG	NEG	NEG	NEG	NEG	POS	NEG	NEG	NEG
Riyadh-2793706	NEG	NEG	NEG	NEG	NEG	NEG	NEG	NEG	NEG	POS	NEG	NEG	NEG
Riyadh-2818797	NEG	NEG	NEG	NEG	NEG	NEG	NEG	NEG	NEG	POS	NEG	NEG	NEG
Riyadh-2822825	NEG	NEG	NEG	NEG	NEG	NEG	NEG	NEG	NEG	POS	NEG	NEG	NEG
Riyadh-2888905	NEG	NEG	NEG	NEG	NEG	NEG	NEG	NEG	NEG	POS	NEG	NEG	NEG
Riyadh-2888915	NEG	NEG	NEG	NEG	NEG	NEG	NEG	NEG	NEG	POS	NEG	NEG	NEG
Riyadh-2567782	NEG	NEG	NEG	NEG	NEG	NEG	NEG	NEG	NEG	POS	NEG	NEG	NEG
Riyadh-2891670	NEG	NEG	NEG	NEG	NEG	NEG	NEG	NEG	NEG	POS	NEG	NEG	NEG
Riyadh-3006920	NEG	NEG	NEG	NEG	NEG	NEG	NEG	NEG	NEG	POS	NEG	NEG	NEG
Riyadh-2817276-1	NEG	NEG	NEG	NEG	NEG	NEG	NEG	NEG	POS	POS	NEG	NEG	NEG
Riyadh-0295102	POS	NEG	POS	POS	NEG	NEG	NEG	NEG	NEG	POS	NEG	NEG	NEG
Riyadh-2820597	POS	NEG	POS	POS	NEG	NEG	NEG	NEG	NEG	POS	NEG	NEG	NEG
Riyadh-2822088	POS	NEG	POS	POS	NEG	NEG	NEG	NEG	NEG	POS	NEG	NEG	NEG
Riyadh-3010092	POS	NEG	POS	POS	NEG	NEG	NEG	NEG	NEG	POS	NEG	NEG	NEG
Riyadh-3022844	POS	NEG	POS	POS	NEG	NEG	NEG	NEG	NEG	POS	NEG	NEG	NEG
Riyadh-3108214-2	POS	NEG	POS	POS	NEG	NEG	NEG	NEG	NEG	POS	NEG	NEG	NEG
Riyadh-2823926	POS	NEG	POS	POS	NEG	NEG	NEG	NEG	NEG	POS	NEG	NEG	NEG
Riyadh-1	POS	NEG	POS	POS	NEG	NEG	NEG	NEG	NEG	POS	NEG	NEG	NEG
Riyadh-2818388	POS	NEG	POS	POS	NEG	NEG	NEG	NEG	POS	POS	NEG	NEG	NEG
Riyadh-2818316	POS	NEG	POS	POS	NEG	NEG	NEG	NEG	NEG	POS	POS	AMB	POS
**CC9/ST834-MRSA-(atypical SCCmec)**													
Riyadh-3103521	NEG	NEG	NEG	NEG	NEG	NEG	POS	NEG	NEG	NEG	NEG	NEG	NEG
**CC22-MRSA-IV, Barnim/UK-EMRSA-15**													
Riyadh-2553359	NEG	NEG	NEG	NEG	POS	NEG	NEG	NEG	NEG	NEG	NEG	NEG	NEG
Riyadh-2571758	NEG	NEG	NEG	NEG	POS	NEG	NEG	NEG	NEG	NEG	NEG	NEG	NEG
Riyadh-3029203	NEG	NEG	NEG	NEG	POS	NEG	NEG	NEG	NEG	NEG	NEG	NEG	NEG
Riyadh-3039785	NEG	NEG	NEG	NEG	POS	NEG	NEG	NEG	NEG	NEG	NEG	NEG	NEG
Riyadh-3105594	NEG	NEG	NEG	NEG	POS	NEG	NEG	NEG	NEG	NEG	NEG	NEG	NEG
Riyadh_IC_204-2	POS	NEG	NEG	AMB	POS	NEG	NEG	NEG	NEG	NEG	NEG	NEG	NEG
Riyadh-3003974	NEG	NEG	NEG	NEG	POS	NEG	NEG	NEG	NEG	NEG	NEG	NEG	NEG
Riyadh_IC_067	NEG	NEG	NEG	NEG	POS	NEG	NEG	NEG	POS	NEG	NEG	NEG	NEG
Riyadh-2988627	NEG	NEG	NEG	NEG	POS	NEG	NEG	NEG	POS	NEG	NEG	NEG	NEG
Riyadh-3112581	NEG	NEG	NEG	NEG	POS	NEG	NEG	NEG	POS	NEG	NEG	NEG	NEG
**CC22-MRSA-IV [PVL+]**													
Riyadh-2781996-1	POS	NEG	NEG	NEG	POS	NEG	NEG	NEG	NEG	NEG	NEG	NEG	NEG
Riyadh-3103432	POS	NEG	NEG	NEG	POS	NEG	NEG	NEG	NEG	NEG	NEG	NEG	NEG
Riyadh-3026502	POS	POS	NEG	NEG	NEG	NEG	NEG	NEG	NEG	NEG	NEG	NEG	NEG
Riyadh-3081378-2	POS	POS	NEG	NEG	POS	NEG	NEG	NEG	NEG	NEG	NEG	NEG	NEG
Riyadh_IC_185	POS	NEG	NEG	AMB	POS	NEG	NEG	NEG	NEG	NEG	NEG	NEG	NEG
Riyadh_IC_204-1	POS	NEG	NEG	AMB	POS	NEG	NEG	NEG	NEG	NEG	NEG	NEG	NEG
Riyadh-2559371	POS	POS	NEG	NEG	POS	NEG	NEG	NEG	NEG	NEG	NEG	NEG	NEG
Riyadh-2753975	POS	NEG	NEG	NEG	POS	NEG	NEG	NEG	NEG	NEG	NEG	NEG	NEG
Riyadh-2775605	POS	NEG	NEG	NEG	POS	NEG	NEG	NEG	NEG	NEG	NEG	NEG	NEG
Riyadh-2781996-2	POS	NEG	NEG	NEG	POS	NEG	NEG	NEG	NEG	NEG	NEG	NEG	NEG
Riyadh-2823783-2	POS	NEG	NEG	NEG	POS	NEG	NEG	NEG	NEG	NEG	NEG	NEG	NEG
Riyadh-2876601	POS	POS	NEG	NEG	POS	NEG	NEG	NEG	NEG	NEG	NEG	NEG	NEG
Riyadh-3036074	POS	NEG	NEG	NEG	POS	NEG	NEG	NEG	NEG	NEG	NEG	NEG	NEG
Riyadh-3053099	POS	POS	NEG	NEG	POS	NEG	NEG	NEG	NEG	NEG	NEG	NEG	NEG
Riyadh-3055366	POS	POS	NEG	NEG	POS	NEG	NEG	NEG	NEG	NEG	NEG	NEG	NEG
Riyadh-3082712	POS	POS	NEG	NEG	POS	NEG	NEG	NEG	NEG	NEG	NEG	NEG	NEG
Riyadh-3087502	POS	POS	NEG	NEG	AMB	NEG	NEG	NEG	NEG	NEG	NEG	NEG	NEG
Riyadh-6	POS	NEG	NEG	NEG	POS	NEG	NEG	NEG	NEG	NEG	NEG	NEG	NEG
Riyadh-7	POS	NEG	NEG	NEG	POS	NEG	NEG	NEG	NEG	NEG	NEG	NEG	NEG
Riyadh-8	POS	NEG	NEG	NEG	POS	NEG	NEG	NEG	NEG	NEG	NEG	NEG	NEG
**CC30-MRSA-IV [PVL+], Southwest Pacific Clone**													
Riyadh-10	NEG	NEG	NEG	NEG	NEG	NEG	NEG	NEG	NEG	NEG	NEG	NEG	NEG
Riyadh_IC_123	NEG	NEG	NEG	NEG	NEG	NEG	NEG	NEG	NEG	NEG	NEG	NEG	NEG
Riyadh-3080713	NEG	NEG	NEG	NEG	NEG	NEG	NEG	NEG	NEG	NEG	NEG	NEG	NEG
Riyadh-2803856	NEG	NEG	NEG	NEG	NEG	NEG	NEG	NEG	NEG	NEG	NEG	NEG	NEG
Riyadh-2817276-1	NEG	NEG	NEG	NEG	NEG	NEG	NEG	NEG	NEG	NEG	NEG	NEG	NEG
Riyadh-2817571-2	NEG	NEG	NEG	NEG	NEG	NEG	NEG	NEG	NEG	NEG	NEG	NEG	NEG
Riyadh-3033868	NEG	NEG	NEG	NEG	NEG	NEG	NEG	NEG	NEG	NEG	NEG	NEG	NEG
Riyadh-2550108	NEG	NEG	POS	POS	NEG	NEG	NEG	NEG	NEG	NEG	NEG	NEG	NEG
Riyadh-3095056-2	NEG	NEG	POS	POS	NEG	NEG	NEG	NEG	NEG	NEG	NEG	NEG	NEG
Riyadh-2819899	NEG	NEG	POS	POS	NEG	NEG	NEG	NEG	NEG	NEG	NEG	NEG	NEG
Riyadh-2821805	NEG	NEG	POS	POS	NEG	NEG	NEG	NEG	NEG	NEG	NEG	NEG	NEG
Riyadh-3013928	NEG	NEG	POS	POS	NEG	NEG	NEG	NEG	NEG	NEG	NEG	NEG	NEG
Riyadh-3029402	NEG	NEG	POS	POS	NEG	NEG	NEG	NEG	NEG	NEG	NEG	NEG	NEG
**CC45/agr IV-MRSA-IV, WA MRSA-23**													
Riyadh-3081378-1	NEG	NEG	NEG	NEG	NEG	NEG	NEG	NEG	NEG	NEG	NEG	NEG	NEG
**CC80-MRSA-IV**													
Riyadh-3107635	NEG	NEG	POS	POS	NEG	POS	NEG	NEG	NEG	NEG	NEG	NEG	NEG
Riyadh-2987458	NEG	NEG	NEG	AMB	NEG	NEG	NEG	NEG	NEG	NEG	NEG	NEG	NEG
**CC80-MRSA-IV [PVL+], European caMRSA Clone**													
Riyadh-2988048	NEG	NEG	NEG	NEG	NEG	NEG	NEG	NEG	NEG	NEG	NEG	NEG	NEG
Riyadh-2990585-2	NEG	NEG	NEG	NEG	NEG	NEG	NEG	NEG	NEG	NEG	NEG	NEG	NEG
Riyadh-2990585-1	NEG	NEG	NEG	NEG	NEG	POS	NEG	NEG	NEG	NEG	NEG	NEG	NEG
Riyadh-2826033	NEG	NEG	POS	POS	NEG	NEG	NEG	NEG	NEG	NEG	NEG	NEG	NEG
Riyadh-1601562	NEG	NEG	POS	POS	NEG	POS	NEG	NEG	NEG	NEG	NEG	NEG	NEG
Riyadh-2569940	NEG	NEG	POS	POS	NEG	POS	NEG	NEG	NEG	NEG	NEG	NEG	NEG
Riyadh-2571692	NEG	NEG	POS	POS	NEG	POS	NEG	NEG	NEG	NEG	NEG	NEG	NEG
Riyadh-2763029	NEG	NEG	POS	POS	NEG	POS	NEG	NEG	NEG	NEG	NEG	NEG	NEG
Riyadh-2767090	NEG	NEG	POS	POS	NEG	POS	NEG	NEG	NEG	NEG	NEG	NEG	NEG
Riyadh-2775130	NEG	NEG	POS	POS	NEG	POS	NEG	NEG	NEG	NEG	NEG	NEG	NEG
Riyadh-2778256	NEG	NEG	POS	POS	NEG	POS	NEG	NEG	NEG	NEG	NEG	NEG	NEG
Riyadh-2817505	NEG	NEG	POS	POS	NEG	POS	NEG	NEG	NEG	NEG	NEG	NEG	NEG
Riyadh-3024912	NEG	NEG	POS	POS	NEG	POS	NEG	NEG	NEG	NEG	NEG	NEG	NEG
Riyadh-2788690	NEG	NEG	POS	POS	NEG	POS	NEG	NEG	NEG	NEG	NEG	NEG	NEG
Riyadh-2829034	NEG	NEG	POS	POS	NEG	POS	NEG	NEG	NEG	NEG	NEG	NEG	NEG
Riyadh-3	NEG	NEG	POS	POS	NEG	POS	NEG	NEG	NEG	NEG	NEG	NEG	NEG
Riyadh-5	NEG	NEG	POS	POS	NEG	POS	NEG	NEG	NEG	NEG	NEG	NEG	NEG
Riyadh-2553167	NEG	NEG	POS	POS	NEG	POS	NEG	NEG	POS	NEG	NEG	NEG	NEG
Riyadh-3002592	NEG	NEG	POS	POS	NEG	POS	NEG	NEG	POS	NEG	NEG	NEG	NEG
**CC88-MRSA-IV [PVL+]**													
Riyadh-2736996	NEG	NEG	NEG	NEG	NEG	NEG	NEG	NEG	POS	NEG	NEG	NEG	NEG
Riyadh-2942396	NEG	NEG	NEG	NEG	NEG	NEG	NEG	NEG	NEG	NEG	NEG	NEG	NEG
Riyadh-3105391	NEG	NEG	NEG	NEG	NEG	NEG	NEG	NEG	POS	NEG	NEG	NEG	NEG
**CC97-MRSA-V**													
Riyadh-0297622	POS	NEG	NEG	NEG	NEG	NEG	POS	NEG	POS	NEG	NEG	NEG	NEG
Riyadh-3025471	NEG	NEG	NEG	NEG	NEG	NEG	POS	NEG	NEG	NEG	NEG	NEG	NEG

	RESISTANCE : MISCELLANEOUS GENES				RESISTANCE : EFFLUX SYSTEMS						RESISTANCE : GLYCOPEPTIDES		
	cfr	fexA	fosB	fosB-plasmid	qacA	qacC (total)	qacC (cons)	qacC (equine)	qacC (SA5, Ssap, ST94)	tetEfflux	vanA	vanB	vanZ
	23S rRNA methyltransferase	chloramphenicol/florfenicol exporter	metallothiol transferase		quaternary ammonium compound resistance protein A	quaternary ammonium compound resistance protein C				Transport-/Effluxprotein	vancomycin resistance gene	vancomycin resistance gene from enterococci and Clostridium	teicoplanin resistance gene from enterococci
**CC1-MRSA-IV&SCCfus, WA MRSA-1/45**													
Riyadh-3108609	NEG	NEG	NEG	NEG	NEG	NEG	NEG	NEG	NEG	POS	NEG	NEG	NEG
**CC1/ST772-MRSA-V [PVL+], "Bengal Bay Clone/WA I**													
Riyadh-2819026	NEG	NEG	POS	AMB	NEG	NEG	NEG	NEG	NEG	POS	NEG	NEG	NEG
**CC5-MRSA-IV, Paediatric clone**													
Riyadh-2915327-1	NEG	NEG	POS	NEG	NEG	NEG	NEG	NEG	NEG	POS	NEG	NEG	NEG
Riyadh-2915327-2	NEG	NEG	POS	NEG	NEG	NEG	NEG	NEG	NEG	POS	NEG	NEG	NEG
Riyadh-2	NEG	NEG	POS	NEG	NEG	NEG	NEG	NEG	NEG	POS	NEG	NEG	NEG
**CC5-MRSA-IV [PVL+], Paediatric clone**													
Riyadh-2986666	NEG	NEG	POS	AMB	NEG	NEG	NEG	NEG	NEG	POS	NEG	NEG	NEG
Riyadh-2913335	NEG	NEG	POS	NEG	NEG	NEG	NEG	NEG	NEG	POS	NEG	NEG	NEG
**CC5-MRSA-IVvar, "Maltese Clone"**													
Riyadh-2983654	NEG	NEG	POS	NEG	NEG	NEG	NEG	NEG	NEG	POS	NEG	NEG	NEG
Riyadh-4	NEG	NEG	POS	NEG	NEG	NEG	NEG	NEG	NEG	POS	NEG	NEG	NEG
Riyadh-2790233	NEG	NEG	POS	NEG	NEG	NEG	NEG	NEG	NEG	POS	NEG	NEG	NEG
**CC5-MRSA-V**													
Riyadh-2568944	NEG	NEG	POS	NEG	NEG	NEG	NEG	NEG	NEG	POS	NEG	NEG	NEG
**CC6-MRSA-IV, WA MRSA-51/66**													
Riyadh-2556168	NEG	NEG	POS	NEG	NEG	NEG	NEG	NEG	NEG	POS	NEG	NEG	NEG
Riyadh-2824507	NEG	NEG	POS	NEG	NEG	NEG	NEG	NEG	NEG	POS	NEG	NEG	NEG
Riyadh-2990831	NEG	NEG	POS	AMB	NEG	NEG	NEG	NEG	NEG	POS	NEG	NEG	NEG
**CC8/ST239-MRSA-III, Vienna/Hungarian/Brazilian Cl**													
Riyadh-9	NEG	NEG	POS	AMB	NEG	NEG	NEG	NEG	NEG	POS	NEG	NEG	NEG
Riyadh-3028763	NEG	NEG	POS	NEG	POS	NEG	NEG	NEG	NEG	POS	NEG	NEG	NEG
Riyadh-2817437	NEG	NEG	POS	AMB	POS	NEG	NEG	NEG	NEG	POS	NEG	NEG	NEG
Riyadh-2793706	NEG	NEG	POS	AMB	POS	NEG	NEG	NEG	NEG	POS	NEG	NEG	NEG
Riyadh-2818797	NEG	NEG	POS	AMB	POS	NEG	NEG	NEG	NEG	POS	NEG	NEG	NEG
Riyadh-2822825	NEG	NEG	POS	AMB	POS	NEG	NEG	NEG	NEG	POS	NEG	NEG	NEG
Riyadh-2888905	NEG	NEG	POS	AMB	POS	NEG	NEG	NEG	NEG	POS	NEG	NEG	NEG
Riyadh-2888915	NEG	NEG	POS	AMB	POS	NEG	NEG	NEG	NEG	POS	NEG	NEG	NEG
Riyadh-2567782	NEG	NEG	POS	NEG	POS	NEG	NEG	NEG	NEG	POS	NEG	NEG	NEG
Riyadh-2891670	NEG	NEG	POS	AMB	POS	NEG	NEG	NEG	NEG	POS	NEG	NEG	NEG
Riyadh-3006920	NEG	NEG	POS	NEG	POS	NEG	NEG	NEG	NEG	POS	NEG	NEG	NEG
Riyadh-2817276-1	NEG	NEG	POS	AMB	POS	NEG	NEG	NEG	NEG	POS	NEG	NEG	NEG
Riyadh-0295102	NEG	NEG	POS	NEG	POS	NEG	NEG	NEG	NEG	POS	NEG	NEG	NEG
Riyadh-2820597	NEG	NEG	POS	AMB	POS	NEG	NEG	NEG	NEG	POS	NEG	NEG	NEG
Riyadh-2822088	NEG	NEG	POS	AMB	POS	NEG	NEG	NEG	NEG	POS	NEG	NEG	NEG
Riyadh-3010092	NEG	NEG	POS	NEG	NEG	NEG	NEG	NEG	NEG	POS	NEG	NEG	NEG
Riyadh-3022844	NEG	NEG	POS	NEG	POS	NEG	NEG	NEG	NEG	POS	NEG	NEG	NEG
Riyadh-3108214-2	NEG	NEG	POS	NEG	POS	NEG	NEG	NEG	NEG	POS	NEG	NEG	NEG
Riyadh-2823926	NEG	NEG	POS	AMB	POS	NEG	NEG	NEG	NEG	POS	NEG	NEG	NEG
Riyadh-1	NEG	NEG	POS	AMB	POS	NEG	NEG	NEG	NEG	POS	NEG	NEG	NEG
Riyadh-2818388	NEG	NEG	POS	AMB	POS	NEG	NEG	NEG	NEG	POS	NEG	NEG	NEG
Riyadh-2818316	NEG	NEG	POS	NEG	POS	NEG	NEG	NEG	NEG	POS	NEG	NEG	NEG
**CC9/ST834-MRSA-(atypical SCCmec)**													
Riyadh-3103521	NEG	NEG	POS	NEG	NEG	NEG	NEG	NEG	NEG	POS	NEG	NEG	NEG
**CC22-MRSA-IV, Barnim/UK-EMRSA-15**													
Riyadh-2553359	NEG	NEG	NEG	NEG	NEG	NEG	NEG	NEG	NEG	NEG	NEG	NEG	NEG
Riyadh-2571758	NEG	NEG	NEG	NEG	NEG	NEG	NEG	NEG	NEG	NEG	NEG	NEG	NEG
Riyadh-3029203	NEG	NEG	NEG	NEG	NEG	NEG	NEG	NEG	NEG	NEG	NEG	NEG	NEG
Riyadh-3039785	NEG	NEG	NEG	NEG	NEG	NEG	NEG	NEG	NEG	NEG	NEG	NEG	NEG
Riyadh-3105594	NEG	NEG	NEG	NEG	NEG	NEG	NEG	NEG	NEG	NEG	NEG	NEG	NEG
Riyadh_IC_204-2	NEG	NEG	NEG	NEG	NEG	NEG	NEG	NEG	NEG	NEG	NEG	NEG	NEG
Riyadh-3003974	NEG	NEG	NEG	NEG	NEG	NEG	NEG	NEG	NEG	NEG	NEG	NEG	NEG
Riyadh_IC_067	NEG	NEG	NEG	NEG	NEG	NEG	NEG	NEG	NEG	NEG	NEG	NEG	NEG
Riyadh-2988627	NEG	NEG	NEG	NEG	NEG	NEG	NEG	NEG	NEG	NEG	NEG	NEG	NEG
Riyadh-3112581	NEG	NEG	NEG	NEG	NEG	NEG	NEG	NEG	NEG	NEG	NEG	NEG	NEG
**CC22-MRSA-IV [PVL+]**													
Riyadh-2781996-1	NEG	NEG	NEG	NEG	NEG	NEG	NEG	NEG	NEG	NEG	NEG	NEG	NEG
Riyadh-3103432	NEG	NEG	NEG	NEG	NEG	NEG	NEG	NEG	NEG	NEG	NEG	NEG	NEG
Riyadh-3026502	NEG	NEG	NEG	NEG	NEG	NEG	NEG	NEG	NEG	NEG	NEG	NEG	NEG
Riyadh-3081378-2	NEG	NEG	NEG	NEG	NEG	NEG	NEG	NEG	NEG	NEG	NEG	NEG	NEG
Riyadh_IC_185	NEG	NEG	NEG	NEG	NEG	NEG	NEG	NEG	NEG	NEG	NEG	NEG	NEG
Riyadh_IC_204-1	NEG	NEG	NEG	NEG	NEG	NEG	NEG	NEG	NEG	NEG	NEG	NEG	NEG
Riyadh-2559371	NEG	NEG	NEG	NEG	NEG	NEG	NEG	NEG	NEG	NEG	NEG	NEG	NEG
Riyadh-2753975	NEG	NEG	NEG	NEG	NEG	NEG	NEG	NEG	NEG	NEG	NEG	NEG	NEG
Riyadh-2775605	NEG	NEG	NEG	NEG	NEG	NEG	NEG	NEG	NEG	NEG	NEG	NEG	NEG
Riyadh-2781996-2	NEG	NEG	NEG	NEG	NEG	NEG	NEG	NEG	NEG	NEG	NEG	NEG	NEG
Riyadh-2823783-2	NEG	NEG	NEG	NEG	NEG	NEG	NEG	NEG	NEG	NEG	NEG	NEG	NEG
Riyadh-2876601	NEG	NEG	NEG	NEG	NEG	NEG	NEG	NEG	NEG	NEG	NEG	NEG	NEG
Riyadh-3036074	NEG	NEG	NEG	NEG	NEG	NEG	NEG	NEG	NEG	NEG	NEG	NEG	NEG
Riyadh-3053099	NEG	NEG	NEG	NEG	NEG	NEG	NEG	NEG	NEG	NEG	NEG	NEG	NEG
Riyadh-3055366	NEG	NEG	NEG	NEG	NEG	NEG	NEG	NEG	NEG	NEG	NEG	NEG	NEG
Riyadh-3082712	NEG	NEG	NEG	NEG	NEG	NEG	NEG	NEG	NEG	NEG	NEG	NEG	NEG
Riyadh-3087502	NEG	NEG	NEG	NEG	NEG	NEG	NEG	NEG	NEG	NEG	NEG	NEG	NEG
Riyadh-6	NEG	NEG	NEG	NEG	NEG	NEG	NEG	NEG	NEG	NEG	NEG	NEG	NEG
Riyadh-7	NEG	NEG	NEG	NEG	NEG	NEG	NEG	NEG	NEG	NEG	NEG	NEG	NEG
Riyadh-8	NEG	NEG	NEG	NEG	NEG	NEG	NEG	NEG	NEG	NEG	NEG	NEG	NEG
**CC30-MRSA-IV [PVL+], Southwest Pacific Clone**													
Riyadh-10	NEG	NEG	POS	NEG	NEG	NEG	NEG	NEG	NEG	POS	NEG	NEG	NEG
Riyadh_IC_123	NEG	NEG	POS	NEG	NEG	NEG	NEG	NEG	NEG	POS	NEG	NEG	NEG
Riyadh-3080713	NEG	NEG	POS	NEG	NEG	NEG	NEG	NEG	NEG	POS	NEG	NEG	NEG
Riyadh-2803856	NEG	NEG	POS	NEG	NEG	NEG	NEG	NEG	NEG	POS	NEG	NEG	NEG
Riyadh-2817276-1	NEG	NEG	POS	AMB	AMB	NEG	NEG	NEG	NEG	POS	NEG	NEG	NEG
Riyadh-2817571-2	NEG	NEG	POS	NEG	NEG	NEG	NEG	NEG	NEG	POS	NEG	NEG	NEG
Riyadh-3033868	NEG	NEG	POS	NEG	NEG	NEG	NEG	NEG	NEG	POS	NEG	NEG	NEG
Riyadh-2550108	NEG	NEG	POS	NEG	NEG	NEG	NEG	NEG	NEG	POS	NEG	NEG	NEG
Riyadh-3095056-2	NEG	NEG	POS	NEG	NEG	NEG	NEG	NEG	NEG	POS	NEG	NEG	NEG
Riyadh-2819899	NEG	NEG	POS	NEG	NEG	NEG	NEG	NEG	NEG	POS	NEG	NEG	NEG
Riyadh-2821805	NEG	NEG	POS	NEG	NEG	NEG	NEG	NEG	NEG	POS	NEG	NEG	NEG
Riyadh-3013928	NEG	NEG	POS	NEG	NEG	NEG	NEG	NEG	NEG	POS	NEG	NEG	NEG
Riyadh-3029402	NEG	NEG	POS	NEG	NEG	NEG	NEG	NEG	NEG	POS	NEG	NEG	NEG
**CC45/agr IV-MRSA-IV, WA MRSA-23**													
Riyadh-3081378-1	NEG	NEG	NEG	NEG	NEG	NEG	NEG	NEG	NEG	POS	NEG	NEG	NEG
**CC80-MRSA-IV**													
Riyadh-3107635	NEG	NEG	NEG	NEG	NEG	NEG	NEG	NEG	NEG	POS	NEG	NEG	NEG
Riyadh-2987458	NEG	NEG	NEG	NEG	NEG	NEG	NEG	NEG	NEG	POS	NEG	NEG	NEG
**CC80-MRSA-IV [PVL+], European caMRSA Clone**													
Riyadh-2988048	NEG	NEG	NEG	NEG	NEG	NEG	NEG	NEG	NEG	POS	NEG	NEG	NEG
Riyadh-2990585-2	NEG	NEG	NEG	NEG	NEG	NEG	NEG	NEG	NEG	POS	NEG	NEG	NEG
Riyadh-2990585-1	NEG	NEG	NEG	NEG	NEG	NEG	NEG	NEG	NEG	POS	NEG	NEG	NEG
Riyadh-2826033	NEG	NEG	NEG	NEG	NEG	NEG	NEG	NEG	NEG	POS	NEG	NEG	NEG
Riyadh-1601562	NEG	NEG	NEG	NEG	NEG	NEG	NEG	NEG	NEG	POS	NEG	NEG	NEG
Riyadh-2569940	NEG	NEG	NEG	NEG	NEG	NEG	NEG	NEG	NEG	POS	NEG	NEG	NEG
Riyadh-2571692	NEG	NEG	NEG	NEG	NEG	NEG	NEG	NEG	NEG	POS	NEG	NEG	NEG
Riyadh-2763029	NEG	NEG	NEG	NEG	NEG	NEG	NEG	NEG	NEG	POS	NEG	NEG	NEG
Riyadh-2767090	NEG	NEG	NEG	NEG	NEG	NEG	NEG	NEG	NEG	POS	NEG	NEG	NEG
Riyadh-2775130	NEG	NEG	NEG	NEG	NEG	NEG	NEG	NEG	NEG	POS	NEG	NEG	NEG
Riyadh-2778256	NEG	NEG	NEG	NEG	NEG	NEG	NEG	NEG	NEG	POS	NEG	NEG	NEG
Riyadh-2817505	NEG	NEG	NEG	NEG	NEG	NEG	NEG	NEG	NEG	POS	NEG	NEG	NEG
Riyadh-3024912	NEG	NEG	NEG	NEG	NEG	NEG	NEG	NEG	NEG	POS	NEG	NEG	NEG
Riyadh-2788690	NEG	NEG	NEG	NEG	NEG	NEG	NEG	NEG	NEG	POS	NEG	NEG	NEG
Riyadh-2829034	NEG	NEG	NEG	NEG	NEG	NEG	NEG	NEG	NEG	POS	NEG	AMB	NEG
Riyadh-3	NEG	NEG	NEG	NEG	NEG	NEG	NEG	NEG	NEG	POS	NEG	NEG	NEG
Riyadh-5	NEG	NEG	NEG	NEG	NEG	NEG	NEG	NEG	NEG	POS	NEG	NEG	NEG
Riyadh-2553167	NEG	NEG	NEG	NEG	NEG	NEG	NEG	NEG	NEG	POS	NEG	NEG	NEG
Riyadh-3002592	NEG	NEG	NEG	NEG	NEG	NEG	NEG	NEG	NEG	POS	NEG	NEG	NEG
**CC88-MRSA-IV [PVL+]**													
Riyadh-2736996	NEG	NEG	NEG	NEG	NEG	NEG	NEG	NEG	NEG	POS	NEG	NEG	NEG
Riyadh-2942396	NEG	NEG	NEG	NEG	NEG	NEG	NEG	NEG	NEG	POS	NEG	NEG	NEG
Riyadh-3105391	NEG	NEG	NEG	NEG	NEG	NEG	NEG	NEG	NEG	POS	NEG	NEG	NEG
**CC97-MRSA-V**													
Riyadh-0297622	NEG	NEG	NEG	NEG	NEG	NEG	NEG	NEG	NEG	POS	NEG	NEG	NEG
Riyadh-3025471	NEG	NEG	NEG	NEG	NEG	NEG	NEG	NEG	NEG	POS	NEG	NEG	NEG

	VIRULENCE : TOX.SHOCK.TOXIN			VIRULENCE : ENTEROTOXINS									
	tst1 (consensus)	tst1 ("human" allele)	tst1 ("bovine" allele)	entA	entA (320E)	entA (N315) / entP	entB	entC	entD	entE	entG	entH	entI
	toxic shock syndrome toxin 1			enterotoxin A	enterotoxin A, allele from strain 320E	enterotoxin A, allele from strain N315 = entero-toxin P	enterotoxin B	enterotoxin C	enterotoxin D	enterotoxin E	enterotoxin G	enterotoxin H	enterotoxin I
**CC1-MRSA-IV&SCCfus, WA MRSA-1/45**													
Riyadh-3108609	NEG	NEG	NEG	POS	NEG	NEG	NEG	NEG	NEG	NEG	NEG	POS	NEG
**CC1/ST772-MRSA-V [PVL+], "Bengal Bay Clone/WA I**													
Riyadh-2819026	AMB	NEG	NEG	POS	AMB	NEG	AMB	POS	NEG	NEG	POS	NEG	POS
**CC5-MRSA-IV, Paediatric clone**													
Riyadh-2915327-1	NEG	NEG	NEG	NEG	NEG	NEG	NEG	NEG	NEG	NEG	POS	NEG	POS
Riyadh-2915327-2	NEG	NEG	NEG	NEG	NEG	NEG	NEG	NEG	NEG	NEG	POS	NEG	POS
Riyadh-2	NEG	NEG	NEG	NEG	NEG	NEG	POS	NEG	NEG	NEG	POS	NEG	POS
**CC5-MRSA-IV [PVL+], Paediatric clone**													
Riyadh-2986666	NEG	AMB	NEG	NEG	NEG	POS	NEG	NEG	NEG	NEG	POS	NEG	POS
Riyadh-2913335	NEG	AMB	NEG	NEG	NEG	POS	NEG	NEG	POS	NEG	POS	NEG	POS
**CC5-MRSA-IVvar, "Maltese Clone"**													
Riyadh-2983654	NEG	NEG	NEG	POS	NEG	NEG	NEG	NEG	NEG	NEG	POS	NEG	POS
Riyadh-4	NEG	NEG	NEG	POS	NEG	NEG	NEG	NEG	NEG	NEG	POS	NEG	POS
Riyadh-2790233	POS	POS	NEG	POS	NEG	NEG	NEG	POS	NEG	NEG	POS	NEG	POS
**CC5-MRSA-V**													
Riyadh-2568944	NEG	NEG	NEG	NEG	NEG	POS	NEG	NEG	POS	NEG	POS	NEG	POS
**CC6-MRSA-IV, WA MRSA-51/66**													
Riyadh-2556168	NEG	NEG	NEG	POS	NEG	NEG	NEG	NEG	NEG	NEG	NEG	NEG	NEG
Riyadh-2824507	NEG	NEG	NEG	POS	NEG	NEG	NEG	NEG	NEG	NEG	NEG	NEG	NEG
Riyadh-2990831	AMB	NEG	NEG	POS	AMB	NEG	NEG	NEG	NEG	NEG	NEG	NEG	NEG
**CC8/ST239-MRSA-III, Vienna/Hungarian/Brazilian Cl**													
Riyadh-9	NEG	NEG	NEG	POS	NEG	NEG	NEG	NEG	NEG	NEG	NEG	NEG	NEG
Riyadh-3028763	NEG	NEG	NEG	NEG	NEG	NEG	NEG	NEG	NEG	NEG	NEG	NEG	NEG
Riyadh-2817437	NEG	NEG	NEG	NEG	NEG	NEG	NEG	NEG	NEG	NEG	NEG	NEG	NEG
Riyadh-2793706	NEG	NEG	NEG	NEG	NEG	NEG	NEG	NEG	NEG	NEG	NEG	NEG	NEG
Riyadh-2818797	NEG	NEG	NEG	NEG	NEG	NEG	NEG	NEG	NEG	NEG	NEG	NEG	NEG
Riyadh-2822825	AMB	NEG	NEG	NEG	NEG	NEG	NEG	NEG	NEG	NEG	NEG	NEG	NEG
Riyadh-2888905	NEG	NEG	NEG	NEG	NEG	NEG	NEG	NEG	NEG	NEG	NEG	NEG	NEG
Riyadh-2888915	NEG	NEG	NEG	NEG	NEG	NEG	NEG	NEG	NEG	NEG	NEG	NEG	NEG
Riyadh-2567782	NEG	NEG	NEG	NEG	NEG	NEG	NEG	NEG	NEG	NEG	NEG	NEG	NEG
Riyadh-2891670	AMB	NEG	NEG	NEG	NEG	NEG	NEG	NEG	NEG	NEG	NEG	NEG	NEG
Riyadh-3006920	NEG	NEG	NEG	NEG	NEG	NEG	NEG	NEG	NEG	NEG	NEG	NEG	NEG
Riyadh-2817276-1	NEG	NEG	NEG	NEG	NEG	NEG	NEG	NEG	NEG	NEG	NEG	NEG	NEG
Riyadh-0295102	NEG	NEG	NEG	NEG	NEG	NEG	NEG	NEG	NEG	NEG	NEG	NEG	NEG
Riyadh-2820597	AMB	NEG	NEG	NEG	NEG	NEG	NEG	NEG	NEG	NEG	NEG	NEG	NEG
Riyadh-2822088	AMB	AMB	NEG	NEG	NEG	NEG	NEG	NEG	NEG	NEG	NEG	NEG	NEG
Riyadh-3010092	NEG	NEG	NEG	NEG	NEG	NEG	NEG	NEG	NEG	NEG	NEG	NEG	NEG
Riyadh-3022844	NEG	NEG	NEG	NEG	NEG	NEG	NEG	NEG	NEG	NEG	NEG	NEG	NEG
Riyadh-3108214-2	NEG	NEG	NEG	NEG	NEG	NEG	NEG	NEG	NEG	NEG	NEG	NEG	NEG
Riyadh-2823926	NEG	NEG	NEG	NEG	NEG	NEG	NEG	NEG	NEG	NEG	NEG	NEG	NEG
Riyadh-1	NEG	NEG	NEG	NEG	NEG	NEG	NEG	NEG	NEG	NEG	NEG	NEG	NEG
Riyadh-2818388	NEG	NEG	NEG	NEG	NEG	NEG	NEG	NEG	NEG	NEG	NEG	NEG	NEG
Riyadh-2818316	NEG	NEG	NEG	NEG	NEG	NEG	NEG	NEG	NEG	NEG	NEG	NEG	NEG
**CC9/ST834-MRSA-(atypical SCCmec)**													
Riyadh-3103521	POS	POS	NEG	NEG	NEG	NEG	NEG	POS	NEG	NEG	NEG	NEG	NEG
**CC22-MRSA-IV, Barnim/UK-EMRSA-15**													
Riyadh-2553359	POS	POS	NEG	NEG	NEG	NEG	NEG	NEG	NEG	NEG	POS	NEG	POS
Riyadh-2571758	POS	POS	NEG	NEG	NEG	NEG	NEG	NEG	NEG	NEG	POS	NEG	POS
Riyadh-3029203	POS	POS	NEG	NEG	NEG	NEG	NEG	NEG	NEG	NEG	POS	NEG	POS
Riyadh-3039785	POS	POS	NEG	NEG	NEG	NEG	NEG	NEG	NEG	NEG	POS	NEG	POS
Riyadh-3105594	POS	POS	NEG	NEG	NEG	NEG	NEG	NEG	NEG	NEG	POS	NEG	POS
Riyadh_IC_204-2	NEG	AMB	NEG	NEG	NEG	NEG	NEG	NEG	NEG	NEG	POS	NEG	POS
Riyadh-3003974	POS	POS	NEG	NEG	NEG	NEG	NEG	NEG	NEG	NEG	POS	NEG	POS
Riyadh_IC_067	NEG	NEG	NEG	NEG	NEG	NEG	NEG	NEG	NEG	NEG	POS	NEG	POS
Riyadh-2988627	NEG	NEG	NEG	NEG	NEG	NEG	NEG	NEG	NEG	NEG	POS	NEG	POS
Riyadh-3112581	NEG	NEG	NEG	NEG	NEG	NEG	NEG	NEG	NEG	NEG	POS	NEG	POS
**CC22-MRSA-IV [PVL+]**													
Riyadh-2781996-1	NEG	NEG	NEG	NEG	NEG	NEG	NEG	NEG	NEG	NEG	POS	NEG	POS
Riyadh-3103432	NEG	NEG	NEG	NEG	NEG	NEG	NEG	NEG	NEG	NEG	POS	NEG	POS
Riyadh-3026502	NEG	NEG	NEG	NEG	NEG	NEG	NEG	NEG	NEG	NEG	POS	NEG	POS
Riyadh-3081378-2	NEG	NEG	NEG	NEG	NEG	NEG	NEG	NEG	NEG	NEG	POS	NEG	POS
Riyadh_IC_185	NEG	AMB	NEG	NEG	NEG	NEG	NEG	NEG	NEG	NEG	POS	NEG	POS
Riyadh_IC_204-1	NEG	AMB	NEG	NEG	NEG	NEG	NEG	NEG	NEG	NEG	POS	NEG	POS
Riyadh-2559371	NEG	NEG	NEG	NEG	NEG	NEG	NEG	NEG	NEG	NEG	POS	NEG	POS
Riyadh-2753975	NEG	AMB	NEG	NEG	NEG	NEG	NEG	NEG	NEG	NEG	POS	NEG	POS
Riyadh-2775605	NEG	NEG	NEG	NEG	NEG	NEG	NEG	NEG	NEG	NEG	POS	NEG	POS
Riyadh-2781996-2	NEG	NEG	NEG	NEG	NEG	NEG	NEG	NEG	NEG	NEG	POS	NEG	POS
Riyadh-2823783-2	NEG	NEG	NEG	NEG	NEG	NEG	NEG	NEG	NEG	NEG	POS	NEG	POS
Riyadh-2876601	NEG	NEG	NEG	NEG	NEG	NEG	NEG	NEG	NEG	NEG	POS	NEG	POS
Riyadh-3036074	NEG	NEG	NEG	NEG	NEG	NEG	NEG	NEG	NEG	NEG	POS	NEG	POS
Riyadh-3053099	NEG	NEG	NEG	NEG	NEG	NEG	NEG	NEG	NEG	NEG	POS	NEG	POS
Riyadh-3055366	NEG	NEG	NEG	NEG	NEG	NEG	NEG	NEG	NEG	NEG	POS	NEG	POS
Riyadh-3082712	NEG	NEG	NEG	NEG	NEG	NEG	NEG	NEG	NEG	NEG	POS	NEG	POS
Riyadh-3087502	NEG	NEG	NEG	NEG	NEG	NEG	NEG	NEG	NEG	NEG	POS	NEG	POS
Riyadh-6	NEG	NEG	NEG	NEG	NEG	NEG	NEG	NEG	NEG	NEG	POS	NEG	POS
Riyadh-7	NEG	NEG	NEG	NEG	NEG	NEG	NEG	NEG	NEG	NEG	POS	NEG	POS
Riyadh-8	NEG	NEG	NEG	NEG	NEG	NEG	NEG	NEG	NEG	NEG	POS	NEG	POS
**CC30-MRSA-IV [PVL+], Southwest Pacific Clone**													
Riyadh-10	NEG	NEG	NEG	NEG	NEG	NEG	NEG	NEG	NEG	NEG	POS	NEG	POS
Riyadh_IC_123	NEG	NEG	NEG	NEG	NEG	NEG	NEG	NEG	NEG	NEG	POS	NEG	POS
Riyadh-3080713	NEG	NEG	NEG	NEG	NEG	NEG	NEG	NEG	NEG	NEG	POS	NEG	POS
Riyadh-2803856	NEG	NEG	NEG	NEG	NEG	NEG	NEG	NEG	NEG	NEG	POS	NEG	POS
Riyadh-2817276-1	AMB	NEG	NEG	NEG	NEG	NEG	NEG	NEG	NEG	NEG	POS	NEG	POS
Riyadh-2817571-2	NEG	NEG	NEG	NEG	NEG	NEG	NEG	NEG	NEG	NEG	POS	NEG	POS
Riyadh-3033868	NEG	NEG	NEG	NEG	NEG	NEG	NEG	NEG	NEG	NEG	POS	NEG	POS
Riyadh-2550108	NEG	NEG	NEG	NEG	NEG	NEG	NEG	NEG	NEG	NEG	POS	NEG	POS
Riyadh-3095056-2	NEG	NEG	NEG	NEG	NEG	NEG	NEG	NEG	NEG	NEG	POS	NEG	POS
Riyadh-2819899	NEG	NEG	NEG	NEG	NEG	NEG	NEG	NEG	NEG	NEG	POS	NEG	POS
Riyadh-2821805	NEG	NEG	NEG	NEG	NEG	NEG	NEG	NEG	NEG	NEG	POS	NEG	POS
Riyadh-3013928	NEG	NEG	NEG	NEG	NEG	NEG	NEG	NEG	NEG	NEG	POS	NEG	POS
Riyadh-3029402	NEG	NEG	NEG	NEG	NEG	NEG	NEG	NEG	NEG	NEG	POS	NEG	POS
**CC45/agr IV-MRSA-IV, WA MRSA-23**													
Riyadh-3081378-1	NEG	NEG	NEG	NEG	NEG	NEG	NEG	NEG	NEG	NEG	POS	NEG	POS
**CC80-MRSA-IV**													
Riyadh-3107635	NEG	NEG	NEG	NEG	NEG	NEG	NEG	NEG	NEG	NEG	NEG	NEG	NEG
Riyadh-2987458	NEG	AMB	NEG	NEG	NEG	NEG	POS	NEG	NEG	NEG	NEG	NEG	NEG
**CC80-MRSA-IV [PVL+], European caMRSA Clone**													
Riyadh-2988048	NEG	NEG	NEG	NEG	NEG	NEG	NEG	NEG	NEG	NEG	NEG	NEG	NEG
Riyadh-2990585-2	NEG	NEG	NEG	NEG	NEG	NEG	NEG	NEG	NEG	NEG	NEG	NEG	NEG
Riyadh-2990585-1	NEG	NEG	NEG	NEG	NEG	NEG	NEG	NEG	NEG	NEG	NEG	NEG	NEG
Riyadh-2826033	NEG	NEG	NEG	NEG	NEG	NEG	NEG	NEG	NEG	NEG	NEG	NEG	NEG
Riyadh-1601562	NEG	NEG	NEG	NEG	NEG	NEG	NEG	NEG	NEG	NEG	NEG	NEG	NEG
Riyadh-2569940	NEG	NEG	NEG	NEG	NEG	NEG	NEG	NEG	NEG	NEG	NEG	NEG	NEG
Riyadh-2571692	NEG	NEG	NEG	NEG	NEG	NEG	NEG	NEG	NEG	NEG	NEG	NEG	NEG
Riyadh-2763029	NEG	NEG	NEG	NEG	NEG	NEG	NEG	NEG	NEG	NEG	NEG	NEG	NEG
Riyadh-2767090	NEG	NEG	NEG	NEG	NEG	NEG	NEG	NEG	NEG	NEG	NEG	NEG	NEG
Riyadh-2775130	NEG	NEG	NEG	NEG	NEG	NEG	NEG	NEG	NEG	NEG	NEG	NEG	NEG
Riyadh-2778256	NEG	NEG	NEG	NEG	NEG	NEG	NEG	NEG	NEG	NEG	NEG	NEG	NEG
Riyadh-2817505	NEG	NEG	NEG	NEG	NEG	NEG	NEG	NEG	NEG	NEG	NEG	NEG	NEG
Riyadh-3024912	NEG	NEG	NEG	NEG	NEG	NEG	NEG	NEG	NEG	NEG	NEG	NEG	NEG
Riyadh-2788690	NEG	NEG	NEG	NEG	NEG	NEG	NEG	NEG	NEG	NEG	NEG	NEG	NEG
Riyadh-2829034	NEG	AMB	NEG	NEG	NEG	NEG	NEG	NEG	NEG	NEG	NEG	NEG	NEG
Riyadh-3	NEG	NEG	NEG	NEG	NEG	NEG	NEG	NEG	NEG	NEG	NEG	NEG	NEG
Riyadh-5	NEG	NEG	NEG	NEG	NEG	NEG	NEG	NEG	NEG	NEG	NEG	NEG	NEG
Riyadh-2553167	NEG	NEG	NEG	NEG	NEG	NEG	NEG	NEG	NEG	NEG	NEG	NEG	NEG
Riyadh-3002592	NEG	NEG	NEG	NEG	NEG	NEG	NEG	NEG	NEG	NEG	NEG	NEG	NEG
**CC88-MRSA-IV [PVL+]**													
Riyadh-2736996	NEG	NEG	NEG	NEG	NEG	NEG	NEG	NEG	NEG	NEG	NEG	NEG	NEG
Riyadh-2942396	AMB	NEG	NEG	NEG	NEG	POS	NEG	NEG	NEG	NEG	NEG	NEG	NEG
Riyadh-3105391	NEG	NEG	NEG	NEG	NEG	POS	NEG	NEG	NEG	NEG	NEG	NEG	NEG
**CC97-MRSA-V**													
Riyadh-0297622	NEG	NEG	NEG	NEG	NEG	NEG	NEG	NEG	NEG	NEG	NEG	NEG	NEG
Riyadh-3025471	NEG	NEG	NEG	NEG	NEG	NEG	NEG	NEG	NEG	NEG	NEG	NEG	NEG

	VIRULENCE : ENTEROTOXINS											
	entJ	entK	entL	entM	entN (cons)	entN (other than RF122)	entO	entQ	entR	entU	entCM14 probe1	entCM14 probe2
	enterotoxin J	enterotoxin K	enterotoxin L	enterotoxin M	enterotoxin N		enterotoxin O	enterotoxin Q	enterotoxin R	Enterotoxin U and/or Y	enterotoxin-like protein ORF CM14	
**CC1-MRSA-IV&SCCfus, WA MRSA-1/45**												
Riyadh-3108609	NEG	POS	NEG	NEG	NEG	NEG	NEG	POS	NEG	NEG	NEG	NEG
**CC1/ST772-MRSA-V [PVL+], "Bengal Bay Clone/WA I**												
Riyadh-2819026	NEG	NEG	POS	POS	POS	POS	POS	NEG	NEG	POS	POS	POS
**CC5-MRSA-IV, Paediatric clone**												
Riyadh-2915327-1	NEG	NEG	NEG	POS	POS	POS	POS	NEG	NEG	POS	NEG	NEG
Riyadh-2915327-2	NEG	NEG	NEG	POS	POS	POS	POS	NEG	NEG	POS	NEG	NEG
Riyadh-2	NEG	NEG	NEG	POS	POS	POS	POS	NEG	NEG	POS	NEG	NEG
**CC5-MRSA-IV [PVL+], Paediatric clone**												
Riyadh-2986666	NEG	NEG	NEG	POS	POS	POS	POS	NEG	NEG	POS	NEG	NEG
Riyadh-2913335	POS	NEG	NEG	POS	POS	POS	POS	NEG	POS	POS	NEG	NEG
**CC5-MRSA-IVvar, "Maltese Clone"**												
Riyadh-2983654	NEG	NEG	NEG	POS	POS	POS	POS	NEG	NEG	POS	NEG	NEG
Riyadh-4	NEG	NEG	NEG	POS	POS	POS	POS	NEG	NEG	POS	NEG	NEG
Riyadh-2790233	NEG	NEG	POS	POS	POS	POS	POS	NEG	NEG	POS	NEG	NEG
**CC5-MRSA-V**												
Riyadh-2568944	POS	NEG	NEG	POS	POS	POS	POS	NEG	POS	POS	NEG	NEG
**CC6-MRSA-IV, WA MRSA-51/66**												
Riyadh-2556168	NEG	NEG	NEG	NEG	NEG	NEG	NEG	NEG	NEG	NEG	NEG	NEG
Riyadh-2824507	NEG	NEG	NEG	NEG	NEG	NEG	NEG	NEG	NEG	NEG	NEG	NEG
Riyadh-2990831	NEG	NEG	NEG	NEG	NEG	NEG	NEG	NEG	NEG	NEG	NEG	NEG
**CC8/ST239-MRSA-III, Vienna/Hungarian/Brazilian Cl**												
Riyadh-9	NEG	POS	NEG	NEG	NEG	NEG	NEG	POS	NEG	NEG	NEG	NEG
Riyadh-3028763	NEG	POS	NEG	NEG	NEG	NEG	NEG	POS	NEG	NEG	NEG	NEG
Riyadh-2817437	NEG	POS	NEG	NEG	NEG	NEG	NEG	POS	NEG	NEG	NEG	NEG
Riyadh-2793706	NEG	POS	NEG	NEG	NEG	NEG	NEG	POS	NEG	NEG	NEG	NEG
Riyadh-2818797	NEG	POS	NEG	NEG	NEG	NEG	NEG	POS	NEG	NEG	NEG	NEG
Riyadh-2822825	NEG	POS	NEG	NEG	NEG	NEG	NEG	POS	NEG	NEG	NEG	NEG
Riyadh-2888905	NEG	POS	NEG	NEG	NEG	NEG	NEG	POS	NEG	NEG	NEG	NEG
Riyadh-2888915	NEG	POS	NEG	NEG	NEG	NEG	NEG	POS	NEG	NEG	NEG	NEG
Riyadh-2567782	NEG	POS	NEG	NEG	NEG	NEG	NEG	POS	NEG	NEG	NEG	NEG
Riyadh-2891670	NEG	POS	NEG	NEG	NEG	NEG	NEG	POS	NEG	NEG	NEG	NEG
Riyadh-3006920	NEG	POS	NEG	NEG	NEG	NEG	NEG	POS	NEG	NEG	NEG	NEG
Riyadh-2817276-1	NEG	POS	NEG	NEG	NEG	NEG	NEG	POS	NEG	NEG	NEG	NEG
Riyadh-0295102	NEG	POS	NEG	NEG	NEG	NEG	NEG	POS	NEG	NEG	NEG	NEG
Riyadh-2820597	NEG	POS	NEG	NEG	NEG	NEG	NEG	POS	NEG	NEG	NEG	NEG
Riyadh-2822088	NEG	POS	NEG	NEG	NEG	NEG	NEG	POS	NEG	NEG	NEG	NEG
Riyadh-3010092	NEG	POS	NEG	NEG	NEG	NEG	NEG	POS	NEG	NEG	NEG	NEG
Riyadh-3022844	NEG	POS	NEG	NEG	NEG	NEG	NEG	POS	NEG	NEG	NEG	NEG
Riyadh-3108214-2	NEG	POS	NEG	NEG	NEG	NEG	NEG	POS	NEG	NEG	NEG	NEG
Riyadh-2823926	NEG	POS	NEG	NEG	NEG	NEG	NEG	POS	NEG	NEG	NEG	NEG
Riyadh-1	NEG	POS	NEG	NEG	NEG	NEG	NEG	POS	NEG	NEG	NEG	NEG
Riyadh-2818388	NEG	POS	NEG	NEG	NEG	NEG	NEG	POS	NEG	NEG	NEG	NEG
Riyadh-2818316	NEG	POS	NEG	NEG	NEG	NEG	NEG	POS	NEG	NEG	NEG	NEG
**CC9/ST834-MRSA-(atypical SCCmec)**												
Riyadh-3103521	NEG	NEG	POS	NEG	NEG	NEG	NEG	NEG	NEG	NEG	NEG	NEG
**CC22-MRSA-IV, Barnim/UK-EMRSA-15**												
Riyadh-2553359	NEG	NEG	NEG	POS	POS	POS	POS	NEG	NEG	POS	NEG	NEG
Riyadh-2571758	NEG	NEG	NEG	POS	POS	POS	POS	NEG	NEG	POS	NEG	NEG
Riyadh-3029203	NEG	NEG	NEG	POS	POS	POS	POS	NEG	NEG	POS	NEG	NEG
Riyadh-3039785	NEG	NEG	NEG	POS	POS	POS	POS	NEG	NEG	POS	NEG	NEG
Riyadh-3105594	NEG	NEG	NEG	POS	POS	POS	POS	NEG	NEG	POS	NEG	NEG
Riyadh_IC_204-2	NEG	NEG	NEG	POS	POS	POS	POS	NEG	NEG	POS	NEG	NEG
Riyadh-3003974	NEG	NEG	NEG	POS	POS	POS	POS	NEG	NEG	POS	NEG	NEG
Riyadh_IC_067	NEG	NEG	NEG	POS	POS	POS	POS	NEG	NEG	POS	NEG	NEG
Riyadh-2988627	NEG	NEG	NEG	POS	POS	POS	POS	NEG	NEG	POS	NEG	NEG
Riyadh-3112581	NEG	NEG	NEG	POS	POS	POS	POS	NEG	NEG	POS	NEG	NEG
**CC22-MRSA-IV [PVL+]**												
Riyadh-2781996-1	NEG	NEG	NEG	POS	POS	POS	POS	NEG	NEG	POS	NEG	NEG
Riyadh-3103432	NEG	NEG	NEG	POS	POS	POS	POS	NEG	NEG	POS	NEG	NEG
Riyadh-3026502	NEG	NEG	NEG	POS	POS	POS	POS	NEG	NEG	POS	NEG	NEG
Riyadh-3081378-2	NEG	NEG	NEG	POS	POS	POS	POS	NEG	NEG	POS	NEG	NEG
Riyadh_IC_185	NEG	NEG	NEG	POS	POS	POS	POS	NEG	NEG	POS	NEG	NEG
Riyadh_IC_204-1	NEG	NEG	NEG	POS	POS	POS	POS	NEG	NEG	POS	NEG	NEG
Riyadh-2559371	NEG	NEG	NEG	POS	POS	POS	POS	NEG	NEG	POS	NEG	NEG
Riyadh-2753975	NEG	NEG	NEG	POS	POS	POS	POS	NEG	NEG	POS	NEG	NEG
Riyadh-2775605	NEG	NEG	NEG	POS	POS	POS	POS	NEG	NEG	POS	NEG	NEG
Riyadh-2781996-2	NEG	NEG	NEG	POS	POS	POS	POS	NEG	NEG	POS	NEG	NEG
Riyadh-2823783-2	NEG	NEG	NEG	POS	POS	POS	POS	NEG	NEG	POS	NEG	NEG
Riyadh-2876601	NEG	NEG	NEG	POS	POS	POS	POS	NEG	NEG	POS	NEG	NEG
Riyadh-3036074	NEG	NEG	NEG	POS	POS	POS	POS	NEG	NEG	POS	NEG	NEG
Riyadh-3053099	NEG	NEG	NEG	POS	POS	POS	POS	NEG	NEG	POS	NEG	NEG
Riyadh-3055366	NEG	NEG	NEG	POS	POS	POS	POS	NEG	NEG	POS	NEG	NEG
Riyadh-3082712	NEG	NEG	NEG	POS	POS	POS	POS	NEG	NEG	POS	NEG	NEG
Riyadh-3087502	NEG	NEG	NEG	POS	POS	POS	POS	NEG	NEG	POS	NEG	NEG
Riyadh-6	NEG	NEG	NEG	POS	POS	POS	POS	NEG	NEG	POS	NEG	NEG
Riyadh-7	NEG	NEG	NEG	POS	POS	POS	POS	NEG	NEG	POS	NEG	NEG
Riyadh-8	NEG	NEG	NEG	POS	POS	POS	POS	NEG	NEG	POS	NEG	NEG
**CC30-MRSA-IV [PVL+], Southwest Pacific Clone**												
Riyadh-10	NEG	NEG	NEG	POS	POS	POS	POS	NEG	NEG	POS	NEG	NEG
Riyadh_IC_123	NEG	NEG	NEG	POS	POS	POS	POS	NEG	NEG	POS	NEG	NEG
Riyadh-3080713	NEG	NEG	NEG	POS	POS	POS	POS	NEG	NEG	POS	NEG	NEG
Riyadh-2803856	NEG	NEG	NEG	POS	POS	POS	POS	NEG	NEG	POS	NEG	NEG
Riyadh-2817276-1	NEG	NEG	NEG	POS	POS	POS	POS	NEG	NEG	POS	NEG	NEG
Riyadh-2817571-2	NEG	NEG	NEG	POS	POS	POS	POS	NEG	NEG	POS	NEG	NEG
Riyadh-3033868	NEG	NEG	NEG	POS	POS	POS	POS	NEG	NEG	POS	NEG	NEG
Riyadh-2550108	NEG	NEG	NEG	POS	POS	POS	POS	NEG	NEG	POS	NEG	NEG
Riyadh-3095056-2	NEG	NEG	NEG	POS	POS	POS	POS	NEG	NEG	POS	NEG	NEG
Riyadh-2819899	NEG	NEG	NEG	POS	POS	POS	POS	NEG	NEG	POS	NEG	NEG
Riyadh-2821805	NEG	NEG	NEG	POS	POS	POS	POS	NEG	NEG	POS	NEG	NEG
Riyadh-3013928	NEG	NEG	NEG	POS	POS	POS	POS	NEG	NEG	POS	NEG	NEG
Riyadh-3029402	NEG	NEG	NEG	POS	POS	POS	POS	NEG	NEG	POS	NEG	NEG
**CC45/agr IV-MRSA-IV, WA MRSA-23**												
Riyadh-3081378-1	POS	NEG	NEG	POS	POS	POS	POS	NEG	POS	POS	NEG	NEG
**CC80-MRSA-IV**												
Riyadh-3107635	NEG	NEG	NEG	NEG	NEG	NEG	NEG	NEG	NEG	NEG	NEG	NEG
Riyadh-2987458	NEG	POS	NEG	NEG	NEG	NEG	NEG	POS	NEG	NEG	NEG	NEG
**CC80-MRSA-IV [PVL+], European caMRSA Clone**												
Riyadh-2988048	NEG	NEG	NEG	NEG	NEG	NEG	NEG	NEG	NEG	NEG	NEG	NEG
Riyadh-2990585-2	NEG	NEG	NEG	NEG	NEG	NEG	NEG	NEG	NEG	NEG	NEG	NEG
Riyadh-2990585-1	NEG	NEG	NEG	NEG	NEG	NEG	NEG	NEG	NEG	NEG	NEG	NEG
Riyadh-2826033	NEG	NEG	NEG	NEG	NEG	NEG	NEG	NEG	NEG	NEG	NEG	NEG
Riyadh-1601562	NEG	NEG	NEG	NEG	NEG	NEG	NEG	NEG	NEG	NEG	NEG	NEG
Riyadh-2569940	NEG	NEG	NEG	NEG	NEG	NEG	NEG	NEG	NEG	NEG	NEG	NEG
Riyadh-2571692	NEG	NEG	NEG	NEG	NEG	NEG	NEG	NEG	NEG	NEG	NEG	NEG
Riyadh-2763029	NEG	NEG	NEG	NEG	NEG	NEG	NEG	NEG	NEG	NEG	NEG	NEG
Riyadh-2767090	NEG	NEG	NEG	NEG	NEG	NEG	NEG	NEG	NEG	NEG	NEG	NEG
Riyadh-2775130	NEG	NEG	NEG	NEG	NEG	NEG	NEG	NEG	NEG	NEG	NEG	NEG
Riyadh-2778256	NEG	NEG	NEG	NEG	NEG	NEG	NEG	NEG	NEG	NEG	NEG	NEG
Riyadh-2817505	NEG	NEG	NEG	NEG	NEG	NEG	NEG	NEG	NEG	NEG	NEG	NEG
Riyadh-3024912	NEG	NEG	NEG	NEG	NEG	NEG	NEG	NEG	NEG	NEG	NEG	NEG
Riyadh-2788690	NEG	NEG	NEG	NEG	NEG	NEG	NEG	NEG	NEG	NEG	NEG	NEG
Riyadh-2829034	NEG	NEG	NEG	NEG	NEG	NEG	NEG	NEG	NEG	NEG	NEG	NEG
Riyadh-3	NEG	NEG	NEG	NEG	NEG	NEG	NEG	NEG	NEG	NEG	NEG	NEG
Riyadh-5	NEG	NEG	NEG	NEG	NEG	NEG	NEG	NEG	NEG	NEG	NEG	NEG
Riyadh-2553167	NEG	NEG	NEG	NEG	NEG	NEG	NEG	NEG	NEG	NEG	NEG	NEG
Riyadh-3002592	NEG	NEG	NEG	NEG	NEG	NEG	NEG	NEG	NEG	NEG	NEG	NEG
**CC88-MRSA-IV [PVL+]**												
Riyadh-2736996	NEG	NEG	NEG	NEG	NEG	NEG	NEG	NEG	NEG	NEG	NEG	NEG
Riyadh-2942396	NEG	NEG	NEG	NEG	NEG	NEG	NEG	NEG	NEG	NEG	NEG	NEG
Riyadh-3105391	NEG	NEG	NEG	NEG	NEG	NEG	NEG	NEG	NEG	NEG	NEG	NEG
**CC97-MRSA-V**												
Riyadh-0297622	NEG	NEG	NEG	NEG	NEG	NEG	NEG	NEG	NEG	NEG	NEG	NEG
Riyadh-3025471	NEG	NEG	NEG	NEG	NEG	NEG	NEG	NEG	NEG	NEG	NEG	NEG

	VIRULENCE : HLG AND LEUKOCIDINS												
	lukF	lukS	lukS (ST22+ST45)	hlgA	lukF-PV	lukS-PV	lukF-PV (P83)	lukM	lukD	lukE	lukX	lukY	lukY (ST30+ST45)
	haemolysin gamma / leukocidin, component B	haemolysin gamma / leukocidin, component C		haemolysin gamma, component A	Panton Valentine leukocidin F component	Panton Valentine leukocidin S component	F component from hypothetical leukocidin from ruminants	S component from hypothetical leukocidin from ruminants	leukocidin D component	leukocidin E component	leukocidin/ haemolysin toxin family protein	leukocidin/haemolysin toxin family protein	
**CC1-MRSA-IV&SCCfus, WA MRSA-1/45**													
Riyadh-3108609	POS	POS	POS	POS	NEG	NEG	NEG	NEG	POS	POS	POS	POS	NEG
**CC1/ST772-MRSA-V [PVL+], "Bengal Bay Clone/WA I**													
Riyadh-2819026	POS	POS	POS	POS	POS	POS	NEG	NEG	NEG	NEG	POS	POS	NEG
**CC5-MRSA-IV, Paediatric clone**													
Riyadh-2915327-1	POS	POS	POS	POS	NEG	NEG	NEG	NEG	POS	POS	POS	POS	NEG
Riyadh-2915327-2	POS	POS	POS	POS	NEG	NEG	NEG	NEG	POS	POS	POS	POS	NEG
Riyadh-2	POS	POS	POS	POS	NEG	NEG	NEG	NEG	POS	POS	POS	POS	NEG
**CC5-MRSA-IV [PVL+], Paediatric clone**													
Riyadh-2986666	POS	POS	POS	POS	POS	POS	NEG	NEG	POS	POS	POS	POS	NEG
Riyadh-2913335	POS	POS	POS	POS	POS	POS	NEG	NEG	POS	POS	POS	POS	NEG
**CC5-MRSA-IVvar, "Maltese Clone"**													
Riyadh-2983654	POS	POS	POS	POS	NEG	NEG	NEG	NEG	POS	POS	POS	POS	NEG
Riyadh-4	POS	POS	POS	POS	NEG	NEG	NEG	NEG	POS	POS	POS	POS	NEG
Riyadh-2790233	POS	POS	POS	POS	NEG	NEG	NEG	NEG	POS	POS	POS	POS	NEG
**CC5-MRSA-V**													
Riyadh-2568944	POS	POS	POS	POS	NEG	NEG	NEG	NEG	POS	POS	POS	POS	NEG
**CC6-MRSA-IV, WA MRSA-51/66**													
Riyadh-2556168	POS	POS	POS	POS	NEG	NEG	NEG	NEG	POS	POS	POS	POS	NEG
Riyadh-2824507	POS	POS	POS	POS	NEG	NEG	NEG	NEG	POS	POS	POS	POS	NEG
Riyadh-2990831	POS	POS	POS	POS	NEG	NEG	NEG	NEG	POS	POS	POS	POS	NEG
**CC8/ST239-MRSA-III, Vienna/Hungarian/Brazilian Cl**													
Riyadh-9	POS	POS	POS	POS	NEG	NEG	NEG	NEG	POS	POS	POS	POS	NEG
Riyadh-3028763	POS	POS	POS	POS	NEG	NEG	NEG	NEG	POS	POS	POS	POS	NEG
Riyadh-2817437	POS	POS	POS	POS	NEG	NEG	NEG	NEG	POS	POS	POS	POS	NEG
Riyadh-2793706	POS	POS	POS	POS	NEG	NEG	NEG	NEG	POS	POS	POS	POS	NEG
Riyadh-2818797	POS	POS	POS	POS	NEG	NEG	NEG	NEG	POS	POS	POS	POS	NEG
Riyadh-2822825	POS	POS	POS	POS	NEG	NEG	NEG	NEG	POS	POS	POS	POS	NEG
Riyadh-2888905	POS	POS	POS	POS	NEG	NEG	NEG	NEG	POS	POS	POS	POS	NEG
Riyadh-2888915	POS	POS	POS	POS	NEG	NEG	NEG	NEG	POS	POS	POS	POS	NEG
Riyadh-2567782	POS	POS	POS	POS	NEG	NEG	NEG	NEG	POS	POS	POS	POS	NEG
Riyadh-2891670	POS	POS	POS	POS	NEG	NEG	NEG	NEG	POS	POS	POS	POS	NEG
Riyadh-3006920	POS	POS	POS	POS	NEG	NEG	NEG	NEG	POS	POS	POS	POS	NEG
Riyadh-2817276-1	POS	POS	POS	POS	NEG	NEG	NEG	NEG	POS	POS	POS	POS	NEG
Riyadh-0295102	POS	POS	POS	POS	NEG	NEG	NEG	NEG	POS	POS	POS	POS	NEG
Riyadh-2820597	POS	POS	POS	POS	NEG	NEG	NEG	NEG	POS	POS	POS	POS	NEG
Riyadh-2822088	POS	POS	POS	POS	NEG	NEG	NEG	NEG	POS	POS	POS	POS	NEG
Riyadh-3010092	POS	POS	POS	POS	NEG	NEG	NEG	NEG	POS	POS	POS	POS	NEG
Riyadh-3022844	POS	POS	POS	POS	NEG	NEG	NEG	NEG	POS	POS	POS	POS	NEG
Riyadh-3108214-2	POS	POS	AMB	POS	NEG	NEG	NEG	NEG	POS	POS	POS	POS	NEG
Riyadh-2823926	POS	POS	POS	POS	NEG	NEG	NEG	NEG	POS	POS	POS	POS	NEG
Riyadh-1	POS	POS	POS	POS	NEG	NEG	NEG	NEG	POS	POS	POS	POS	NEG
Riyadh-2818388	POS	POS	POS	POS	NEG	NEG	NEG	NEG	POS	POS	POS	POS	NEG
Riyadh-2818316	POS	POS	POS	POS	NEG	NEG	NEG	NEG	POS	POS	POS	POS	NEG
**CC9/ST834-MRSA-(atypical SCCmec)**													
Riyadh-3103521	POS	POS	POS	POS	NEG	NEG	NEG	NEG	POS	POS	POS	POS	NEG
**CC22-MRSA-IV, Barnim/UK-EMRSA-15**													
Riyadh-2553359	POS	POS	POS	POS	NEG	NEG	NEG	NEG	NEG	NEG	POS	POS	NEG
Riyadh-2571758	POS	POS	POS	POS	NEG	NEG	NEG	NEG	NEG	NEG	POS	POS	NEG
Riyadh-3029203	POS	POS	POS	POS	NEG	NEG	NEG	NEG	NEG	NEG	POS	POS	NEG
Riyadh-3039785	POS	POS	POS	POS	NEG	NEG	NEG	NEG	NEG	NEG	POS	POS	NEG
Riyadh-3105594	POS	POS	POS	POS	NEG	NEG	NEG	NEG	NEG	NEG	POS	POS	NEG
Riyadh_IC_204-2	POS	POS	POS	POS	NEG	NEG	NEG	NEG	NEG	NEG	POS	POS	NEG
Riyadh-3003974	POS	POS	POS	POS	NEG	NEG	NEG	NEG	NEG	NEG	POS	POS	NEG
Riyadh_IC_067	POS	POS	POS	POS	NEG	NEG	NEG	NEG	NEG	NEG	POS	POS	NEG
Riyadh-2988627	POS	POS	POS	POS	NEG	NEG	NEG	NEG	NEG	NEG	POS	POS	NEG
Riyadh-3112581	POS	AMB	POS	POS	NEG	NEG	NEG	NEG	NEG	NEG	POS	POS	NEG
**CC22-MRSA-IV [PVL+]**													
Riyadh-2781996-1	POS	AMB	POS	POS	POS	POS	NEG	NEG	NEG	NEG	POS	POS	NEG
Riyadh-3103432	POS	POS	POS	POS	POS	POS	NEG	NEG	NEG	NEG	POS	POS	NEG
Riyadh-3026502	NEG	NEG	POS	POS	POS	POS	NEG	NEG	NEG	NEG	POS	POS	NEG
Riyadh-3081378-2	AMB	AMB	POS	POS	POS	POS	NEG	NEG	NEG	NEG	POS	POS	NEG
Riyadh_IC_185	POS	POS	POS	POS	POS	POS	NEG	NEG	NEG	NEG	POS	POS	NEG
Riyadh_IC_204-1	POS	POS	POS	POS	POS	POS	NEG	NEG	NEG	NEG	POS	POS	NEG
Riyadh-2559371	POS	POS	POS	POS	POS	POS	NEG	NEG	NEG	NEG	POS	POS	NEG
Riyadh-2753975	POS	POS	POS	POS	POS	POS	NEG	NEG	NEG	NEG	POS	POS	NEG
Riyadh-2775605	POS	POS	POS	POS	POS	POS	NEG	NEG	NEG	NEG	POS	POS	NEG
Riyadh-2781996-2	POS	AMB	POS	POS	POS	POS	NEG	NEG	NEG	NEG	POS	POS	NEG
Riyadh-2823783-2	POS	POS	POS	POS	POS	POS	NEG	NEG	NEG	NEG	POS	POS	NEG
Riyadh-2876601	POS	POS	POS	POS	POS	POS	NEG	NEG	NEG	NEG	POS	POS	NEG
Riyadh-3036074	POS	POS	POS	POS	POS	POS	NEG	NEG	NEG	NEG	POS	POS	NEG
Riyadh-3053099	POS	POS	POS	POS	POS	POS	NEG	NEG	NEG	NEG	POS	POS	NEG
Riyadh-3055366	POS	POS	POS	POS	POS	POS	NEG	NEG	NEG	NEG	POS	POS	NEG
Riyadh-3082712	POS	POS	POS	POS	POS	POS	NEG	NEG	NEG	NEG	POS	POS	NEG
Riyadh-3087502	NEG	NEG	POS	AMB	POS	POS	NEG	NEG	NEG	NEG	AMB	POS	NEG
Riyadh-6	POS	POS	POS	POS	POS	POS	NEG	NEG	NEG	NEG	POS	POS	NEG
Riyadh-7	POS	POS	POS	POS	POS	POS	NEG	NEG	NEG	NEG	POS	POS	NEG
Riyadh-8	POS	POS	POS	POS	POS	POS	NEG	NEG	NEG	NEG	POS	POS	NEG
**CC30-MRSA-IV [PVL+], Southwest Pacific Clone**													
Riyadh-10	POS	POS	POS	POS	POS	POS	NEG	NEG	NEG	NEG	POS	NEG	POS
Riyadh_IC_123	POS	POS	POS	POS	POS	POS	NEG	NEG	NEG	NEG	POS	NEG	POS
Riyadh-3080713	POS	POS	POS	POS	POS	POS	NEG	NEG	NEG	NEG	POS	NEG	POS
Riyadh-2803856	POS	POS	POS	POS	POS	POS	NEG	NEG	NEG	NEG	POS	NEG	POS
Riyadh-2817276-1	POS	POS	POS	POS	POS	POS	NEG	NEG	NEG	NEG	POS	NEG	POS
Riyadh-2817571-2	POS	POS	POS	POS	POS	POS	NEG	NEG	NEG	NEG	POS	NEG	POS
Riyadh-3033868	POS	POS	POS	POS	POS	POS	NEG	NEG	NEG	NEG	POS	NEG	POS
Riyadh-2550108	POS	POS	POS	POS	POS	POS	NEG	NEG	NEG	NEG	POS	NEG	POS
Riyadh-3095056-2	POS	POS	POS	POS	POS	POS	NEG	NEG	NEG	NEG	POS	NEG	POS
Riyadh-2819899	POS	POS	POS	POS	POS	POS	NEG	NEG	NEG	NEG	POS	NEG	POS
Riyadh-2821805	POS	POS	POS	POS	POS	POS	NEG	NEG	NEG	NEG	POS	NEG	POS
Riyadh-3013928	POS	POS	POS	POS	POS	POS	NEG	NEG	NEG	NEG	POS	NEG	POS
Riyadh-3029402	POS	POS	POS	POS	POS	POS	NEG	NEG	NEG	NEG	POS	NEG	POS
**CC45/agr IV-MRSA-IV, WA MRSA-23**													
Riyadh-3081378-1	POS	NEG	POS	POS	NEG	NEG	NEG	NEG	NEG	NEG	POS	NEG	POS
**CC80-MRSA-IV**													
Riyadh-3107635	POS	POS	POS	POS	NEG	NEG	NEG	NEG	POS	POS	POS	POS	NEG
Riyadh-2987458	POS	POS	POS	POS	NEG	NEG	NEG	NEG	POS	POS	POS	POS	NEG
**CC80-MRSA-IV [PVL+], European caMRSA Clone**													
Riyadh-2988048	POS	POS	AMB	POS	POS	POS	NEG	NEG	POS	POS	POS	POS	NEG
Riyadh-2990585-2	POS	POS	AMB	POS	POS	POS	NEG	NEG	POS	POS	POS	POS	NEG
Riyadh-2990585-1	POS	POS	POS	POS	POS	POS	NEG	NEG	POS	POS	POS	POS	NEG
Riyadh-2826033	POS	POS	POS	POS	POS	POS	NEG	NEG	POS	POS	POS	POS	NEG
Riyadh-1601562	POS	POS	POS	POS	POS	POS	NEG	NEG	POS	POS	POS	POS	NEG
Riyadh-2569940	POS	POS	POS	POS	POS	POS	NEG	NEG	POS	POS	POS	POS	NEG
Riyadh-2571692	POS	POS	POS	POS	POS	POS	NEG	NEG	POS	POS	POS	POS	NEG
Riyadh-2763029	POS	POS	POS	POS	POS	POS	NEG	NEG	POS	POS	POS	POS	NEG
Riyadh-2767090	POS	POS	POS	POS	POS	POS	NEG	NEG	POS	POS	POS	POS	NEG
Riyadh-2775130	POS	POS	AMB	POS	POS	POS	NEG	NEG	POS	POS	POS	POS	NEG
Riyadh-2778256	POS	POS	POS	POS	POS	POS	NEG	NEG	POS	POS	POS	POS	NEG
Riyadh-2817505	POS	POS	POS	POS	POS	POS	NEG	NEG	POS	POS	POS	POS	NEG
Riyadh-3024912	POS	POS	AMB	POS	POS	POS	NEG	NEG	POS	POS	POS	POS	NEG
Riyadh-2788690	POS	POS	AMB	POS	POS	POS	NEG	NEG	POS	POS	POS	POS	NEG
Riyadh-2829034	POS	POS	POS	POS	POS	POS	NEG	NEG	POS	POS	POS	POS	NEG
Riyadh-3	POS	POS	POS	POS	POS	POS	NEG	NEG	POS	POS	POS	POS	NEG
Riyadh-5	POS	POS	POS	POS	POS	POS	NEG	NEG	POS	POS	POS	POS	NEG
Riyadh-2553167	POS	POS	POS	POS	POS	POS	NEG	NEG	POS	POS	POS	POS	NEG
Riyadh-3002592	POS	POS	POS	POS	POS	POS	NEG	NEG	POS	POS	POS	POS	NEG
**CC88-MRSA-IV [PVL+]**													
Riyadh-2736996	POS	POS	POS	POS	POS	POS	NEG	NEG	POS	POS	POS	POS	NEG
Riyadh-2942396	POS	POS	POS	POS	POS	POS	NEG	NEG	POS	POS	POS	POS	NEG
Riyadh-3105391	POS	POS	POS	POS	POS	POS	NEG	NEG	POS	POS	POS	POS	NEG
**CC97-MRSA-V**													
Riyadh-0297622	POS	POS	POS	POS	NEG	NEG	NEG	NEG	POS	POS	POS	POS	NEG
Riyadh-3025471	POS	POS	POS	POS	NEG	NEG	NEG	NEG	POS	POS	POS	POS	NEG

	VIRULENCE : HAEMOLYSINS								VIRULENCE : HLB-CONV PHAGES		
	hl	hla	hlIII (cons)	hlIII (other than RF122)	hlb-probe 1	hlb-probe 2	hlb-probe 3	un-truncated hlb	sak	chp	scn
	putative membrane protein	haemolysin alpha	putative membrane protein		haemolysin beta				staphylo-kinase	chemotaxis-inhibiting protein (CHIPS)	Staphyl. Comple-ment inhibitor
**CC1-MRSA-IV&SCCfus, WA MRSA-1/45**											
Riyadh-3108609	POS	POS	POS	POS	POS	POS	POS	AMB	POS	NEG	POS
**CC1/ST772-MRSA-V [PVL+], "Bengal Bay Clone/WA I**											
Riyadh-2819026	POS	POS	POS	POS	AMB	NEG	POS	POS	NEG	AMB	POS
**CC5-MRSA-IV, Paediatric clone**											
Riyadh-2915327-1	POS	POS	POS	POS	POS	POS	POS	NEG	POS	POS	POS
Riyadh-2915327-2	POS	POS	POS	POS	POS	POS	AMB	POS	NEG	NEG	AMB
Riyadh-2	POS	POS	POS	POS	POS	POS	POS	NEG	POS	POS	POS
**CC5-MRSA-IV [PVL+], Paediatric clone**											
Riyadh-2986666	POS	POS	POS	POS	POS	POS	POS	AMB	POS	AMB	POS
Riyadh-2913335	POS	POS	POS	POS	POS	POS	POS	AMB	POS	NEG	POS
**CC5-MRSA-IVvar, "Maltese Clone"**											
Riyadh-2983654	POS	POS	POS	POS	POS	POS	POS	AMB	POS	NEG	POS
Riyadh-4	POS	POS	POS	POS	POS	POS	POS	NEG	POS	NEG	POS
Riyadh-2790233	POS	POS	POS	POS	POS	POS	POS	NEG	POS	NEG	POS
**CC5-MRSA-V**											
Riyadh-2568944	POS	POS	POS	POS	POS	POS	POS	NEG	POS	AMB	POS
**CC6-MRSA-IV, WA MRSA-51/66**											
Riyadh-2556168	POS	POS	POS	POS	POS	POS	POS	NEG	POS	AMB	POS
Riyadh-2824507	POS	POS	POS	POS	POS	POS	POS	NEG	POS	NEG	POS
Riyadh-2990831	POS	POS	POS	POS	POS	POS	POS	POS	POS	AMB	POS
**CC8/ST239-MRSA-III, Vienna/Hungarian/Brazilian Cl**											
Riyadh-9	POS	NEG	POS	POS	POS	POS	POS	AMB	POS	NEG	POS
Riyadh-3028763	POS	POS	POS	POS	POS	POS	POS	AMB	POS	POS	POS
Riyadh-2817437	POS	POS	POS	POS	POS	POS	POS	POS	POS	POS	POS
Riyadh-2793706	POS	POS	POS	POS	POS	POS	POS	AMB	POS	POS	POS
Riyadh-2818797	POS	POS	POS	POS	POS	POS	POS	NEG	POS	POS	POS
Riyadh-2822825	POS	POS	POS	POS	POS	POS	POS	POS	POS	POS	POS
Riyadh-2888905	POS	POS	POS	POS	POS	POS	POS	AMB	POS	POS	POS
Riyadh-2888915	POS	POS	POS	POS	POS	POS	POS	NEG	POS	POS	POS
Riyadh-2567782	POS	POS	POS	POS	POS	POS	POS	NEG	POS	POS	POS
Riyadh-2891670	POS	POS	POS	POS	POS	POS	POS	AMB	POS	POS	POS
Riyadh-3006920	POS	POS	POS	POS	POS	POS	POS	AMB	POS	POS	POS
Riyadh-2817276-1	POS	POS	POS	POS	POS	POS	POS	AMB	POS	POS	POS
Riyadh-0295102	POS	POS	POS	POS	POS	POS	POS	AMB	POS	POS	POS
Riyadh-2820597	POS	POS	POS	POS	POS	POS	POS	POS	POS	POS	POS
Riyadh-2822088	POS	POS	POS	POS	POS	POS	POS	POS	POS	POS	POS
Riyadh-3010092	POS	POS	POS	POS	POS	POS	POS	AMB	POS	POS	POS
Riyadh-3022844	POS	POS	POS	POS	POS	POS	POS	AMB	POS	POS	POS
Riyadh-3108214-2	POS	POS	POS	POS	POS	POS	POS	NEG	POS	POS	POS
Riyadh-2823926	POS	POS	POS	POS	POS	POS	POS	AMB	POS	POS	POS
Riyadh-1	POS	POS	POS	POS	POS	POS	POS	AMB	POS	POS	POS
Riyadh-2818388	POS	POS	POS	POS	POS	POS	POS	AMB	POS	POS	POS
Riyadh-2818316	POS	POS	POS	POS	POS	POS	POS	POS	POS	POS	POS
**CC9/ST834-MRSA-(atypical SCCmec)**											
Riyadh-3103521	POS	POS	POS	POS	POS	POS	POS	NEG	POS	POS	POS
**CC22-MRSA-IV, Barnim/UK-EMRSA-15**											
Riyadh-2553359	POS	POS	NEG	NEG	POS	POS	POS	NEG	POS	POS	POS
Riyadh-2571758	POS	POS	NEG	NEG	POS	POS	POS	AMB	POS	POS	POS
Riyadh-3029203	POS	POS	NEG	NEG	POS	POS	POS	NEG	POS	POS	POS
Riyadh-3039785	POS	POS	NEG	NEG	POS	POS	POS	NEG	POS	POS	POS
Riyadh-3105594	POS	POS	NEG	NEG	POS	POS	POS	NEG	POS	POS	POS
Riyadh_IC_204-2	POS	POS	NEG	NEG	POS	POS	POS	AMB	POS	POS	POS
Riyadh-3003974	POS	POS	NEG	NEG	POS	POS	POS	NEG	POS	POS	POS
Riyadh_IC_067	POS	POS	NEG	NEG	POS	POS	POS	POS	NEG	AMB	NEG
Riyadh-2988627	POS	POS	NEG	NEG	POS	POS	POS	AMB	POS	POS	POS
Riyadh-3112581	POS	POS	NEG	NEG	POS	POS	POS	NEG	POS	POS	POS
**CC22-MRSA-IV [PVL+]**											
Riyadh-2781996-1	POS	POS	NEG	NEG	POS	POS	POS	NEG	POS	POS	POS
Riyadh-3103432	POS	POS	NEG	NEG	POS	POS	POS	NEG	POS	POS	POS
Riyadh-3026502	POS	AMB	NEG	NEG	POS	POS	POS	NEG	POS	POS	POS
Riyadh-3081378-2	POS	POS	NEG	NEG	POS	POS	POS	AMB	POS	POS	POS
Riyadh_IC_185	POS	POS	AMB	NEG	POS	POS	POS	AMB	POS	POS	POS
Riyadh_IC_204-1	POS	POS	NEG	NEG	POS	POS	POS	AMB	POS	POS	POS
Riyadh-2559371	POS	POS	NEG	NEG	POS	POS	POS	NEG	POS	POS	POS
Riyadh-2753975	POS	POS	NEG	NEG	POS	POS	POS	NEG	POS	POS	POS
Riyadh-2775605	POS	POS	NEG	NEG	POS	POS	POS	NEG	POS	POS	POS
Riyadh-2781996-2	POS	POS	NEG	NEG	POS	POS	POS	NEG	POS	POS	POS
Riyadh-2823783-2	POS	POS	NEG	NEG	POS	POS	POS	AMB	POS	POS	POS
Riyadh-2876601	POS	POS	NEG	NEG	POS	POS	POS	POS	POS	POS	POS
Riyadh-3036074	POS	POS	NEG	NEG	POS	POS	POS	AMB	POS	POS	POS
Riyadh-3053099	POS	POS	NEG	NEG	POS	POS	POS	NEG	POS	POS	POS
Riyadh-3055366	POS	POS	NEG	NEG	POS	POS	POS	NEG	POS	POS	POS
Riyadh-3082712	POS	POS	NEG	NEG	POS	POS	POS	NEG	POS	POS	POS
Riyadh-3087502	POS	AMB	NEG	NEG	POS	POS	POS	NEG	POS	POS	POS
Riyadh-6	POS	POS	NEG	NEG	POS	POS	POS	NEG	POS	POS	POS
Riyadh-7	POS	POS	NEG	NEG	POS	POS	POS	NEG	POS	POS	POS
Riyadh-8	POS	POS	NEG	NEG	POS	POS	POS	NEG	POS	POS	POS
**CC30-MRSA-IV [PVL+], Southwest Pacific Clone**											
Riyadh-10	POS	POS	POS	POS	POS	POS	POS	NEG	POS	POS	POS
Riyadh_IC_123	POS	POS	POS	POS	POS	POS	POS	AMB	POS	POS	POS
Riyadh-3080713	POS	POS	POS	POS	POS	POS	POS	AMB	POS	POS	POS
Riyadh-2803856	POS	POS	POS	POS	POS	POS	POS	POS	POS	POS	POS
Riyadh-2817276-1	POS	POS	POS	POS	POS	POS	POS	POS	POS	POS	POS
Riyadh-2817571-2	POS	POS	POS	POS	POS	POS	POS	POS	POS	POS	POS
Riyadh-3033868	POS	POS	POS	POS	AMB	POS	AMB	NEG	POS	POS	POS
Riyadh-2550108	POS	POS	POS	POS	POS	POS	POS	AMB	POS	POS	POS
Riyadh-3095056-2	POS	POS	POS	POS	POS	POS	POS	NEG	POS	POS	POS
Riyadh-2819899	POS	POS	POS	POS	POS	POS	POS	AMB	POS	POS	POS
Riyadh-2821805	POS	POS	POS	POS	AMB	AMB	AMB	NEG	POS	POS	POS
Riyadh-3013928	POS	POS	POS	POS	POS	POS	POS	AMB	POS	POS	POS
Riyadh-3029402	POS	POS	POS	POS	POS	POS	POS	AMB	POS	POS	POS
**CC45/agr IV-MRSA-IV, WA MRSA-23**											
Riyadh-3081378-1	POS	POS	POS	NEG	NEG	NEG	NEG	NEG	POS	POS	POS
**CC80-MRSA-IV**											
Riyadh-3107635	POS	POS	POS	POS	NEG	POS	NEG	NEG	POS	NEG	POS
Riyadh-2987458	POS	POS	POS	POS	POS	POS	POS	NEG	POS	POS	POS
**CC80-MRSA-IV [PVL+], European caMRSA Clone**											
Riyadh-2988048	POS	POS	POS	POS	AMB	POS	NEG	NEG	POS	NEG	POS
Riyadh-2990585-2	POS	POS	POS	POS	AMB	POS	NEG	NEG	POS	NEG	POS
Riyadh-2990585-1	POS	POS	POS	POS	POS	POS	NEG	AMB	POS	NEG	POS
Riyadh-2826033	POS	POS	POS	POS	POS	POS	NEG	AMB	POS	NEG	POS
Riyadh-1601562	POS	POS	POS	POS	POS	POS	NEG	NEG	POS	NEG	POS
Riyadh-2569940	POS	POS	POS	POS	POS	POS	AMB	AMB	POS	AMB	POS
Riyadh-2571692	POS	POS	POS	POS	POS	POS	NEG	AMB	POS	AMB	POS
Riyadh-2763029	POS	POS	POS	POS	AMB	POS	NEG	AMB	POS	NEG	POS
Riyadh-2767090	POS	POS	POS	POS	AMB	POS	NEG	NEG	POS	NEG	POS
Riyadh-2775130	POS	POS	POS	POS	AMB	POS	NEG	NEG	POS	NEG	POS
Riyadh-2778256	POS	POS	POS	POS	POS	POS	NEG	NEG	POS	NEG	POS
Riyadh-2817505	POS	POS	POS	POS	POS	POS	AMB	AMB	POS	NEG	POS
Riyadh-3024912	POS	POS	POS	POS	AMB	POS	NEG	NEG	POS	NEG	POS
Riyadh-2788690	POS	POS	POS	POS	AMB	POS	NEG	NEG	POS	NEG	POS
Riyadh-2829034	POS	POS	POS	POS	POS	POS	NEG	AMB	POS	AMB	POS
Riyadh-3	POS	POS	POS	POS	NEG	POS	NEG	NEG	POS	AMB	POS
Riyadh-5	POS	POS	POS	POS	POS	POS	NEG	AMB	POS	AMB	POS
Riyadh-2553167	POS	POS	POS	POS	POS	POS	POS	NEG	POS	AMB	POS
Riyadh-3002592	POS	POS	POS	POS	POS	POS	POS	AMB	POS	NEG	POS
**CC88-MRSA-IV [PVL+]**											
Riyadh-2736996	POS	POS	POS	POS	POS	POS	POS	POS	NEG	NEG	NEG
Riyadh-2942396	POS	POS	POS	POS	POS	POS	POS	AMB	POS	POS	POS
Riyadh-3105391	POS	POS	POS	POS	POS	POS	POS	NEG	POS	POS	POS
**CC97-MRSA-V**											
Riyadh-0297622	POS	POS	POS	POS	POS	POS	POS	NEG	POS	NEG	POS
Riyadh-3025471	POS	POS	POS	POS	POS	POS	POS	POS	POS	AMB	POS

	VIRULENCE : EXFOL.TOXINS			VIRULENCE : EPITHEL. DIFF. INHIB			VIRULENCE : ACME LOCUS			
	etA	etB	etD	edinA	edinB	edinC	arcA-SCC	arcB-SCC	arcC-SCC	arcD-SCC
	exfoliative toxin serotype A	exfoliative toxin serotype B	exfoliative toxin D	epidermal cell differen-tiation inhibitor	epidermal cell differen-tiation inhibitor B	epidermal cell differen-tiation inhibitor C	ACME-locus	ACME-locus: ornithincarb-amoyltrans-ferase	ACME-locus: carbamat-kinase	ACME-locus: arginine/orni-thine-antiporter
**CC1-MRSA-IV&SCCfus, WA MRSA-1/45**										
Riyadh-3108609	NEG	NEG	NEG	NEG	NEG	NEG	NEG	NEG	NEG	NEG
**CC1/ST772-MRSA-V [PVL+], "Bengal Bay Clone/WA I**										
Riyadh-2819026	NEG	NEG	NEG	NEG	NEG	NEG	NEG	NEG	NEG	NEG
**CC5-MRSA-IV, Paediatric clone**										
Riyadh-2915327-1	NEG	NEG	NEG	NEG	NEG	NEG	NEG	NEG	NEG	NEG
Riyadh-2915327-2	NEG	NEG	NEG	NEG	NEG	NEG	NEG	NEG	NEG	NEG
Riyadh-2	NEG	NEG	NEG	POS	NEG	NEG	NEG	NEG	NEG	NEG
**CC5-MRSA-IV [PVL+], Paediatric clone**										
Riyadh-2986666	NEG	NEG	NEG	NEG	NEG	NEG	NEG	NEG	NEG	NEG
Riyadh-2913335	NEG	NEG	NEG	NEG	NEG	NEG	NEG	NEG	NEG	NEG
**CC5-MRSA-IVvar, "Maltese Clone"**										
Riyadh-2983654	NEG	NEG	NEG	NEG	NEG	NEG	NEG	NEG	NEG	NEG
Riyadh-4	NEG	NEG	NEG	NEG	NEG	NEG	NEG	NEG	NEG	NEG
Riyadh-2790233	NEG	NEG	NEG	NEG	NEG	NEG	NEG	NEG	NEG	NEG
**CC5-MRSA-V**										
Riyadh-2568944	NEG	NEG	NEG	NEG	NEG	NEG	NEG	NEG	NEG	NEG
**CC6-MRSA-IV, WA MRSA-51/66**										
Riyadh-2556168	NEG	NEG	NEG	NEG	NEG	NEG	NEG	NEG	NEG	NEG
Riyadh-2824507	NEG	NEG	NEG	NEG	NEG	NEG	NEG	NEG	NEG	NEG
Riyadh-2990831	NEG	NEG	NEG	NEG	NEG	NEG	NEG	NEG	NEG	NEG
**CC8/ST239-MRSA-III, Vienna/Hungarian/Brazilian Cl**										
Riyadh-9	NEG	NEG	NEG	NEG	NEG	NEG	NEG	NEG	NEG	NEG
Riyadh-3028763	NEG	NEG	NEG	NEG	NEG	NEG	NEG	NEG	NEG	NEG
Riyadh-2817437	NEG	NEG	NEG	NEG	NEG	NEG	NEG	NEG	NEG	NEG
Riyadh-2793706	NEG	NEG	NEG	NEG	NEG	NEG	NEG	NEG	NEG	NEG
Riyadh-2818797	NEG	NEG	NEG	NEG	NEG	NEG	NEG	NEG	NEG	NEG
Riyadh-2822825	NEG	NEG	NEG	NEG	NEG	NEG	NEG	NEG	NEG	NEG
Riyadh-2888905	NEG	NEG	NEG	NEG	NEG	NEG	NEG	NEG	NEG	NEG
Riyadh-2888915	NEG	NEG	NEG	NEG	NEG	NEG	NEG	NEG	NEG	NEG
Riyadh-2567782	NEG	NEG	NEG	NEG	NEG	NEG	NEG	NEG	NEG	NEG
Riyadh-2891670	NEG	NEG	NEG	NEG	NEG	NEG	NEG	NEG	NEG	NEG
Riyadh-3006920	NEG	NEG	NEG	NEG	NEG	NEG	NEG	NEG	NEG	NEG
Riyadh-2817276-1	NEG	NEG	NEG	NEG	NEG	NEG	NEG	NEG	NEG	NEG
Riyadh-0295102	NEG	NEG	NEG	NEG	NEG	NEG	NEG	NEG	NEG	NEG
Riyadh-2820597	NEG	NEG	NEG	NEG	NEG	NEG	NEG	NEG	NEG	NEG
Riyadh-2822088	NEG	NEG	NEG	NEG	NEG	NEG	NEG	NEG	NEG	NEG
Riyadh-3010092	NEG	NEG	NEG	NEG	NEG	NEG	NEG	NEG	NEG	NEG
Riyadh-3022844	NEG	NEG	NEG	NEG	NEG	NEG	NEG	NEG	NEG	NEG
Riyadh-3108214-2	NEG	NEG	NEG	NEG	NEG	NEG	NEG	NEG	NEG	NEG
Riyadh-2823926	NEG	NEG	NEG	NEG	NEG	NEG	NEG	AMB	NEG	AMB
Riyadh-1	NEG	NEG	NEG	NEG	NEG	NEG	NEG	NEG	NEG	NEG
Riyadh-2818388	NEG	NEG	NEG	NEG	NEG	NEG	NEG	NEG	NEG	NEG
Riyadh-2818316	NEG	NEG	NEG	NEG	NEG	NEG	NEG	NEG	NEG	NEG
**CC9/ST834-MRSA-(atypical SCCmec)**										
Riyadh-3103521	NEG	NEG	NEG	NEG	NEG	NEG	NEG	NEG	NEG	NEG
**CC22-MRSA-IV, Barnim/UK-EMRSA-15**										
Riyadh-2553359	NEG	NEG	NEG	NEG	NEG	NEG	NEG	NEG	NEG	NEG
Riyadh-2571758	NEG	NEG	NEG	NEG	NEG	NEG	NEG	NEG	NEG	NEG
Riyadh-3029203	NEG	NEG	NEG	NEG	NEG	NEG	NEG	NEG	NEG	NEG
Riyadh-3039785	NEG	NEG	NEG	NEG	NEG	NEG	NEG	NEG	NEG	NEG
Riyadh-3105594	NEG	NEG	NEG	NEG	NEG	NEG	NEG	NEG	NEG	NEG
Riyadh_IC_204-2	NEG	NEG	NEG	NEG	NEG	NEG	NEG	NEG	NEG	NEG
Riyadh-3003974	NEG	NEG	NEG	NEG	NEG	NEG	NEG	NEG	NEG	NEG
Riyadh_IC_067	NEG	NEG	NEG	NEG	NEG	NEG	NEG	NEG	NEG	NEG
Riyadh-2988627	NEG	NEG	NEG	NEG	NEG	NEG	NEG	NEG	NEG	NEG
Riyadh-3112581	NEG	NEG	NEG	NEG	NEG	NEG	NEG	NEG	NEG	NEG
**CC22-MRSA-IV [PVL+]**										
Riyadh-2781996-1	NEG	NEG	NEG	NEG	NEG	NEG	NEG	NEG	NEG	NEG
Riyadh-3103432	NEG	NEG	NEG	NEG	NEG	NEG	NEG	NEG	NEG	NEG
Riyadh-3026502	NEG	NEG	NEG	NEG	NEG	NEG	NEG	NEG	NEG	NEG
Riyadh-3081378-2	NEG	NEG	NEG	NEG	NEG	NEG	NEG	NEG	NEG	NEG
Riyadh_IC_185	NEG	NEG	NEG	NEG	NEG	NEG	NEG	NEG	NEG	NEG
Riyadh_IC_204-1	NEG	NEG	NEG	NEG	NEG	NEG	NEG	NEG	NEG	NEG
Riyadh-2559371	NEG	NEG	NEG	NEG	NEG	NEG	NEG	NEG	NEG	NEG
Riyadh-2753975	NEG	NEG	NEG	NEG	NEG	NEG	NEG	NEG	NEG	NEG
Riyadh-2775605	NEG	NEG	NEG	NEG	NEG	NEG	NEG	NEG	NEG	NEG
Riyadh-2781996-2	NEG	NEG	NEG	NEG	NEG	NEG	NEG	NEG	NEG	NEG
Riyadh-2823783-2	NEG	NEG	NEG	NEG	NEG	NEG	NEG	NEG	NEG	NEG
Riyadh-2876601	NEG	NEG	NEG	NEG	NEG	NEG	NEG	NEG	NEG	NEG
Riyadh-3036074	NEG	NEG	NEG	NEG	NEG	NEG	NEG	NEG	NEG	NEG
Riyadh-3053099	NEG	NEG	NEG	NEG	NEG	NEG	NEG	NEG	NEG	NEG
Riyadh-3055366	NEG	NEG	NEG	NEG	NEG	NEG	NEG	NEG	NEG	NEG
Riyadh-3082712	NEG	NEG	NEG	NEG	NEG	NEG	NEG	NEG	NEG	NEG
Riyadh-3087502	NEG	NEG	NEG	NEG	NEG	NEG	NEG	NEG	NEG	NEG
Riyadh-6	NEG	NEG	NEG	NEG	NEG	NEG	NEG	NEG	NEG	NEG
Riyadh-7	NEG	NEG	NEG	NEG	NEG	NEG	NEG	NEG	NEG	NEG
Riyadh-8	NEG	NEG	NEG	NEG	NEG	NEG	NEG	NEG	NEG	NEG
**CC30-MRSA-IV [PVL+], Southwest Pacific Clone**										
Riyadh-10	NEG	NEG	NEG	NEG	NEG	NEG	NEG	NEG	NEG	NEG
Riyadh_IC_123	NEG	NEG	NEG	NEG	NEG	NEG	NEG	NEG	NEG	NEG
Riyadh-3080713	NEG	NEG	NEG	NEG	NEG	NEG	NEG	NEG	NEG	NEG
Riyadh-2803856	NEG	NEG	NEG	NEG	NEG	NEG	NEG	NEG	NEG	NEG
Riyadh-2817276-1	NEG	NEG	NEG	NEG	NEG	NEG	NEG	NEG	NEG	NEG
Riyadh-2817571-2	NEG	NEG	NEG	NEG	NEG	NEG	NEG	NEG	NEG	NEG
Riyadh-3033868	NEG	NEG	NEG	NEG	NEG	NEG	NEG	NEG	NEG	NEG
Riyadh-2550108	NEG	NEG	NEG	NEG	NEG	NEG	NEG	NEG	NEG	NEG
Riyadh-3095056-2	NEG	NEG	NEG	NEG	NEG	NEG	NEG	NEG	NEG	NEG
Riyadh-2819899	NEG	NEG	NEG	NEG	NEG	NEG	NEG	NEG	NEG	NEG
Riyadh-2821805	NEG	NEG	NEG	NEG	NEG	NEG	NEG	NEG	NEG	NEG
Riyadh-3013928	NEG	NEG	NEG	NEG	NEG	NEG	NEG	NEG	NEG	NEG
Riyadh-3029402	NEG	NEG	NEG	NEG	NEG	NEG	NEG	NEG	NEG	NEG
**CC45/agr IV-MRSA-IV, WA MRSA-23**										
Riyadh-3081378-1	NEG	NEG	NEG	NEG	NEG	NEG	NEG	NEG	NEG	NEG
**CC80-MRSA-IV**										
Riyadh-3107635	NEG	NEG	POS	NEG	POS	NEG	NEG	NEG	NEG	NEG
Riyadh-2987458	NEG	NEG	POS	NEG	POS	NEG	NEG	NEG	NEG	NEG
**CC80-MRSA-IV [PVL+], European caMRSA Clone**										
Riyadh-2988048	NEG	NEG	POS	NEG	POS	NEG	NEG	NEG	NEG	NEG
Riyadh-2990585-2	NEG	NEG	POS	NEG	POS	NEG	NEG	NEG	NEG	NEG
Riyadh-2990585-1	NEG	NEG	POS	NEG	POS	NEG	NEG	NEG	NEG	NEG
Riyadh-2826033	NEG	NEG	POS	NEG	POS	NEG	NEG	NEG	NEG	NEG
Riyadh-1601562	NEG	NEG	POS	NEG	POS	NEG	NEG	NEG	NEG	NEG
Riyadh-2569940	NEG	NEG	POS	NEG	POS	NEG	NEG	NEG	NEG	NEG
Riyadh-2571692	NEG	NEG	POS	NEG	POS	NEG	NEG	NEG	NEG	NEG
Riyadh-2763029	NEG	NEG	POS	NEG	POS	NEG	NEG	NEG	NEG	NEG
Riyadh-2767090	NEG	NEG	POS	NEG	POS	NEG	NEG	NEG	NEG	NEG
Riyadh-2775130	NEG	NEG	POS	NEG	POS	NEG	NEG	NEG	NEG	NEG
Riyadh-2778256	NEG	NEG	POS	NEG	POS	NEG	NEG	NEG	NEG	NEG
Riyadh-2817505	NEG	NEG	POS	NEG	POS	NEG	NEG	NEG	NEG	NEG
Riyadh-3024912	NEG	NEG	POS	NEG	POS	NEG	NEG	NEG	NEG	NEG
Riyadh-2788690	NEG	NEG	POS	NEG	POS	NEG	NEG	NEG	NEG	NEG
Riyadh-2829034	NEG	NEG	POS	NEG	POS	NEG	NEG	NEG	NEG	NEG
Riyadh-3	NEG	NEG	POS	NEG	POS	NEG	NEG	NEG	NEG	NEG
Riyadh-5	NEG	NEG	POS	NEG	POS	NEG	NEG	NEG	NEG	NEG
Riyadh-2553167	NEG	NEG	POS	NEG	POS	NEG	NEG	NEG	NEG	NEG
Riyadh-3002592	NEG	NEG	POS	NEG	POS	NEG	NEG	NEG	NEG	NEG
**CC88-MRSA-IV [PVL+]**										
Riyadh-2736996	NEG	NEG	NEG	NEG	NEG	NEG	NEG	NEG	NEG	NEG
Riyadh-2942396	NEG	NEG	NEG	NEG	NEG	NEG	NEG	NEG	NEG	NEG
Riyadh-3105391	NEG	NEG	NEG	NEG	NEG	NEG	NEG	NEG	NEG	NEG
**CC97-MRSA-V**										
Riyadh-0297622	NEG	NEG	NEG	NEG	NEG	NEG	NEG	NEG	NEG	NEG
Riyadh-3025471	NEG	NEG	NEG	NEG	NEG	NEG	NEG	NEG	NEG	NEG

	VIRULENCE : PROTEASES									
	aur (cons)	aur (Other than MRSA252)	aur (MRSA252)	splA	splB	splE	sspA	sspB	sspP (cons)	sspP (other than ST93)
	aureolysin			serin-protease A	serin-protease B	serin-protease E	glutamylendopeptidase	staphopain B, protease	staphopain A (staphylopain A), protease	
**CC1-MRSA-IV&SCCfus, WA MRSA-1/45**										
Riyadh-3108609	POS	POS	NEG	POS	POS	POS	POS	POS	POS	POS
**CC1/ST772-MRSA-V [PVL+], "Bengal Bay Clone/WA I**										
Riyadh-2819026	POS	POS	NEG	NEG	NEG	NEG	POS	POS	POS	POS
**CC5-MRSA-IV, Paediatric clone**										
Riyadh-2915327-1	POS	POS	NEG	POS	POS	NEG	POS	POS	POS	POS
Riyadh-2915327-2	POS	POS	NEG	POS	POS	NEG	POS	POS	POS	POS
Riyadh-2	POS	POS	NEG	POS	POS	NEG	POS	POS	POS	POS
**CC5-MRSA-IV [PVL+], Paediatric clone**										
Riyadh-2986666	POS	POS	NEG	POS	POS	NEG	POS	POS	POS	POS
Riyadh-2913335	POS	POS	NEG	POS	POS	NEG	POS	POS	POS	POS
**CC5-MRSA-IVvar, "Maltese Clone"**										
Riyadh-2983654	POS	POS	NEG	POS	POS	NEG	POS	POS	POS	POS
Riyadh-4	POS	POS	NEG	POS	POS	NEG	POS	POS	POS	POS
Riyadh-2790233	POS	POS	NEG	POS	POS	NEG	POS	POS	POS	POS
**CC5-MRSA-V**										
Riyadh-2568944	POS	POS	NEG	POS	POS	NEG	POS	POS	POS	POS
**CC6-MRSA-IV, WA MRSA-51/66**										
Riyadh-2556168	POS	POS	NEG	POS	POS	POS	POS	POS	POS	POS
Riyadh-2824507	POS	POS	NEG	POS	POS	POS	POS	POS	POS	POS
Riyadh-2990831	POS	POS	NEG	POS	POS	POS	POS	POS	POS	POS
**CC8/ST239-MRSA-III, Vienna/Hungarian/Brazilian Cl**										
Riyadh-9	POS	NEG	POS	POS	POS	POS	POS	POS	POS	POS
Riyadh-3028763	POS	NEG	POS	POS	POS	NEG	POS	POS	POS	POS
Riyadh-2817437	POS	NEG	POS	POS	POS	NEG	POS	POS	POS	POS
Riyadh-2793706	POS	NEG	POS	POS	POS	NEG	POS	POS	POS	POS
Riyadh-2818797	POS	NEG	POS	POS	POS	NEG	POS	POS	POS	POS
Riyadh-2822825	POS	NEG	POS	POS	POS	NEG	POS	POS	POS	POS
Riyadh-2888905	POS	NEG	POS	POS	POS	NEG	POS	POS	POS	POS
Riyadh-2888915	POS	NEG	POS	POS	POS	NEG	POS	POS	POS	POS
Riyadh-2567782	POS	NEG	POS	POS	POS	NEG	POS	POS	POS	POS
Riyadh-2891670	POS	NEG	POS	POS	POS	NEG	POS	POS	POS	POS
Riyadh-3006920	POS	NEG	POS	POS	POS	NEG	POS	POS	POS	POS
Riyadh-2817276-1	POS	NEG	POS	POS	POS	NEG	POS	POS	POS	POS
Riyadh-0295102	POS	NEG	POS	POS	POS	NEG	POS	POS	POS	AMB
Riyadh-2820597	POS	NEG	POS	POS	POS	NEG	POS	POS	POS	POS
Riyadh-2822088	POS	NEG	POS	POS	POS	NEG	POS	POS	POS	POS
Riyadh-3010092	POS	NEG	POS	POS	POS	NEG	POS	POS	POS	POS
Riyadh-3022844	POS	NEG	POS	POS	POS	NEG	POS	POS	POS	POS
Riyadh-3108214-2	POS	NEG	POS	POS	POS	NEG	POS	POS	POS	POS
Riyadh-2823926	POS	NEG	POS	POS	POS	NEG	POS	POS	POS	POS
Riyadh-1	POS	NEG	POS	POS	POS	NEG	POS	POS	POS	POS
Riyadh-2818388	POS	NEG	POS	POS	POS	NEG	POS	POS	POS	POS
Riyadh-2818316	POS	NEG	POS	POS	POS	NEG	POS	POS	POS	POS
**CC9/ST834-MRSA-(atypical SCCmec)**										
Riyadh-3103521	POS	POS	NEG	POS	POS	POS	POS	POS	POS	POS
**CC22-MRSA-IV, Barnim/UK-EMRSA-15**										
Riyadh-2553359	POS	NEG	POS	NEG	NEG	NEG	POS	POS	POS	POS
Riyadh-2571758	POS	NEG	POS	NEG	NEG	NEG	POS	POS	POS	POS
Riyadh-3029203	POS	NEG	POS	NEG	NEG	NEG	POS	POS	POS	POS
Riyadh-3039785	POS	NEG	POS	NEG	NEG	NEG	POS	POS	POS	POS
Riyadh-3105594	POS	NEG	POS	NEG	NEG	NEG	POS	POS	POS	POS
Riyadh_IC_204-2	POS	NEG	POS	NEG	NEG	NEG	POS	POS	POS	POS
Riyadh-3003974	POS	NEG	POS	NEG	NEG	NEG	POS	POS	POS	POS
Riyadh_IC_067	POS	NEG	POS	NEG	NEG	NEG	POS	POS	POS	POS
Riyadh-2988627	POS	NEG	POS	NEG	NEG	NEG	POS	POS	POS	POS
Riyadh-3112581	POS	NEG	POS	NEG	NEG	NEG	POS	POS	POS	POS
**CC22-MRSA-IV [PVL+]**										
Riyadh-2781996-1	POS	NEG	POS	NEG	NEG	NEG	POS	POS	POS	POS
Riyadh-3103432	POS	NEG	POS	NEG	NEG	NEG	POS	POS	POS	POS
Riyadh-3026502	POS	NEG	POS	NEG	NEG	NEG	POS	POS	POS	POS
Riyadh-3081378-2	POS	NEG	POS	NEG	NEG	NEG	POS	POS	POS	POS
Riyadh_IC_185	POS	NEG	POS	NEG	NEG	NEG	POS	POS	POS	POS
Riyadh_IC_204-1	POS	NEG	POS	NEG	NEG	NEG	POS	POS	POS	POS
Riyadh-2559371	POS	NEG	POS	NEG	NEG	NEG	POS	POS	POS	POS
Riyadh-2753975	POS	NEG	POS	NEG	NEG	NEG	POS	POS	POS	POS
Riyadh-2775605	POS	NEG	POS	NEG	NEG	NEG	POS	POS	POS	POS
Riyadh-2781996-2	POS	NEG	POS	NEG	NEG	NEG	POS	POS	POS	POS
Riyadh-2823783-2	POS	NEG	POS	NEG	NEG	NEG	POS	POS	POS	POS
Riyadh-2876601	POS	NEG	POS	NEG	NEG	NEG	POS	POS	POS	POS
Riyadh-3036074	POS	NEG	POS	NEG	NEG	NEG	POS	POS	POS	POS
Riyadh-3053099	POS	NEG	POS	NEG	NEG	NEG	POS	POS	POS	POS
Riyadh-3055366	POS	NEG	POS	NEG	NEG	NEG	POS	POS	POS	POS
Riyadh-3082712	POS	NEG	POS	NEG	NEG	NEG	POS	POS	POS	POS
Riyadh-3087502	POS	NEG	POS	NEG	NEG	NEG	POS	POS	POS	POS
Riyadh-6	POS	NEG	POS	NEG	NEG	NEG	POS	POS	POS	POS
Riyadh-7	POS	NEG	POS	NEG	NEG	NEG	POS	POS	POS	POS
Riyadh-8	POS	NEG	POS	NEG	NEG	NEG	POS	POS	POS	POS
**CC30-MRSA-IV [PVL+], Southwest Pacific Clone**										
Riyadh-10	POS	NEG	POS	NEG	NEG	POS	POS	POS	POS	POS
Riyadh_IC_123	POS	NEG	POS	NEG	NEG	POS	POS	POS	POS	POS
Riyadh-3080713	POS	NEG	POS	NEG	NEG	POS	POS	POS	POS	POS
Riyadh-2803856	POS	NEG	POS	NEG	NEG	POS	POS	POS	POS	POS
Riyadh-2817276-1	POS	NEG	POS	NEG	NEG	POS	POS	POS	POS	POS
Riyadh-2817571-2	POS	NEG	POS	NEG	NEG	POS	POS	POS	POS	POS
Riyadh-3033868	POS	NEG	POS	NEG	NEG	POS	POS	POS	POS	POS
Riyadh-2550108	POS	NEG	POS	NEG	NEG	POS	POS	POS	POS	POS
Riyadh-3095056-2	POS	NEG	POS	NEG	NEG	POS	POS	POS	POS	POS
Riyadh-2819899	POS	NEG	POS	NEG	NEG	POS	POS	POS	POS	POS
Riyadh-2821805	POS	NEG	POS	NEG	NEG	POS	POS	POS	POS	POS
Riyadh-3013928	POS	NEG	POS	NEG	NEG	POS	POS	POS	POS	POS
Riyadh-3029402	POS	NEG	POS	NEG	NEG	POS	POS	POS	POS	POS
**CC45/agr IV-MRSA-IV, WA MRSA-23**										
Riyadh-3081378-1	POS	NEG	POS	NEG	NEG	NEG	POS	POS	POS	POS
**CC80-MRSA-IV**										
Riyadh-3107635	POS	POS	NEG	POS	POS	NEG	POS	POS	POS	POS
Riyadh-2987458	POS	POS	NEG	POS	POS	NEG	POS	POS	POS	POS
**CC80-MRSA-IV [PVL+], European caMRSA Clone**										
Riyadh-2988048	POS	POS	NEG	POS	POS	NEG	POS	POS	POS	POS
Riyadh-2990585-2	POS	POS	NEG	POS	POS	NEG	POS	POS	POS	POS
Riyadh-2990585-1	POS	POS	NEG	POS	POS	NEG	POS	POS	POS	POS
Riyadh-2826033	POS	POS	NEG	POS	POS	NEG	POS	POS	POS	POS
Riyadh-1601562	POS	POS	NEG	POS	POS	NEG	POS	POS	POS	POS
Riyadh-2569940	POS	POS	NEG	POS	POS	NEG	POS	POS	POS	POS
Riyadh-2571692	POS	POS	NEG	POS	POS	NEG	POS	POS	POS	POS
Riyadh-2763029	POS	POS	NEG	POS	POS	NEG	POS	POS	POS	POS
Riyadh-2767090	POS	POS	NEG	POS	POS	NEG	POS	POS	POS	POS
Riyadh-2775130	POS	POS	NEG	POS	POS	NEG	POS	POS	POS	POS
Riyadh-2778256	POS	POS	NEG	POS	POS	NEG	POS	POS	POS	POS
Riyadh-2817505	POS	POS	NEG	POS	POS	NEG	POS	POS	POS	POS
Riyadh-3024912	POS	POS	NEG	POS	POS	NEG	POS	POS	POS	POS
Riyadh-2788690	POS	POS	NEG	POS	POS	NEG	POS	POS	POS	POS
Riyadh-2829034	POS	POS	NEG	POS	POS	NEG	POS	POS	POS	POS
Riyadh-3	POS	POS	NEG	POS	POS	NEG	POS	POS	POS	POS
Riyadh-5	POS	POS	NEG	POS	POS	NEG	POS	POS	POS	POS
Riyadh-2553167	POS	POS	NEG	POS	POS	NEG	POS	POS	POS	POS
Riyadh-3002592	POS	POS	NEG	POS	POS	NEG	POS	POS	POS	POS
**CC88-MRSA-IV [PVL+]**										
Riyadh-2736996	POS	POS	NEG	POS	POS	NEG	POS	POS	POS	POS
Riyadh-2942396	POS	POS	NEG	POS	POS	NEG	POS	POS	POS	POS
Riyadh-3105391	POS	POS	NEG	POS	POS	NEG	POS	POS	POS	POS
**CC97-MRSA-V**										
Riyadh-0297622	POS	POS	NEG	POS	POS	POS	POS	POS	POS	POS
Riyadh-3025471	POS	POS	NEG	POS	POS	POS	POS	POS	POS	POS

	VIRULENCE : STAPHYLOCOCCAL SUPERANTIGEN/ENTEROTOXIN-LIKE GENES (SET/SSL)												
	setC	set6-var1_11	set6-var2_11	set6-var1_12	set6-var2_12	set6-var4_11	ssl01-RF122	ssl01/set6 (COL)	ssl01/set6 (Mu50+N315)	ssl01/set6 (MW2+MSSA476)	ssl01/set6 (MRSA252)	ssl01/set6 (RF122)	ssl01/set6 (other alleles)
	staphyl. exotoxin-like protein	staphylococcal superantigen-like protein 1											
**CC1-MRSA-IV&SCCfus, WA MRSA-1/45**													
Riyadh-3108609	POS	NEG	POS	NEG	POS	NEG	NEG	NEG	NEG	POS	NEG	NEG	NEG
**CC1/ST772-MRSA-V [PVL+], "Bengal Bay Clone/WA I**													
Riyadh-2819026	POS	POS	NEG	POS	NEG	POS	NEG	POS	AMB	NEG	NEG	NEG	NEG
**CC5-MRSA-IV, Paediatric clone**													
Riyadh-2915327-1	POS	POS	NEG	POS	NEG	POS	NEG	NEG	POS	NEG	NEG	NEG	NEG
Riyadh-2915327-2	POS	POS	NEG	POS	NEG	POS	NEG	NEG	POS	NEG	NEG	NEG	NEG
Riyadh-2	POS	POS	NEG	POS	NEG	POS	NEG	NEG	POS	NEG	NEG	NEG	NEG
**CC5-MRSA-IV [PVL+], Paediatric clone**													
Riyadh-2986666	POS	POS	AMB	POS	AMB	POS	NEG	POS	AMB	NEG	NEG	NEG	NEG
Riyadh-2913335	POS	POS	AMB	POS	AMB	POS	NEG	POS	AMB	NEG	NEG	NEG	NEG
**CC5-MRSA-IVvar, "Maltese Clone"**													
Riyadh-2983654	POS	POS	AMB	POS	AMB	POS	NEG	POS	AMB	NEG	NEG	NEG	NEG
Riyadh-4	POS	POS	NEG	POS	NEG	POS	NEG	NEG	POS	NEG	NEG	NEG	NEG
Riyadh-2790233	POS	POS	AMB	POS	NEG	POS	NEG	POS	AMB	NEG	NEG	NEG	NEG
**CC5-MRSA-V**													
Riyadh-2568944	POS	POS	NEG	POS	NEG	POS	NEG	NEG	POS	NEG	NEG	NEG	NEG
**CC6-MRSA-IV, WA MRSA-51/66**													
Riyadh-2556168	POS	NEG	NEG	AMB	NEG	POS	POS	NEG	NEG	NEG	NEG	NEG	POS
Riyadh-2824507	POS	NEG	NEG	AMB	NEG	POS	POS	NEG	NEG	NEG	NEG	NEG	POS
Riyadh-2990831	POS	NEG	NEG	POS	NEG	POS	POS	NEG	NEG	NEG	NEG	NEG	POS
**CC8/ST239-MRSA-III, Vienna/Hungarian/Brazilian Cl**													
Riyadh-9	POS	POS	NEG	POS	NEG	POS	NEG	POS	AMB	NEG	NEG	NEG	NEG
Riyadh-3028763	POS	POS	NEG	POS	NEG	POS	NEG	POS	AMB	NEG	NEG	NEG	NEG
Riyadh-2817437	POS	POS	NEG	POS	NEG	POS	NEG	POS	AMB	NEG	NEG	NEG	NEG
Riyadh-2793706	POS	POS	NEG	POS	NEG	POS	NEG	POS	AMB	NEG	NEG	NEG	NEG
Riyadh-2818797	POS	POS	NEG	POS	NEG	POS	NEG	POS	AMB	NEG	NEG	NEG	NEG
Riyadh-2822825	POS	POS	NEG	POS	NEG	POS	NEG	POS	AMB	NEG	NEG	NEG	NEG
Riyadh-2888905	POS	POS	NEG	POS	NEG	POS	NEG	POS	AMB	NEG	NEG	NEG	NEG
Riyadh-2888915	POS	POS	NEG	POS	NEG	POS	NEG	POS	AMB	NEG	NEG	NEG	NEG
Riyadh-2567782	POS	POS	NEG	POS	NEG	POS	NEG	POS	AMB	NEG	NEG	NEG	NEG
Riyadh-2891670	POS	POS	NEG	POS	NEG	POS	NEG	POS	AMB	NEG	NEG	NEG	NEG
Riyadh-3006920	POS	POS	NEG	POS	NEG	POS	NEG	POS	AMB	NEG	NEG	NEG	NEG
Riyadh-2817276-1	POS	POS	NEG	POS	NEG	POS	NEG	POS	AMB	NEG	NEG	NEG	NEG
Riyadh-0295102	POS	POS	NEG	POS	NEG	POS	NEG	POS	AMB	NEG	NEG	NEG	NEG
Riyadh-2820597	POS	POS	NEG	POS	NEG	POS	NEG	POS	AMB	NEG	NEG	NEG	NEG
Riyadh-2822088	POS	POS	NEG	POS	NEG	POS	NEG	POS	AMB	NEG	NEG	NEG	NEG
Riyadh-3010092	POS	POS	NEG	AMB	NEG	POS	NEG	POS	AMB	NEG	NEG	NEG	NEG
Riyadh-3022844	POS	POS	NEG	AMB	NEG	POS	NEG	POS	AMB	NEG	NEG	NEG	NEG
Riyadh-3108214-2	POS	POS	NEG	AMB	NEG	POS	NEG	POS	AMB	NEG	NEG	NEG	NEG
Riyadh-2823926	POS	POS	NEG	POS	NEG	POS	NEG	POS	AMB	NEG	NEG	NEG	NEG
Riyadh-1	POS	POS	NEG	POS	NEG	POS	NEG	POS	AMB	NEG	NEG	NEG	NEG
Riyadh-2818388	POS	POS	NEG	POS	NEG	POS	NEG	POS	AMB	NEG	NEG	NEG	NEG
Riyadh-2818316	POS	POS	NEG	POS	NEG	POS	NEG	POS	AMB	NEG	NEG	NEG	NEG
**CC9/ST834-MRSA-(atypical SCCmec)**													
Riyadh-3103521	POS	NEG	POS	NEG	NEG	NEG	NEG	NEG	NEG	NEG	NEG	NEG	POS
**CC22-MRSA-IV, Barnim/UK-EMRSA-15**													
Riyadh-2553359	POS	NEG	POS	POS	NEG	NEG	NEG	NEG	NEG	NEG	POS	NEG	NEG
Riyadh-2571758	POS	NEG	POS	POS	NEG	NEG	NEG	NEG	NEG	NEG	POS	NEG	NEG
Riyadh-3029203	POS	NEG	POS	POS	NEG	NEG	NEG	NEG	NEG	NEG	POS	NEG	NEG
Riyadh-3039785	POS	NEG	POS	POS	NEG	NEG	NEG	NEG	NEG	NEG	POS	NEG	NEG
Riyadh-3105594	POS	NEG	POS	POS	NEG	NEG	NEG	NEG	NEG	NEG	POS	NEG	NEG
Riyadh_IC_204-2	POS	NEG	POS	POS	NEG	NEG	NEG	NEG	NEG	NEG	POS	NEG	NEG
Riyadh-3003974	POS	NEG	POS	POS	NEG	NEG	NEG	NEG	NEG	NEG	POS	NEG	NEG
Riyadh_IC_067	POS	NEG	POS	POS	NEG	NEG	NEG	NEG	NEG	NEG	POS	NEG	NEG
Riyadh-2988627	POS	NEG	POS	POS	NEG	NEG	NEG	NEG	NEG	NEG	POS	NEG	NEG
Riyadh-3112581	POS	NEG	POS	POS	NEG	NEG	NEG	NEG	NEG	NEG	POS	NEG	NEG
**CC22-MRSA-IV [PVL+]**													
Riyadh-2781996-1	POS	NEG	POS	POS	NEG	NEG	NEG	NEG	NEG	NEG	POS	NEG	NEG
Riyadh-3103432	POS	NEG	POS	POS	NEG	NEG	NEG	NEG	NEG	NEG	POS	NEG	NEG
Riyadh-3026502	POS	NEG	POS	POS	NEG	NEG	NEG	NEG	NEG	NEG	POS	NEG	NEG
Riyadh-3081378-2	POS	NEG	POS	POS	NEG	NEG	NEG	NEG	NEG	NEG	POS	NEG	NEG
Riyadh_IC_185	POS	NEG	POS	POS	NEG	NEG	NEG	NEG	NEG	NEG	POS	NEG	NEG
Riyadh_IC_204-1	POS	NEG	POS	POS	NEG	NEG	NEG	NEG	NEG	NEG	POS	NEG	NEG
Riyadh-2559371	POS	NEG	POS	POS	NEG	NEG	NEG	NEG	NEG	NEG	POS	NEG	NEG
Riyadh-2753975	POS	NEG	POS	POS	NEG	NEG	NEG	NEG	NEG	NEG	POS	NEG	NEG
Riyadh-2775605	POS	NEG	POS	POS	NEG	NEG	NEG	NEG	NEG	NEG	POS	NEG	NEG
Riyadh-2781996-2	POS	NEG	POS	POS	NEG	NEG	NEG	NEG	NEG	NEG	POS	NEG	NEG
Riyadh-2823783-2	POS	NEG	POS	POS	NEG	NEG	NEG	NEG	NEG	NEG	POS	NEG	NEG
Riyadh-2876601	POS	NEG	POS	POS	NEG	NEG	NEG	NEG	NEG	NEG	POS	NEG	NEG
Riyadh-3036074	POS	NEG	POS	POS	NEG	NEG	NEG	NEG	NEG	NEG	POS	NEG	NEG
Riyadh-3053099	POS	NEG	POS	POS	NEG	NEG	NEG	NEG	NEG	NEG	POS	NEG	NEG
Riyadh-3055366	POS	NEG	POS	POS	NEG	NEG	NEG	NEG	NEG	NEG	POS	NEG	NEG
Riyadh-3082712	POS	NEG	POS	POS	NEG	NEG	NEG	NEG	NEG	NEG	POS	NEG	NEG
Riyadh-3087502	POS	NEG	POS	AMB	NEG	NEG	NEG	NEG	NEG	NEG	NEG	NEG	POS
Riyadh-6	POS	NEG	POS	POS	NEG	NEG	NEG	NEG	NEG	NEG	POS	NEG	NEG
Riyadh-7	POS	NEG	POS	POS	NEG	NEG	NEG	NEG	NEG	NEG	POS	NEG	NEG
Riyadh-8	POS	NEG	POS	POS	NEG	NEG	NEG	NEG	NEG	NEG	POS	NEG	NEG
**CC30-MRSA-IV [PVL+], Southwest Pacific Clone**													
Riyadh-10	NEG	NEG	POS	POS	NEG	POS	NEG	NEG	NEG	NEG	POS	NEG	NEG
Riyadh_IC_123	NEG	NEG	POS	POS	NEG	POS	NEG	NEG	NEG	NEG	POS	NEG	NEG
Riyadh-3080713	NEG	NEG	POS	POS	NEG	POS	NEG	NEG	NEG	NEG	POS	NEG	NEG
Riyadh-2803856	NEG	NEG	POS	POS	NEG	POS	NEG	NEG	NEG	NEG	POS	NEG	NEG
Riyadh-2817276-1	NEG	NEG	POS	POS	AMB	POS	NEG	NEG	NEG	NEG	POS	NEG	NEG
Riyadh-2817571-2	NEG	NEG	POS	POS	NEG	POS	NEG	NEG	NEG	NEG	POS	NEG	NEG
Riyadh-3033868	NEG	NEG	POS	POS	NEG	POS	NEG	NEG	NEG	NEG	POS	NEG	NEG
Riyadh-2550108	NEG	NEG	POS	POS	NEG	POS	NEG	NEG	NEG	NEG	POS	NEG	NEG
Riyadh-3095056-2	NEG	NEG	POS	POS	NEG	POS	NEG	NEG	NEG	NEG	POS	NEG	NEG
Riyadh-2819899	NEG	NEG	POS	POS	NEG	POS	NEG	NEG	NEG	NEG	POS	NEG	NEG
Riyadh-2821805	NEG	NEG	POS	POS	NEG	POS	NEG	NEG	NEG	NEG	POS	NEG	NEG
Riyadh-3013928	NEG	NEG	POS	POS	NEG	POS	NEG	NEG	NEG	NEG	POS	NEG	NEG
Riyadh-3029402	NEG	NEG	POS	POS	NEG	POS	NEG	NEG	NEG	NEG	POS	NEG	NEG
**CC45/agr IV-MRSA-IV, WA MRSA-23**													
Riyadh-3081378-1	NEG	NEG	POS	NEG	NEG	NEG	NEG	NEG	NEG	NEG	NEG	NEG	POS
**CC80-MRSA-IV**													
Riyadh-3107635	POS	NEG	NEG	POS	NEG	POS	POS	NEG	NEG	NEG	NEG	NEG	POS
Riyadh-2987458	POS	NEG	NEG	AMB	NEG	POS	POS	NEG	NEG	NEG	NEG	NEG	POS
**CC80-MRSA-IV [PVL+], European caMRSA Clone**													
Riyadh-2988048	POS	NEG	NEG	AMB	NEG	POS	POS	NEG	NEG	NEG	NEG	NEG	POS
Riyadh-2990585-2	POS	NEG	NEG	AMB	NEG	POS	POS	NEG	NEG	NEG	NEG	NEG	POS
Riyadh-2990585-1	POS	NEG	NEG	AMB	NEG	POS	POS	NEG	NEG	NEG	NEG	NEG	POS
Riyadh-2826033	POS	NEG	NEG	POS	NEG	POS	POS	NEG	NEG	NEG	NEG	NEG	POS
Riyadh-1601562	POS	NEG	NEG	AMB	NEG	POS	POS	NEG	NEG	NEG	NEG	NEG	POS
Riyadh-2569940	POS	NEG	NEG	POS	NEG	POS	POS	NEG	NEG	NEG	NEG	NEG	POS
Riyadh-2571692	POS	NEG	NEG	POS	NEG	POS	POS	NEG	NEG	NEG	NEG	NEG	POS
Riyadh-2763029	POS	NEG	NEG	POS	NEG	POS	POS	NEG	NEG	NEG	NEG	NEG	POS
Riyadh-2767090	POS	NEG	NEG	AMB	NEG	POS	POS	NEG	NEG	NEG	NEG	NEG	POS
Riyadh-2775130	POS	NEG	NEG	AMB	NEG	POS	POS	NEG	NEG	NEG	NEG	NEG	POS
Riyadh-2778256	POS	NEG	NEG	AMB	NEG	POS	POS	NEG	NEG	NEG	NEG	NEG	POS
Riyadh-2817505	POS	NEG	NEG	POS	NEG	POS	POS	NEG	NEG	NEG	NEG	NEG	POS
Riyadh-3024912	POS	NEG	NEG	AMB	NEG	POS	POS	NEG	NEG	NEG	NEG	NEG	POS
Riyadh-2788690	POS	NEG	NEG	AMB	NEG	POS	POS	NEG	NEG	NEG	NEG	NEG	POS
Riyadh-2829034	POS	NEG	NEG	POS	NEG	POS	POS	NEG	NEG	NEG	NEG	NEG	POS
Riyadh-3	POS	NEG	NEG	POS	NEG	POS	POS	NEG	NEG	NEG	NEG	NEG	POS
Riyadh-5	POS	NEG	NEG	POS	NEG	POS	POS	NEG	NEG	NEG	NEG	NEG	POS
Riyadh-2553167	POS	NEG	NEG	POS	NEG	POS	POS	NEG	NEG	NEG	NEG	NEG	POS
Riyadh-3002592	POS	NEG	NEG	POS	NEG	POS	POS	NEG	NEG	NEG	NEG	NEG	POS
**CC88-MRSA-IV [PVL+]**													
Riyadh-2736996	POS	NEG	POS	POS	NEG	POS	AMB	NEG	NEG	NEG	POS	NEG	NEG
Riyadh-2942396	POS	NEG	POS	POS	NEG	POS	AMB	NEG	NEG	NEG	POS	NEG	NEG
Riyadh-3105391	POS	NEG	AMB	AMB	NEG	POS	NEG	NEG	NEG	NEG	NEG	NEG	POS
**CC97-MRSA-V**													
Riyadh-0297622	POS	NEG	NEG	NEG	NEG	NEG	POS	NEG	NEG	NEG	NEG	POS	NEG
Riyadh-3025471	POS	NEG	AMB	NEG	NEG	NEG	POS	NEG	NEG	NEG	NEG	NEG	POS

	VIRULENCE : STAPHYLOCOCCAL SUPERANTIGEN/ENTEROTOXIN-LIKE GENES (SET/SSL)												
	ssl02/set7	ssl02/set7 (MRSA252)	ssl03/set8_probe 1	ssl03/set8_probe 2	ssl03/set8 (MRSA252, SAR0424)	ssl04/set9	ssl04/set9 (MRSA252, SAR0425)	ssl05/set3_probe 1	ssl05/set3 (RF122, probe-611)	ssl05/set3_probe 2 (612)	ssl05/set3 (MRSA252)	ssl06/set21	ssl06 (NCTC8325+ MW2)
	staphylococcal superantigen-like protein 2		staphylococcal superantigen-like protein 3			staphylococcal superantigen-like protein 4		staphylococcal superantigen-like protein 5				staphylococcal superantigen-like protein 6	
**CC1-MRSA-IV&SCCfus, WA MRSA-1/45**													
Riyadh-3108609	POS	AMB	POS	POS	NEG	POS	NEG	POS	AMB	POS	NEG	POS	POS
**CC1/ST772-MRSA-V [PVL+], "Bengal Bay Clone/WA I**													
Riyadh-2819026	POS	AMB	POS	POS	NEG	POS	AMB	POS	AMB	POS	AMB	NEG	NEG
**CC5-MRSA-IV, Paediatric clone**													
Riyadh-2915327-1	POS	AMB	POS	POS	NEG	POS	NEG	POS	AMB	POS	NEG	NEG	NEG
Riyadh-2915327-2	POS	AMB	POS	POS	NEG	POS	NEG	POS	AMB	POS	NEG	NEG	NEG
Riyadh-2	POS	AMB	POS	POS	NEG	POS	NEG	POS	AMB	POS	NEG	NEG	NEG
**CC5-MRSA-IV [PVL+], Paediatric clone**													
Riyadh-2986666	POS	AMB	POS	POS	NEG	POS	AMB	POS	AMB	POS	NEG	NEG	NEG
Riyadh-2913335	POS	AMB	POS	POS	NEG	POS	NEG	POS	AMB	POS	NEG	NEG	NEG
**CC5-MRSA-IVvar, "Maltese Clone"**													
Riyadh-2983654	POS	AMB	POS	POS	NEG	POS	NEG	POS	AMB	POS	NEG	NEG	NEG
Riyadh-4	POS	AMB	POS	POS	NEG	POS	NEG	POS	AMB	POS	NEG	NEG	NEG
Riyadh-2790233	POS	AMB	POS	POS	NEG	POS	NEG	POS	AMB	POS	NEG	NEG	NEG
**CC5-MRSA-V**													
Riyadh-2568944	POS	AMB	POS	POS	NEG	POS	NEG	POS	AMB	POS	NEG	NEG	NEG
**CC6-MRSA-IV, WA MRSA-51/66**													
Riyadh-2556168	POS	AMB	POS	POS	NEG	POS	NEG	POS	AMB	POS	NEG	NEG	NEG
Riyadh-2824507	POS	AMB	POS	POS	NEG	POS	NEG	POS	AMB	POS	NEG	NEG	NEG
Riyadh-2990831	POS	AMB	POS	POS	NEG	POS	NEG	POS	AMB	POS	AMB	NEG	NEG
**CC8/ST239-MRSA-III, Vienna/Hungarian/Brazilian Cl**													
Riyadh-9	POS	AMB	POS	POS	NEG	POS	NEG	POS	AMB	POS	NEG	POS	POS
Riyadh-3028763	POS	AMB	POS	POS	NEG	POS	NEG	POS	AMB	POS	NEG	POS	POS
Riyadh-2817437	POS	AMB	POS	POS	NEG	POS	NEG	POS	POS	AMB	NEG	POS	POS
Riyadh-2793706	POS	AMB	POS	POS	NEG	POS	NEG	POS	POS	AMB	NEG	POS	POS
Riyadh-2818797	POS	AMB	POS	POS	NEG	POS	AMB	POS	AMB	POS	NEG	POS	POS
Riyadh-2822825	POS	AMB	POS	POS	NEG	POS	NEG	POS	POS	AMB	NEG	POS	POS
Riyadh-2888905	POS	AMB	POS	POS	NEG	POS	NEG	POS	AMB	POS	NEG	POS	POS
Riyadh-2888915	POS	AMB	POS	POS	NEG	POS	NEG	POS	POS	AMB	NEG	POS	POS
Riyadh-2567782	POS	AMB	POS	POS	NEG	POS	NEG	POS	AMB	POS	NEG	POS	POS
Riyadh-2891670	POS	AMB	POS	POS	NEG	POS	NEG	POS	POS	AMB	NEG	POS	POS
Riyadh-3006920	POS	AMB	POS	POS	NEG	POS	NEG	POS	AMB	POS	NEG	POS	POS
Riyadh-2817276-1	POS	AMB	POS	POS	NEG	POS	NEG	POS	POS	AMB	NEG	POS	POS
Riyadh-0295102	POS	AMB	POS	POS	NEG	POS	NEG	POS	POS	AMB	NEG	POS	POS
Riyadh-2820597	POS	AMB	POS	POS	NEG	POS	NEG	POS	AMB	POS	NEG	POS	POS
Riyadh-2822088	POS	AMB	POS	POS	NEG	POS	AMB	POS	POS	AMB	NEG	POS	POS
Riyadh-3010092	POS	AMB	POS	POS	NEG	POS	NEG	POS	AMB	POS	NEG	POS	POS
Riyadh-3022844	POS	AMB	POS	POS	NEG	POS	NEG	POS	AMB	POS	NEG	POS	POS
Riyadh-3108214-2	POS	NEG	POS	POS	NEG	POS	NEG	POS	AMB	POS	NEG	POS	POS
Riyadh-2823926	POS	AMB	POS	POS	NEG	POS	NEG	POS	POS	POS	NEG	POS	POS
Riyadh-1	POS	AMB	POS	POS	NEG	POS	NEG	POS	AMB	POS	NEG	POS	POS
Riyadh-2818388	POS	AMB	POS	POS	NEG	POS	NEG	POS	AMB	POS	NEG	POS	POS
Riyadh-2818316	POS	AMB	POS	POS	NEG	POS	NEG	POS	AMB	POS	NEG	POS	POS
**CC9/ST834-MRSA-(atypical SCCmec)**													
Riyadh-3103521	POS	AMB	POS	POS	NEG	POS	NEG	POS	AMB	POS	NEG	NEG	NEG
**CC22-MRSA-IV, Barnim/UK-EMRSA-15**													
Riyadh-2553359	AMB	POS	NEG	NEG	AMB	NEG	POS	POS	POS	NEG	POS	NEG	AMB
Riyadh-2571758	AMB	POS	NEG	NEG	AMB	NEG	POS	POS	POS	NEG	POS	NEG	AMB
Riyadh-3029203	POS	AMB	NEG	NEG	AMB	NEG	POS	POS	NEG	NEG	POS	NEG	AMB
Riyadh-3039785	AMB	POS	NEG	NEG	AMB	NEG	POS	POS	NEG	NEG	POS	NEG	NEG
Riyadh-3105594	AMB	POS	NEG	NEG	AMB	NEG	POS	POS	NEG	NEG	POS	NEG	AMB
Riyadh_IC_204-2	AMB	POS	NEG	NEG	POS	NEG	POS	POS	POS	NEG	POS	NEG	POS
Riyadh-3003974	AMB	POS	AMB	NEG	POS	NEG	POS	POS	AMB	NEG	POS	NEG	POS
Riyadh_IC_067	POS	AMB	NEG	NEG	POS	NEG	POS	POS	POS	NEG	POS	NEG	AMB
Riyadh-2988627	AMB	POS	NEG	NEG	AMB	NEG	POS	POS	POS	NEG	POS	NEG	AMB
Riyadh-3112581	AMB	POS	NEG	NEG	AMB	NEG	POS	POS	NEG	NEG	POS	NEG	NEG
**CC22-MRSA-IV [PVL+]**													
Riyadh-2781996-1	AMB	POS	NEG	NEG	NEG	NEG	AMB	POS	NEG	NEG	POS	NEG	NEG
Riyadh-3103432	AMB	POS	NEG	NEG	POS	NEG	POS	POS	NEG	NEG	POS	NEG	NEG
Riyadh-3026502	AMB	POS	NEG	NEG	NEG	NEG	AMB	POS	NEG	NEG	POS	NEG	NEG
Riyadh-3081378-2	AMB	POS	NEG	NEG	AMB	NEG	POS	POS	POS	NEG	POS	NEG	NEG
Riyadh_IC_185	AMB	POS	NEG	NEG	POS	NEG	POS	POS	POS	NEG	POS	NEG	POS
Riyadh_IC_204-1	POS	AMB	NEG	NEG	POS	NEG	POS	POS	POS	NEG	POS	NEG	POS
Riyadh-2559371	AMB	POS	NEG	NEG	POS	NEG	POS	POS	POS	NEG	POS	NEG	AMB
Riyadh-2753975	AMB	POS	NEG	NEG	AMB	NEG	POS	POS	POS	NEG	POS	NEG	AMB
Riyadh-2775605	AMB	POS	NEG	NEG	NEG	NEG	AMB	POS	AMB	NEG	POS	NEG	NEG
Riyadh-2781996-2	AMB	POS	NEG	NEG	NEG	NEG	AMB	POS	NEG	NEG	POS	NEG	NEG
Riyadh-2823783-2	AMB	POS	NEG	NEG	AMB	NEG	POS	POS	AMB	NEG	POS	NEG	AMB
Riyadh-2876601	POS	AMB	NEG	NEG	POS	NEG	POS	POS	NEG	NEG	POS	NEG	AMB
Riyadh-3036074	AMB	POS	NEG	NEG	POS	NEG	POS	POS	POS	NEG	POS	NEG	POS
Riyadh-3053099	AMB	POS	NEG	NEG	AMB	NEG	POS	POS	NEG	NEG	POS	NEG	NEG
Riyadh-3055366	AMB	POS	NEG	NEG	AMB	NEG	POS	POS	NEG	NEG	POS	NEG	NEG
Riyadh-3082712	AMB	POS	NEG	NEG	AMB	NEG	POS	POS	NEG	NEG	POS	NEG	NEG
Riyadh-3087502	AMB	POS	NEG	NEG	NEG	NEG	AMB	POS	NEG	NEG	POS	NEG	NEG
Riyadh-6	AMB	POS	NEG	NEG	AMB	NEG	POS	POS	POS	NEG	POS	NEG	AMB
Riyadh-7	AMB	POS	NEG	NEG	AMB	NEG	POS	POS	POS	NEG	POS	NEG	AMB
Riyadh-8	AMB	POS	NEG	NEG	AMB	NEG	POS	POS	NEG	NEG	POS	NEG	NEG
**CC30-MRSA-IV [PVL+], Southwest Pacific Clone**													
Riyadh-10	NEG	POS	NEG	NEG	POS	NEG	POS	NEG	NEG	NEG	POS	NEG	AMB
Riyadh_IC_123	AMB	POS	NEG	NEG	POS	NEG	POS	NEG	POS	NEG	POS	NEG	POS
Riyadh-3080713	NEG	POS	NEG	NEG	POS	NEG	POS	NEG	NEG	NEG	POS	NEG	AMB
Riyadh-2803856	AMB	POS	NEG	POS	POS	NEG	POS	NEG	AMB	NEG	POS	NEG	POS
Riyadh-2817276-1	AMB	POS	AMB	POS	POS	AMB	POS	NEG	POS	NEG	POS	NEG	POS
Riyadh-2817571-2	NEG	POS	NEG	POS	POS	AMB	POS	NEG	POS	NEG	POS	NEG	POS
Riyadh-3033868	NEG	POS	NEG	NEG	POS	NEG	AMB	NEG	NEG	NEG	POS	NEG	AMB
Riyadh-2550108	NEG	POS	NEG	NEG	POS	NEG	POS	NEG	NEG	NEG	POS	NEG	POS
Riyadh-3095056-2	NEG	POS	NEG	NEG	POS	NEG	POS	NEG	NEG	NEG	POS	NEG	NEG
Riyadh-2819899	NEG	POS	NEG	NEG	POS	NEG	POS	NEG	NEG	NEG	POS	NEG	POS
Riyadh-2821805	NEG	POS	NEG	AMB	POS	NEG	POS	NEG	NEG	NEG	POS	NEG	AMB
Riyadh-3013928	AMB	POS	NEG	AMB	POS	NEG	POS	NEG	NEG	NEG	POS	NEG	AMB
Riyadh-3029402	AMB	POS	NEG	AMB	POS	NEG	POS	NEG	NEG	NEG	POS	NEG	POS
**CC45/agr IV-MRSA-IV, WA MRSA-23**													
Riyadh-3081378-1	NEG	POS	NEG	NEG	NEG	NEG	AMB	NEG	NEG	NEG	POS	NEG	NEG
**CC80-MRSA-IV**													
Riyadh-3107635	POS	AMB	POS	POS	NEG	POS	NEG	POS	AMB	POS	NEG	NEG	NEG
Riyadh-2987458	POS	AMB	POS	POS	NEG	POS	NEG	POS	AMB	POS	NEG	NEG	NEG
**CC80-MRSA-IV [PVL+], European caMRSA Clone**													
Riyadh-2988048	POS	NEG	POS	POS	NEG	POS	NEG	POS	AMB	POS	NEG	NEG	NEG
Riyadh-2990585-2	POS	AMB	POS	POS	NEG	POS	NEG	POS	AMB	POS	NEG	NEG	NEG
Riyadh-2990585-1	POS	AMB	POS	POS	NEG	POS	NEG	POS	AMB	POS	NEG	NEG	NEG
Riyadh-2826033	POS	AMB	POS	POS	NEG	POS	NEG	POS	AMB	POS	NEG	NEG	NEG
Riyadh-1601562	POS	NEG	POS	POS	NEG	POS	NEG	POS	AMB	POS	NEG	NEG	NEG
Riyadh-2569940	POS	AMB	POS	POS	NEG	POS	NEG	POS	AMB	POS	AMB	NEG	NEG
Riyadh-2571692	POS	AMB	POS	POS	NEG	POS	NEG	POS	AMB	POS	AMB	NEG	NEG
Riyadh-2763029	POS	AMB	POS	POS	NEG	POS	NEG	POS	AMB	POS	NEG	NEG	NEG
Riyadh-2767090	POS	AMB	POS	POS	NEG	POS	NEG	POS	AMB	POS	NEG	NEG	NEG
Riyadh-2775130	POS	NEG	POS	POS	NEG	POS	NEG	POS	AMB	POS	NEG	NEG	NEG
Riyadh-2778256	POS	AMB	POS	POS	NEG	POS	NEG	POS	AMB	POS	NEG	NEG	NEG
Riyadh-2817505	POS	AMB	POS	POS	NEG	POS	AMB	POS	AMB	POS	AMB	NEG	NEG
Riyadh-3024912	POS	AMB	POS	POS	NEG	POS	NEG	POS	AMB	POS	NEG	NEG	NEG
Riyadh-2788690	POS	AMB	POS	POS	NEG	POS	NEG	POS	AMB	POS	NEG	NEG	NEG
Riyadh-2829034	POS	AMB	POS	POS	NEG	POS	NEG	POS	AMB	POS	NEG	NEG	NEG
Riyadh-3	POS	AMB	POS	POS	NEG	POS	NEG	POS	AMB	POS	NEG	NEG	NEG
Riyadh-5	POS	AMB	POS	POS	NEG	POS	NEG	POS	AMB	POS	NEG	NEG	NEG
Riyadh-2553167	POS	AMB	POS	POS	NEG	POS	NEG	POS	AMB	POS	NEG	NEG	NEG
Riyadh-3002592	POS	AMB	POS	POS	NEG	POS	NEG	POS	AMB	POS	NEG	NEG	NEG
**CC88-MRSA-IV [PVL+]**													
Riyadh-2736996	POS	AMB	POS	POS	NEG	POS	AMB	NEG	POS	AMB	NEG	POS	POS
Riyadh-2942396	POS	AMB	POS	POS	NEG	POS	NEG	NEG	AMB	POS	NEG	POS	POS
Riyadh-3105391	POS	AMB	POS	POS	NEG	POS	NEG	NEG	AMB	POS	NEG	POS	POS
**CC97-MRSA-V**													
Riyadh-0297622	POS	AMB	POS	POS	NEG	POS	NEG	POS	AMB	POS	NEG	NEG	AMB
Riyadh-3025471	POS	AMB	POS	POS	NEG	POS	AMB	POS	AMB	POS	NEG	NEG	AMB

	VIRULENCE : STAPHYLOCOCCAL SUPERANTIGEN/ENTEROTOXIN-LIKE GENES (SET/SSL)							
	ssl07/set1	ssl07/set1 (MRSA252)	ssl07/set1 (AF188836)	ssl08/set12_probe 1	ssl08/set12_probe 2	ssl09/set5_probe 1	ssl09/set5_probe 2	ssl09/set5 (MRSA252)
	staphylococcal superantigen-like protein 7			staphylococcal superantigen-like protein 8		staphylococcal superantigen-like protein 9		
**CC1-MRSA-IV&SCCfus, WA MRSA-1/45**								
Riyadh-3108609	POS	AMB	NEG	POS	POS	POS	POS	NEG
**CC1/ST772-MRSA-V [PVL+], "Bengal Bay Clone/WA I**								
Riyadh-2819026	POS	AMB	AMB	POS	POS	POS	POS	NEG
**CC5-MRSA-IV, Paediatric clone**								
Riyadh-2915327-1	POS	AMB	AMB	POS	POS	POS	POS	NEG
Riyadh-2915327-2	POS	AMB	NEG	POS	POS	POS	POS	NEG
Riyadh-2	POS	AMB	AMB	POS	POS	POS	POS	NEG
**CC5-MRSA-IV [PVL+], Paediatric clone**								
Riyadh-2986666	AMB	POS	AMB	POS	POS	POS	POS	NEG
Riyadh-2913335	POS	AMB	AMB	POS	POS	POS	POS	NEG
**CC5-MRSA-IVvar, "Maltese Clone"**								
Riyadh-2983654	AMB	POS	AMB	POS	POS	POS	POS	NEG
Riyadh-4	POS	AMB	AMB	POS	POS	POS	POS	NEG
Riyadh-2790233	AMB	POS	NEG	POS	POS	POS	POS	NEG
**CC5-MRSA-V**								
Riyadh-2568944	POS	AMB	AMB	POS	POS	POS	POS	NEG
**CC6-MRSA-IV, WA MRSA-51/66**								
Riyadh-2556168	POS	NEG	NEG	POS	POS	POS	POS	NEG
Riyadh-2824507	POS	NEG	NEG	POS	POS	POS	POS	NEG
Riyadh-2990831	POS	NEG	AMB	POS	POS	POS	POS	NEG
**CC8/ST239-MRSA-III, Vienna/Hungarian/Brazilian Cl**								
Riyadh-9	POS	AMB	AMB	POS	POS	POS	POS	NEG
Riyadh-3028763	POS	AMB	AMB	POS	POS	POS	POS	NEG
Riyadh-2817437	AMB	POS	AMB	POS	POS	POS	POS	NEG
Riyadh-2793706	AMB	POS	AMB	POS	POS	POS	POS	NEG
Riyadh-2818797	AMB	POS	AMB	POS	POS	POS	POS	NEG
Riyadh-2822825	AMB	POS	AMB	POS	POS	POS	POS	NEG
Riyadh-2888905	AMB	POS	AMB	POS	POS	POS	POS	NEG
Riyadh-2888915	AMB	POS	AMB	POS	POS	POS	POS	NEG
Riyadh-2567782	POS	AMB	AMB	POS	POS	POS	POS	NEG
Riyadh-2891670	AMB	POS	AMB	POS	POS	POS	POS	NEG
Riyadh-3006920	AMB	POS	AMB	POS	POS	POS	POS	NEG
Riyadh-2817276-1	AMB	POS	AMB	POS	POS	POS	POS	NEG
Riyadh-0295102	AMB	POS	AMB	POS	POS	POS	POS	NEG
Riyadh-2820597	AMB	POS	AMB	POS	POS	POS	POS	NEG
Riyadh-2822088	AMB	POS	AMB	POS	POS	POS	POS	NEG
Riyadh-3010092	POS	AMB	AMB	POS	POS	POS	POS	NEG
Riyadh-3022844	POS	AMB	AMB	POS	POS	POS	POS	NEG
Riyadh-3108214-2	POS	AMB	NEG	POS	POS	POS	POS	NEG
Riyadh-2823926	AMB	POS	AMB	POS	POS	POS	POS	NEG
Riyadh-1	POS	AMB	AMB	POS	POS	POS	POS	NEG
Riyadh-2818388	AMB	POS	AMB	POS	POS	POS	POS	NEG
Riyadh-2818316	AMB	POS	AMB	POS	POS	POS	POS	NEG
**CC9/ST834-MRSA-(atypical SCCmec)**								
Riyadh-3103521	POS	AMB	AMB	POS	POS	POS	POS	NEG
**CC22-MRSA-IV, Barnim/UK-EMRSA-15**								
Riyadh-2553359	AMB	AMB	POS	NEG	NEG	POS	POS	NEG
Riyadh-2571758	AMB	POS	AMB	NEG	NEG	POS	POS	NEG
Riyadh-3029203	AMB	POS	AMB	NEG	NEG	POS	POS	NEG
Riyadh-3039785	AMB	POS	AMB	NEG	NEG	POS	POS	NEG
Riyadh-3105594	AMB	POS	AMB	NEG	NEG	POS	POS	NEG
Riyadh_IC_204-2	AMB	POS	AMB	NEG	NEG	POS	POS	NEG
Riyadh-3003974	AMB	POS	AMB	NEG	NEG	POS	POS	NEG
Riyadh_IC_067	AMB	POS	AMB	NEG	NEG	POS	POS	NEG
Riyadh-2988627	AMB	POS	AMB	NEG	NEG	POS	POS	NEG
Riyadh-3112581	AMB	AMB	POS	NEG	NEG	POS	POS	NEG
**CC22-MRSA-IV [PVL+]**								
Riyadh-2781996-1	AMB	POS	AMB	NEG	NEG	POS	POS	NEG
Riyadh-3103432	AMB	POS	AMB	NEG	NEG	POS	POS	NEG
Riyadh-3026502	NEG	AMB	POS	NEG	NEG	POS	POS	NEG
Riyadh-3081378-2	AMB	AMB	POS	NEG	NEG	POS	POS	NEG
Riyadh_IC_185	AMB	POS	AMB	NEG	NEG	POS	POS	NEG
Riyadh_IC_204-1	AMB	POS	AMB	NEG	NEG	POS	POS	NEG
Riyadh-2559371	AMB	AMB	POS	NEG	NEG	POS	POS	NEG
Riyadh-2753975	AMB	POS	AMB	NEG	NEG	POS	POS	NEG
Riyadh-2775605	AMB	POS	AMB	NEG	NEG	POS	POS	NEG
Riyadh-2781996-2	AMB	AMB	POS	NEG	NEG	POS	POS	NEG
Riyadh-2823783-2	AMB	POS	POS	NEG	NEG	POS	POS	NEG
Riyadh-2876601	AMB	AMB	POS	NEG	NEG	POS	POS	NEG
Riyadh-3036074	AMB	POS	AMB	NEG	NEG	POS	POS	NEG
Riyadh-3053099	AMB	POS	AMB	NEG	NEG	POS	POS	NEG
Riyadh-3055366	AMB	POS	AMB	NEG	NEG	POS	POS	NEG
Riyadh-3082712	AMB	POS	AMB	NEG	NEG	POS	POS	NEG
Riyadh-3087502	NEG	AMB	POS	NEG	NEG	AMB	POS	NEG
Riyadh-6	AMB	AMB	POS	NEG	NEG	POS	POS	NEG
Riyadh-7	AMB	AMB	POS	NEG	NEG	POS	POS	NEG
Riyadh-8	AMB	AMB	POS	NEG	NEG	POS	POS	NEG
**CC30-MRSA-IV [PVL+], Southwest Pacific Clone**								
Riyadh-10	NEG	POS	AMB	NEG	NEG	NEG	NEG	POS
Riyadh_IC_123	AMB	POS	AMB	NEG	NEG	NEG	NEG	POS
Riyadh-3080713	NEG	POS	AMB	NEG	NEG	NEG	NEG	POS
Riyadh-2803856	NEG	POS	AMB	NEG	NEG	NEG	NEG	POS
Riyadh-2817276-1	AMB	POS	AMB	NEG	NEG	NEG	NEG	POS
Riyadh-2817571-2	AMB	POS	AMB	NEG	NEG	NEG	NEG	POS
Riyadh-3033868	NEG	POS	AMB	NEG	NEG	NEG	NEG	POS
Riyadh-2550108	AMB	POS	AMB	NEG	NEG	NEG	NEG	POS
Riyadh-3095056-2	NEG	POS	NEG	NEG	NEG	NEG	NEG	POS
Riyadh-2819899	NEG	POS	AMB	NEG	NEG	NEG	NEG	POS
Riyadh-2821805	NEG	POS	NEG	NEG	NEG	NEG	NEG	POS
Riyadh-3013928	NEG	POS	AMB	NEG	NEG	NEG	NEG	POS
Riyadh-3029402	NEG	POS	AMB	NEG	NEG	NEG	NEG	POS
**CC45/agr IV-MRSA-IV, WA MRSA-23**								
Riyadh-3081378-1	NEG	NEG	POS	NEG	NEG	NEG	NEG	POS
**CC80-MRSA-IV**								
Riyadh-3107635	POS	AMB	AMB	POS	POS	POS	POS	NEG
Riyadh-2987458	AMB	POS	AMB	POS	POS	POS	POS	NEG
**CC80-MRSA-IV [PVL+], European caMRSA Clone**								
Riyadh-2988048	POS	AMB	NEG	POS	POS	POS	POS	NEG
Riyadh-2990585-2	POS	AMB	NEG	POS	POS	POS	POS	NEG
Riyadh-2990585-1	AMB	POS	AMB	POS	POS	POS	POS	NEG
Riyadh-2826033	POS	AMB	AMB	POS	POS	POS	POS	NEG
Riyadh-1601562	POS	AMB	AMB	POS	POS	POS	POS	NEG
Riyadh-2569940	POS	AMB	AMB	POS	POS	POS	POS	NEG
Riyadh-2571692	POS	AMB	AMB	POS	POS	POS	POS	NEG
Riyadh-2763029	AMB	POS	AMB	POS	POS	POS	POS	NEG
Riyadh-2767090	AMB	POS	NEG	POS	POS	POS	POS	NEG
Riyadh-2775130	POS	AMB	NEG	POS	POS	POS	POS	NEG
Riyadh-2778256	AMB	POS	NEG	POS	POS	POS	POS	NEG
Riyadh-2817505	AMB	POS	AMB	POS	POS	POS	POS	NEG
Riyadh-3024912	POS	AMB	NEG	POS	POS	POS	POS	NEG
Riyadh-2788690	AMB	POS	NEG	POS	POS	POS	POS	NEG
Riyadh-2829034	POS	AMB	AMB	POS	POS	POS	POS	NEG
Riyadh-3	POS	AMB	AMB	POS	POS	POS	POS	NEG
Riyadh-5	POS	AMB	AMB	POS	POS	POS	POS	NEG
Riyadh-2553167	POS	AMB	AMB	POS	POS	POS	POS	NEG
Riyadh-3002592	AMB	POS	AMB	POS	POS	POS	POS	NEG
**CC88-MRSA-IV [PVL+]**								
Riyadh-2736996	AMB	POS	AMB	POS	POS	POS	POS	NEG
Riyadh-2942396	AMB	POS	AMB	POS	POS	POS	POS	NEG
Riyadh-3105391	POS	AMB	AMB	POS	POS	POS	POS	NEG
**CC97-MRSA-V**								
Riyadh-0297622	AMB	POS	AMB	POS	POS	POS	POS	NEG
Riyadh-3025471	POS	AMB	AMB	POS	POS	POS	POS	NEG

	VIRULENCE : STAPHYLOCOCCAL SUPERANTIGEN/ENTEROTOXIN-LIKE GENES (SET/SSL)											
	ssl10/set4	ssl10 (RF122)	ssl10/set4 (MRSA252)	ssl11/set2 (COL)	ssl11+set2(Mu50+N315)	ssl11+set2(MW2+MSSA 476)	ssl11/set2 (MRSA252)	setB3	setB3 (MRSA252)	setB2	setB2 (MRSA252)	setB1
	staphylococcal superantigen-like protein 10			staphylococcal superantigene-like protein 11				staphylococcal exotoxin-like protein, second locus				
**CC1-MRSA-IV&SCCfus, WA MRSA-1/45**												
Riyadh-3108609	POS	NEG	AMB	NEG	NEG	POS	NEG	POS	NEG	POS	NEG	POS
**CC1/ST772-MRSA-V [PVL+], "Bengal Bay Clone/WA I**												
Riyadh-2819026	POS	AMB	AMB	NEG	NEG	NEG	NEG	POS	NEG	POS	NEG	POS
**CC5-MRSA-IV, Paediatric clone**												
Riyadh-2915327-1	POS	NEG	AMB	NEG	POS	NEG	NEG	POS	NEG	POS	NEG	POS
Riyadh-2915327-2	POS	NEG	AMB	NEG	POS	NEG	NEG	POS	NEG	POS	NEG	POS
Riyadh-2	POS	AMB	AMB	NEG	POS	NEG	NEG	POS	NEG	POS	NEG	POS
**CC5-MRSA-IV [PVL+], Paediatric clone**												
Riyadh-2986666	POS	AMB	AMB	NEG	POS	NEG	NEG	POS	NEG	POS	NEG	POS
Riyadh-2913335	POS	AMB	AMB	NEG	POS	NEG	NEG	POS	NEG	POS	NEG	POS
**CC5-MRSA-IVvar, "Maltese Clone"**												
Riyadh-2983654	POS	NEG	AMB	NEG	POS	NEG	NEG	POS	NEG	POS	NEG	POS
Riyadh-4	POS	AMB	AMB	NEG	POS	NEG	NEG	POS	NEG	POS	NEG	POS
Riyadh-2790233	POS	NEG	AMB	NEG	POS	NEG	NEG	POS	NEG	POS	NEG	POS
**CC5-MRSA-V**												
Riyadh-2568944	POS	AMB	AMB	NEG	POS	NEG	NEG	POS	NEG	POS	NEG	POS
**CC6-MRSA-IV, WA MRSA-51/66**												
Riyadh-2556168	POS	AMB	AMB	NEG	POS	NEG	NEG	POS	NEG	POS	NEG	POS
Riyadh-2824507	POS	AMB	NEG	NEG	POS	NEG	NEG	POS	NEG	POS	NEG	POS
Riyadh-2990831	POS	AMB	AMB	NEG	POS	NEG	NEG	POS	NEG	POS	NEG	POS
**CC8/ST239-MRSA-III, Vienna/Hungarian/Brazilian Cl**												
Riyadh-9	POS	AMB	AMB	POS	NEG	NEG	NEG	POS	NEG	POS	NEG	POS
Riyadh-3028763	POS	AMB	AMB	POS	NEG	NEG	NEG	POS	NEG	POS	NEG	POS
Riyadh-2817437	POS	AMB	AMB	POS	NEG	NEG	NEG	POS	NEG	POS	NEG	POS
Riyadh-2793706	POS	NEG	AMB	POS	NEG	NEG	NEG	POS	NEG	POS	NEG	POS
Riyadh-2818797	POS	AMB	AMB	POS	NEG	NEG	NEG	POS	NEG	POS	NEG	POS
Riyadh-2822825	POS	AMB	AMB	POS	NEG	AMB	NEG	POS	NEG	POS	NEG	POS
Riyadh-2888905	POS	AMB	AMB	POS	NEG	NEG	NEG	POS	NEG	POS	NEG	POS
Riyadh-2888915	POS	AMB	AMB	POS	NEG	NEG	NEG	POS	NEG	POS	NEG	POS
Riyadh-2567782	POS	NEG	AMB	POS	NEG	NEG	NEG	POS	NEG	POS	NEG	POS
Riyadh-2891670	POS	AMB	AMB	POS	NEG	NEG	NEG	POS	NEG	POS	NEG	POS
Riyadh-3006920	POS	NEG	AMB	POS	NEG	NEG	NEG	POS	NEG	POS	NEG	POS
Riyadh-2817276-1	POS	AMB	AMB	POS	NEG	NEG	NEG	POS	NEG	POS	NEG	POS
Riyadh-0295102	POS	AMB	AMB	POS	NEG	NEG	NEG	POS	NEG	POS	NEG	POS
Riyadh-2820597	POS	AMB	AMB	POS	NEG	NEG	NEG	POS	NEG	POS	NEG	POS
Riyadh-2822088	POS	AMB	AMB	POS	NEG	NEG	NEG	POS	NEG	POS	NEG	POS
Riyadh-3010092	POS	NEG	AMB	POS	NEG	NEG	NEG	POS	NEG	POS	NEG	POS
Riyadh-3022844	POS	NEG	AMB	POS	NEG	NEG	NEG	POS	NEG	POS	NEG	POS
Riyadh-3108214-2	POS	NEG	AMB	POS	NEG	NEG	NEG	POS	NEG	POS	NEG	POS
Riyadh-2823926	POS	AMB	AMB	POS	NEG	NEG	NEG	POS	NEG	POS	NEG	POS
Riyadh-1	POS	AMB	AMB	POS	NEG	NEG	NEG	POS	NEG	POS	NEG	POS
Riyadh-2818388	POS	AMB	AMB	POS	NEG	NEG	NEG	POS	NEG	POS	NEG	POS
Riyadh-2818316	POS	AMB	AMB	POS	NEG	NEG	NEG	POS	NEG	POS	NEG	POS
**CC9/ST834-MRSA-(atypical SCCmec)**												
Riyadh-3103521	POS	NEG	AMB	NEG	NEG	NEG	NEG	POS	NEG	POS	NEG	POS
**CC22-MRSA-IV, Barnim/UK-EMRSA-15**												
Riyadh-2553359	POS	NEG	AMB	NEG	NEG	NEG	NEG	NEG	NEG	NEG	NEG	NEG
Riyadh-2571758	AMB	NEG	POS	NEG	#ZAHL!	NEG	NEG	NEG	NEG	NEG	NEG	AMB
Riyadh-3029203	POS	NEG	AMB	NEG	NEG	NEG	NEG	NEG	NEG	NEG	NEG	AMB
Riyadh-3039785	POS	NEG	AMB	NEG	NEG	NEG	NEG	NEG	NEG	NEG	NEG	AMB
Riyadh-3105594	POS	NEG	AMB	NEG	NEG	NEG	NEG	NEG	NEG	NEG	NEG	NEG
Riyadh_IC_204-2	POS	NEG	AMB	NEG	NEG	NEG	NEG	NEG	NEG	NEG	NEG	AMB
Riyadh-3003974	POS	NEG	AMB	NEG	NEG	NEG	NEG	NEG	NEG	NEG	NEG	POS
Riyadh_IC_067	POS	NEG	AMB	NEG	NEG	NEG	NEG	NEG	NEG	NEG	NEG	AMB
Riyadh-2988627	POS	NEG	AMB	NEG	NEG	NEG	NEG	NEG	NEG	NEG	NEG	AMB
Riyadh-3112581	POS	NEG	NEG	NEG	NEG	NEG	NEG	NEG	NEG	NEG	NEG	NEG
**CC22-MRSA-IV [PVL+]**												
Riyadh-2781996-1	POS	NEG	AMB	NEG	NEG	NEG	NEG	NEG	NEG	NEG	NEG	NEG
Riyadh-3103432	POS	NEG	AMB	NEG	NEG	NEG	NEG	NEG	NEG	NEG	NEG	AMB
Riyadh-3026502	POS	NEG	AMB	NEG	NEG	NEG	NEG	NEG	NEG	NEG	NEG	AMB
Riyadh-3081378-2	POS	NEG	AMB	NEG	NEG	NEG	NEG	NEG	NEG	NEG	NEG	AMB
Riyadh_IC_185	POS	AMB	AMB	NEG	NEG	NEG	NEG	NEG	NEG	AMB	NEG	POS
Riyadh_IC_204-1	POS	NEG	AMB	NEG	NEG	NEG	NEG	NEG	NEG	NEG	NEG	AMB
Riyadh-2559371	POS	NEG	AMB	NEG	NEG	NEG	NEG	NEG	NEG	NEG	NEG	AMB
Riyadh-2753975	POS	NEG	AMB	NEG	NEG	NEG	NEG	NEG	NEG	NEG	NEG	AMB
Riyadh-2775605	POS	NEG	AMB	NEG	NEG	NEG	NEG	NEG	NEG	NEG	NEG	NEG
Riyadh-2781996-2	POS	NEG	NEG	NEG	NEG	NEG	NEG	NEG	NEG	NEG	NEG	NEG
Riyadh-2823783-2	POS	NEG	AMB	NEG	NEG	NEG	NEG	NEG	NEG	NEG	NEG	AMB
Riyadh-2876601	POS	NEG	AMB	NEG	NEG	NEG	NEG	NEG	NEG	NEG	NEG	AMB
Riyadh-3036074	POS	NEG	AMB	NEG	NEG	NEG	NEG	NEG	NEG	NEG	NEG	AMB
Riyadh-3053099	POS	NEG	AMB	NEG	NEG	NEG	NEG	NEG	NEG	NEG	NEG	NEG
Riyadh-3055366	POS	NEG	AMB	NEG	NEG	NEG	NEG	NEG	NEG	NEG	NEG	NEG
Riyadh-3082712	POS	NEG	AMB	NEG	NEG	NEG	NEG	NEG	NEG	NEG	NEG	NEG
Riyadh-3087502	POS	NEG	AMB	NEG	NEG	NEG	NEG	NEG	NEG	NEG	NEG	NEG
Riyadh-6	POS	NEG	AMB	NEG	NEG	NEG	NEG	NEG	NEG	NEG	NEG	NEG
Riyadh-7	POS	NEG	AMB	NEG	NEG	NEG	NEG	NEG	NEG	NEG	NEG	NEG
Riyadh-8	POS	NEG	AMB	NEG	NEG	NEG	NEG	NEG	NEG	NEG	NEG	NEG
**CC30-MRSA-IV [PVL+], Southwest Pacific Clone**												
Riyadh-10	AMB	NEG	POS	NEG	NEG	NEG	POS	NEG	POS	NEG	POS	POS
Riyadh_IC_123	AMB	NEG	POS	NEG	NEG	NEG	POS	NEG	POS	NEG	POS	POS
Riyadh-3080713	POS	NEG	AMB	NEG	NEG	NEG	POS	NEG	POS	NEG	POS	POS
Riyadh-2803856	AMB	NEG	POS	NEG	NEG	NEG	POS	NEG	POS	NEG	POS	POS
Riyadh-2817276-1	AMB	NEG	POS	NEG	NEG	NEG	POS	NEG	POS	NEG	POS	POS
Riyadh-2817571-2	AMB	NEG	POS	NEG	NEG	NEG	POS	NEG	POS	NEG	POS	POS
Riyadh-3033868	POS	NEG	AMB	NEG	NEG	NEG	POS	NEG	POS	NEG	POS	POS
Riyadh-2550108	AMB	AMB	POS	NEG	NEG	NEG	POS	NEG	POS	NEG	POS	POS
Riyadh-3095056-2	POS	NEG	AMB	NEG	NEG	NEG	POS	NEG	POS	NEG	POS	POS
Riyadh-2819899	POS	NEG	AMB	NEG	NEG	NEG	POS	NEG	POS	NEG	POS	POS
Riyadh-2821805	AMB	NEG	POS	NEG	NEG	NEG	POS	NEG	POS	NEG	POS	POS
Riyadh-3013928	AMB	NEG	POS	NEG	NEG	NEG	POS	NEG	POS	NEG	POS	POS
Riyadh-3029402	POS	NEG	AMB	NEG	NEG	NEG	POS	NEG	POS	NEG	POS	POS
**CC45/agr IV-MRSA-IV, WA MRSA-23**												
Riyadh-3081378-1	AMB	NEG	POS	NEG	NEG	NEG	NEG	NEG	POS	NEG	NEG	NEG
**CC80-MRSA-IV**												
Riyadh-3107635	POS	AMB	AMB	NEG	NEG	NEG	NEG	POS	NEG	POS	NEG	POS
Riyadh-2987458	POS	NEG	AMB	NEG	NEG	NEG	NEG	POS	NEG	POS	NEG	POS
**CC80-MRSA-IV [PVL+], European caMRSA Clone**												
Riyadh-2988048	POS	NEG	AMB	NEG	NEG	NEG	NEG	POS	NEG	POS	NEG	POS
Riyadh-2990585-2	POS	NEG	AMB	NEG	NEG	NEG	NEG	POS	NEG	POS	NEG	POS
Riyadh-2990585-1	POS	NEG	AMB	NEG	NEG	NEG	NEG	POS	NEG	POS	NEG	POS
Riyadh-2826033	POS	AMB	AMB	NEG	NEG	NEG	NEG	POS	NEG	POS	NEG	POS
Riyadh-1601562	POS	NEG	AMB	NEG	NEG	NEG	NEG	POS	NEG	POS	NEG	POS
Riyadh-2569940	POS	AMB	AMB	NEG	NEG	NEG	NEG	POS	NEG	POS	NEG	POS
Riyadh-2571692	POS	AMB	AMB	NEG	NEG	NEG	NEG	POS	NEG	POS	NEG	POS
Riyadh-2763029	POS	AMB	AMB	NEG	NEG	NEG	NEG	POS	NEG	POS	NEG	POS
Riyadh-2767090	POS	AMB	AMB	NEG	NEG	NEG	NEG	POS	NEG	POS	NEG	POS
Riyadh-2775130	POS	NEG	AMB	NEG	NEG	NEG	NEG	POS	NEG	POS	NEG	POS
Riyadh-2778256	POS	AMB	AMB	NEG	NEG	NEG	NEG	POS	NEG	POS	NEG	POS
Riyadh-2817505	POS	AMB	AMB	NEG	NEG	NEG	NEG	POS	NEG	POS	NEG	POS
Riyadh-3024912	POS	NEG	AMB	NEG	NEG	NEG	NEG	POS	NEG	POS	NEG	POS
Riyadh-2788690	POS	NEG	AMB	NEG	NEG	NEG	NEG	POS	NEG	POS	NEG	POS
Riyadh-2829034	POS	AMB	AMB	NEG	NEG	NEG	NEG	POS	NEG	POS	NEG	POS
Riyadh-3	POS	AMB	AMB	NEG	NEG	NEG	NEG	POS	NEG	POS	NEG	POS
Riyadh-5	POS	AMB	AMB	NEG	NEG	NEG	NEG	POS	NEG	POS	NEG	POS
Riyadh-2553167	POS	AMB	AMB	NEG	NEG	NEG	NEG	POS	NEG	POS	NEG	POS
Riyadh-3002592	POS	AMB	AMB	NEG	NEG	NEG	NEG	POS	NEG	POS	NEG	POS
**CC88-MRSA-IV [PVL+]**												
Riyadh-2736996	POS	AMB	AMB	NEG	NEG	NEG	AMB	POS	NEG	POS	NEG	POS
Riyadh-2942396	POS	AMB	AMB	NEG	NEG	NEG	POS	POS	NEG	POS	NEG	POS
Riyadh-3105391	POS	NEG	AMB	NEG	NEG	NEG	AMB	POS	NEG	POS	NEG	POS
**CC97-MRSA-V**												
Riyadh-0297622	POS	AMB	AMB	NEG	NEG	NEG	NEG	POS	NEG	POS	NEG	POS
Riyadh-3025471	POS	NEG	AMB	NEG	NEG	POS	NEG	POS	NEG	POS	NEG	POS

	CAPSULE- AND BIOFILM-ASSOCIATED GENES						
	cap 1 (total)	cap 5 (total)	cap 8 (total)	icaA	icaC	icaD	bap
	Capsule type 1	Capsule type 5	Capsule type 8	intercellular adhesion protein A	intercellular adhesion protein C	biofilm PIA synthesis protein D	surface protein involved in biofilm formation
**CC1-MRSA-IV&SCCfus, WA MRSA-1/45**							
Riyadh-3108609	NEG	NEG	POS	POS	POS	POS	NEG
**CC1/ST772-MRSA-V [PVL+], "Bengal Bay Clone/WA I**							
Riyadh-2819026	NEG	POS	NEG	POS	POS	POS	NEG
**CC5-MRSA-IV, Paediatric clone**							
Riyadh-2915327-1	NEG	POS	NEG	POS	POS	POS	NEG
Riyadh-2915327-2	NEG	POS	NEG	POS	POS	POS	NEG
Riyadh-2	NEG	POS	NEG	POS	POS	POS	NEG
**CC5-MRSA-IV [PVL+], Paediatric clone**							
Riyadh-2986666	AMB	POS	NEG	POS	POS	POS	NEG
Riyadh-2913335	NEG	POS	NEG	POS	POS	POS	NEG
**CC5-MRSA-IVvar, "Maltese Clone"**							
Riyadh-2983654	AMB	POS	NEG	POS	POS	POS	NEG
Riyadh-4	NEG	POS	NEG	POS	POS	POS	NEG
Riyadh-2790233	NEG	POS	NEG	POS	POS	POS	NEG
**CC5-MRSA-V**							
Riyadh-2568944	NEG	POS	NEG	POS	POS	POS	NEG
**CC6-MRSA-IV, WA MRSA-51/66**							
Riyadh-2556168	NEG	NEG	POS	POS	POS	POS	NEG
Riyadh-2824507	NEG	NEG	POS	POS	POS	POS	NEG
Riyadh-2990831	AMB	NEG	POS	POS	POS	POS	NEG
**CC8/ST239-MRSA-III, Vienna/Hungarian/Brazilian Cl**							
Riyadh-9	NEG	NEG	POS	POS	POS	POS	NEG
Riyadh-3028763	NEG	NEG	POS	POS	POS	POS	NEG
Riyadh-2817437	NEG	NEG	POS	POS	POS	POS	NEG
Riyadh-2793706	NEG	NEG	POS	POS	POS	POS	NEG
Riyadh-2818797	NEG	NEG	POS	POS	POS	POS	NEG
Riyadh-2822825	NEG	NEG	POS	POS	POS	POS	NEG
Riyadh-2888905	NEG	NEG	POS	POS	POS	POS	NEG
Riyadh-2888915	NEG	NEG	POS	POS	POS	POS	NEG
Riyadh-2567782	NEG	NEG	POS	POS	POS	POS	NEG
Riyadh-2891670	NEG	NEG	POS	POS	POS	POS	NEG
Riyadh-3006920	NEG	NEG	POS	POS	POS	POS	NEG
Riyadh-2817276-1	NEG	NEG	POS	POS	POS	POS	NEG
Riyadh-0295102	NEG	NEG	POS	POS	POS	POS	NEG
Riyadh-2820597	NEG	NEG	POS	POS	POS	POS	NEG
Riyadh-2822088	NEG	NEG	POS	POS	POS	POS	NEG
Riyadh-3010092	NEG	NEG	POS	POS	POS	POS	NEG
Riyadh-3022844	NEG	NEG	POS	POS	POS	POS	NEG
Riyadh-3108214-2	NEG	NEG	POS	POS	POS	POS	NEG
Riyadh-2823926	NEG	NEG	POS	POS	POS	POS	NEG
Riyadh-1	NEG	NEG	POS	POS	POS	POS	NEG
Riyadh-2818388	NEG	NEG	POS	POS	POS	POS	NEG
Riyadh-2818316	NEG	NEG	POS	POS	POS	POS	NEG
**CC9/ST834-MRSA-(atypical SCCmec)**							
Riyadh-3103521	NEG	NEG	POS	POS	POS	POS	NEG
**CC22-MRSA-IV, Barnim/UK-EMRSA-15**							
Riyadh-2553359	NEG	POS	NEG	POS	POS	POS	NEG
Riyadh-2571758	NEG	POS	NEG	POS	POS	POS	NEG
Riyadh-3029203	NEG	POS	NEG	POS	POS	POS	NEG
Riyadh-3039785	NEG	POS	NEG	POS	POS	POS	NEG
Riyadh-3105594	NEG	POS	NEG	POS	POS	POS	NEG
Riyadh_IC_204-2	NEG	POS	NEG	POS	POS	POS	NEG
Riyadh-3003974	NEG	POS	NEG	POS	POS	POS	NEG
Riyadh_IC_067	NEG	POS	NEG	POS	POS	POS	NEG
Riyadh-2988627	NEG	POS	NEG	POS	POS	POS	NEG
Riyadh-3112581	NEG	POS	NEG	POS	POS	POS	NEG
**CC22-MRSA-IV [PVL+]**							
Riyadh-2781996-1	NEG	POS	NEG	POS	POS	POS	NEG
Riyadh-3103432	NEG	POS	NEG	POS	POS	POS	NEG
Riyadh-3026502	NEG	POS	NEG	POS	POS	POS	NEG
Riyadh-3081378-2	NEG	POS	NEG	POS	POS	POS	NEG
Riyadh_IC_185	NEG	POS	NEG	POS	POS	POS	NEG
Riyadh_IC_204-1	NEG	POS	NEG	POS	POS	POS	NEG
Riyadh-2559371	NEG	POS	NEG	POS	POS	POS	NEG
Riyadh-2753975	NEG	POS	NEG	POS	POS	POS	NEG
Riyadh-2775605	NEG	POS	NEG	POS	POS	POS	NEG
Riyadh-2781996-2	NEG	POS	NEG	POS	POS	POS	NEG
Riyadh-2823783-2	NEG	POS	NEG	POS	POS	POS	NEG
Riyadh-2876601	NEG	POS	NEG	POS	POS	POS	NEG
Riyadh-3036074	NEG	POS	NEG	POS	POS	POS	NEG
Riyadh-3053099	NEG	POS	NEG	POS	POS	POS	NEG
Riyadh-3055366	NEG	POS	NEG	POS	POS	POS	NEG
Riyadh-3082712	NEG	POS	NEG	POS	POS	POS	NEG
Riyadh-3087502	NEG	POS	NEG	POS	POS	POS	NEG
Riyadh-6	NEG	POS	NEG	POS	POS	POS	NEG
Riyadh-7	NEG	POS	NEG	POS	POS	POS	NEG
Riyadh-8	NEG	POS	NEG	POS	POS	POS	NEG
**CC30-MRSA-IV [PVL+], Southwest Pacific Clone**							
Riyadh-10	NEG	NEG	POS	POS	POS	POS	NEG
Riyadh_IC_123	NEG	NEG	POS	POS	POS	POS	NEG
Riyadh-3080713	NEG	NEG	POS	POS	POS	POS	NEG
Riyadh-2803856	NEG	NEG	POS	POS	POS	POS	NEG
Riyadh-2817276-1	NEG	NEG	POS	POS	POS	POS	NEG
Riyadh-2817571-2	NEG	NEG	POS	POS	POS	POS	NEG
Riyadh-3033868	NEG	NEG	POS	POS	POS	POS	NEG
Riyadh-2550108	NEG	NEG	POS	POS	POS	POS	NEG
Riyadh-3095056-2	NEG	NEG	POS	POS	POS	POS	NEG
Riyadh-2819899	NEG	NEG	POS	POS	POS	POS	NEG
Riyadh-2821805	NEG	NEG	POS	POS	POS	POS	NEG
Riyadh-3013928	NEG	NEG	POS	POS	POS	POS	NEG
Riyadh-3029402	NEG	NEG	POS	POS	POS	POS	NEG
**CC45/agr IV-MRSA-IV, WA MRSA-23**							
Riyadh-3081378-1	NEG	NEG	POS	POS	POS	POS	NEG
**CC80-MRSA-IV**							
Riyadh-3107635	NEG	NEG	POS	POS	POS	POS	NEG
Riyadh-2987458	NEG	NEG	POS	POS	POS	POS	NEG
**CC80-MRSA-IV [PVL+], European caMRSA Clone**							
Riyadh-2988048	NEG	NEG	POS	POS	POS	POS	NEG
Riyadh-2990585-2	NEG	NEG	POS	POS	POS	POS	NEG
Riyadh-2990585-1	NEG	NEG	POS	POS	POS	POS	NEG
Riyadh-2826033	NEG	NEG	POS	POS	POS	POS	NEG
Riyadh-1601562	NEG	NEG	POS	POS	POS	POS	NEG
Riyadh-2569940	NEG	NEG	POS	POS	POS	POS	NEG
Riyadh-2571692	NEG	NEG	POS	POS	POS	POS	NEG
Riyadh-2763029	NEG	NEG	POS	POS	POS	POS	NEG
Riyadh-2767090	NEG	NEG	POS	POS	POS	POS	NEG
Riyadh-2775130	NEG	NEG	POS	POS	POS	POS	NEG
Riyadh-2778256	NEG	NEG	POS	POS	POS	POS	NEG
Riyadh-2817505	NEG	NEG	POS	POS	POS	POS	NEG
Riyadh-3024912	NEG	NEG	POS	POS	POS	POS	NEG
Riyadh-2788690	NEG	NEG	POS	POS	POS	POS	NEG
Riyadh-2829034	NEG	NEG	POS	POS	POS	POS	NEG
Riyadh-3	NEG	NEG	POS	POS	POS	POS	NEG
Riyadh-5	NEG	NEG	POS	POS	POS	POS	NEG
Riyadh-2553167	NEG	NEG	POS	POS	POS	POS	NEG
Riyadh-3002592	NEG	NEG	POS	POS	POS	POS	NEG
**CC88-MRSA-IV [PVL+]**							
Riyadh-2736996	AMB	NEG	POS	POS	POS	POS	NEG
Riyadh-2942396	AMB	NEG	POS	POS	POS	POS	NEG
Riyadh-3105391	NEG	NEG	POS	POS	POS	POS	NEG
**CC97-MRSA-V**							
Riyadh-0297622	NEG	POS	NEG	POS	POS	POS	NEG
Riyadh-3025471	NEG	POS	NEG	POS	POS	POS	NEG

	ADHAESION FACTORS / GENES ENCODING MICROBIAL SURFACE COMPONENTS RECOGNIZING ADHESIVE MATRIX MOLECULES (MSCRAMM GENES)											
	bbp (total)	bbp (cons)	bbp(COL+MW2)	bbp(MRSA252)	bbp(Mu50)	bbp(RF122)	bbp(ST45)	clfA (total)	clfA (cons)	clfA (COL+RF122)	clfA (MRSA252)	clfA (Mu50+MW2)
	bone sialoprotein-binding protein							clumping factor A				
**CC1-MRSA-IV&SCCfus, WA MRSA-1/45**												
Riyadh-3108609	POS	POS	POS	NEG	AMB	NEG	NEG	POS	POS	NEG	NEG	POS
**CC1/ST772-MRSA-V [PVL+], "Bengal Bay Clone/WA I**												
Riyadh-2819026	POS	POS	AMB	NEG	POS	NEG	AMB	POS	POS	NEG	NEG	POS
**CC5-MRSA-IV, Paediatric clone**												
Riyadh-2915327-1	POS	POS	NEG	NEG	POS	NEG	NEG	POS	POS	NEG	NEG	POS
Riyadh-2915327-2	POS	POS	NEG	NEG	POS	NEG	NEG	POS	POS	NEG	NEG	POS
Riyadh-2	POS	POS	NEG	NEG	POS	NEG	NEG	POS	POS	NEG	NEG	POS
**CC5-MRSA-IV [PVL+], Paediatric clone**												
Riyadh-2986666	POS	POS	AMB	NEG	POS	NEG	AMB	POS	POS	NEG	NEG	POS
Riyadh-2913335	POS	POS	AMB	NEG	POS	NEG	AMB	POS	POS	NEG	NEG	POS
**CC5-MRSA-IVvar, "Maltese Clone"**												
Riyadh-2983654	POS	POS	AMB	NEG	POS	NEG	AMB	POS	POS	NEG	NEG	POS
Riyadh-4	POS	POS	NEG	NEG	POS	NEG	NEG	POS	POS	NEG	NEG	POS
Riyadh-2790233	POS	POS	AMB	NEG	POS	NEG	NEG	POS	POS	NEG	NEG	POS
**CC5-MRSA-V**												
Riyadh-2568944	POS	POS	NEG	NEG	POS	NEG	NEG	POS	POS	NEG	NEG	POS
**CC6-MRSA-IV, WA MRSA-51/66**												
Riyadh-2556168	POS	POS	POS	NEG	NEG	NEG	NEG	POS	POS	NEG	NEG	POS
Riyadh-2824507	POS	POS	POS	NEG	NEG	NEG	NEG	POS	POS	NEG	NEG	POS
Riyadh-2990831	POS	POS	POS	NEG	AMB	NEG	NEG	POS	POS	NEG	NEG	POS
**CC8/ST239-MRSA-III, Vienna/Hungarian/Brazilian Cl**												
Riyadh-9	POS	POS	POS	NEG	AMB	NEG	NEG	POS	POS	POS	AMB	AMB
Riyadh-3028763	POS	POS	POS	NEG	AMB	NEG	AMB	POS	POS	AMB	AMB	POS
Riyadh-2817437	POS	POS	POS	NEG	AMB	NEG	NEG	POS	POS	AMB	AMB	POS
Riyadh-2793706	POS	POS	POS	NEG	NEG	NEG	NEG	POS	POS	AMB	AMB	POS
Riyadh-2818797	POS	POS	POS	NEG	AMB	NEG	AMB	POS	POS	POS	AMB	AMB
Riyadh-2822825	POS	POS	POS	NEG	AMB	NEG	NEG	POS	POS	POS	AMB	AMB
Riyadh-2888905	POS	POS	POS	NEG	AMB	NEG	AMB	POS	POS	POS	AMB	AMB
Riyadh-2888915	POS	POS	POS	NEG	AMB	NEG	AMB	POS	POS	POS	AMB	AMB
Riyadh-2567782	POS	POS	POS	NEG	NEG	NEG	NEG	POS	POS	POS	AMB	AMB
Riyadh-2891670	POS	POS	POS	NEG	AMB	NEG	AMB	POS	POS	POS	AMB	AMB
Riyadh-3006920	POS	POS	POS	NEG	NEG	NEG	NEG	POS	POS	POS	AMB	AMB
Riyadh-2817276-1	POS	POS	POS	NEG	AMB	NEG	NEG	POS	POS	AMB	POS	AMB
Riyadh-0295102	POS	POS	POS	NEG	AMB	NEG	NEG	POS	POS	AMB	AMB	POS
Riyadh-2820597	POS	POS	POS	NEG	AMB	NEG	AMB	POS	POS	POS	AMB	AMB
Riyadh-2822088	POS	POS	POS	NEG	AMB	NEG	NEG	POS	POS	POS	AMB	AMB
Riyadh-3010092	POS	POS	POS	NEG	NEG	NEG	NEG	POS	POS	AMB	AMB	POS
Riyadh-3022844	POS	POS	POS	NEG	NEG	NEG	NEG	POS	POS	AMB	AMB	POS
Riyadh-3108214-2	POS	POS	POS	NEG	NEG	NEG	NEG	POS	POS	POS	AMB	AMB
Riyadh-2823926	POS	POS	POS	NEG	AMB	NEG	NEG	POS	POS	POS	AMB	POS
Riyadh-1	POS	POS	POS	NEG	AMB	NEG	NEG	POS	POS	POS	AMB	AMB
Riyadh-2818388	POS	POS	POS	NEG	AMB	NEG	AMB	POS	POS	POS	AMB	AMB
Riyadh-2818316	POS	POS	POS	NEG	AMB	NEG	AMB	NEG	NEG	NEG	NEG	NEG
**CC9/ST834-MRSA-(atypical SCCmec)**												
Riyadh-3103521	POS	POS	POS	NEG	NEG	NEG	AMB	POS	POS	POS	AMB	AMB
**CC22-MRSA-IV, Barnim/UK-EMRSA-15**												
Riyadh-2553359	POS	POS	NEG	NEG	NEG	NEG	NEG	POS	POS	NEG	NEG	POS
Riyadh-2571758	POS	POS	NEG	NEG	POS	NEG	NEG	POS	POS	NEG	NEG	POS
Riyadh-3029203	POS	POS	NEG	NEG	POS	NEG	AMB	POS	POS	NEG	NEG	POS
Riyadh-3039785	POS	POS	NEG	NEG	POS	NEG	NEG	POS	POS	NEG	NEG	POS
Riyadh-3105594	POS	POS	NEG	NEG	POS	NEG	NEG	POS	POS	NEG	NEG	POS
Riyadh_IC_204-2	POS	POS	NEG	NEG	POS	NEG	NEG	POS	POS	NEG	NEG	POS
Riyadh-3003974	POS	POS	NEG	NEG	AMB	NEG	NEG	POS	POS	NEG	NEG	POS
Riyadh_IC_067	POS	POS	NEG	NEG	POS	NEG	NEG	POS	POS	NEG	NEG	POS
Riyadh-2988627	POS	POS	NEG	NEG	NEG	NEG	NEG	POS	POS	NEG	NEG	POS
Riyadh-3112581	POS	POS	NEG	NEG	NEG	NEG	NEG	POS	POS	NEG	NEG	POS
**CC22-MRSA-IV [PVL+]**												
Riyadh-2781996-1	POS	POS	NEG	NEG	NEG	NEG	NEG	POS	POS	NEG	NEG	POS
Riyadh-3103432	POS	POS	NEG	NEG	POS	NEG	NEG	POS	POS	NEG	NEG	POS
Riyadh-3026502	POS	POS	NEG	NEG	NEG	NEG	POS	POS	POS	NEG	NEG	POS
Riyadh-3081378-2	POS	POS	NEG	NEG	NEG	NEG	NEG	POS	POS	NEG	NEG	POS
Riyadh_IC_185	POS	POS	NEG	NEG	POS	NEG	NEG	POS	POS	NEG	NEG	POS
Riyadh_IC_204-1	POS	POS	NEG	NEG	POS	NEG	NEG	POS	POS	NEG	NEG	POS
Riyadh-2559371	POS	POS	NEG	NEG	POS	NEG	NEG	POS	POS	NEG	NEG	POS
Riyadh-2753975	POS	POS	NEG	NEG	POS	NEG	NEG	POS	POS	NEG	NEG	POS
Riyadh-2775605	POS	POS	NEG	NEG	NEG	NEG	NEG	POS	POS	NEG	NEG	POS
Riyadh-2781996-2	POS	POS	NEG	NEG	NEG	NEG	NEG	POS	POS	NEG	NEG	POS
Riyadh-2823783-2	POS	POS	NEG	NEG	AMB	NEG	NEG	POS	POS	NEG	NEG	POS
Riyadh-2876601	POS	POS	NEG	NEG	POS	NEG	NEG	POS	POS	NEG	NEG	POS
Riyadh-3036074	POS	POS	NEG	NEG	POS	NEG	AMB	POS	POS	NEG	NEG	POS
Riyadh-3053099	POS	POS	NEG	NEG	POS	NEG	NEG	POS	POS	NEG	NEG	POS
Riyadh-3055366	POS	POS	NEG	NEG	POS	NEG	NEG	POS	POS	NEG	NEG	POS
Riyadh-3082712	POS	POS	NEG	NEG	POS	NEG	NEG	POS	POS	NEG	NEG	POS
Riyadh-3087502	POS	POS	NEG	NEG	NEG	NEG	NEG	POS	POS	NEG	NEG	POS
Riyadh-6	POS	POS	NEG	NEG	NEG	NEG	NEG	POS	POS	NEG	NEG	POS
Riyadh-7	POS	POS	NEG	NEG	NEG	NEG	NEG	POS	POS	NEG	NEG	POS
Riyadh-8	POS	POS	NEG	NEG	NEG	NEG	NEG	POS	POS	NEG	NEG	POS
**CC30-MRSA-IV [PVL+], Southwest Pacific Clone**												
Riyadh-10	POS	POS	NEG	POS	NEG	NEG	NEG	POS	POS	POS	AMB	NEG
Riyadh_IC_123	POS	POS	AMB	POS	NEG	NEG	NEG	POS	POS	POS	AMB	AMB
Riyadh-3080713	POS	POS	NEG	POS	NEG	NEG	NEG	POS	POS	POS	AMB	NEG
Riyadh-2803856	POS	POS	AMB	POS	NEG	NEG	NEG	POS	POS	POS	AMB	NEG
Riyadh-2817276-1	POS	POS	AMB	POS	NEG	NEG	AMB	POS	POS	POS	AMB	AMB
Riyadh-2817571-2	NEG	NEG	NEG	NEG	NEG	NEG	NEG	POS	POS	POS	AMB	AMB
Riyadh-3033868	POS	POS	NEG	POS	NEG	NEG	NEG	POS	POS	POS	AMB	NEG
Riyadh-2550108	POS	POS	NEG	POS	NEG	NEG	NEG	POS	POS	POS	AMB	NEG
Riyadh-3095056-2	POS	POS	NEG	POS	NEG	NEG	NEG	POS	POS	POS	AMB	NEG
Riyadh-2819899	POS	POS	NEG	POS	NEG	NEG	NEG	POS	POS	POS	AMB	NEG
Riyadh-2821805	POS	POS	NEG	POS	NEG	NEG	NEG	POS	POS	POS	AMB	NEG
Riyadh-3013928	POS	POS	AMB	POS	NEG	NEG	NEG	POS	POS	POS	AMB	NEG
Riyadh-3029402	POS	POS	AMB	POS	NEG	NEG	AMB	POS	POS	POS	AMB	NEG
**CC45/agr IV-MRSA-IV, WA MRSA-23**												
Riyadh-3081378-1	POS	POS	NEG	NEG	NEG	NEG	POS	POS	POS	NEG	NEG	POS
**CC80-MRSA-IV**												
Riyadh-3107635	POS	POS	NEG	NEG	POS	NEG	NEG	POS	POS	NEG	NEG	POS
Riyadh-2987458	POS	POS	AMB	NEG	POS	NEG	AMB	POS	POS	NEG	NEG	POS
**CC80-MRSA-IV [PVL+], European caMRSA Clone**												
Riyadh-2988048	POS	POS	NEG	NEG	POS	NEG	NEG	POS	POS	NEG	NEG	POS
Riyadh-2990585-2	POS	POS	NEG	NEG	POS	NEG	NEG	POS	POS	NEG	NEG	POS
Riyadh-2990585-1	POS	POS	AMB	NEG	POS	NEG	NEG	POS	POS	NEG	NEG	POS
Riyadh-2826033	POS	NEG	POS	NEG	NEG	NEG	NEG	POS	POS	NEG	NEG	POS
Riyadh-1601562	NEG	NEG	NEG	NEG	NEG	NEG	NEG	POS	POS	NEG	NEG	POS
Riyadh-2569940	POS	POS	AMB	NEG	POS	NEG	AMB	POS	POS	NEG	NEG	POS
Riyadh-2571692	POS	POS	AMB	NEG	POS	NEG	AMB	POS	POS	NEG	NEG	POS
Riyadh-2763029	POS	POS	AMB	NEG	POS	NEG	AMB	POS	POS	NEG	NEG	POS
Riyadh-2767090	POS	POS	NEG	NEG	POS	NEG	NEG	POS	POS	NEG	NEG	POS
Riyadh-2775130	POS	POS	NEG	NEG	POS	NEG	NEG	POS	POS	NEG	NEG	POS
Riyadh-2778256	POS	POS	NEG	NEG	POS	NEG	NEG	POS	POS	NEG	NEG	POS
Riyadh-2817505	POS	POS	AMB	NEG	POS	NEG	AMB	POS	POS	NEG	NEG	POS
Riyadh-3024912	POS	POS	NEG	NEG	POS	NEG	NEG	POS	POS	NEG	NEG	POS
Riyadh-2788690	POS	POS	NEG	NEG	POS	NEG	NEG	POS	POS	NEG	NEG	POS
Riyadh-2829034	POS	POS	AMB	NEG	POS	NEG	NEG	POS	POS	NEG	NEG	POS
Riyadh-3	POS	POS	NEG	NEG	POS	NEG	NEG	POS	POS	NEG	NEG	POS
Riyadh-5	POS	POS	NEG	NEG	POS	NEG	AMB	POS	POS	NEG	NEG	POS
Riyadh-2553167	POS	POS	NEG	NEG	POS	NEG	NEG	POS	POS	NEG	NEG	POS
Riyadh-3002592	POS	POS	AMB	NEG	POS	NEG	NEG	POS	POS	NEG	NEG	POS
**CC88-MRSA-IV [PVL+]**												
Riyadh-2736996	POS	POS	AMB	NEG	POS	NEG	AMB	POS	POS	NEG	NEG	POS
Riyadh-2942396	POS	POS	AMB	NEG	POS	NEG	NEG	POS	POS	NEG	NEG	POS
Riyadh-3105391	POS	POS	NEG	NEG	POS	NEG	NEG	POS	POS	NEG	NEG	POS
**CC97-MRSA-V**												
Riyadh-0297622	POS	POS	AMB	NEG	POS	NEG	NEG	POS	POS	AMB	AMB	AMB
Riyadh-3025471	POS	POS	NEG	NEG	POS	NEG	NEG	POS	POS	AMB	AMB	POS

	ADHAESION FACTORS / GENES ENCODING MICROBIAL SURFACE COMPONENTS RECOGNIZING ADHESIVE MATRIX MOLECULES (MSCRAMM GENES)												
	clfB (total)	clfB (cons)	clfB (COL+Mu50)	clfB (MW2)	clfB (RF122)	cna	ebh (cons)	ebpS (total)	ebpS_probe 612	ebpS_probe 614	ebpS (01-1111)	ebpS (COL)	eno
	clumping factor B					collagen-binding adhesin	cell wall associated fibronectin-binding protein	cell surface elastin binding protein					enolase
**CC1-MRSA-IV&SCCfus, WA MRSA-1/45**													
Riyadh-3108609	POS	POS	NEG	POS	AMB	POS	POS	POS	POS	POS	NEG	NEG	POS
**CC1/ST772-MRSA-V [PVL+], "Bengal Bay Clone/WA I**													
Riyadh-2819026	POS	POS	NEG	NEG	POS	POS	POS	POS	POS	POS	NEG	NEG	POS
**CC5-MRSA-IV, Paediatric clone**													
Riyadh-2915327-1	POS	POS	POS	NEG	NEG	NEG	POS	POS	POS	POS	NEG	NEG	POS
Riyadh-2915327-2	POS	POS	POS	NEG	NEG	NEG	POS	POS	POS	POS	NEG	NEG	POS
Riyadh-2	POS	POS	POS	NEG	NEG	NEG	POS	POS	POS	POS	NEG	NEG	POS
**CC5-MRSA-IV [PVL+], Paediatric clone**													
Riyadh-2986666	POS	POS	POS	NEG	NEG	NEG	POS	POS	POS	POS	NEG	NEG	POS
Riyadh-2913335	POS	POS	POS	NEG	NEG	NEG	POS	POS	POS	POS	NEG	NEG	POS
**CC5-MRSA-IVvar, "Maltese Clone"**													
Riyadh-2983654	POS	POS	POS	NEG	NEG	NEG	POS	POS	POS	POS	NEG	NEG	POS
Riyadh-4	POS	POS	POS	NEG	NEG	NEG	POS	POS	POS	POS	NEG	NEG	POS
Riyadh-2790233	POS	POS	POS	NEG	NEG	NEG	POS	POS	POS	POS	NEG	NEG	POS
**CC5-MRSA-V**													
Riyadh-2568944	POS	POS	POS	NEG	NEG	NEG	POS	POS	POS	POS	NEG	NEG	POS
**CC6-MRSA-IV, WA MRSA-51/66**													
Riyadh-2556168	POS	POS	NEG	NEG	POS	POS	POS	POS	POS	POS	NEG	NEG	POS
Riyadh-2824507	POS	POS	NEG	NEG	POS	POS	POS	POS	POS	POS	NEG	NEG	POS
Riyadh-2990831	POS	POS	AMB	NEG	POS	POS	POS	POS	POS	POS	NEG	NEG	POS
**CC8/ST239-MRSA-III, Vienna/Hungarian/Brazilian Cl**													
Riyadh-9	POS	POS	NEG	NEG	POS	POS	POS	POS	POS	POS	NEG	POS	POS
Riyadh-3028763	POS	POS	NEG	NEG	POS	POS	POS	POS	POS	POS	NEG	POS	POS
Riyadh-2817437	POS	POS	NEG	NEG	POS	POS	POS	POS	POS	POS	NEG	POS	POS
Riyadh-2793706	POS	POS	NEG	NEG	POS	POS	POS	POS	POS	POS	NEG	POS	POS
Riyadh-2818797	POS	POS	NEG	NEG	POS	POS	POS	POS	POS	POS	NEG	POS	POS
Riyadh-2822825	POS	POS	AMB	NEG	POS	POS	POS	POS	POS	POS	NEG	POS	POS
Riyadh-2888905	POS	POS	NEG	NEG	POS	POS	POS	POS	POS	POS	NEG	POS	POS
Riyadh-2888915	POS	POS	AMB	NEG	POS	POS	POS	POS	POS	POS	NEG	POS	POS
Riyadh-2567782	POS	POS	NEG	NEG	POS	POS	NEG	POS	POS	POS	NEG	POS	POS
Riyadh-2891670	POS	POS	NEG	NEG	POS	POS	POS	POS	POS	POS	NEG	POS	POS
Riyadh-3006920	POS	POS	NEG	NEG	POS	POS	POS	POS	POS	POS	NEG	POS	POS
Riyadh-2817276-1	POS	POS	NEG	NEG	POS	POS	POS	POS	POS	POS	NEG	POS	POS
Riyadh-0295102	POS	POS	NEG	NEG	POS	POS	POS	POS	POS	POS	NEG	POS	POS
Riyadh-2820597	POS	POS	AMB	NEG	POS	POS	POS	POS	POS	POS	NEG	POS	POS
Riyadh-2822088	POS	POS	AMB	NEG	POS	POS	POS	POS	POS	POS	NEG	POS	POS
Riyadh-3010092	POS	POS	NEG	NEG	POS	POS	POS	POS	POS	POS	NEG	POS	POS
Riyadh-3022844	POS	POS	NEG	NEG	POS	POS	POS	POS	POS	POS	NEG	POS	POS
Riyadh-3108214-2	POS	POS	NEG	NEG	POS	POS	POS	POS	POS	POS	NEG	POS	POS
Riyadh-2823926	POS	POS	NEG	NEG	POS	POS	POS	POS	POS	POS	NEG	POS	POS
Riyadh-1	POS	POS	NEG	NEG	POS	POS	POS	POS	POS	POS	NEG	POS	POS
Riyadh-2818388	POS	POS	NEG	NEG	POS	POS	POS	POS	POS	POS	NEG	POS	POS
Riyadh-2818316	POS	POS	NEG	NEG	POS	POS	POS	POS	POS	POS	NEG	POS	POS
**CC9/ST834-MRSA-(atypical SCCmec)**													
Riyadh-3103521	POS	POS	NEG	NEG	POS	NEG	POS	POS	POS	POS	NEG	NEG	POS
**CC22-MRSA-IV, Barnim/UK-EMRSA-15**													
Riyadh-2553359	POS	POS	NEG	NEG	POS	POS	NEG	POS	POS	POS	NEG	POS	POS
Riyadh-2571758	POS	POS	NEG	NEG	POS	POS	NEG	POS	POS	POS	NEG	POS	POS
Riyadh-3029203	POS	POS	NEG	NEG	POS	POS	NEG	POS	POS	POS	NEG	POS	POS
Riyadh-3039785	POS	POS	NEG	NEG	POS	POS	NEG	POS	POS	POS	NEG	POS	POS
Riyadh-3105594	POS	POS	NEG	NEG	POS	POS	NEG	POS	POS	POS	NEG	POS	POS
Riyadh_IC_204-2	POS	POS	AMB	NEG	POS	POS	NEG	POS	POS	POS	NEG	POS	POS
Riyadh-3003974	POS	POS	NEG	NEG	POS	POS	NEG	POS	POS	POS	NEG	POS	POS
Riyadh_IC_067	POS	POS	NEG	NEG	POS	POS	NEG	POS	POS	POS	NEG	POS	POS
Riyadh-2988627	POS	POS	NEG	NEG	POS	POS	NEG	POS	POS	POS	NEG	POS	POS
Riyadh-3112581	POS	POS	NEG	NEG	POS	POS	NEG	POS	POS	POS	NEG	POS	POS
**CC22-MRSA-IV [PVL+]**													
Riyadh-2781996-1	POS	POS	NEG	NEG	POS	POS	NEG	POS	POS	POS	NEG	POS	POS
Riyadh-3103432	POS	POS	NEG	NEG	POS	POS	NEG	POS	POS	POS	NEG	POS	POS
Riyadh-3026502	POS	POS	NEG	NEG	NEG	POS	NEG	POS	POS	POS	NEG	POS	POS
Riyadh-3081378-2	POS	POS	NEG	NEG	POS	POS	NEG	POS	POS	POS	NEG	POS	POS
Riyadh_IC_185	POS	POS	NEG	NEG	POS	POS	NEG	POS	POS	POS	NEG	POS	POS
Riyadh_IC_204-1	POS	POS	NEG	NEG	POS	POS	NEG	POS	POS	POS	NEG	POS	POS
Riyadh-2559371	POS	POS	NEG	NEG	POS	POS	NEG	POS	POS	POS	NEG	POS	POS
Riyadh-2753975	POS	POS	NEG	NEG	POS	POS	NEG	POS	POS	POS	NEG	POS	POS
Riyadh-2775605	POS	POS	NEG	NEG	POS	POS	NEG	POS	POS	POS	NEG	POS	POS
Riyadh-2781996-2	POS	POS	NEG	NEG	POS	POS	NEG	POS	POS	POS	NEG	POS	POS
Riyadh-2823783-2	POS	POS	NEG	NEG	POS	POS	NEG	POS	POS	POS	NEG	POS	POS
Riyadh-2876601	POS	POS	NEG	NEG	POS	POS	NEG	POS	POS	POS	NEG	POS	POS
Riyadh-3036074	POS	POS	NEG	NEG	POS	POS	NEG	POS	POS	POS	NEG	POS	POS
Riyadh-3053099	POS	POS	NEG	NEG	POS	POS	NEG	POS	POS	POS	NEG	POS	POS
Riyadh-3055366	POS	POS	NEG	NEG	POS	POS	NEG	POS	POS	POS	NEG	POS	POS
Riyadh-3082712	POS	POS	NEG	NEG	POS	POS	NEG	POS	POS	POS	NEG	POS	POS
Riyadh-3087502	POS	POS	NEG	NEG	NEG	POS	NEG	POS	POS	POS	NEG	POS	POS
Riyadh-6	POS	POS	NEG	NEG	POS	POS	NEG	POS	POS	POS	NEG	POS	POS
Riyadh-7	POS	POS	NEG	NEG	POS	POS	NEG	POS	POS	POS	NEG	POS	POS
Riyadh-8	POS	POS	NEG	NEG	POS	POS	NEG	POS	POS	POS	NEG	POS	POS
**CC30-MRSA-IV [PVL+], Southwest Pacific Clone**													
Riyadh-10	POS	POS	NEG	NEG	POS	POS	POS	POS	POS	POS	NEG	NEG	POS
Riyadh_IC_123	POS	POS	NEG	NEG	POS	POS	POS	POS	POS	POS	NEG	NEG	POS
Riyadh-3080713	POS	POS	NEG	NEG	POS	POS	POS	POS	POS	POS	NEG	NEG	POS
Riyadh-2803856	POS	POS	NEG	NEG	POS	POS	POS	POS	POS	POS	NEG	NEG	POS
Riyadh-2817276-1	POS	POS	NEG	NEG	POS	POS	POS	POS	POS	POS	NEG	NEG	POS
Riyadh-2817571-2	POS	POS	NEG	NEG	POS	POS	POS	POS	POS	POS	NEG	NEG	POS
Riyadh-3033868	POS	POS	NEG	NEG	POS	POS	POS	POS	POS	POS	NEG	NEG	POS
Riyadh-2550108	POS	POS	NEG	NEG	NEG	POS	POS	POS	POS	POS	NEG	NEG	POS
Riyadh-3095056-2	POS	POS	NEG	NEG	NEG	POS	POS	POS	POS	POS	NEG	NEG	POS
Riyadh-2819899	POS	POS	NEG	NEG	NEG	POS	POS	POS	POS	POS	NEG	NEG	POS
Riyadh-2821805	POS	POS	NEG	NEG	NEG	POS	POS	POS	POS	POS	NEG	NEG	POS
Riyadh-3013928	POS	POS	NEG	NEG	NEG	POS	POS	POS	POS	POS	NEG	NEG	POS
Riyadh-3029402	POS	POS	NEG	NEG	NEG	POS	POS	POS	POS	POS	NEG	NEG	POS
**CC45/agr IV-MRSA-IV, WA MRSA-23**													
Riyadh-3081378-1	POS	POS	NEG	POS	AMB	POS	POS	POS	NEG	POS	POS	NEG	POS
**CC80-MRSA-IV**													
Riyadh-3107635	POS	POS	POS	NEG	NEG	NEG	POS	POS	POS	POS	NEG	NEG	POS
Riyadh-2987458	POS	POS	POS	NEG	NEG	NEG	POS	POS	POS	POS	NEG	NEG	POS
**CC80-MRSA-IV [PVL+], European caMRSA Clone**													
Riyadh-2988048	POS	POS	POS	NEG	NEG	NEG	POS	POS	POS	POS	NEG	NEG	POS
Riyadh-2990585-2	POS	POS	POS	NEG	NEG	NEG	POS	POS	POS	POS	NEG	NEG	POS
Riyadh-2990585-1	POS	POS	POS	NEG	NEG	NEG	POS	POS	POS	POS	NEG	NEG	POS
Riyadh-2826033	POS	POS	POS	NEG	NEG	NEG	POS	POS	POS	POS	NEG	NEG	POS
Riyadh-1601562	POS	POS	POS	NEG	NEG	NEG	POS	POS	POS	POS	NEG	NEG	POS
Riyadh-2569940	POS	POS	POS	NEG	NEG	NEG	POS	POS	POS	POS	NEG	NEG	POS
Riyadh-2571692	POS	POS	POS	NEG	NEG	NEG	POS	POS	POS	POS	NEG	NEG	POS
Riyadh-2763029	POS	POS	POS	NEG	NEG	NEG	POS	POS	POS	POS	NEG	NEG	POS
Riyadh-2767090	POS	POS	POS	NEG	NEG	NEG	POS	POS	POS	POS	NEG	NEG	POS
Riyadh-2775130	POS	POS	POS	NEG	NEG	NEG	POS	POS	POS	POS	NEG	NEG	POS
Riyadh-2778256	POS	POS	POS	NEG	NEG	NEG	POS	POS	POS	POS	NEG	NEG	POS
Riyadh-2817505	POS	POS	POS	NEG	NEG	NEG	POS	POS	POS	POS	NEG	NEG	POS
Riyadh-3024912	POS	POS	POS	NEG	NEG	NEG	POS	POS	POS	POS	NEG	NEG	POS
Riyadh-2788690	POS	POS	POS	NEG	NEG	NEG	POS	POS	POS	POS	NEG	NEG	POS
Riyadh-2829034	POS	POS	POS	NEG	NEG	NEG	POS	POS	POS	POS	NEG	NEG	POS
Riyadh-3	POS	POS	POS	NEG	NEG	NEG	POS	POS	POS	POS	NEG	NEG	POS
Riyadh-5	POS	POS	POS	NEG	NEG	NEG	POS	POS	POS	POS	NEG	NEG	POS
Riyadh-2553167	POS	POS	POS	NEG	NEG	NEG	POS	POS	POS	POS	NEG	NEG	POS
Riyadh-3002592	POS	POS	POS	NEG	NEG	NEG	POS	POS	POS	POS	NEG	NEG	POS
**CC88-MRSA-IV [PVL+]**													
Riyadh-2736996	POS	POS	NEG	NEG	POS	NEG	POS	POS	POS	POS	NEG	POS	POS
Riyadh-2942396	POS	POS	NEG	NEG	POS	NEG	POS	POS	POS	POS	NEG	POS	POS
Riyadh-3105391	POS	POS	NEG	NEG	POS	NEG	POS	POS	POS	POS	NEG	POS	POS
**CC97-MRSA-V**													
Riyadh-0297622	POS	POS	NEG	NEG	POS	NEG	POS	POS	POS	POS	NEG	NEG	POS
Riyadh-3025471	POS	POS	NEG	NEG	POS	NEG	POS	POS	POS	POS	NEG	NEG	POS

	ADHAESION FACTORS / GENES ENCODING MICROBIAL SURFACE COMPONENTS RECOGNIZING ADHESIVE MATRIX MOLECULES (MSCRAMM GENES)							
	fib	fib (MRSA252)	fnbA (total)	fnbA (cons)	fnbA (COL)	fnbA (MRSA252)	fnbA (Mu50+MW2)	fnbA (RF122)
	fibrinogen binding protein (19 kDa)		fibronectin-binding protein A					
**CC1-MRSA-IV&SCCfus, WA MRSA-1/45**								
Riyadh-3108609	POS	NEG	POS	POS	NEG	NEG	POS	NEG
**CC1/ST772-MRSA-V [PVL+], "Bengal Bay Clone/WA [illegible]**								
Riyadh-2819026	POS	AMB	POS	POS	NEG	NEG	POS	NEG
**CC5-MRSA-IV, Paediatric clone**								
Riyadh-2915327-1	POS	NEG	POS	POS	NEG	NEG	POS	NEG
Riyadh-2915327-2	POS	NEG	POS	POS	NEG	NEG	POS	NEG
Riyadh-2	POS	NEG	POS	POS	NEG	NEG	POS	NEG
**CC5-MRSA-IV [PVL+], Paediatric clone**								
Riyadh-2986666	POS	NEG	POS	POS	NEG	NEG	POS	NEG
Riyadh-2913335	POS	NEG	POS	POS	NEG	NEG	POS	NEG
**CC5-MRSA-IVvar, "Maltese Clone"**								
Riyadh-2983654	POS	NEG	POS	POS	NEG	NEG	POS	NEG
Riyadh-4	POS	NEG	POS	POS	NEG	NEG	POS	NEG
Riyadh-2790233	POS	NEG	POS	POS	NEG	NEG	POS	NEG
**CC5-MRSA-V**								
Riyadh-2568944	POS	NEG	POS	POS	NEG	NEG	POS	NEG
**CC6-MRSA-IV, WA MRSA-51/66**								
Riyadh-2556168	POS	NEG	POS	POS	NEG	POS	NEG	NEG
Riyadh-2824507	POS	NEG	POS	POS	NEG	POS	NEG	NEG
Riyadh-2990831	POS	AMB	POS	POS	NEG	POS	NEG	NEG
**CC8/ST239-MRSA-III, Vienna/Hungarian/Brazilian Cl**								
Riyadh-9	POS	NEG	POS	POS	POS	NEG	NEG	NEG
Riyadh-3028763	POS	NEG	POS	POS	POS	NEG	NEG	NEG
Riyadh-2817437	POS	NEG	POS	POS	POS	NEG	NEG	NEG
Riyadh-2793706	POS	NEG	POS	POS	POS	NEG	NEG	NEG
Riyadh-2818797	POS	NEG	POS	POS	POS	NEG	NEG	NEG
Riyadh-2822825	POS	AMB	POS	POS	POS	AMB	NEG	NEG
Riyadh-2888905	POS	NEG	POS	POS	POS	AMB	NEG	NEG
Riyadh-2888915	POS	NEG	POS	POS	POS	AMB	NEG	NEG
Riyadh-2567782	POS	NEG	POS	POS	POS	NEG	NEG	NEG
Riyadh-2891670	POS	NEG	POS	POS	POS	AMB	NEG	NEG
Riyadh-3006920	POS	NEG	POS	POS	POS	NEG	NEG	NEG
Riyadh-2817276-1	POS	NEG	POS	POS	POS	AMB	NEG	NEG
Riyadh-0295102	POS	NEG	POS	POS	POS	NEG	NEG	NEG
Riyadh-2820597	POS	NEG	POS	POS	POS	AMB	NEG	NEG
Riyadh-2822088	POS	AMB	POS	POS	POS	AMB	NEG	NEG
Riyadh-3010092	POS	NEG	POS	POS	POS	NEG	NEG	NEG
Riyadh-3022844	POS	NEG	POS	POS	POS	NEG	NEG	NEG
Riyadh-3108214-2	POS	NEG	POS	POS	POS	NEG	NEG	NEG
Riyadh-2823926	POS	NEG	POS	POS	POS	NEG	NEG	NEG
Riyadh-1	POS	AMB	POS	POS	POS	NEG	NEG	NEG
Riyadh-2818388	POS	NEG	POS	POS	POS	NEG	NEG	NEG
Riyadh-2818316	POS	NEG	POS	POS	POS	NEG	NEG	NEG
**CC9/ST834-MRSA-(atypical SCCmec)**								
Riyadh-3103521	POS	NEG	POS	POS	NEG	POS	NEG	NEG
**CC22-MRSA-IV, Barnim/UK-EMRSA-15**								
Riyadh-2553359	NEG	POS	POS	POS	NEG	NEG	POS	NEG
Riyadh-2571758	NEG	POS	POS	POS	NEG	NEG	POS	NEG
Riyadh-3029203	NEG	POS	POS	POS	NEG	NEG	POS	NEG
Riyadh-3039785	NEG	POS	POS	POS	NEG	NEG	POS	NEG
Riyadh-3105594	NEG	POS	POS	POS	NEG	NEG	POS	NEG
Riyadh_IC_204-2	NEG	POS	POS	POS	NEG	AMB	POS	NEG
Riyadh-3003974	NEG	POS	POS	POS	NEG	NEG	POS	NEG
Riyadh_IC_067	NEG	POS	POS	POS	NEG	NEG	POS	NEG
Riyadh-2988627	NEG	POS	POS	POS	NEG	NEG	POS	NEG
Riyadh-3112581	NEG	POS	POS	POS	NEG	NEG	POS	NEG
**CC22-MRSA-IV [PVL+]**								
Riyadh-2781996-1	NEG	POS	POS	POS	NEG	NEG	POS	NEG
Riyadh-3103432	NEG	POS	POS	POS	NEG	NEG	POS	NEG
Riyadh-3026502	NEG	POS	POS	POS	NEG	NEG	POS	NEG
Riyadh-3081378-2	NEG	POS	POS	POS	NEG	NEG	POS	NEG
Riyadh_IC_185	NEG	POS	POS	POS	NEG	NEG	POS	NEG
Riyadh_IC_204-1	NEG	POS	POS	POS	NEG	AMB	POS	NEG
Riyadh-2559371	NEG	POS	POS	POS	NEG	NEG	POS	NEG
Riyadh-2753975	NEG	POS	POS	POS	NEG	NEG	POS	NEG
Riyadh-2775605	NEG	POS	POS	POS	NEG	NEG	POS	NEG
Riyadh-2781996-2	NEG	POS	POS	POS	NEG	NEG	POS	NEG
Riyadh-2823783-2	NEG	POS	POS	POS	NEG	NEG	POS	NEG
Riyadh-2876601	NEG	POS	POS	POS	NEG	NEG	POS	NEG
Riyadh-3036074	NEG	POS	POS	POS	NEG	NEG	POS	NEG
Riyadh-3053099	NEG	POS	POS	POS	NEG	NEG	POS	NEG
Riyadh-3055366	NEG	POS	POS	POS	NEG	NEG	POS	NEG
Riyadh-3082712	NEG	POS	POS	POS	NEG	NEG	POS	NEG
Riyadh-3087502	NEG	POS	POS	POS	NEG	NEG	POS	NEG
Riyadh-6	NEG	POS	POS	POS	NEG	NEG	POS	NEG
Riyadh-7	NEG	POS	POS	POS	NEG	NEG	POS	NEG
Riyadh-8	NEG	POS	POS	POS	NEG	NEG	POS	NEG
**CC30-MRSA-IV [PVL+], Southwest Pacific Clone**								
Riyadh-10	NEG	POS	POS	POS	NEG	POS	NEG	NEG
Riyadh_IC_123	NEG	POS	POS	POS	NEG	POS	NEG	NEG
Riyadh-3080713	NEG	POS	POS	POS	NEG	POS	NEG	NEG
Riyadh-2803856	NEG	POS	POS	POS	NEG	POS	NEG	NEG
Riyadh-2817276-1	NEG	POS	POS	POS	NEG	POS	NEG	NEG
Riyadh-2817571-2	NEG	POS	POS	POS	NEG	POS	NEG	NEG
Riyadh-3033868	NEG	POS	POS	POS	NEG	POS	NEG	NEG
Riyadh-2550108	NEG	POS	POS	POS	NEG	POS	NEG	NEG
Riyadh-3095056-2	NEG	POS	POS	POS	NEG	POS	NEG	NEG
Riyadh-2819899	NEG	POS	POS	POS	NEG	POS	NEG	NEG
Riyadh-2821805	NEG	POS	POS	POS	NEG	POS	NEG	NEG
Riyadh-3013928	NEG	POS	POS	POS	NEG	POS	NEG	NEG
Riyadh-3029402	NEG	POS	POS	POS	NEG	POS	NEG	NEG
**CC45/agr IV-MRSA-IV, WA MRSA-23**								
Riyadh-3081378-1	NEG	AMB	POS	POS	NEG	POS	NEG	NEG
**CC80-MRSA-IV**								
Riyadh-3107635	POS	NEG	POS	POS	NEG	NEG	NEG	NEG
Riyadh-2987458	POS	NEG	POS	POS	NEG	NEG	NEG	NEG
**CC80-MRSA-IV [PVL+], European caMRSA Clone**								
Riyadh-2988048	POS	NEG	POS	POS	NEG	NEG	NEG	NEG
Riyadh-2990585-2	POS	NEG	POS	POS	NEG	NEG	NEG	NEG
Riyadh-2990585-1	POS	NEG	POS	POS	NEG	NEG	NEG	NEG
Riyadh-2826033	POS	NEG	POS	POS	NEG	NEG	NEG	NEG
Riyadh-1601562	POS	NEG	POS	POS	NEG	NEG	NEG	NEG
Riyadh-2569940	POS	AMB	POS	POS	NEG	NEG	NEG	NEG
Riyadh-2571692	POS	AMB	POS	POS	NEG	NEG	NEG	NEG
Riyadh-2763029	POS	NEG	POS	POS	NEG	NEG	NEG	NEG
Riyadh-2767090	POS	NEG	POS	POS	NEG	NEG	NEG	NEG
Riyadh-2775130	POS	NEG	POS	POS	NEG	NEG	NEG	NEG
Riyadh-2778256	POS	NEG	POS	POS	NEG	NEG	NEG	NEG
Riyadh-2817505	POS	NEG	POS	POS	NEG	NEG	NEG	NEG
Riyadh-3024912	POS	NEG	POS	POS	NEG	NEG	NEG	NEG
Riyadh-2788690	POS	NEG	POS	POS	NEG	NEG	NEG	NEG
Riyadh-2829034	POS	NEG	POS	POS	NEG	NEG	NEG	NEG
Riyadh-3	POS	NEG	POS	POS	NEG	NEG	NEG	NEG
Riyadh-5	POS	NEG	POS	POS	NEG	NEG	NEG	NEG
Riyadh-2553167	POS	NEG	POS	POS	NEG	NEG	NEG	NEG
Riyadh-3002592	POS	NEG	POS	POS	NEG	AMB	NEG	NEG
**CC88-MRSA-IV [PVL+]**								
Riyadh-2736996	POS	NEG	POS	POS	NEG	NEG	NEG	NEG
Riyadh-2942396	POS	AMB	POS	POS	NEG	NEG	NEG	NEG
Riyadh-3105391	POS	NEG	POS	POS	NEG	NEG	NEG	NEG
**CC97-MRSA-V**								
Riyadh-0297622	POS	NEG	POS	POS	NEG	NEG	NEG	NEG
Riyadh-3025471	POS	NEG	POS	POS	NEG	NEG	NEG	NEG

	ADHAESION FACTORS / GENES ENCODING MICROBIAL SURFACE COMPONENTS RECOGNIZING ADHESIVE MATRIX MOLECULES (MSCRAMM GENES)										
	fnbB (total)	fnbB (COL)	fnbB (COL+Mu50+MW2)	fnbB (Mu50)	fnbB (MW2)	fnbB (ST15)	fnbB (ST45-2)	map (total)	map (COL)	map (MRSA252)	map (Mu50+MW2)
	fibronectin-binding protein B							Major histocompatibility complex class II analog protein (=Extracellular adherence protein, eap)			
**CC1-MRSA-IV&SCCfus, WA MRSA-1/45**											
Riyadh-3108609	POS	NEG	POS	NEG	AMB	NEG	NEG	POS	NEG	NEG	POS
**CC1/ST772-MRSA-V [PVL+], "Bengal Bay Clone/WA I**											
Riyadh-2819026	POS	NEG	AMB	AMB	NEG	POS	NEG	POS	POS	AMB	POS
**CC5-MRSA-IV, Paediatric clone**											
Riyadh-2915327-1	POS	NEG	AMB	POS	NEG	AMB	NEG	POS	POS	NEG	POS
Riyadh-2915327-2	POS	NEG	AMB	POS	NEG	AMB	NEG	POS	POS	NEG	POS
Riyadh-2	POS	NEG	AMB	POS	NEG	AMB	NEG	POS	POS	NEG	POS
**CC5-MRSA-IV [PVL+], Paediatric clone**											
Riyadh-2986666	POS	NEG	AMB	POS	NEG	AMB	NEG	POS	POS	NEG	POS
Riyadh-2913335	POS	NEG	AMB	POS	NEG	AMB	NEG	POS	POS	NEG	POS
**CC5-MRSA-IVvar, "Maltese Clone"**											
Riyadh-2983654	POS	NEG	POS	AMB	NEG	AMB	NEG	POS	POS	NEG	POS
Riyadh-4	POS	NEG	AMB	POS	NEG	AMB	NEG	POS	POS	NEG	POS
Riyadh-2790233	POS	NEG	AMB	POS	NEG	AMB	NEG	POS	POS	NEG	POS
**CC5-MRSA-V**											
Riyadh-2568944	POS	NEG	AMB	POS	NEG	AMB	NEG	POS	POS	NEG	POS
**CC6-MRSA-IV, WA MRSA-51/66**											
Riyadh-2556168	POS	NEG	AMB	POS	NEG	NEG	NEG	POS	NEG	NEG	POS
Riyadh-2824507	POS	NEG	AMB	POS	NEG	NEG	NEG	POS	NEG	NEG	POS
Riyadh-2990831	POS	NEG	AMB	POS	NEG	NEG	NEG	POS	AMB	NEG	POS
**CC8/ST239-MRSA-III, Vienna/Hungarian/Brazilian Cl**											
Riyadh-9	POS	POS	AMB	AMB	NEG	NEG	NEG	POS	POS	NEG	AMB
Riyadh-3028763	POS	POS	AMB	AMB	NEG	NEG	NEG	POS	POS	NEG	POS
Riyadh-2817437	POS	POS	AMB	AMB	NEG	NEG	NEG	POS	POS	NEG	POS
Riyadh-2793706	POS	POS	AMB	AMB	NEG	NEG	NEG	POS	POS	NEG	POS
Riyadh-2818797	POS	POS	AMB	AMB	NEG	NEG	NEG	POS	POS	NEG	POS
Riyadh-2822825	POS	POS	AMB	AMB	NEG	NEG	NEG	POS	POS	NEG	POS
Riyadh-2888905	POS	POS	AMB	AMB	NEG	NEG	NEG	POS	POS	NEG	POS
Riyadh-2888915	POS	POS	AMB	AMB	NEG	NEG	NEG	NEG	NEG	NEG	NEG
Riyadh-2567782	POS	POS	AMB	AMB	NEG	NEG	NEG	POS	POS	NEG	AMB
Riyadh-2891670	POS	POS	AMB	AMB	NEG	NEG	NEG	POS	POS	NEG	POS
Riyadh-3006920	POS	POS	AMB	AMB	NEG	NEG	NEG	POS	POS	NEG	AMB
Riyadh-2817276-1	POS	POS	AMB	AMB	NEG	NEG	NEG	POS	POS	NEG	POS
Riyadh-0295102	POS	POS	AMB	AMB	NEG	NEG	NEG	POS	POS	NEG	AMB
Riyadh-2820597	POS	POS	AMB	AMB	NEG	NEG	NEG	POS	POS	NEG	POS
Riyadh-2822088	POS	POS	AMB	AMB	NEG	NEG	NEG	POS	POS	NEG	POS
Riyadh-3010092	POS	POS	AMB	AMB	NEG	NEG	NEG	POS	POS	NEG	AMB
Riyadh-3022844	POS	POS	AMB	AMB	NEG	NEG	NEG	POS	POS	NEG	AMB
Riyadh-3108214-2	POS	POS	AMB	NEG	NEG	NEG	NEG	POS	POS	NEG	NEG
Riyadh-2823926	POS	POS	AMB	POS	NEG	NEG	NEG	POS	POS	NEG	POS
Riyadh-1	POS	POS	AMB	AMB	NEG	NEG	NEG	POS	POS	NEG	POS
Riyadh-2818388	POS	POS	AMB	AMB	NEG	NEG	NEG	POS	POS	NEG	POS
Riyadh-2818316	POS	POS	AMB	AMB	NEG	NEG	NEG	POS	POS	NEG	POS
**CC9/ST834-MRSA-(atypical SCCmec)**											
Riyadh-3103521	POS	NEG	AMB	POS	NEG	AMB	NEG	POS	NEG	NEG	POS
**CC22-MRSA-IV, Barnim/UK-EMRSA-15**											
Riyadh-2553359	NEG	NEG	NEG	NEG	NEG	NEG	NEG	POS	NEG	NEG	POS
Riyadh-2571758	NEG	NEG	NEG	NEG	NEG	NEG	NEG	POS	NEG	NEG	POS
Riyadh-3029203	NEG	NEG	NEG	NEG	NEG	NEG	NEG	POS	NEG	NEG	POS
Riyadh-3039785	NEG	NEG	NEG	NEG	NEG	NEG	NEG	POS	NEG	NEG	POS
Riyadh-3105594	NEG	NEG	NEG	NEG	NEG	NEG	NEG	POS	NEG	NEG	POS
Riyadh_IC_204-2	POS	NEG	AMB	NEG	POS	NEG	NEG	POS	NEG	NEG	POS
Riyadh-3003974	NEG	NEG	NEG	NEG	NEG	NEG	NEG	POS	NEG	NEG	POS
Riyadh_IC_067	NEG	NEG	NEG	NEG	NEG	NEG	NEG	POS	NEG	NEG	POS
Riyadh-2988627	NEG	NEG	NEG	NEG	NEG	NEG	NEG	POS	NEG	NEG	POS
Riyadh-3112581	NEG	NEG	NEG	NEG	NEG	NEG	NEG	POS	NEG	NEG	POS
**CC22-MRSA-IV [PVL+]**											
Riyadh-2781996-1	POS	NEG	AMB	NEG	POS	NEG	NEG	POS	NEG	NEG	POS
Riyadh-3103432	POS	NEG	AMB	NEG	POS	NEG	NEG	POS	NEG	NEG	POS
Riyadh-3026502	NEG	NEG	NEG	NEG	NEG	NEG	NEG	NEG	NEG	NEG	AMB
Riyadh-3081378-2	POS	NEG	AMB	NEG	POS	NEG	NEG	POS	NEG	NEG	POS
Riyadh_IC_185	POS	NEG	AMB	NEG	POS	NEG	NEG	POS	NEG	NEG	POS
Riyadh_IC_204-1	POS	NEG	AMB	NEG	POS	NEG	NEG	POS	NEG	NEG	POS
Riyadh-2559371	POS	NEG	AMB	NEG	POS	NEG	NEG	POS	NEG	NEG	POS
Riyadh-2753975	POS	NEG	AMB	NEG	POS	NEG	NEG	POS	NEG	NEG	POS
Riyadh-2775605	POS	NEG	AMB	NEG	POS	NEG	NEG	POS	NEG	NEG	POS
Riyadh-2781996-2	POS	NEG	AMB	NEG	POS	NEG	NEG	POS	NEG	NEG	POS
Riyadh-2823783-2	POS	NEG	AMB	NEG	POS	NEG	NEG	POS	NEG	NEG	POS
Riyadh-2876601	POS	NEG	POS	NEG	AMB	NEG	NEG	POS	NEG	NEG	POS
Riyadh-3036074	POS	NEG	AMB	NEG	POS	NEG	NEG	POS	NEG	NEG	POS
Riyadh-3053099	POS	NEG	POS	NEG	AMB	NEG	NEG	POS	NEG	NEG	POS
Riyadh-3055366	POS	NEG	AMB	NEG	POS	NEG	NEG	POS	NEG	NEG	POS
Riyadh-3082712	POS	NEG	AMB	NEG	POS	NEG	NEG	POS	NEG	NEG	POS
Riyadh-3087502	POS	NEG	AMB	NEG	POS	NEG	NEG	NEG	NEG	NEG	AMB
Riyadh-6	POS	NEG	AMB	NEG	POS	NEG	NEG	POS	NEG	NEG	POS
Riyadh-7	POS	NEG	AMB	NEG	POS	NEG	NEG	POS	NEG	NEG	POS
Riyadh-8	POS	NEG	AMB	NEG	POS	NEG	NEG	POS	NEG	NEG	POS
**CC30-MRSA-IV [PVL+], Southwest Pacific Clone**											
Riyadh-10	POS	NEG	AMB	POS	NEG	NEG	NEG	POS	NEG	POS	NEG
Riyadh_IC_123	POS	NEG	AMB	POS	NEG	NEG	NEG	POS	NEG	POS	NEG
Riyadh-3080713	POS	NEG	POS	AMB	NEG	NEG	NEG	POS	NEG	POS	NEG
Riyadh-2803856	POS	NEG	AMB	POS	NEG	NEG	NEG	POS	NEG	POS	NEG
Riyadh-2817276-1	POS	NEG	POS	AMB	NEG	NEG	NEG	POS	NEG	POS	AMB
Riyadh-2817571-2	POS	NEG	AMB	POS	NEG	NEG	NEG	POS	NEG	POS	NEG
Riyadh-3033868	POS	NEG	AMB	POS	NEG	NEG	NEG	POS	NEG	POS	NEG
Riyadh-2550108	POS	NEG	AMB	POS	NEG	NEG	NEG	POS	NEG	POS	NEG
Riyadh-3095056-2	POS	NEG	POS	AMB	NEG	NEG	NEG	POS	NEG	POS	NEG
Riyadh-2819899	POS	NEG	AMB	POS	NEG	NEG	NEG	POS	NEG	POS	NEG
Riyadh-2821805	POS	NEG	POS	AMB	NEG	NEG	NEG	POS	NEG	POS	NEG
Riyadh-3013928	POS	NEG	POS	AMB	NEG	NEG	NEG	POS	NEG	POS	NEG
Riyadh-3029402	POS	NEG	POS	AMB	NEG	NEG	NEG	POS	NEG	POS	NEG
**CC45/agr IV-MRSA-IV, WA MRSA-23**											
Riyadh-3081378-1	POS	NEG	AMB	NEG	NEG	NEG	POS	POS	NEG	POS	NEG
**CC80-MRSA-IV**											
Riyadh-3107635	POS	NEG	AMB	NEG	POS	NEG	NEG	POS	NEG	NEG	POS
Riyadh-2987458	POS	NEG	POS	NEG	AMB	NEG	NEG	POS	NEG	NEG	POS
**CC80-MRSA-IV [PVL+], European caMRSA Clone**											
Riyadh-2988048	POS	NEG	AMB	NEG	POS	NEG	NEG	POS	NEG	NEG	POS
Riyadh-2990585-2	POS	NEG	POS	NEG	AMB	NEG	NEG	POS	NEG	NEG	POS
Riyadh-2990585-1	POS	NEG	POS	NEG	AMB	NEG	NEG	POS	AMB	NEG	POS
Riyadh-2826033	POS	NEG	POS	NEG	AMB	NEG	NEG	POS	AMB	NEG	POS
Riyadh-1601562	POS	NEG	AMB	NEG	POS	NEG	NEG	POS	NEG	NEG	POS
Riyadh-2569940	POS	NEG	AMB	NEG	POS	NEG	NEG	POS	POS	NEG	POS
Riyadh-2571692	POS	NEG	AMB	NEG	POS	NEG	NEG	POS	POS	NEG	POS
Riyadh-2763029	POS	NEG	AMB	NEG	POS	NEG	NEG	POS	AMB	NEG	POS
Riyadh-2767090	POS	NEG	AMB	NEG	POS	NEG	NEG	POS	NEG	NEG	POS
Riyadh-2775130	POS	NEG	AMB	NEG	POS	NEG	NEG	POS	NEG	NEG	POS
Riyadh-2778256	POS	NEG	POS	NEG	AMB	NEG	NEG	POS	NEG	NEG	POS
Riyadh-2817505	POS	NEG	POS	NEG	AMB	NEG	NEG	POS	AMB	NEG	POS
Riyadh-3024912	POS	NEG	POS	NEG	AMB	NEG	NEG	POS	NEG	NEG	POS
Riyadh-2788690	POS	NEG	AMB	NEG	POS	NEG	NEG	POS	NEG	NEG	POS
Riyadh-2829034	POS	NEG	AMB	NEG	POS	NEG	NEG	POS	AMB	NEG	POS
Riyadh-3	POS	NEG	AMB	NEG	POS	NEG	NEG	POS	AMB	NEG	POS
Riyadh-5	POS	NEG	AMB	NEG	POS	NEG	NEG	POS	AMB	NEG	POS
Riyadh-2553167	POS	NEG	AMB	NEG	POS	NEG	NEG	POS	NEG	NEG	POS
Riyadh-3002592	POS	NEG	POS	NEG	AMB	NEG	NEG	POS	AMB	NEG	POS
**CC88-MRSA-IV [PVL+]**											
Riyadh-2736996	POS	NEG	AMB	POS	NEG	NEG	NEG	POS	AMB	AMB	POS
Riyadh-2942396	POS	NEG	POS	AMB	NEG	NEG	NEG	POS	AMB	NEG	POS
Riyadh-3105391	POS	NEG	AMB	POS	NEG	NEG	NEG	POS	NEG	NEG	POS
**CC97-MRSA-V**											
Riyadh-0297622	POS	NEG	AMB	POS	NEG	NEG	NEG	POS	POS	AMB	POS
Riyadh-3025471	POS	NEG	AMB	POS	NEG	NEG	NEG	POS	POS	NEG	POS

	ADHAESION FACTORS / GENES ENCODING MICROBIAL SURFACE COMPONENTS RECOGNIZING ADHESIVE MATRIX MOLECULES (MSCRAMM GENES)										
	sasG (total)	sasG (COL+Mu50)	sasG (MW2)	sasG (OtherThan MRSA252+RF122)	sdrC (total)	sdrC (cons)	sdrC (B1)	sdrC (COL)	sdrC (Mu50)	sdrC (MW2+MRSA252+RF122)	sdrC (OtherThan252+RF122)
	Staphylococcus aureus surface protein G				Ser-Asp rich fibrinogen-/bone sialoprotein-binding protein C						
**CC1-MRSA-IV&SCCfus, WA MRSA-1/45**											
Riyadh-3108609	POS	NEG	POS	POS	POS	POS	NEG	NEG	NEG	POS	POS
**CC1/ST772-MRSA-V [PVL+], "Bengal Bay Clone/WA I**											
Riyadh-2819026	POS	NEG	POS	POS	POS	POS	AMB	NEG	POS	POS	POS
**CC5-MRSA-IV, Paediatric clone**											
Riyadh-2915327-1	POS	POS	NEG	POS	POS	POS	NEG	NEG	POS	NEG	POS
Riyadh-2915327-2	POS	POS	NEG	POS	POS	POS	NEG	NEG	POS	NEG	POS
Riyadh-2	POS	POS	NEG	POS	POS	POS	NEG	NEG	POS	NEG	POS
**CC5-MRSA-IV [PVL+], Paediatric clone**											
Riyadh-2986666	POS	POS	AMB	POS	POS	POS	AMB	NEG	POS	AMB	POS
Riyadh-2913335	POS	POS	NEG	POS	POS	POS	AMB	NEG	POS	NEG	POS
**CC5-MRSA-IVvar, "Maltese Clone"**											
Riyadh-2983654	POS	POS	NEG	POS	POS	POS	AMB	NEG	POS	NEG	POS
Riyadh-4	POS	POS	NEG	POS	POS	POS	NEG	NEG	POS	NEG	POS
Riyadh-2790233	POS	POS	NEG	POS	POS	POS	AMB	NEG	POS	NEG	POS
**CC5-MRSA-V**											
Riyadh-2568944	POS	POS	NEG	POS	POS	POS	NEG	NEG	POS	NEG	POS
**CC6-MRSA-IV, WA MRSA-51/66**											
Riyadh-2556168	POS	NEG	POS	POS	POS	POS	NEG	POS	NEG	NEG	POS
Riyadh-2824507	POS	NEG	POS	POS	POS	POS	NEG	POS	NEG	NEG	POS
Riyadh-2990831	POS	NEG	POS	POS	POS	POS	AMB	POS	AMB	AMB	POS
**CC8/ST239-MRSA-III, Vienna/Hungarian/Brazilian Cl**											
Riyadh-9	POS	POS	NEG	POS	POS	POS	NEG	POS	NEG	NEG	POS
Riyadh-3028763	POS	POS	NEG	POS	POS	POS	AMB	POS	AMB	NEG	POS
Riyadh-2817437	POS	POS	NEG	POS	POS	POS	AMB	POS	AMB	NEG	POS
Riyadh-2793706	POS	POS	NEG	POS	POS	POS	AMB	POS	AMB	NEG	POS
Riyadh-2818797	POS	POS	NEG	POS	POS	POS	NEG	POS	NEG	NEG	POS
Riyadh-2822825	POS	POS	NEG	POS	POS	POS	POS	POS	AMB	AMB	POS
Riyadh-2888905	POS	POS	NEG	POS	POS	POS	AMB	POS	AMB	NEG	POS
Riyadh-2888915	POS	POS	AMB	POS	POS	POS	AMB	POS	AMB	AMB	POS
Riyadh-2567782	POS	POS	NEG	POS	POS	POS	NEG	POS	NEG	NEG	POS
Riyadh-2891670	POS	POS	NEG	POS	POS	POS	AMB	POS	AMB	NEG	POS
Riyadh-3006920	POS	POS	NEG	POS	POS	POS	AMB	POS	AMB	NEG	POS
Riyadh-2817276-1	POS	POS	NEG	POS	POS	POS	AMB	POS	AMB	NEG	POS
Riyadh-0295102	POS	POS	NEG	POS	POS	POS	NEG	POS	NEG	NEG	POS
Riyadh-2820597	POS	POS	NEG	POS	POS	POS	POS	POS	AMB	NEG	POS
Riyadh-2822088	POS	POS	NEG	POS	POS	POS	POS	POS	POS	AMB	POS
Riyadh-3010092	POS	POS	NEG	POS	POS	POS	AMB	POS	NEG	NEG	POS
Riyadh-3022844	POS	POS	NEG	POS	POS	POS	AMB	POS	AMB	NEG	POS
Riyadh-3108214-2	POS	POS	NEG	POS	POS	POS	NEG	POS	NEG	NEG	POS
Riyadh-2823926	POS	POS	NEG	POS	POS	POS	AMB	POS	AMB	NEG	POS
Riyadh-1	POS	POS	NEG	POS	POS	POS	AMB	POS	NEG	NEG	POS
Riyadh-2818388	POS	POS	NEG	POS	POS	POS	AMB	POS	AMB	NEG	POS
Riyadh-2818316	POS	POS	NEG	POS	POS	POS	AMB	POS	AMB	NEG	POS
**CC9/ST834-MRSA-(atypical SCCmec)**											
Riyadh-3103521	POS	POS	NEG	POS	POS	POS	AMB	NEG	POS	POS	POS
**CC22-MRSA-IV, Barnim/UK-EMRSA-15**											
Riyadh-2553359	POS	NEG	POS	POS	POS	POS	NEG	NEG	POS	NEG	POS
Riyadh-2571758	POS	NEG	POS	POS	POS	POS	NEG	NEG	POS	AMB	POS
Riyadh-3029203	POS	NEG	POS	POS	POS	POS	NEG	NEG	POS	NEG	POS
Riyadh-3039785	POS	NEG	POS	POS	POS	POS	NEG	NEG	POS	NEG	POS
Riyadh-3105594	POS	NEG	POS	POS	POS	POS	NEG	NEG	POS	NEG	POS
Riyadh_IC_204-2	POS	NEG	POS	POS	POS	POS	AMB	NEG	POS	AMB	POS
Riyadh-3003974	POS	NEG	POS	POS	POS	POS	NEG	NEG	POS	NEG	POS
Riyadh_IC_067	POS	NEG	POS	POS	POS	POS	NEG	NEG	POS	NEG	POS
Riyadh-2988627	POS	NEG	POS	POS	POS	POS	NEG	NEG	POS	NEG	POS
Riyadh-3112581	POS	NEG	POS	POS	POS	POS	NEG	NEG	POS	NEG	POS
**CC22-MRSA-IV [PVL+]**											
Riyadh-2781996-1	POS	NEG	POS	POS	POS	POS	NEG	NEG	POS	NEG	POS
Riyadh-3103432	POS	NEG	POS	POS	POS	POS	NEG	NEG	POS	NEG	POS
Riyadh-3026502	POS	NEG	POS	POS	POS	POS	NEG	NEG	POS	NEG	POS
Riyadh-3081378-2	POS	NEG	POS	POS	POS	POS	NEG	NEG	POS	NEG	POS
Riyadh_IC_185	POS	NEG	POS	POS	POS	POS	AMB	NEG	POS	NEG	POS
Riyadh_IC_204-1	POS	NEG	POS	POS	POS	POS	AMB	NEG	POS	NEG	POS
Riyadh-2559371	POS	NEG	POS	POS	POS	POS	AMB	NEG	POS	NEG	POS
Riyadh-2753975	POS	NEG	POS	POS	POS	POS	NEG	NEG	POS	NEG	POS
Riyadh-2775605	POS	NEG	POS	POS	POS	POS	NEG	NEG	POS	NEG	POS
Riyadh-2781996-2	POS	NEG	POS	POS	POS	POS	NEG	NEG	POS	NEG	POS
Riyadh-2823783-2	POS	NEG	POS	POS	POS	POS	NEG	NEG	POS	NEG	POS
Riyadh-2876601	POS	NEG	POS	POS	POS	POS	NEG	NEG	POS	NEG	POS
Riyadh-3036074	POS	NEG	POS	POS	POS	POS	AMB	NEG	POS	AMB	POS
Riyadh-3053099	POS	NEG	POS	POS	POS	POS	NEG	NEG	POS	NEG	POS
Riyadh-3055366	POS	NEG	POS	POS	POS	POS	NEG	NEG	POS	NEG	POS
Riyadh-3082712	POS	NEG	POS	POS	POS	POS	NEG	NEG	POS	NEG	POS
Riyadh-3087502	POS	NEG	POS	POS	POS	POS	NEG	NEG	POS	NEG	POS
Riyadh-6	POS	NEG	POS	POS	POS	POS	NEG	NEG	POS	NEG	POS
Riyadh-7	POS	NEG	POS	POS	POS	POS	NEG	NEG	POS	NEG	POS
Riyadh-8	POS	NEG	POS	POS	POS	POS	NEG	NEG	POS	NEG	POS
**CC30-MRSA-IV [PVL+], Southwest Pacific Clone**											
Riyadh-10	NEG	NEG	NEG	NEG	POS	POS	NEG	NEG	NEG	POS	NEG
Riyadh_IC_123	NEG	NEG	NEG	NEG	POS	POS	NEG	NEG	NEG	POS	NEG
Riyadh-3080713	NEG	NEG	NEG	NEG	POS	POS	NEG	NEG	NEG	POS	NEG
Riyadh-2803856	NEG	NEG	NEG	NEG	POS	POS	NEG	NEG	NEG	POS	NEG
Riyadh-2817276-1	NEG	NEG	NEG	NEG	POS	POS	NEG	NEG	NEG	POS	NEG
Riyadh-2817571-2	NEG	NEG	NEG	NEG	POS	POS	NEG	NEG	NEG	POS	NEG
Riyadh-3033868	NEG	NEG	NEG	NEG	POS	POS	NEG	NEG	NEG	AMB	NEG
Riyadh-2550108	NEG	NEG	NEG	NEG	POS	POS	NEG	NEG	NEG	POS	NEG
Riyadh-3095056-2	NEG	NEG	NEG	NEG	POS	POS	NEG	NEG	NEG	AMB	NEG
Riyadh-2819899	NEG	NEG	NEG	NEG	POS	POS	NEG	NEG	NEG	POS	NEG
Riyadh-2821805	NEG	NEG	NEG	NEG	POS	POS	NEG	NEG	NEG	NEG	NEG
Riyadh-3013928	NEG	NEG	NEG	NEG	POS	POS	NEG	NEG	NEG	AMB	NEG
Riyadh-3029402	NEG	NEG	NEG	NEG	POS	POS	NEG	NEG	AMB	POS	NEG
**CC45/agr IV-MRSA-IV, WA MRSA-23**											
Riyadh-3081378-1	POS	NEG	POS	POS	POS	POS	POS	NEG	NEG	AMB	NEG
**CC80-MRSA-IV**											
Riyadh-3107635	POS	NEG	POS	POS	POS	POS	NEG	POS	NEG	NEG	POS
Riyadh-2987458	POS	NEG	POS	POS	POS	POS	NEG	POS	NEG	NEG	POS
**CC80-MRSA-IV [PVL+], European caMRSA Clone**											
Riyadh-2988048	POS	NEG	POS	POS	POS	POS	NEG	POS	NEG	NEG	POS
Riyadh-2990585-2	POS	NEG	POS	POS	POS	POS	NEG	POS	NEG	NEG	POS
Riyadh-2990585-1	POS	NEG	POS	POS	POS	POS	NEG	POS	NEG	NEG	POS
Riyadh-2826033	POS	NEG	POS	POS	POS	POS	AMB	NEG	NEG	NEG	POS
Riyadh-1601562	POS	NEG	POS	POS	POS	POS	NEG	NEG	NEG	NEG	POS
Riyadh-2569940	POS	NEG	POS	POS	POS	POS	NEG	POS	NEG	NEG	POS
Riyadh-2571692	POS	NEG	POS	POS	POS	POS	AMB	POS	NEG	NEG	POS
Riyadh-2763029	POS	NEG	POS	POS	POS	POS	NEG	POS	NEG	NEG	POS
Riyadh-2767090	POS	NEG	POS	POS	POS	POS	NEG	POS	NEG	NEG	POS
Riyadh-2775130	POS	NEG	POS	POS	POS	POS	NEG	POS	NEG	NEG	POS
Riyadh-2778256	POS	NEG	POS	POS	POS	POS	NEG	POS	NEG	NEG	POS
Riyadh-2817505	POS	NEG	POS	POS	POS	POS	AMB	POS	NEG	NEG	POS
Riyadh-3024912	POS	NEG	POS	POS	POS	POS	NEG	POS	NEG	NEG	POS
Riyadh-2788690	POS	NEG	POS	POS	POS	POS	NEG	POS	NEG	NEG	POS
Riyadh-2829034	POS	NEG	POS	POS	POS	POS	AMB	POS	NEG	NEG	POS
Riyadh-3	POS	NEG	POS	POS	POS	POS	NEG	POS	NEG	NEG	POS
Riyadh-5	POS	NEG	POS	POS	POS	POS	NEG	POS	NEG	NEG	POS
Riyadh-2553167	POS	NEG	POS	POS	POS	POS	NEG	POS	NEG	NEG	POS
Riyadh-3002592	POS	NEG	POS	POS	POS	POS	AMB	POS	NEG	NEG	POS
**CC88-MRSA-IV [PVL+]**											
Riyadh-2736996	POS	NEG	POS	POS	POS	POS	NEG	NEG	POS	POS	POS
Riyadh-2942396	POS	NEG	POS	POS	POS	POS	AMB	NEG	POS	POS	POS
Riyadh-3105391	POS	NEG	POS	POS	POS	POS	NEG	NEG	POS	POS	POS
**CC97-MRSA-V**											
Riyadh-0297622	POS	POS	NEG	POS	POS	POS	NEG	NEG	POS	POS	POS
Riyadh-3025471	POS	POS	NEG	POS	POS	POS	NEG	NEG	POS	POS	POS

	ADHAESION FACTORS / GENES ENCODING MICROBIAL SURFACE COMPONENTS RECOGNIZING ADHESIVE MATRIX MOLECULES (MSCRAMM GENES)										
	sdrD (total)	sdrD (cons)	sdrD (COL+MW2)	sdrD (Mu50)	sdrD (other)	vwb (total)	vwb (cons)	vwb (COL+MW2)	vwb (MRSA252)	vwb (Mu50)	vwb (RF122)
	Ser-Asp rich fibrinogen-/bone sialoprotein-binding protein D					van Willebrand factor binding protein					
**CC1-MRSA-IV&SCCfus, WA MRSA-1/45**											
Riyadh-3108609	POS	POS	POS	NEG	NEG	POS	POS	POS	NEG	NEG	NEG
**CC1/ST772-MRSA-V [PVL+], "Bengal Bay Clone/WA I**											
Riyadh-2819026	POS	POS	NEG	NEG	POS	POS	POS	NEG	NEG	NEG	NEG
**CC5-MRSA-IV, Paediatric clone**											
Riyadh-2915327-1	POS	POS	NEG	POS	NEG	POS	POS	NEG	NEG	POS	NEG
Riyadh-2915327-2	POS	POS	NEG	POS	NEG	POS	POS	NEG	NEG	POS	NEG
Riyadh-2	POS	POS	NEG	POS	NEG	POS	POS	NEG	NEG	POS	NEG
**CC5-MRSA-IV [PVL+], Paediatric clone**											
Riyadh-2986666	POS	POS	NEG	POS	NEG	POS	POS	NEG	NEG	POS	NEG
Riyadh-2913335	POS	POS	NEG	POS	NEG	POS	POS	NEG	NEG	POS	NEG
**CC5-MRSA-IVvar, "Maltese Clone"**											
Riyadh-2983654	POS	POS	NEG	POS	NEG	POS	POS	NEG	NEG	POS	NEG
Riyadh-4	POS	POS	NEG	POS	NEG	POS	POS	NEG	NEG	POS	NEG
Riyadh-2790233	POS	POS	NEG	POS	NEG	POS	POS	NEG	NEG	POS	NEG
**CC5-MRSA-V**											
Riyadh-2568944	POS	POS	NEG	POS	NEG	POS	POS	NEG	NEG	POS	NEG
**CC6-MRSA-IV, WA MRSA-51/66**											
Riyadh-2556168	POS	POS	NEG	POS	NEG	POS	POS	NEG	POS	NEG	NEG
Riyadh-2824507	POS	POS	NEG	POS	NEG	POS	POS	NEG	POS	NEG	NEG
Riyadh-2990831	POS	POS	NEG	POS	NEG	POS	POS	NEG	POS	NEG	NEG
**CC8/ST239-MRSA-III, Vienna/Hungarian/Brazilian Cl**											
Riyadh-9	POS	POS	POS	NEG	NEG	POS	POS	POS	NEG	NEG	NEG
Riyadh-3028763	POS	POS	POS	NEG	NEG	POS	POS	POS	NEG	NEG	NEG
Riyadh-2817437	POS	POS	POS	NEG	NEG	POS	POS	POS	NEG	NEG	NEG
Riyadh-2793706	POS	POS	POS	NEG	NEG	POS	POS	POS	NEG	NEG	NEG
Riyadh-2818797	POS	POS	POS	NEG	NEG	POS	POS	POS	NEG	NEG	NEG
Riyadh-2822825	POS	POS	POS	NEG	NEG	POS	POS	POS	NEG	NEG	NEG
Riyadh-2888905	POS	POS	POS	NEG	NEG	POS	POS	POS	NEG	NEG	NEG
Riyadh-2888915	POS	POS	POS	NEG	NEG	POS	POS	POS	NEG	NEG	NEG
Riyadh-2567782	POS	POS	POS	NEG	NEG	POS	POS	POS	NEG	NEG	NEG
Riyadh-2891670	POS	POS	POS	NEG	NEG	POS	POS	POS	NEG	NEG	NEG
Riyadh-3006920	POS	POS	POS	NEG	NEG	POS	POS	POS	NEG	NEG	NEG
Riyadh-2817276-1	POS	POS	POS	NEG	NEG	POS	POS	POS	NEG	NEG	NEG
Riyadh-0295102	POS	POS	POS	NEG	NEG	POS	POS	POS	NEG	NEG	NEG
Riyadh-2820597	POS	POS	POS	NEG	NEG	POS	POS	POS	NEG	NEG	NEG
Riyadh-2822088	POS	POS	POS	NEG	NEG	POS	POS	POS	NEG	NEG	NEG
Riyadh-3010092	POS	POS	POS	NEG	NEG	POS	POS	POS	NEG	NEG	NEG
Riyadh-3022844	POS	POS	POS	NEG	NEG	POS	POS	POS	NEG	NEG	NEG
Riyadh-3108214-2	POS	POS	POS	NEG	NEG	POS	POS	POS	NEG	NEG	NEG
Riyadh-2823926	POS	POS	POS	NEG	NEG	POS	POS	POS	NEG	NEG	NEG
Riyadh-1	POS	POS	POS	NEG	NEG	POS	POS	POS	NEG	NEG	NEG
Riyadh-2818388	POS	POS	POS	NEG	NEG	POS	POS	POS	NEG	NEG	NEG
Riyadh-2818316	POS	POS	POS	NEG	NEG	NEG	NEG	NEG	NEG	NEG	NEG
**CC9/ST834-MRSA-(atypical SCCmec)**											
Riyadh-3103521	POS	POS	NEG	NEG	POS	POS	POS	POS	NEG	NEG	NEG
**CC22-MRSA-IV, Barnim/UK-EMRSA-15**											
Riyadh-2553359	POS	POS	NEG	NEG	POS	POS	POS	NEG	NEG	NEG	POS
Riyadh-2571758	POS	POS	NEG	NEG	POS	POS	POS	NEG	NEG	NEG	POS
Riyadh-3029203	POS	POS	NEG	NEG	POS	POS	POS	NEG	NEG	NEG	POS
Riyadh-3039785	POS	POS	NEG	NEG	POS	POS	POS	NEG	NEG	NEG	POS
Riyadh-3105594	POS	POS	NEG	NEG	POS	POS	POS	NEG	NEG	NEG	POS
Riyadh_IC_204-2	POS	POS	NEG	NEG	POS	POS	POS	NEG	NEG	NEG	POS
Riyadh-3003974	POS	POS	NEG	NEG	POS	POS	POS	NEG	NEG	NEG	POS
Riyadh_IC_067	POS	POS	NEG	NEG	POS	POS	POS	NEG	NEG	NEG	POS
Riyadh-2988627	POS	POS	NEG	NEG	POS	POS	POS	NEG	NEG	NEG	POS
Riyadh-3112581	POS	POS	NEG	NEG	POS	POS	POS	NEG	NEG	NEG	POS
**CC22-MRSA-IV [PVL+]**											
Riyadh-2781996-1	POS	POS	NEG	NEG	POS	POS	POS	NEG	NEG	NEG	POS
Riyadh-3103432	POS	POS	NEG	NEG	POS	POS	POS	NEG	NEG	NEG	POS
Riyadh-3026502	POS	POS	NEG	NEG	POS	POS	POS	NEG	NEG	NEG	POS
Riyadh-3081378-2	POS	POS	NEG	NEG	POS	POS	POS	NEG	NEG	NEG	POS
Riyadh_IC_185	POS	POS	NEG	NEG	POS	POS	POS	NEG	NEG	NEG	POS
Riyadh_IC_204-1	POS	POS	NEG	NEG	POS	POS	POS	NEG	NEG	NEG	POS
Riyadh-2559371	POS	POS	NEG	NEG	POS	POS	POS	NEG	NEG	NEG	POS
Riyadh-2753975	POS	POS	NEG	NEG	POS	POS	POS	NEG	NEG	NEG	POS
Riyadh-2775605	POS	POS	NEG	NEG	POS	POS	POS	NEG	NEG	NEG	POS
Riyadh-2781996-2	POS	POS	NEG	NEG	POS	POS	POS	NEG	NEG	NEG	POS
Riyadh-2823783-2	POS	POS	NEG	NEG	POS	POS	POS	NEG	NEG	NEG	POS
Riyadh-2876601	POS	POS	NEG	NEG	POS	POS	POS	NEG	NEG	NEG	POS
Riyadh-3036074	POS	POS	NEG	NEG	POS	POS	POS	NEG	NEG	NEG	POS
Riyadh-3053099	POS	POS	NEG	NEG	POS	POS	POS	NEG	NEG	NEG	POS
Riyadh-3055366	POS	POS	NEG	NEG	POS	POS	POS	NEG	NEG	NEG	POS
Riyadh-3082712	POS	POS	NEG	NEG	POS	POS	POS	NEG	NEG	NEG	POS
Riyadh-3087502	POS	POS	NEG	NEG	POS	POS	POS	NEG	NEG	NEG	POS
Riyadh-6	POS	POS	NEG	NEG	POS	POS	POS	NEG	NEG	NEG	POS
Riyadh-7	POS	POS	NEG	NEG	POS	POS	POS	NEG	NEG	NEG	POS
Riyadh-8	POS	POS	NEG	NEG	POS	POS	POS	NEG	NEG	NEG	POS
**CC30-MRSA-IV [PVL+], Southwest Pacific Clone**											
Riyadh-10	POS	POS	NEG	NEG	POS	POS	POS	NEG	POS	NEG	NEG
Riyadh_IC_123	POS	POS	NEG	NEG	POS	POS	POS	NEG	POS	NEG	NEG
Riyadh-3080713	POS	POS	NEG	NEG	POS	POS	POS	NEG	POS	NEG	NEG
Riyadh-2803856	POS	POS	NEG	NEG	POS	POS	POS	NEG	POS	NEG	NEG
Riyadh-2817276-1	POS	POS	NEG	NEG	POS	POS	POS	NEG	POS	NEG	NEG
Riyadh-2817571-2	POS	POS	NEG	NEG	POS	POS	POS	NEG	POS	NEG	NEG
Riyadh-3033868	POS	POS	NEG	NEG	POS	POS	POS	NEG	POS	NEG	NEG
Riyadh-2550108	POS	POS	NEG	NEG	POS	POS	POS	NEG	POS	NEG	NEG
Riyadh-3095056-2	POS	POS	NEG	NEG	POS	POS	POS	NEG	POS	NEG	NEG
Riyadh-2819899	POS	POS	NEG	NEG	POS	POS	POS	NEG	POS	NEG	NEG
Riyadh-2821805	POS	POS	NEG	NEG	POS	POS	POS	NEG	POS	NEG	NEG
Riyadh-3013928	POS	POS	NEG	NEG	POS	POS	POS	NEG	POS	NEG	NEG
Riyadh-3029402	POS	POS	NEG	NEG	POS	POS	POS	NEG	POS	NEG	NEG
**CC45/agr IV-MRSA-IV, WA MRSA-23**											
Riyadh-3081378-1	POS	POS	NEG	NEG	POS	POS	POS	NEG	NEG	NEG	NEG
**CC80-MRSA-IV**											
Riyadh-3107635	POS	POS	NEG	NEG	POS	POS	POS	NEG	POS	NEG	NEG
Riyadh-2987458	POS	POS	NEG	NEG	POS	POS	POS	NEG	POS	NEG	NEG
**CC80-MRSA-IV [PVL+], European caMRSA Clone**											
Riyadh-2988048	POS	POS	NEG	NEG	POS	POS	POS	NEG	POS	NEG	NEG
Riyadh-2990585-2	POS	POS	NEG	NEG	POS	POS	POS	NEG	POS	NEG	NEG
Riyadh-2990585-1	POS	POS	NEG	NEG	POS	POS	POS	NEG	POS	NEG	NEG
Riyadh-2826033	NEG	NEG	NEG	NEG	NEG	POS	POS	NEG	POS	NEG	NEG
Riyadh-1601562	NEG	NEG	NEG	NEG	NEG	POS	POS	NEG	POS	NEG	NEG
Riyadh-2569940	POS	POS	NEG	NEG	POS	POS	POS	NEG	POS	NEG	NEG
Riyadh-2571692	POS	POS	NEG	NEG	POS	POS	POS	NEG	POS	NEG	NEG
Riyadh-2763029	POS	POS	NEG	NEG	POS	POS	POS	NEG	POS	NEG	NEG
Riyadh-2767090	POS	POS	NEG	NEG	POS	POS	POS	NEG	POS	NEG	NEG
Riyadh-2775130	POS	POS	NEG	NEG	POS	POS	POS	NEG	POS	NEG	NEG
Riyadh-2778256	POS	POS	NEG	NEG	POS	POS	POS	NEG	POS	NEG	NEG
Riyadh-2817505	POS	POS	NEG	NEG	POS	POS	POS	NEG	POS	NEG	NEG
Riyadh-3024912	POS	POS	NEG	NEG	POS	POS	POS	NEG	POS	NEG	NEG
Riyadh-2788690	POS	POS	NEG	NEG	POS	POS	POS	NEG	POS	NEG	NEG
Riyadh-2829034	POS	POS	NEG	NEG	POS	POS	POS	NEG	POS	NEG	NEG
Riyadh-3	POS	POS	NEG	NEG	POS	POS	POS	NEG	POS	NEG	NEG
Riyadh-5	POS	POS	NEG	NEG	POS	POS	POS	NEG	POS	NEG	NEG
Riyadh-2553167	POS	POS	NEG	NEG	POS	POS	POS	NEG	POS	NEG	NEG
Riyadh-3002592	POS	POS	NEG	NEG	POS	POS	POS	NEG	POS	NEG	NEG
**CC88-MRSA-IV [PVL+]**											
Riyadh-2736996	POS	POS	NEG	NEG	POS	POS	POS	NEG	NEG	POS	NEG
Riyadh-2942396	POS	POS	NEG	NEG	POS	POS	POS	NEG	NEG	POS	NEG
Riyadh-3105391	POS	POS	NEG	NEG	POS	POS	POS	NEG	NEG	POS	NEG
**CC97-MRSA-V**											
Riyadh-0297622	POS	POS	NEG	POS	NEG	POS	POS	NEG	NEG	NEG	POS
Riyadh-3025471	POS	POS	NEG	POS	NEG	POS	POS	NEG	NEG	NEG	POS

	IMMUNODOMINANT ANTIGEN B		DEFENSIN RESIST.		TRANSFERRIN BINDING PROTEIN			PUTATIVE TRANSPORTER			
	isaB	isaB (MRSA252)	mprF (COL+MW2)	mprF (Mu50+252)	isdA (cons)	isdA (MRSA252)	isdA (Other Than MRSA252)	lmrP (OtherThanR F122)	lmrP (OtherThanR F122)	lmrP (RF122)	lmrP (RF122)
	immunodominant antigen B		defensin resistance protein		transferrin-binding protein			hypothetical protein, similar to integral membrane protein LmrP			
**CC1-MRSA-IV&SCCfus, WA MRSA-1/45**											
Riyadh-3108609	POS	AMB	POS	AMB	POS	AMB	POS	POS	POS	NEG	NEG
**CC1/ST772-MRSA-V [PVL+], "Bengal Bay Clone/WA I**											
Riyadh-2819026	POS	AMB	POS	AMB	POS	AMB	POS	POS	POS	NEG	NEG
**CC5-MRSA-IV, Paediatric clone**											
Riyadh-2915327-1	POS	AMB	AMB	POS	POS	NEG	POS	POS	POS	NEG	NEG
Riyadh-2915327-2	POS	AMB	AMB	POS	POS	NEG	POS	POS	POS	NEG	NEG
Riyadh-2	POS	AMB	AMB	POS	POS	AMB	POS	POS	POS	NEG	NEG
**CC5-MRSA-IV [PVL+], Paediatric clone**											
Riyadh-2986666	POS	AMB	POS	AMB	POS	AMB	POS	POS	POS	NEG	NEG
Riyadh-2913335	POS	AMB	POS	AMB	POS	AMB	POS	POS	POS	NEG	NEG
**CC5-MRSA-IVvar, "Maltese Clone"**											
Riyadh-2983654	POS	AMB	POS	AMB	POS	AMB	POS	POS	POS	NEG	NEG
Riyadh-4	POS	AMB	POS	AMB	POS	AMB	POS	POS	POS	NEG	NEG
Riyadh-2790233	POS	AMB	POS	AMB	POS	NEG	POS	POS	POS	NEG	NEG
**CC5-MRSA-V**											
Riyadh-2568944	POS	AMB	AMB	POS	POS	AMB	POS	POS	POS	NEG	NEG
**CC6-MRSA-IV, WA MRSA-51/66**											
Riyadh-2556168	POS	AMB	POS	AMB	POS	AMB	POS	POS	POS	NEG	NEG
Riyadh-2824507	POS	AMB	POS	AMB	POS	NEG	POS	POS	POS	NEG	NEG
Riyadh-2990831	POS	AMB	POS	AMB	POS	AMB	POS	POS	POS	NEG	NEG
**CC8/ST239-MRSA-III, Vienna/Hungarian/Brazilian Cl**											
Riyadh-9	AMB	POS	POS	AMB	POS	AMB	POS	POS	POS	NEG	NEG
Riyadh-3028763	NEG	POS	POS	AMB	POS	AMB	POS	POS	POS	NEG	NEG
Riyadh-2817437	AMB	POS	POS	AMB	POS	AMB	POS	POS	POS	NEG	NEG
Riyadh-2793706	NEG	POS	POS	AMB	POS	AMB	POS	POS	POS	NEG	NEG
Riyadh-2818797	AMB	POS	POS	AMB	POS	AMB	POS	POS	POS	NEG	NEG
Riyadh-2822825	AMB	POS	POS	AMB	POS	AMB	POS	POS	POS	NEG	NEG
Riyadh-2888905	AMB	POS	POS	AMB	POS	AMB	POS	POS	POS	NEG	NEG
Riyadh-2888915	AMB	POS	POS	AMB	POS	AMB	POS	POS	POS	NEG	NEG
Riyadh-2567782	NEG	POS	POS	AMB	POS	AMB	POS	POS	POS	NEG	NEG
Riyadh-2891670	AMB	POS	POS	AMB	POS	AMB	POS	POS	POS	NEG	NEG
Riyadh-3006920	NEG	POS	POS	AMB	POS	AMB	POS	POS	POS	NEG	NEG
Riyadh-2817276-1	AMB	POS	POS	AMB	POS	AMB	POS	POS	POS	NEG	NEG
Riyadh-0295102	NEG	POS	POS	AMB	POS	AMB	POS	POS	POS	NEG	NEG
Riyadh-2820597	AMB	POS	POS	AMB	POS	AMB	POS	POS	POS	NEG	NEG
Riyadh-2822088	AMB	POS	POS	AMB	POS	AMB	POS	POS	POS	NEG	NEG
Riyadh-3010092	NEG	POS	POS	AMB	POS	AMB	POS	POS	POS	NEG	NEG
Riyadh-3022844	NEG	POS	POS	AMB	POS	AMB	POS	POS	POS	NEG	NEG
Riyadh-3108214-2	NEG	POS	POS	AMB	POS	NEG	POS	POS	POS	NEG	NEG
Riyadh-2823926	NEG	POS	POS	AMB	POS	AMB	POS	POS	POS	NEG	NEG
Riyadh-1	AMB	POS	POS	AMB	POS	AMB	POS	POS	POS	NEG	NEG
Riyadh-2818388	NEG	POS	POS	AMB	POS	AMB	POS	POS	POS	NEG	NEG
Riyadh-2818316	NEG	POS	POS	AMB	POS	AMB	POS	POS	POS	NEG	NEG
**CC9/ST834-MRSA-(atypical SCCmec)**											
Riyadh-3103521	POS	AMB	POS	AMB	POS	AMB	POS	POS	POS	NEG	NEG
**CC22-MRSA-IV, Barnim/UK-EMRSA-15**											
Riyadh-2553359	AMB	POS	AMB	POS	POS	AMB	POS	POS	POS	NEG	NEG
Riyadh-2571758	AMB	POS	POS	AMB	POS	AMB	POS	POS	POS	NEG	NEG
Riyadh-3029203	NEG	POS	POS	AMB	POS	AMB	POS	POS	POS	NEG	NEG
Riyadh-3039785	NEG	POS	AMB	POS	POS	AMB	POS	POS	POS	NEG	NEG
Riyadh-3105594	NEG	POS	POS	AMB	POS	AMB	POS	POS	POS	NEG	NEG
Riyadh_IC_204-2	AMB	POS	POS	AMB	POS	AMB	POS	POS	POS	NEG	NEG
Riyadh-3003974	NEG	POS	POS	AMB	POS	AMB	POS	POS	POS	#ZAHL!	NEG
Riyadh_IC_067	NEG	POS	POS	AMB	POS	AMB	POS	POS	POS	NEG	NEG
Riyadh-2988627	AMB	POS	POS	AMB	POS	AMB	POS	POS	POS	NEG	NEG
Riyadh-3112581	NEG	POS	AMB	POS	POS	AMB	POS	POS	POS	NEG	NEG
**CC22-MRSA-IV [PVL+]**											
Riyadh-2781996-1	NEG	POS	POS	AMB	POS	NEG	POS	POS	POS	NEG	NEG
Riyadh-3103432	NEG	POS	POS	AMB	POS	AMB	POS	POS	POS	NEG	NEG
Riyadh-3026502	NEG	POS	AMB	POS	POS	NEG	POS	POS	POS	NEG	NEG
Riyadh-3081378-2	NEG	POS	AMB	POS	POS	AMB	POS	POS	POS	NEG	NEG
Riyadh_IC_185	NEG	POS	POS	AMB	POS	AMB	POS	POS	POS	NEG	NEG
Riyadh_IC_204-1	AMB	POS	POS	AMB	POS	AMB	POS	POS	POS	NEG	NEG
Riyadh-2559371	AMB	POS	POS	AMB	POS	AMB	POS	POS	POS	NEG	NEG
Riyadh-2753975	NEG	POS	POS	AMB	POS	AMB	POS	POS	POS	NEG	NEG
Riyadh-2775605	NEG	POS	POS	POS	POS	NEG	POS	POS	POS	NEG	NEG
Riyadh-2781996-2	NEG	POS	AMB	POS	POS	NEG	POS	POS	POS	NEG	NEG
Riyadh-2823783-2	NEG	POS	POS	POS	POS	AMB	POS	POS	POS	NEG	NEG
Riyadh-2876601	NEG	POS	AMB	POS	POS	AMB	POS	POS	POS	NEG	NEG
Riyadh-3036074	AMB	POS	POS	AMB	POS	AMB	POS	POS	POS	NEG	NEG
Riyadh-3053099	NEG	POS	POS	AMB	POS	AMB	POS	POS	POS	NEG	NEG
Riyadh-3055366	NEG	POS	AMB	POS	POS	AMB	POS	POS	POS	NEG	NEG
Riyadh-3082712	NEG	POS	POS	AMB	POS	AMB	POS	POS	POS	NEG	NEG
Riyadh-3087502	NEG	POS	AMB	POS	POS	NEG	POS	POS	POS	NEG	NEG
Riyadh-6	NEG	POS	AMB	POS	POS	AMB	POS	POS	POS	NEG	NEG
Riyadh-7	AMB	POS	AMB	POS	POS	AMB	POS	POS	POS	NEG	NEG
Riyadh-8	NEG	POS	AMB	POS	POS	AMB	POS	POS	POS	NEG	NEG
**CC30-MRSA-IV [PVL+], Southwest Pacific Clone**											
Riyadh-10	AMB	POS	AMB	POS	POS	POS	NEG	POS	POS	NEG	NEG
Riyadh_IC_123	AMB	POS	POS	AMB	POS	POS	NEG	POS	POS	NEG	NEG
Riyadh-3080713	NEG	POS	AMB	POS	POS	POS	NEG	POS	POS	NEG	NEG
Riyadh-2803856	NEG	POS	POS	AMB	POS	POS	NEG	POS	POS	NEG	NEG
Riyadh-2817276-1	AMB	POS	POS	AMB	POS	POS	AMB	POS	POS	NEG	NEG
Riyadh-2817571-2	AMB	POS	POS	AMB	POS	POS	AMB	POS	POS	NEG	NEG
Riyadh-3033868	NEG	POS	AMB	POS	POS	POS	NEG	POS	POS	NEG	NEG
Riyadh-2550108	NEG	POS	AMB	POS	POS	POS	NEG	POS	POS	NEG	NEG
Riyadh-3095056-2	NEG	POS	AMB	POS	POS	POS	NEG	POS	POS	NEG	NEG
Riyadh-2819899	NEG	POS	AMB	POS	POS	POS	NEG	POS	POS	NEG	NEG
Riyadh-2821805	NEG	POS	AMB	POS	POS	POS	NEG	POS	POS	NEG	NEG
Riyadh-3013928	NEG	POS	POS	AMB	POS	POS	NEG	POS	POS	NEG	NEG
Riyadh-3029402	NEG	POS	POS	AMB	POS	POS	NEG	POS	POS	NEG	NEG
**CC45/agr IV-MRSA-IV, WA MRSA-23**											
Riyadh-3081378-1	NEG	POS	NEG	POS	POS	POS	NEG	NEG	NEG	POS	POS
**CC80-MRSA-IV**											
Riyadh-3107635	POS	AMB	POS	AMB	POS	AMB	POS	POS	POS	NEG	NEG
Riyadh-2987458	POS	AMB	POS	AMB	POS	AMB	POS	POS	POS	NEG	NEG
**CC80-MRSA-IV [PVL+], European caMRSA Clone**											
Riyadh-2988048	POS	AMB	AMB	POS	POS	NEG	POS	POS	POS	NEG	NEG
Riyadh-2990585-2	POS	AMB	POS	AMB	POS	NEG	POS	POS	POS	NEG	NEG
Riyadh-2990585-1	POS	AMB	POS	AMB	POS	AMB	POS	POS	POS	NEG	NEG
Riyadh-2826033	POS	AMB	POS	AMB	POS	AMB	POS	POS	POS	NEG	NEG
Riyadh-1601562	POS	AMB	POS	AMB	POS	AMB	POS	POS	POS	NEG	NEG
Riyadh-2569940	AMB	POS	POS	AMB	POS	AMB	POS	POS	POS	NEG	NEG
Riyadh-2571692	POS	AMB	POS	AMB	POS	AMB	POS	POS	POS	NEG	NEG
Riyadh-2763029	POS	AMB	POS	AMB	POS	AMB	POS	POS	POS	NEG	NEG
Riyadh-2767090	POS	AMB	POS	AMB	POS	AMB	POS	POS	POS	NEG	NEG
Riyadh-2775130	AMB	POS	POS	AMB	POS	NEG	POS	POS	POS	NEG	NEG
Riyadh-2778256	POS	AMB	POS	AMB	POS	AMB	POS	POS	POS	NEG	NEG
Riyadh-2817505	POS	AMB	POS	AMB	POS	AMB	POS	POS	POS	NEG	NEG
Riyadh-3024912	POS	AMB	POS	AMB	POS	AMB	POS	POS	POS	NEG	NEG
Riyadh-2788690	POS	AMB	POS	AMB	POS	AMB	POS	POS	POS	NEG	NEG
Riyadh-2829034	POS	AMB	POS	AMB	POS	AMB	POS	POS	POS	NEG	NEG
Riyadh-3	POS	AMB	POS	AMB	POS	AMB	POS	POS	POS	NEG	NEG
Riyadh-5	POS	AMB	POS	AMB	POS	AMB	POS	POS	POS	NEG	NEG
Riyadh-2553167	POS	AMB	AMB	POS	POS	AMB	POS	POS	POS	NEG	NEG
Riyadh-3002592	POS	AMB	POS	AMB	POS	AMB	POS	POS	POS	NEG	NEG
**CC88-MRSA-IV [PVL+]**											
Riyadh-2736996	POS	AMB	POS	AMB	POS	AMB	POS	POS	POS	NEG	NEG
Riyadh-2942396	POS	AMB	POS	AMB	POS	AMB	POS	POS	POS	NEG	NEG
Riyadh-3105391	POS	AMB	POS	AMB	POS	NEG	POS	POS	POS	NEG	NEG
**CC97-MRSA-V**											
Riyadh-0297622	POS	AMB	POS	AMB	POS	AMB	POS	POS	POS	NEG	NEG
Riyadh-3025471	POS	AMB	POS	AMB	POS	AMB	POS	POS	POS	NEG	NEG

	TYPE I RESTRICTION-MODIFICATION SYSTEM, SINGLE SEQUENCE SPECIFICITY PROTEIN					TYPE I RESTRICTION-MODIFICATION SYSTEM, SINGLE SEQUENCE SPECIFICITY PROTEIN							
	hsdS1 (RF122)	hsdS2 (ST5+ST8)	hsdS2 (MW2+476)	hsdS2 (RF122)	hsdS2 (MRSA252)	hsdS3 (all other than RF122+ MRSA252)	hsdS3 (ST8+ST1+RF122)	hsdS3 (Mu50+ N315)	hsdS3 (CC51+ MRSA252)	hsdS3 (MRSA252)	hsdSx (CC25)	hsdSx (CC15)	hsdSx (etd)
	type I site-specific deoxyribo-nuclease subunit, 1st locus	type I site-specific deoxyribonuclease subunit, 2nd locus				type I site-specific deoxyribonuclease subunit, 3rd locus					type I site-specific deoxyribonuclease subunit, unknown locus		
**CC1-MRSA-IV&SCCfus, WA MRSA-1/45**													
Riyadh-3108609	NEG	NEG	POS	NEG	NEG	POS	POS	NEG	NEG	NEG	POS	NEG	NEG
**CC1/ST772-MRSA-V [PVL+], "Bengal Bay Clone/WA I**													
Riyadh-2819026	NEG	NEG	POS	NEG	NEG	POS	NEG	NEG	NEG	NEG	POS	POS	NEG
**CC5-MRSA-IV, Paediatric clone**													
Riyadh-2915327-1	NEG	POS	AMB	NEG	NEG	POS	NEG	POS	NEG	NEG	POS	AMB	NEG
Riyadh-2915327-2	NEG	POS	AMB	NEG	NEG	POS	NEG	POS	NEG	NEG	POS	NEG	NEG
Riyadh-2	NEG	NEG	AMB	NEG	NEG	POS	NEG	POS	AMB	NEG	POS	AMB	NEG
**CC5-MRSA-IV [PVL+], Paediatric clone**													
Riyadh-2986666	NEG	POS	AMB	NEG	NEG	POS	NEG	POS	NEG	NEG	POS	AMB	NEG
Riyadh-2913335	NEG	POS	AMB	NEG	NEG	POS	NEG	POS	NEG	NEG	POS	AMB	NEG
**CC5-MRSA-IVvar, "Maltese Clone"**													
Riyadh-2983654	NEG	POS	AMB	NEG	NEG	POS	NEG	POS	NEG	NEG	POS	AMB	NEG
Riyadh-4	NEG	POS	AMB	NEG	NEG	POS	NEG	POS	NEG	NEG	POS	NEG	NEG
Riyadh-2790233	NEG	POS	AMB	NEG	NEG	POS	NEG	POS	NEG	NEG	POS	AMB	NEG
**CC5-MRSA-V**													
Riyadh-2568944	NEG	POS	AMB	NEG	NEG	POS	NEG	POS	NEG	NEG	POS	AMB	NEG
**CC6-MRSA-IV, WA MRSA-51/66**													
Riyadh-2556168	NEG	NEG	NEG	NEG	NEG	POS	POS	NEG	POS	NEG	POS	AMB	NEG
Riyadh-2824507	NEG	NEG	AMB	NEG	NEG	POS	POS	NEG	POS	NEG	POS	AMB	NEG
Riyadh-2990831	NEG	NEG	POS	NEG	NEG	POS	POS	NEG	POS	NEG	POS	AMB	NEG
**CC8/ST239-MRSA-III, Vienna/Hungarian/Brazilian Cl**													
Riyadh-9	NEG	NEG	AMB	NEG	NEG	POS	POS	NEG	NEG	NEG	POS	NEG	NEG
Riyadh-3028763	NEG	POS	POS	NEG	NEG	POS	POS	NEG	NEG	NEG	POS	NEG	NEG
Riyadh-2817437	NEG	POS	POS	NEG	NEG	POS	POS	NEG	NEG	NEG	POS	NEG	NEG
Riyadh-2793706	NEG	POS	AMB	NEG	NEG	POS	POS	NEG	NEG	NEG	POS	NEG	NEG
Riyadh-2818797	NEG	POS	POS	NEG	NEG	POS	POS	NEG	NEG	NEG	POS	AMB	NEG
Riyadh-2822825	NEG	POS	AMB	NEG	NEG	POS	POS	NEG	NEG	NEG	POS	NEG	NEG
Riyadh-2888905	NEG	POS	AMB	NEG	NEG	POS	POS	NEG	NEG	NEG	POS	NEG	NEG
Riyadh-2888915	NEG	POS	POS	NEG	NEG	POS	POS	NEG	NEG	NEG	POS	NEG	NEG
Riyadh-2567782	NEG	POS	AMB	NEG	NEG	POS	POS	NEG	NEG	NEG	POS	NEG	NEG
Riyadh-2891670	NEG	POS	POS	NEG	NEG	POS	POS	NEG	NEG	NEG	POS	NEG	NEG
Riyadh-3006920	NEG	POS	AMB	NEG	NEG	POS	POS	NEG	NEG	NEG	POS	NEG	NEG
Riyadh-2817276-1	NEG	POS	POS	NEG	NEG	POS	POS	NEG	NEG	NEG	POS	NEG	NEG
Riyadh-0295102	NEG	POS	AMB	NEG	NEG	POS	POS	NEG	NEG	NEG	POS	NEG	NEG
Riyadh-2820597	NEG	POS	POS	NEG	NEG	POS	POS	NEG	NEG	NEG	POS	NEG	NEG
Riyadh-2822088	NEG	POS	POS	NEG	NEG	POS	POS	NEG	NEG	NEG	POS	NEG	NEG
Riyadh-3010092	NEG	POS	AMB	NEG	NEG	POS	POS	NEG	NEG	NEG	POS	NEG	NEG
Riyadh-3022844	NEG	POS	AMB	NEG	NEG	POS	POS	NEG	NEG	NEG	POS	NEG	NEG
Riyadh-3108214-2	NEG	POS	AMB	NEG	NEG	POS	POS	NEG	NEG	NEG	POS	NEG	NEG
Riyadh-2823926	NEG	POS	POS	NEG	NEG	POS	POS	NEG	NEG	NEG	POS	NEG	NEG
Riyadh-1	NEG	POS	AMB	NEG	NEG	POS	POS	NEG	NEG	NEG	POS	NEG	NEG
Riyadh-2818388	NEG	POS	POS	NEG	NEG	POS	POS	NEG	NEG	NEG	POS	NEG	NEG
Riyadh-2818316	NEG	POS	AMB	NEG	NEG	POS	POS	NEG	NEG	NEG	POS	NEG	NEG
**CC9/ST834-MRSA-(atypical SCCmec)**													
Riyadh-3103521	NEG	NEG	POS	NEG	NEG	NEG	NEG	NEG	NEG	NEG	NEG	NEG	NEG
**CC22-MRSA-IV, Barnim/UK-EMRSA-15**													
Riyadh-2553359	NEG	POS	NEG	NEG	NEG	AMB	NEG	NEG	NEG	NEG	POS	AMB	NEG
Riyadh-2571758	NEG	POS	AMB	NEG	NEG	POS	NEG	NEG	NEG	NEG	POS	NEG	NEG
Riyadh-3029203	NEG	POS	POS	NEG	NEG	AMB	NEG	NEG	NEG	NEG	POS	AMB	NEG
Riyadh-3039785	NEG	POS	POS	NEG	NEG	AMB	NEG	NEG	NEG	NEG	POS	AMB	NEG
Riyadh-3105594	NEG	POS	AMB	NEG	NEG	AMB	NEG	NEG	NEG	NEG	POS	NEG	NEG
Riyadh_IC_204-2	NEG	POS	AMB	NEG	NEG	POS	NEG	NEG	NEG	NEG	POS	AMB	NEG
Riyadh-3003974	NEG	POS	POS	NEG	NEG	POS	NEG	NEG	NEG	NEG	POS	AMB	NEG
Riyadh_IC_067	NEG	POS	AMB	NEG	NEG	POS	NEG	NEG	NEG	NEG	POS	AMB	NEG
Riyadh-2988627	NEG	POS	AMB	NEG	NEG	AMB	NEG	NEG	NEG	NEG	POS	AMB	NEG
Riyadh-3112581	NEG	POS	AMB	NEG	NEG	NEG	NEG	NEG	NEG	NEG	POS	NEG	NEG
**CC22-MRSA-IV [PVL+]**													
Riyadh-2781996-1	NEG	POS	AMB	NEG	NEG	NEG	NEG	NEG	NEG	NEG	POS	AMB	NEG
Riyadh-3103432	NEG	POS	AMB	NEG	NEG	AMB	NEG	NEG	NEG	NEG	POS	NEG	NEG
Riyadh-3026502	NEG	POS	NEG	NEG	NEG	NEG	NEG	NEG	NEG	NEG	AMB	AMB	NEG
Riyadh-3081378-2	NEG	POS	NEG	NEG	NEG	AMB	NEG	NEG	NEG	NEG	POS	NEG	NEG
Riyadh_IC_185	NEG	POS	AMB	NEG	NEG	POS	NEG	NEG	NEG	NEG	POS	AMB	NEG
Riyadh_IC_204-1	NEG	POS	AMB	NEG	NEG	POS	NEG	NEG	NEG	NEG	POS	AMB	NEG
Riyadh-2559371	NEG	POS	NEG	NEG	NEG	POS	NEG	NEG	NEG	NEG	POS	AMB	NEG
Riyadh-2753975	NEG	POS	AMB	NEG	NEG	AMB	NEG	NEG	NEG	NEG	POS	AMB	NEG
Riyadh-2775605	NEG	POS	AMB	NEG	NEG	NEG	NEG	NEG	NEG	NEG	POS	NEG	NEG
Riyadh-2781996-2	NEG	POS	AMB	NEG	NEG	NEG	NEG	NEG	NEG	NEG	POS	NEG	NEG
Riyadh-2823783-2	NEG	POS	AMB	NEG	NEG	POS	NEG	NEG	NEG	NEG	POS	AMB	NEG
Riyadh-2876601	NEG	POS	AMB	NEG	NEG	AMB	NEG	NEG	NEG	NEG	POS	AMB	NEG
Riyadh-3036074	NEG	POS	POS	NEG	NEG	POS	NEG	NEG	NEG	NEG	POS	AMB	NEG
Riyadh-3053099	NEG	POS	AMB	NEG	NEG	AMB	NEG	NEG	NEG	NEG	POS	AMB	NEG
Riyadh-3055366	NEG	POS	AMB	NEG	NEG	NEG	NEG	NEG	NEG	NEG	POS	AMB	NEG
Riyadh-3082712	NEG	POS	POS	NEG	NEG	AMB	NEG	NEG	NEG	NEG	POS	AMB	NEG
Riyadh-3087502	NEG	POS	NEG	NEG	NEG	NEG	NEG	NEG	NEG	NEG	AMB	NEG	NEG
Riyadh-6	NEG	POS	NEG	NEG	NEG	AMB	NEG	NEG	NEG	NEG	POS	AMB	NEG
Riyadh-7	NEG	POS	NEG	NEG	NEG	AMB	NEG	NEG	NEG	NEG	POS	AMB	NEG
Riyadh-8	NEG	POS	NEG	NEG	NEG	AMB	NEG	NEG	NEG	NEG	POS	AMB	NEG
**CC30-MRSA-IV [PVL+], Southwest Pacific Clone**													
Riyadh-10	NEG	NEG	NEG	NEG	POS	NEG	NEG	NEG	POS	POS	POS	AMB	NEG
Riyadh_IC_123	NEG	NEG	AMB	NEG	POS	NEG	NEG	NEG	POS	POS	POS	NEG	NEG
Riyadh-3080713	NEG	NEG	AMB	NEG	POS	NEG	NEG	NEG	POS	POS	POS	AMB	NEG
Riyadh-2803856	NEG	NEG	POS	NEG	POS	AMB	NEG	NEG	POS	POS	POS	AMB	NEG
Riyadh-2817276-1	NEG	NEG	POS	NEG	POS	POS	NEG	NEG	POS	POS	POS	AMB	NEG
Riyadh-2817571-2	NEG	NEG	POS	NEG	POS	AMB	NEG	NEG	POS	POS	POS	NEG	NEG
Riyadh-3033868	NEG	NEG	AMB	NEG	POS	NEG	NEG	NEG	POS	POS	POS	NEG	NEG
Riyadh-2550108	NEG	NEG	NEG	NEG	POS	NEG	NEG	NEG	POS	POS	POS	AMB	NEG
Riyadh-3095056-2	NEG	NEG	AMB	NEG	POS	NEG	NEG	NEG	POS	POS	POS	NEG	NEG
Riyadh-2819899	NEG	NEG	POS	NEG	POS	NEG	NEG	NEG	POS	POS	POS	AMB	NEG
Riyadh-2821805	NEG	NEG	NEG	NEG	POS	NEG	NEG	NEG	POS	POS	POS	NEG	NEG
Riyadh-3013928	NEG	NEG	AMB	NEG	POS	NEG	NEG	NEG	POS	POS	POS	AMB	NEG
Riyadh-3029402	NEG	NEG	POS	NEG	POS	NEG	NEG	NEG	POS	POS	POS	AMB	NEG
**CC45/agr IV-MRSA-IV, WA MRSA-23**													
Riyadh-3081378-1	NEG	NEG	NEG	NEG	POS	POS	NEG	NEG	POS	NEG	AMB	AMB	NEG
**CC80-MRSA-IV**													
Riyadh-3107635	NEG	NEG	POS	NEG	NEG	NEG	NEG	NEG	NEG	NEG	NEG	AMB	POS
Riyadh-2987458	NEG	NEG	AMB	NEG	NEG	NEG	NEG	NEG	NEG	NEG	AMB	AMB	POS
**CC80-MRSA-IV [PVL+], European caMRSA Clone**													
Riyadh-2988048	NEG	NEG	POS	NEG	NEG	NEG	NEG	NEG	NEG	NEG	NEG	NEG	POS
Riyadh-2990585-2	NEG	NEG	AMB	NEG	NEG	NEG	NEG	NEG	NEG	NEG	NEG	AMB	POS
Riyadh-2990585-1	NEG	NEG	AMB	NEG	NEG	NEG	NEG	NEG	NEG	NEG	AMB	AMB	POS
Riyadh-2826033	NEG	NEG	AMB	NEG	NEG	NEG	NEG	NEG	NEG	NEG	AMB	AMB	POS
Riyadh-1601562	NEG	NEG	AMB	NEG	NEG	NEG	NEG	NEG	NEG	NEG	NEG	NEG	POS
Riyadh-2569940	NEG	NEG	POS	NEG	NEG	NEG	NEG	NEG	NEG	NEG	POS	POS	POS
Riyadh-2571692	NEG	NEG	POS	NEG	NEG	NEG	NEG	NEG	NEG	NEG	POS	POS	POS
Riyadh-2763029	NEG	NEG	AMB	NEG	NEG	NEG	NEG	NEG	NEG	NEG	AMB	POS	POS
Riyadh-2767090	NEG	NEG	AMB	NEG	NEG	NEG	NEG	NEG	NEG	NEG	NEG	AMB	POS
Riyadh-2775130	NEG	NEG	AMB	NEG	NEG	NEG	NEG	NEG	NEG	NEG	NEG	AMB	POS
Riyadh-2778256	NEG	NEG	POS	NEG	NEG	NEG	NEG	NEG	NEG	NEG	NEG	AMB	POS
Riyadh-2817505	NEG	NEG	POS	NEG	NEG	NEG	NEG	NEG	NEG	NEG	AMB	AMB	POS
Riyadh-3024912	NEG	NEG	AMB	NEG	NEG	NEG	NEG	NEG	NEG	NEG	NEG	AMB	POS
Riyadh-2788690	NEG	NEG	AMB	NEG	NEG	NEG	NEG	NEG	NEG	NEG	AMB	AMB	POS
Riyadh-2829034	NEG	NEG	AMB	NEG	NEG	NEG	NEG	NEG	NEG	NEG	POS	AMB	POS
Riyadh-3	NEG	NEG	AMB	NEG	NEG	NEG	NEG	NEG	NEG	NEG	AMB	POS	POS
Riyadh-5	NEG	NEG	POS	NEG	NEG	NEG	NEG	NEG	NEG	NEG	POS	POS	POS
Riyadh-2553167	NEG	NEG	AMB	NEG	NEG	NEG	NEG	NEG	NEG	NEG	AMB	AMB	POS
Riyadh-3002592	NEG	NEG	AMB	NEG	NEG	NEG	NEG	NEG	NEG	NEG	AMB	AMB	POS
**CC88-MRSA-IV [PVL+]**													
Riyadh-2736996	POS	NEG	AMB	NEG	NEG	POS	NEG	NEG	NEG	POS	POS	AMB	NEG
Riyadh-2942396	POS	NEG	AMB	NEG	NEG	POS	NEG	NEG	NEG	POS	POS	AMB	NEG
Riyadh-3105391	POS	NEG	AMB	NEG	NEG	POS	NEG	NEG	NEG	POS	POS	NEG	NEG
**CC97-MRSA-V**													
Riyadh-0297622	NEG	NEG	AMB	NEG	NEG	AMB	NEG	NEG	NEG	NEG	POS	AMB	NEG
Riyadh-3025471	NEG	NEG	AMB	NEG	NEG	AMB	NEG	NEG	AMB	NEG	POS	NEG	NEG

	MISCELLANEOUS GENES			HYALURONATE LYASE							
	Q2FXC0	Q2YUB3	Q7A4X2	hysA1 (MRSA252)	hysA1 (MRSA252+RF122) and/or hysA2 (cons)	hysA1 (MRSA252+RF122) and/or hysA2 [illegible]	hysA2 (all other than MRSA252)	hysA2 (COL+USA300+NCTC8325)	hysA2 (all other than COL+USA300+NCTC8325)	hysA2 (all other than COL+USA300+NCTC8325)	hysA2 (MRSA252)
	hypothetical protein, located next to serine protease operon	Unspecific efflux/trans-porter	hypothetical protein	hyaluronate lyase, first / second locus			hyaluronate lyase, second locus				
**CC1-MRSA-IV&SCCfus, WA MRSA-1/45**											
Riyadh-3108609	POS	NEG	NEG	NEG	POS	NEG	POS	NEG	POS	POS	NEG
**CC1/ST772-MRSA-V [PVL+], "Bengal Bay Clone/WA I**											
Riyadh-2819026	NEG	POS	POS	NEG	POS	NEG	POS	NEG	POS	POS	NEG
**CC5-MRSA-IV, Paediatric clone**											
Riyadh-2915327-1	NEG	AMB	POS	NEG	POS	NEG	POS	NEG	POS	POS	AMB
Riyadh-2915327-2	NEG	NEG	POS	NEG	POS	NEG	POS	NEG	POS	POS	NEG
Riyadh-2	NEG	NEG	POS	NEG	POS	NEG	POS	NEG	POS	POS	AMB
**CC5-MRSA-IV [PVL+], Paediatric clone**											
Riyadh-2986666	NEG	AMB	POS	NEG	POS	NEG	POS	NEG	POS	POS	AMB
Riyadh-2913335	NEG	AMB	POS	NEG	POS	NEG	POS	NEG	POS	POS	NEG
**CC5-MRSA-IVvar, "Maltese Clone"**											
Riyadh-2983654	NEG	AMB	POS	NEG	POS	NEG	POS	NEG	POS	POS	AMB
Riyadh-4	NEG	NEG	POS	NEG	POS	NEG	POS	NEG	POS	POS	NEG
Riyadh-2790233	NEG	AMB	POS	NEG	POS	NEG	POS	NEG	POS	POS	NEG
**CC5-MRSA-V**											
Riyadh-2568944	NEG	NEG	POS	NEG	POS	NEG	POS	NEG	POS	POS	NEG
**CC6-MRSA-IV, WA MRSA-51/66**											
Riyadh-2556168	POS	NEG	NEG	NEG	POS	NEG	NEG	NEG	POS	POS	NEG
Riyadh-2824507	POS	NEG	NEG	NEG	POS	NEG	NEG	NEG	POS	POS	NEG
Riyadh-2990831	POS	POS	NEG	NEG	POS	NEG	NEG	NEG	POS	POS	NEG
**CC8/ST239-MRSA-III, Vienna/Hungarian/Brazilian Cl**											
Riyadh-9	POS	NEG	NEG	NEG	POS	POS	POS	POS	NEG	NEG	AMB
Riyadh-3028763	POS	AMB	NEG	NEG	POS	POS	POS	POS	NEG	NEG	AMB
Riyadh-2817437	POS	AMB	NEG	NEG	POS	POS	POS	POS	NEG	NEG	AMB
Riyadh-2793706	POS	NEG	NEG	NEG	POS	POS	POS	POS	NEG	NEG	NEG
Riyadh-2818797	POS	POS	NEG	NEG	POS	POS	POS	POS	NEG	NEG	NEG
Riyadh-2822825	POS	POS	NEG	NEG	POS	POS	POS	POS	NEG	NEG	POS
Riyadh-2888905	POS	AMB	NEG	NEG	POS	POS	POS	POS	NEG	NEG	AMB
Riyadh-2888915	POS	AMB	NEG	NEG	POS	POS	POS	POS	NEG	NEG	NEG
Riyadh-2567782	POS	NEG	NEG	NEG	POS	POS	POS	POS	NEG	NEG	NEG
Riyadh-2891670	POS	AMB	NEG	NEG	POS	POS	POS	POS	NEG	NEG	NEG
Riyadh-3006920	POS	AMB	NEG	NEG	POS	POS	POS	POS	NEG	NEG	AMB
Riyadh-2817276-1	POS	AMB	NEG	NEG	POS	POS	POS	POS	NEG	NEG	AMB
Riyadh-0295102	POS	NEG	NEG	NEG	POS	POS	POS	POS	NEG	NEG	NEG
Riyadh-2820597	POS	POS	NEG	NEG	POS	POS	POS	POS	NEG	NEG	NEG
Riyadh-2822088	POS	POS	NEG	NEG	POS	POS	POS	POS	NEG	NEG	AMB
Riyadh-3010092	POS	AMB	NEG	NEG	POS	POS	POS	POS	NEG	NEG	NEG
Riyadh-3022844	POS	AMB	NEG	NEG	POS	POS	POS	POS	NEG	NEG	NEG
Riyadh-3108214-2	POS	NEG	NEG	NEG	POS	POS	POS	POS	NEG	NEG	NEG
Riyadh-2823926	POS	POS	NEG	NEG	POS	POS	POS	POS	NEG	NEG	POS
Riyadh-1	POS	AMB	NEG	NEG	POS	POS	POS	POS	NEG	NEG	POS
Riyadh-2818388	POS	POS	NEG	NEG	POS	POS	POS	POS	NEG	NEG	NEG
Riyadh-2818316	POS	AMB	NEG	NEG	POS	POS	POS	POS	NEG	NEG	AMB
**CC9/ST834-MRSA-(atypical SCCmec)**											
Riyadh-3103521	NEG	NEG	NEG	NEG	POS	NEG	NEG	NEG	POS	POS	NEG
**CC22-MRSA-IV, Barnim/UK-EMRSA-15**											
Riyadh-2553359	NEG	AMB	POS	NEG	POS	NEG	POS	NEG	POS	POS	POS
Riyadh-2571758	NEG	AMB	POS	NEG	POS	NEG	POS	NEG	POS	POS	POS
Riyadh-3029203	NEG	AMB	POS	NEG	POS	NEG	AMB	NEG	POS	POS	POS
Riyadh-3039785	NEG	AMB	POS	NEG	POS	NEG	AMB	NEG	POS	POS	POS
Riyadh-3105594	NEG	AMB	POS	NEG	POS	NEG	AMB	NEG	POS	POS	POS
Riyadh_IC_204-2	NEG	POS	POS	NEG	POS	NEG	AMB	NEG	POS	POS	POS
Riyadh-3003974	NEG	POS	POS	NEG	POS	NEG	AMB	NEG	POS	POS	POS
Riyadh_IC_067	NEG	AMB	POS	NEG	POS	NEG	AMB	NEG	POS	POS	POS
Riyadh-2988627	NEG	AMB	POS	NEG	POS	NEG	AMB	NEG	POS	POS	AMB
Riyadh-3112581	NEG	NEG	POS	NEG	POS	NEG	AMB	NEG	POS	POS	POS
**CC22-MRSA-IV [PVL+]**											
Riyadh-2781996-1	NEG	NEG	POS	NEG	POS	NEG	AMB	NEG	POS	POS	AMB
Riyadh-3103432	NEG	AMB	POS	NEG	POS	NEG	NEG	NEG	POS	POS	POS
Riyadh-3026502	NEG	NEG	POS	NEG	POS	NEG	NEG	NEG	POS	POS	NEG
Riyadh-3081378-2	NEG	AMB	POS	NEG	POS	NEG	NEG	NEG	POS	POS	AMB
Riyadh_IC_185	NEG	AMB	POS	NEG	POS	NEG	POS	NEG	POS	POS	POS
Riyadh_IC_204-1	NEG	AMB	POS	NEG	POS	NEG	POS	NEG	POS	POS	POS
Riyadh-2559371	NEG	AMB	POS	NEG	POS	NEG	POS	NEG	POS	POS	POS
Riyadh-2753975	NEG	AMB	POS	NEG	POS	NEG	AMB	NEG	POS	POS	POS
Riyadh-2775605	NEG	NEG	POS	NEG	POS	NEG	NEG	NEG	POS	POS	POS
Riyadh-2781996-2	NEG	NEG	POS	NEG	POS	NEG	NEG	NEG	POS	POS	AMB
Riyadh-2823783-2	NEG	AMB	POS	NEG	POS	NEG	POS	NEG	POS	POS	POS
Riyadh-2876601	NEG	AMB	POS	NEG	POS	NEG	NEG	NEG	POS	POS	POS
Riyadh-3036074	NEG	POS	POS	NEG	POS	NEG	POS	NEG	POS	POS	POS
Riyadh-3053099	NEG	AMB	POS	NEG	POS	NEG	AMB	NEG	POS	POS	POS
Riyadh-3055366	NEG	AMB	POS	NEG	POS	NEG	AMB	NEG	POS	POS	POS
Riyadh-3082712	NEG	AMB	POS	NEG	POS	NEG	AMB	NEG	POS	POS	POS
Riyadh-3087502	NEG	NEG	POS	NEG	POS	NEG	NEG	NEG	POS	POS	NEG
Riyadh-6	NEG	NEG	POS	NEG	POS	NEG	POS	NEG	POS	POS	POS
Riyadh-7	NEG	NEG	POS	NEG	POS	NEG	POS	NEG	POS	POS	POS
Riyadh-8	NEG	NEG	POS	NEG	POS	NEG	POS	NEG	POS	POS	POS
**CC30-MRSA-IV [PVL+], Southwest Pacific Clone**											
Riyadh-10	NEG	NEG	POS	POS	POS	POS	NEG	POS	POS	POS	POS
Riyadh_IC_123	NEG	AMB	POS	POS	POS	POS	NEG	POS	POS	POS	POS
Riyadh-3080713	NEG	AMB	POS	POS	POS	POS	NEG	POS	POS	POS	POS
Riyadh-2803856	NEG	AMB	POS	POS	POS	POS	NEG	POS	POS	POS	POS
Riyadh-2817276-1	NEG	POS	POS	POS	POS	POS	NEG	POS	POS	POS	POS
Riyadh-2817571-2	NEG	POS	POS	POS	POS	POS	NEG	POS	POS	POS	POS
Riyadh-3033868	NEG	NEG	POS	POS	POS	POS	NEG	POS	POS	POS	POS
Riyadh-2550108	NEG	NEG	POS	POS	POS	POS	NEG	POS	POS	POS	POS
Riyadh-3095056-2	NEG	NEG	POS	POS	POS	POS	NEG	POS	POS	POS	POS
Riyadh-2819899	NEG	NEG	POS	POS	POS	POS	NEG	POS	POS	POS	POS
Riyadh-2821805	NEG	NEG	POS	POS	POS	POS	NEG	POS	POS	POS	POS
Riyadh-3013928	NEG	AMB	POS	POS	POS	POS	NEG	POS	POS	POS	POS
Riyadh-3029402	NEG	AMB	POS	POS	POS	POS	NEG	POS	POS	POS	POS
**CC45/agr IV-MRSA-IV, WA MRSA-23**											
Riyadh-3081378-1	NEG	NEG	POS	NEG	POS	POS	AMB	POS	NEG	NEG	NEG
**CC80-MRSA-IV**											
Riyadh-3107635	NEG	AMB	NEG	NEG	POS	NEG	POS	NEG	POS	POS	POS
Riyadh-2987458	NEG	AMB	NEG	NEG	POS	NEG	POS	NEG	POS	POS	POS
**CC80-MRSA-IV [PVL+], European caMRSA Clone**											
Riyadh-2988048	NEG	NEG	NEG	NEG	POS	NEG	POS	NEG	POS	POS	POS
Riyadh-2990585-2	NEG	NEG	NEG	NEG	POS	NEG	POS	NEG	POS	POS	POS
Riyadh-2990585-1	NEG	AMB	NEG	NEG	POS	NEG	POS	NEG	POS	POS	POS
Riyadh-2826033	NEG	AMB	NEG	NEG	POS	NEG	POS	NEG	POS	POS	POS
Riyadh-1601562	NEG	NEG	NEG	NEG	POS	NEG	POS	NEG	POS	POS	POS
Riyadh-2569940	NEG	AMB	NEG	NEG	POS	NEG	POS	NEG	POS	POS	POS
Riyadh-2571692	NEG	NEG	NEG	NEG	POS	NEG	POS	NEG	POS	POS	POS
Riyadh-2763029	NEG	AMB	NEG	NEG	POS	NEG	POS	NEG	POS	POS	POS
Riyadh-2767090	NEG	AMB	NEG	NEG	POS	NEG	POS	NEG	POS	POS	POS
Riyadh-2775130	NEG	NEG	NEG	NEG	POS	NEG	POS	NEG	POS	POS	AMB
Riyadh-2778256	NEG	AMB	NEG	NEG	POS	NEG	POS	NEG	POS	POS	POS
Riyadh-2817505	NEG	AMB	NEG	NEG	POS	NEG	POS	NEG	POS	POS	POS
Riyadh-3024912	NEG	NEG	NEG	NEG	POS	NEG	POS	NEG	POS	POS	POS
Riyadh-2788690	NEG	AMB	NEG	NEG	POS	NEG	POS	NEG	POS	POS	POS
Riyadh-2829034	NEG	AMB	NEG	NEG	POS	NEG	POS	NEG	POS	POS	POS
Riyadh-3	NEG	NEG	NEG	NEG	POS	NEG	POS	NEG	POS	POS	POS
Riyadh-5	NEG	AMB	NEG	NEG	POS	NEG	POS	NEG	POS	POS	POS
Riyadh-2553167	NEG	NEG	NEG	NEG	POS	NEG	POS	NEG	POS	POS	POS
Riyadh-3002592	NEG	AMB	NEG	NEG	POS	NEG	POS	NEG	POS	POS	AMB
**CC88-MRSA-IV [PVL+]**											
Riyadh-2736996	NEG	AMB	NEG	NEG	POS	NEG	POS	NEG	POS	POS	NEG
Riyadh-2942396	NEG	AMB	NEG	NEG	POS	NEG	POS	NEG	POS	POS	AMB
Riyadh-3105391	NEG	NEG	NEG	NEG	POS	NEG	POS	NEG	POS	POS	NEG
**CC97-MRSA-V**											
Riyadh-0297622	NEG	NEG	POS	NEG	POS	NEG	NEG	NEG	POS	POS	NEG
Riyadh-3025471	NEG	AMB	POS	NEG	POS	NEG	POS	NEG	POS	POS	POS
